# Taxonomic revision of the New World members of the trapdoor spider genus *Ummidia* Thorell (Araneae, Mygalomorphae, Halonoproctidae)

**DOI:** 10.3897/zookeys.1027.54888

**Published:** 2021-04-02

**Authors:** Rebecca L. Godwin, Jason E. Bond

**Affiliations:** 1 Department of Entomology and Nematology, University of California, Davis 1 Shields Ave, Davis, CA, 95616 University of California Davis United States of America

**Keywords:** Biodiversity, Halonoproctidae, Mygalomorphae, new species, spider taxonomy, *
Ummidia
*

## Abstract

This study documents a comprehensive taxonomic treatment of the New World *Ummidia* species. At the onset of this work the genus comprised 27 species and one subspecies with a cosmopolitan distribution that includes North America, South America, Asia, northern Africa, and Europe; of these species the majority of the nominal diversity can be attributed to the New World where 20 species have been previously described. *Ummidia oaxacana* (Chamberlin, 1925) is considered a *nomen dubium*; *U. tuobita* (Chamberlin, 1917) and *U. absoluta* (Gertsch and Mulaik, 1940) are both considered junior synonyms of *U. audouini* (Lucas, 1835); the subspecies *U. carabivora emarginata* (Atkinson, 1886) is considered a junior synonym of *U. carabivora* (Atkinson, 1886); *U. pygmaea* (Chamberlin and Ivie, 1945) is considered a junior synonym of *U. beatula* (Gertsch and Mulaik, 1940); *U. celsa* (Gertsch and Mulaik, 1940) is considered a junior synonym of *U. funerea* (Gertsch, 1936); *Hebestatis lanthanus* (Valerio, 1987) is considered a junior synonym of *U. rugosa* (Karsch, 1880). Thirty-three new species are described: *U. neilgaimani*, *U. gingoteague*, *U. rongodwini*, *U. okefenokee*, *U. richmond*, *U. macarthuri*, *U. colemanae*, *U. rosillos*, *U. mercedesburnsae*, *U. paulacushingae*, *U. waunekaae*, *U. gertschi*, *U. timcotai*, *U. gabrieli*, *U. pesiou*, *U. rodeo*, *U. huascazaloya*, *U. anaya*, *U. cuicatec*, *U. brandicarlileae*, *U. riverai*, *U. frankellerae*, *U. hondurena*, *U. yojoa*, *U. matagalpa*, *U. carlosviquezi*, *U. varablanca*, *U. quepoa*, *U. cerrohoya*, *U. quijichacaca*, *U. tibacuy*, *U. neblina*, *U. tunapuna*.

## Introduction

The trapdoor spider genus *Ummidia* (Mygalomorphae: Halonoproctidae) has long been recognized as being in serious need of taxonomic treatment. At the beginning of this study, *Ummidia* contained 27 nominal species, 20 of which are found in the New World. *Ummidia asperula* (Simon, 1889) and *U. glabra* (Doleschall in Ausserer, 1871) are found in Venezuela and Brazil, respectively. *Ummidia salebrosa* (Simon, 1891b) is described from St. Vincent, and *U. nidulans* (Fabricius, 1787) from the West Indies. Six species are described from Mexico and Central America: *U. erema* (Chamberlin, 1925), *U. oaxacana* (Chamberlin, 1925), *U. pustulosa* (Becker, 1879), *U. rugosa* (Karsch, 1880), *U. zebrina* (Pickard-Cambridge, 1897), and *U. zilchi* (Kraus, 1955). In the United States there are ten nominal species, *U. absoluta* (Gertsch and Mulaik, 1940), *U. audouini* (Lucas, 1835), *U. beatula* (Gertsch and Mulaik, 1940), *U. carabivora* (Atkinson, 1886), *U. carabivora emarginata* (Atkinson, 1886), *U. celsa* (Gertsch and Mulaik, 1940), *U. funerea* (Gertsch, 1936), *U. modesta* (Banks, 1901), *U. pygmaea* (Chamberlin and Ivie, 1945), and *U. tuobita* (Chamberlin, 1917), which range from as far north as Maryland and Ohio, south to Florida and to Arizona in the west. Unfortunately, the descriptions for these species (Ausserer 1875, Atkinson 1886, Gertsch and Mulaik 1940) are either vague or utilize characters that are not necessarily useful in species diagnosis. In short, taxonomic work on New World *Ummidia* has been sparse aside from these original descriptions, the most recent of which are over half a century old. Despite these shortcomings the genus has long captured the imaginations of spider taxonomists due to its putative remarkable diversity, recognized as potentially containing as many as 50 undescribed North American species (Hendrixson and Bond 2005).

Since Raven’s (1985) revision of all mygalomorph spider genera, the taxonomic composition of the family Ctenizidae and consequent placement of *Ummidia* has remained problematic, particularly with the application of molecular approaches to resolving relationships (Hedin and Bond 2006, Bond et al. 2012). Historically a member of the Ctenizidae until genomic data revealed that the family (*sensu lato*) comprised genera belonging to three different independent lineages (Godwin et al. 2018), *Ummidia* is now placed within the family Halonoproctidae (Pocock, 1901), subfamily Ummidiinae (Ortiz, 2007) which also contains *Latouchia* (Pocock, 1901) and *Conothele* (Thorell, 1878). Likewise, whether or not *Ummidia* and *Conothele* constitute independent genera has been the subject of long-standing debate (Main 1998, Decae 2010, Opatova et al. 2013). The genera are morphologically similar but found in different regions, with *Ummidia* occurring in Central Asia, the Mediterranean, and Americas and *Conothele* in Australia and southeast Asia. Given their apparent morphological similarity Decae (2010) and Main (1998) concluded that the genera should be synonymized, but phylogenetic analyses using hundreds of loci and extensive sampling of both genera from across their ranges showed conclusively that the genera form two independent evolutionary lineages (Godwin et al. 2018). A more recent study which placed these genera in the context of all Mygalomorphae estimated the divergence time of the two lineages at just over 50 mya which is comparable to generic level divergences for other mygalomorph taxa (Opatova et al. 2020). A second relatively recent phylogenetic study by Opatova et al. (2013) also shows that the North American *Ummidia* species form a monophyletic group exclusive of the Old World taxa.

*Ummidia* are medium-sized trapdoor spiders that construct silk-lined burrows, usually with cork-type doors (Bond and Coyle 1995). These burrows are cryptic and difficult to find and are often covered in leaf litter (Gertsch and Wallace 1936, Coyle 1981, Bond and Coyle 1995). Ballooning, a behavior that distinguishes some *Ummidia* from most other mygalomorphs, has been observed in a few species. A peculiar behavior among most mygalomorphs, it was first noted to occur in *Ummidia* by Baerg (1928) but observed again by Coyle (1985), Coyle et al. (1985), Eberhard (2006), and Fisher et al. (2014). This behavior is significant for biogeographical studies, as *Ummidia* species have been found on multiple islands in the Caribbean, including the volcanic island of St. Vincent (Simon 1891b) and possibly Bermuda (Whitehead unpublished), 1000 km off the eastern coast of the US. The observation of ballooning by *Ummidia* is especially interesting because it facilitates the dispersal of individuals over geographic features that would otherwise serve as barriers to gene flow. Increased gene flow due to long-distance dispersal via ballooning by *Ummidia* species represents a significant departure from what is known for other studied mygalomorph taxa; that is, trapdoor spiders are considered to have extreme population structuring and are considered to be non-vagile (Bond et al. 2001, 2006, Hendrixson and Bond 2005, Arnedo and Ferrández 2007, Starrett and Hedin 2007, Bond and Stockman 2008, Satler et al. 2011).

**Map 1.**
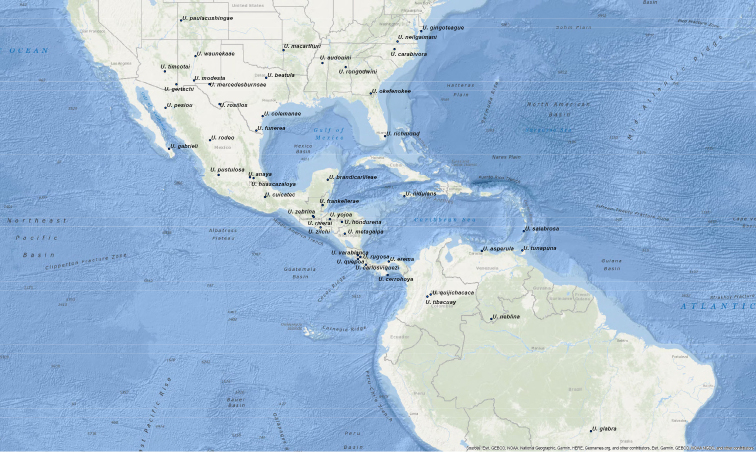
Map of type localities.

**Figure 1.**
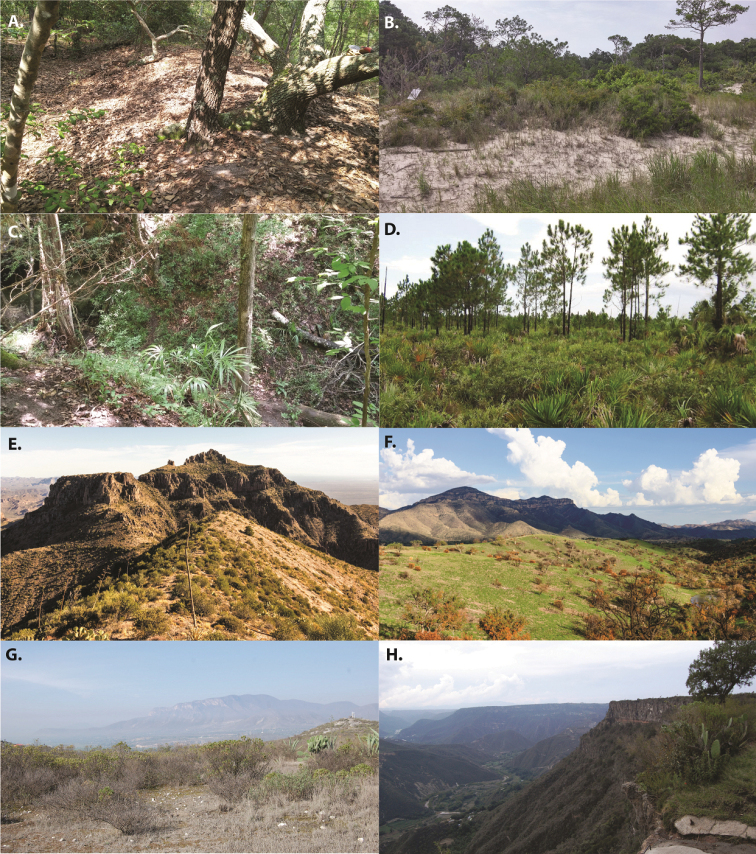
Diversity of *Ummidia* habitats **A** First Landing State Park, Virginia **B** Savage Neck Dunes Natural Area, Virginia **C** Torreya State Park, Florida **D** pine rockland habitat, Miami-Dade County, Florida; photograph credit Frank Ridgely **E** Superstition Mountains, Arizona; photograph credit Timothy Cota **F** near Pajarito Mountains, Arizona; photograph credit Timothy Cota **G** Santiago De Anaya, Hidalgo, Mexico; photograph credit Paula Cushing **H** Peña del Aire Park, Hidalgo, Mexico; photograph credit Paula Cushing.

We present here the first species level revision of *Ummidia* from the Nearctic region wherein we provide updated descriptions for all described species and describe 33 new species. Although species of *Ummidia* are very widespread and occur in a number of habitats (Map 1, Fig. 1), they tend to be very patchy in their distribution. This, paired with the highly cryptic nature of their burrows, make them very difficult to collect, and so by necessity this revision is based almost entirely on historic rather than newly collected material. Much of this material was amassed from a number of collectors and institutions by the late Dr. Willis Gertsch, who spent over 40 years working on a revision of the group. Gertsch never published his work on *Ummidia* prior to his passing in 1999, but his notes and correspondence, stored in the archives of the American Museum of Natural History proved useful and insightful in the completion of this work. A quote, taken from these correspondences, is possibly the best expression of the difficulty of revising this morphologically homogenous group, “this is the most difficult ctenizid genus I know with such feeble, variable, erratic, aberrant characters that I find myself uncertain as to...what is a species.” With the material available to us, we have done our best to accurately delimit species but openly acknowledge that this work represents a first attempt and that much work remains on this enigmatic, geographically widespread, and notoriously difficult genus of trapdoor spiders.

## Materials and methods

### Institutional abbreviations

**AMNH** American Museum of Natural History; New York, New York

**BME** Bohart Museum of Entomology; University of California Davis; Davis, California

**CMC** Cincinnati Museum Center; Cincinnati, Ohio

**CNAN** Colección Nacional de Arácnidos of Instituto de Biología, CNAN; Mexico City, Mexico

**DMNS** Denver Museum of Nature and Science; Denver, Colorado

**MCZ** Museum of Comparative Zoology, Harvard; Boston, Massachusetts

**MEM** Mississippi Entomological Museum, Mississippi State University; Starkville, Mississippi

**MNHN** Muséum National D’Histoire Naturelle, Paris

**MSU** Midwestern State University; Wichita Falls, Texas

**NHMUK** United Kingdom Museum of Natural History; London, England

**NUCMHN** Museo de Historia Natural; Universidad Nacional de Colombia, Bogota, Colombia

**OSU** Oklahoma State University; Stillwater, Oklahoma

**SMF** Senckenberg Museum; Frankfurt, Germany

**TAMU** Texas A&M University; College Station, Texas

**UFMNH** University of Florida Museum of Natural History; Gainesville, Florida

**VMNH** Virginia Museum of Natural History; Martinsville, Virginia

### Quantitative morphological abbreviations

**AER, PER** Anterior/posterior eye row

**AME, PME** Anterior/posterior median eyes

**ALE, PLE** Anterior/posterior lateral eyes

**Bl** Bulb length

**Cap** Caput length

**CL, CW** Carapace length/width

**ENDl, ENDw** Palpal endite length/width

**F1, F1w** Femur I length/width

**F3, F3w** Femur III length/width

**F4, F4w** Femur IV length/width

**LBl, LBw** Labium length/width

**Mt1** Metatarsus I length

**Mt3** Metatarsus III length

**Mt4** Metatarsus IV length

**MtSp, MtSr** Number of spines on the prolateral and retrolateral faces of the metatarsus I

**P1** Patella I length

**P3** Patella III length

**P4** Patella IV length

**PF** Palp femur length

**PP** Palp patella length

**PTi** Palp tibia length (females)

**PTl, PTw** Palpal tibia length/width (males)

**PTr** Palp tarsus length

**Sd3** Saddle length

**STRl**, **STRw** Sternum length/width

**Ti1** Tibia I length

**Ti3** Tibia III length

**Ti4** Tibia IV length

**Tr1** Tarsus I length

**Tr3** Tarsus III length

**Tr4** Tarsus IV length

**TrSp, TrSr** Number of spines on the prolateral and retrolateral faces of the tarsus I

**TSp, TSpv, TSr, TSrv** Number of spines on the prolateral, proventral, retrolateral, and retroventral faces of tibia I

### Measurement, characterization, and illustration of morphological features

Approximately 800 specimens of *Ummidia* from various collections (AMNH, BME, CMC, DMNS, MCZ, MEM, MNHN, MSU, NUCMHN, OSU, SMF, TAMU, UFMNH, NHMUK, CNAN, VMNH) have been examined. Each specimen has been given a unique voucher number deposited in each vial. Specimens were measured using a Leica M165C equipped with Leica Analysis Suite Software, and all measurements are given in millimeters. Measurements of leg article lengths were taken from the mid-proximal point of articulation to the mid-distal point of the article in accordance with Bond 2012 (Figs 11–16). Carapace, leg, and abdomen coloration are described semi-quantitatively using Munsell® Color Charts (Windsor, NY) and are given using the color name and color notation (hue value/chroma).

We evaluated the morphological variation among species using principle components analysis. Base R (R Core Team 2014) scripts and the package “ggplot2” (Wickham 2016) were used. All measurements were log transformed to account for differences in body size, and morphospace was evaluated by plotting PC1 against PC2. PCAs were run on males and females separately further divided by geographic region. Specimens from the South America and Caribbean region were not included due to insufficient sampling.

For representative male specimens, photographs were taken of the retrolateral aspect of the palp and the retro- and prolateral aspects of the mating clasper. The genital region was removed from the abdominal wall and tissues dissolved by incubating in trypsin overnight for representative females. Spermathecae were then examined, measured, and photographed. All specimens were photographed using a Visionary Digital Imaging System (Visionary Digital^TM^, Richmond, VA), which recorded images in multiple focal planes. The images were then assembled into a single focused image using Helicon Focus software (Helicon Soft, Ltd. Ukraine). Line drawings of male mating claspers and palpal tibiae were created using these digital images, which were traced as vector line drawings using Adobe Illustrator (Adobe Systems Inc.). Habitus illustrations were created both for described and undescribed species in order to create digitized records of the type material. Type specimens were photographed in the same manner as other structures and then modified in Adobe Illustrator (Adobe Systems Inc.) to create symmetrical morphological illustrations of type specimens. However, given the poor condition of many of the historical specimens, in some cases fully symmetrical illustrations were not possible.

### Locality data, georeferencing

The locality data from these specimens were databased and georeferenced by hand using Google Earth (WGS84 datum). Due to the vague nature of many older locality labels on museum material, some localities are imprecise. In cases where only a town or county is given as a locality, the specimens were georeferenced to the geographic center of the given locality. Therefore, latitude and longitude were recorded to the fourth decimal place not as an indication of the precision of the recorded locality or the exact location, but as an indication of the precision of the point assigned during georeferencing. A precision ranking for each georeferenced point was given as a superscript in the material examined section. The rankings are as follows: 1 = exact coordinates given, 2 = amended exact coordinates (i.e., exact coordinates given but were emended on validation), 3 = public land survey system (or herein geographic place name), 4 = within 1 km radius, 5 = within 5 km radius, 6 = within 10 km radius, 7 = to county or > 10 km, 8 = to state, and 9 = to project region (Murphy et al. 2004). Species distribution maps were then generated in ArcGIS using either given or assigned points.

### Designation of types and species groups

Given their conserved morphology and sexual dimorphism, matching males to females within a species without the aid of molecular data can be quite challenging. In this revision we have opted for a conservative approach in designating holotypes and paratypes. For new species being described from both sexes, the male is designated as holotype as males tend to possess more diagnostic characters than females. If the female specimen is either from the same locality as the holotype or relatively nearby, it is designated as a paratype. For new species where males and females are available, but not collected in geographic close proximity, the female specimen is described as an exemplar. For described species museum types were examined. These specimens were photographed and measured in the same manner as those examined for new species and new descriptions and diagnoses were written. We have done this in the hope of making these specimen data and digitized images available for future study of these old and valuable specimens. Locality data for type specimens is provided in Table 1 and displayed in Map 1.

**Table 1.** Summary of *Ummidia* New World diversity documenting locality and latitude/longitude for each type locality. * Locality data for the type specimen of *Ummidia audouini* is given only as “North America” so the latitude and longitude shown and displayed in Map 1 are from male exemplar specimen UMM194 from Ecru, Mississippi.

Species	Locality	Latitude/Longitude
*Ummidia audouini*	North America	34.3403 -89.0141*
*Ummidia neilgaimani* sp. nov.	Charlotte County, Virginia	36.7698 -78.6356
*Ummidia gingoteague* sp. nov.	Accomack County, Virginia	37.9083 -75.3568
*Ummidia carabivora*	Chapel Hill, North Carolina	35.9131 -79.0557
*Ummidia rongodwini* sp. nov.	Calhoun County, Alabama	33.8795 -85.7889
*Ummidia okefenokee* sp. nov.	Charlton County, Georgia	30.8056 -82.3371
*Ummidia richmond* sp. nov.	Miami-Dade County, Florida	25.605 -80.3566
*Ummidia macarthuri* sp. nov.	Washington County Arkansas	35.8167 -94.395
*Ummidia beatula*	Dallas County, Texas	32.6472 -96.7913
*Ummidia colemanae* sp. nov.	San Patricio County, Texas	28.0852 -97.2647
*Ummidia funerea*	Edinburg, Texas	26.3005 -98.1623
*Ummidia rosillos* sp. nov.	Brewster County, Texas	29.5385 -103.2323
*Ummidia mercedesburnsae* sp. nov.	Culberson County, Texas	31.8945 -104.6331
*Ummidia paulacushingae* sp. nov.	Mesa County, Colorado	39.0636 -108.5518
*Ummidia modesta*	Dona Ana County, New Mexico	32.338 -106.7661
*Ummidia waunekaae* sp. nov.	Bernalillo County, New Mexico	35.1303 -106.5521
*Ummidia gertschi* sp. nov.	Cochise County, Arizona	31.8842 -109.206
*Ummidia timcotai* sp. nov.	Globe, Gila County, Arizona	33.3940 -110.7864
*Ummidia gabrieli* sp. nov.	La Paz, Baja California Sur, Mexico	24.0345 -110.227
*Ummidia pesiou* sp. nov.	Sonora, Mexico	29.0894 -110.6947
*Ummidia rodeo* sp. nov.	Durango, Mexico	25.1081 -104.5556
*Ummidia pustulosa*	Amula (now Jalisco), Mexico	20.6934 -103.356
*Ummidia huascazaloya* sp. nov.	Hidalgo, Mexico	20.2762 -98.5169
*Ummidia anaya* sp. nov.	Hidalgo, Mexico	20.3884 -99.0275
*Ummidia cuicatec* sp. nov.	Oaxaca, Mexico	17.7984 -96.9519
*Ummidia brandicarlileae* sp. nov.	Yucatan, Mexico	20.0603 -88.2654
*Ummidia zebrina*	Guatemala	15.2812 -90.3420
*Ummidia riverai* sp. nov.	Baja Verapaz, Guatemala	15.1631 -90.2316
*Ummidia frankellerae* sp. nov.	Cayo, Belize	16.733 -88.986
*Ummidia zilchi*	San Salvador, El Salvador	13.6921 -89.219
*Ummidia hondurena* sp. nov.	Honduras	14.5036 -86.3206
*Ummidia yojoa* sp. nov.	Dept. of Cortes, Honduras	14.8715 -87.9821
*Ummidia matagalpa* sp. nov.	Matagalpa, Nicaragua	12.9207 -85.9172
*Ummidia rugosa*	Costa Rica	9.7703 -83.7751
*Ummidia carlosviquezi* sp. nov.	Puntarenas, Costa Rica	8.7125 -83.0051
*Ummidia varablanca* sp. nov.	Heredia, Costa Rica	10.0024 -84.1216
*Ummidia quepoa* sp. nov.	Puntarenas, Costa Rica	9.4828 -84.0948
*Ummidia cerrohoya* sp. nov.	Los Santos, Panama	7.2986 -80.0014
*Ummidia erema*	Panama Canal Zone, Panama	9.1520 -79.8467
*Ummidia quijichacaca* sp. nov.	Cundinamarca, Colombia	4.6049 -74.0565
*Ummidia tibacuy* sp. nov.	Cundinamarca, Colombia	4.3283 -74.5081
*Ummidia asperula*	Maiquetia, Venezuela	10.5778 -66.9973
*Ummidia neblina* sp. nov.	Amazonas, Venezuela	1.2395 -65.6675
*Ummidia tunapuna* sp. nov.	St. George, Trinidad and Tobago	10.6638 -61.3950
*Ummidia salebrosa*	St Vincent	13.2536 -61.187
*Ummidia nidulans*	St. Elizabeth, Jamaica	17.9244 -77.6869
*Ummidia glabra*	Brazil	-14.1241 -51.9029

**Map 2.**
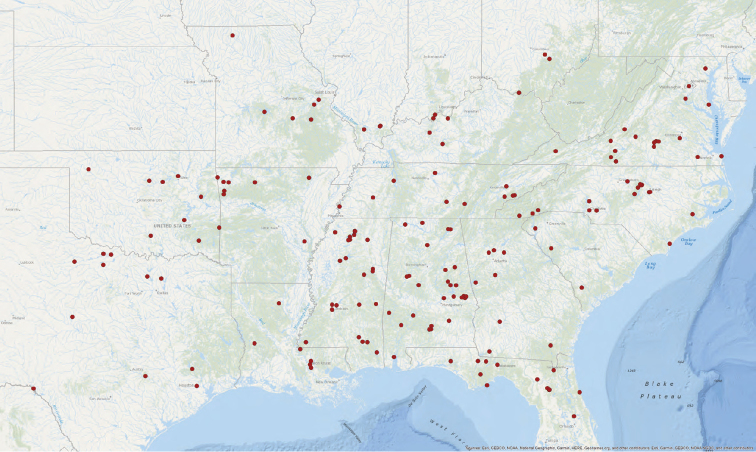
Locality records for *Ummidia audouini*.

**Map 3.**
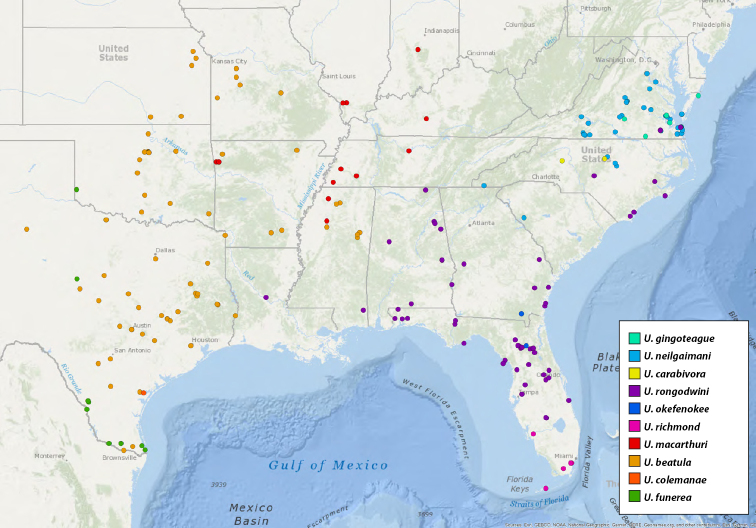
Locality records for eastern United States species.

**Map 4.**
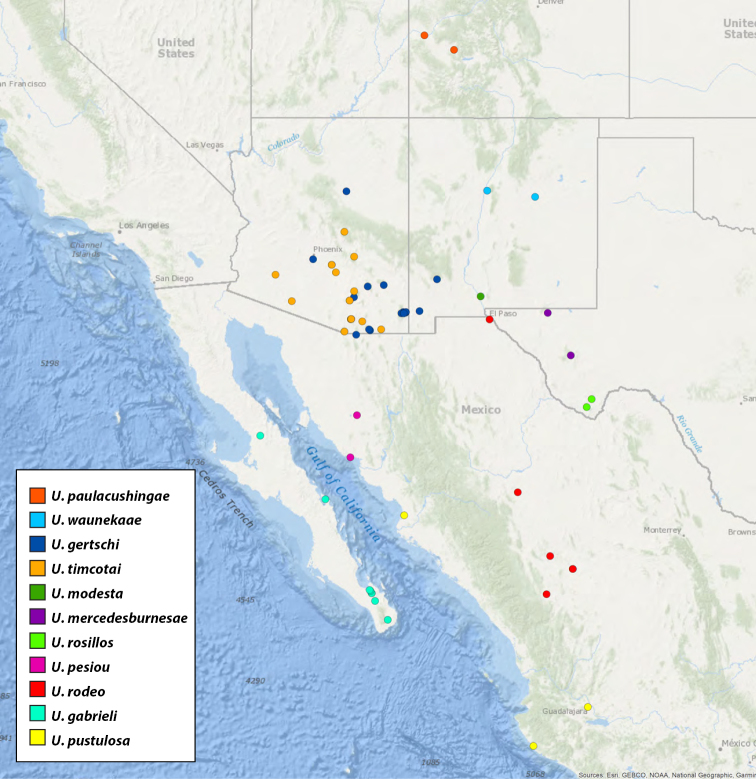
Locality records for western United States and northern Mexico species.

**Map 5.**
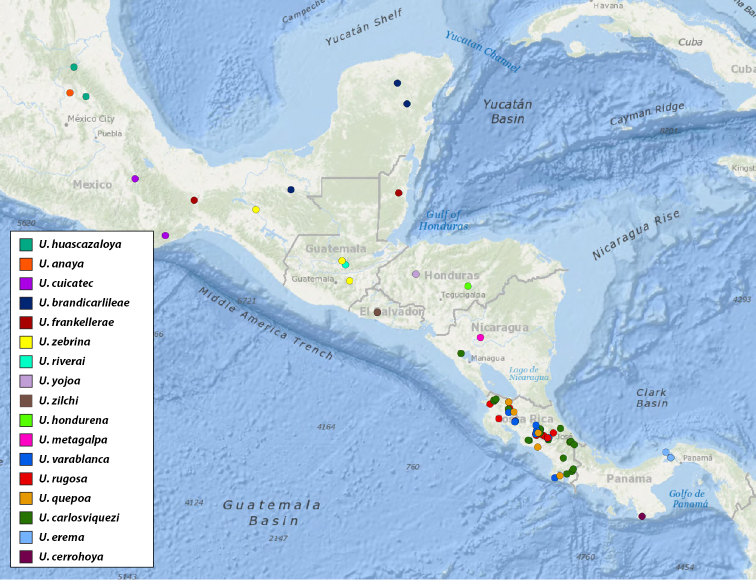
Locality records for southern Mexico and Central American species.

**Map 6.**
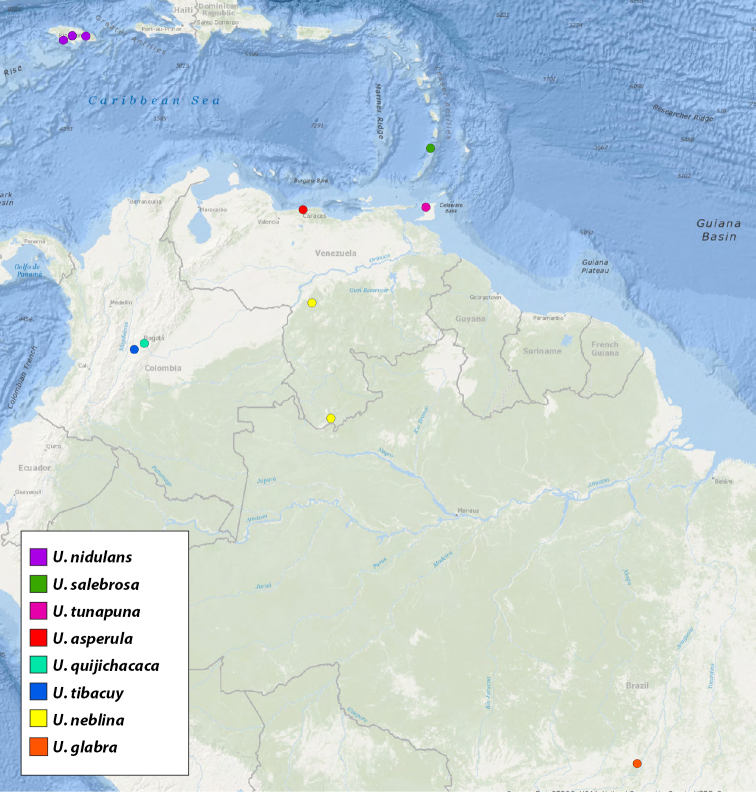
Locality records for South American and Caribbean species.

For pragmatic purposes, we have divided the genus into four species groups: the eastern United States (Maps 2, 3), western United States and northern Mexico (Map 4), southern Mexico and Central America (Map 5), and South America and the Caribbean (Map 6). These groupings are based solely on geography and gaps between species and in sampling. They do not represent phylogenetic relationships, rather are meant to facilitate the clarity of keys and diagnoses.

**Species concept applied**: Species were delineated using a morphological species concept in which species are defined as populations or groups of populations that share a set of phenotypic traits and are distinguishable from other populations or groups.

## Results

**Figure 2.**
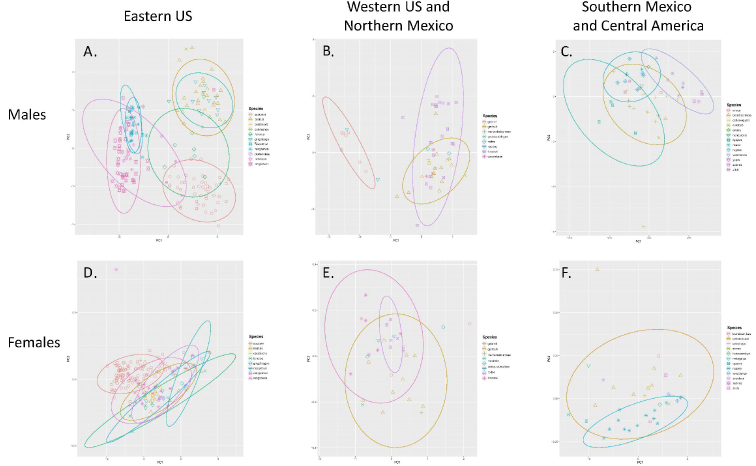
PCA morphospace plotted from quantitative measurements of **A** males of western United States species **B** males of western United States and northern Mexico species **C** males of southern Mexico and Central America species **D** females of eastern United States species **E** females of western United States and northern Mexico species **F** females of southern Mexico and Central America species.

Examination of museum material confirms the undescribed diversity of *Ummidia* noted by Bond and Hendrixson (2005) and others. As expected, many previously used characters (specific eye formation, certain carapace characters, etc.) were highly variable and thus insufficient for species determination, in most cases. Results of PCAs of morphological measures emphasize what has long been known of the morphological homogeneity of mygalomorph spiders (Bond and Opell 2002). Plots of PC1 and PC2 for male species (Fig. 2A–C) divided by region show some degree of separation, though species tend to still overlap in morphospace. For females (Fig. 2D–F) the overlap in morphospace among species is extreme, emphasizing the incredible difficulty in delimiting or identifying species based on female specimens.

**Figure 3.**
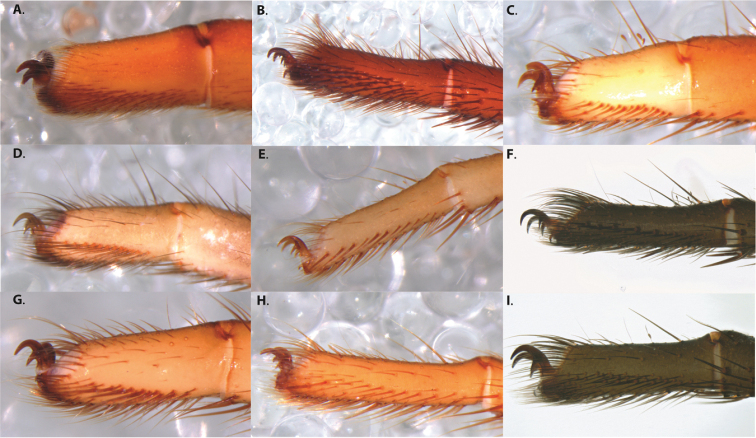
Retrolateral face of tarsus IV for **A***Ummidia audouini* female AR2674 **B***Ummidia audouini* male UMM179 **C***Ummidia hondurena* sp. nov. female SENCK8568 **D***Ummidia gingoteague* sp. nov. female AUMS16243 **E***Ummidia gingoteague* sp. nov. male UMM003 **F***Ummidia huascazaloya* sp. nov. male CNAN-T01384 **G***Ummidia neilgaimani* sp. nov. female UMM240 **H***Ummidia neilgaimani* sp. nov. male UMM016 **I***Ummidia anaya* sp. nov. male CNAN-T01385.

After examining and comparing large numbers of specimens, a list of putatively taxonomically informative characters was created. These included, but are not limited to, pattern and number of prolateral, retrolateral, and retrolaterodistal spines on the tibia of leg I (mating clasper) of males, palpal tibia and bulb size and embolus length, the presence or absence of a brush or longitudinal comb on the retrolateral face of tarsus IV (Fig. 3), and spermathecal bulb and stalk shape.

Detailed locality and associated GIS data, specimen material examined records, and morphological measurements and counts can be downloaded online from the Dryad Data Repository at https://doi.org/10.25338/B8K349.

### Taxonomy

#### Family Halonoproctidae Pocock, 1901

**Subfamily Halonoproctinae Pocock, 1901**

Composition: *Bothriocyrtum* Simon, 1891a, *Cyclocosmia* Ausserer, 1871, *Hebestatis* Simon, 1903.

#### Subfamily Ummidiinae Ortiz, 2007

Composition: *Conothele* Thorell, 1878; *Latouchia* Pocock, 1901; *Ummidia* Thorell, 1875.

##### Genus Ummidia

Taxon classificationAnimaliaAraneaeHalonoproctidae

Thorell, 1875

B1FA24F2-0C60-5DC9-8E36-3E7D58E3F15A

http://zoobank.org/FB0A4D7B-72E2-4F6B-AB6D-0EB71EB85C87

Ummidia Thorell, 1875.
Pachylomerus Ausserer, 1871.

###### Diagnosis.

*Ummidia* can be differentiated from all other halonoproctid genera except *Conothele* by the presence of a dorsal, glabrous, saddle-like depression on tibia III. *Ummidia* can be further distinguished from *Bothriocyrtum*, *Cyclocosmia*, and *Hebestatis* by the presence of clavate trichobothria and the absence of lateral sternal sigilla.

###### General description.

Small to large trapdoor spiders. Cephalothorax longer than wide. Pars cephalica highest behind the eyes and sloping posteriorly. Carapace evenly sclerotized; rough/rugose in males, smooth/shiny in females. Fovea procurved, and deep. At least median eyes on tubercle, eye diameter variable. Anterior eye row procurved, posterior eye row relatively straight to slightly recurved or procurved. Carapace of ethanol preserved specimens yellow, reddish, or dark brown. Living spiders usually black to very dark brown. Abdominal coloration generally dark grey, occasionally with a bright white dorsal patch, pale opalescence, or light patches at apodemes.

Sternum usually approximately as wide as long. Lacking lateral sigilla, posterior sigilla large, central, and indistinct. Palpal endites and labium usually with cuspules, size and number of cuspules variable. Palpal endites longer than wide and without anterior lobe. Chelicerae biserially dentate, cheliceral rastellum generally consisting of large spines on a mound.

With two pairs of spinnerets. Posterior median spinnerets (PMS) short and unsegmented. Posterior lateral spinnerets (PLS) relatively short and three segmented with apical segment shortest and domed. Females with single-lobed spermathecae, which are lightly sclerotized with apical bulb. Male palpal bulb with thin embolus.

Females lacking scopulae, but palps, legs I & II with numerous curved, thorn-like spines laterally. Males with scopulae on legs I & II, tarsi cylindrical to proximally enlarged. Anterior legs with few to many prominent spines on the tibia, metatarsus, and tarsus. Posterior legs stouter with heavy spines. Tibia III with dorsal, glabrous, saddle-like depression. Tarsi with mix of clavate and filiform trichobothria arranged in wide band.

###### Distribution.

*Ummidia* is a wide-ranging genus, found in the southwestern Mediterranean, Central Asia, and in the Americas from as far north as Ohio and Maryland west to Arizona and south to Brazil, including the Greater and Lesser Antilles.

###### Keys to males by region.

As with many members of Mygalomorphae, the highly conserved morphology of *Ummidia* makes identification non-trivial. In addition, females in particular frequently lack reliably identifiable characters of any sort. The authors have provided keys to the males of species divided by region with notes on females where appropriate, but it should be noted that the keys should be used in combination with other information, to include species descriptions, knowledge of the specimen’s locality, and, for males, the season in which it was collected. In addition, for the reasons mentioned above, there is no key to *Ummidia* of South America and the Caribbean due to the lack of male material for all species except *Ummidia asperula*.

### Key to the *Ummidia* of the eastern United States

**Table:** 

1	Brush on retrolateral face of tarsus IV (Fig. 3A,B)	***Ummidia audouini***
–	Without brush on retrolateral face of tarsus IV	**2**
2	Pars cephalica extending 4/5 CL and fovea 1/3 CW	***Ummidia carabivora***
–	Pars cephalica and fovea of usual size	**3**
3	Anterior eye row very strongly procurved in males and females, such that the posterior margin of the ALE is even with the anterior margin of the AME, male embolus sinuous	***Ummidia funerea***
–	Eyes otherwise, male embolus evenly curved	**4**
4	With pale dorsal heart patch on abdomen	***Ummidia rongodwini* sp. nov.**
–	Lacking pale dorsal heart patch	**5**
5	CL 4.5–6, 0–1 PL spines on tibia I, comb on retrolateral face of tarsus IV indistinct (Fig. 3G, H), males dispersing Sept-Nov	**6**
–	With or without PL spines on tibia I, comb on retrolateral face of tarsus IV distinct (Fig. 3D, E), males dispersing May–July	**7**
6	Occurring east of the Appalachians, tubercle under AME only, females with spermathecae straight	***Ummidia neilgaimani* sp. nov.**
–	Occurring west of the Appalachians, all eyes on tubercle, females with spermathecae curved medially and then anteriorly	***Ummidia macarthuri* sp. nov.**
7	Tibia I with ≤ 5 PL spines	**8**
–	Tibia I with > 5 PL spines	**10**
8	CL > 6, eyes relatively small on defined mound removed from carapace margin, tibia I RL spines variable (2–17)	***Ummidia richmond* sp. nov.**
–	CL < 6, eyes otherwise, tibia I with 15–20 RL spines	**9**
9	CL < 3, occurring in south Texas	***Ummidia colemanae* sp. nov.**
–	CL > 3, occurring in north Florida and south Georgia	***Ummidia okefenokee* sp. nov.**
–	10. Tibia I with 2–10 PL spines, females with spermathecae bending medially, bulbs facing anteriorly, occurring in eastern Virginia	***Ummidia gingoteague* sp. nov.**
–	Tibia I with 11–15 PL spines, females with spermathecae straight, occurring west of the Appalachian Mountains	***Ummidia beatula***

### Key to the *Ummidia* of the western United States and northern Mexico

**Table:** 

1	Embolus evenly curved, tibia I with 5–20 PL spines	**2**
–	Embolus longer, sinuous, tibia I PL spines variable	**5**
2	Retrolateral face of tarsus IV with distinct comb	**3**
–	Retrolateral face of tarsus IV with comb loose or indistinct	**4**
3	Tibia I with relatively fewer PL (5–13) and RL (12–24) spines, males emerging in September, occurring in western Colorado	***Ummidia paulacushingae* sp. nov.**
–	Tibia I with relatively more PL (8–21) and RL (15–46) spines, male emerging June to August, occurring in southern Arizona	***Ummidia gertschi* sp. nov.**
4	Tibia I with < 8 PL and < 15 Rl spines, occurring in west Texas	***Ummidia mercedesburnsae* sp. nov.**
–	Tibia I with > 8 PL and > 15 RL spines, occurring in northern New Mexico	***Ummidia waunekaae* sp. nov.**
5	Tibia I with < 5 PL spines	**6**
–	Tibia I with > 5 PL spines	**7**
6	Retrolateral face of tarsus IV with brush, abdomen with pale dorsal heart patch	***Ummidia rosillos* sp. nov.**
–	Retrolateral face of tarsus IV without brush, abdomen lacking pale dorsal heart patch	***Ummidia gabrieli* sp. nov.**
7	Tibia I with <10 PL spines	***Ummidia modesta***
–	Tibia I with >10 PL spines	**8**
8	Retrolateral face of tarsus IV with brush, tibia I with > 20 PL spines	***Ummidia timcotai* sp. nov.**
–	Retrolateral face of tarsus IV without brush, tibia I < 20 spines	***Ummidia rodeo* sp. nov.**

### Key to the *Ummidia* of southern Mexico and Central America

**Table:** 

1	Embolus with single even curve	**2**
–	Embolus sinuous	**5**
2	Without defined comb or brush on retrolateral face of tarsus IV, tibia I with < 10 PL and < 22 RL spines, tarsi light, lacking spines on tarsus I	***Ummidia varablanca* sp. nov.**
–	Not as above	**3**
3	Embolus short and palpal tibia 3.5 ´ long as wide, tibia I with 15–20 PL and 20–35 RL spines, retrolateral face of tarsus IV with defined comb	***Ummidia zilchi***
–	Not as above	**4**
4	CL > 8, comb on retrolateral face of tarsus IV loose or indistinct	***Ummidia quepoa* sp. nov.**
–	CL < 8, retrolateral face of tarsus IV with distinct comb	***Ummidia carlosviquezi* sp. nov.**
5	Abdomen with pale horizontal stripes	***Ummidia zebrina***
–	Abdomen without horizontal stripes	**6**
6	Embolus very long and sinuous ending in strong curve	***Ummidia cuicatec* sp. nov.**
–	Embolus not as above	**7**
7	Tibia I with < 10 PL spines	**8**
–	Tibia I with > 10 PL spines	**10**
8	Tibia I without PL spines and 3 large RL spines, limbs stocky, Mt I thickened distally	***Ummidia yojoa* sp. nov.**
–	Tibia I with RL spines as usual	**9**
9	Tarsus I thickened proximately, with PL spines on Mt and Tr I	***Ummidia brandicarlileae* sp. nov.**
–	Tarsus I not thickened proximately, with PL spines on Mt and Tr I	***Ummidia huascazaloya* sp. nov.**
10	With pale dorsal heart patch on abdomen	**11**
–	Without pale dorsal heart patch on abdomen	**12**
11	Tarsi light in color and retrolateral face of tarsus IV with distinct comb of alternating long and short spinules	***Ummidia riverai* sp. nov.**
–	Tarsi not as above	***Ummidia rugosa***
12	Tibia I with > 16PL and > 25 RL spines, tarsi light	***Ummidia hondurena* sp. nov.**
–	Tibia I with fewer spines on PL and RL faces, tarsi variable	**13**
13	Tibia I > 20 RL spines, tarsi light, and retrolateral face of tarsus IV with distinct comb, occurring in Panama	***Ummidia erema***
–	Tibia I with < 20 RL spines, tarsi not light, retrolateral face of tarsus IV without distinct comb (Fig. 3F), occurring in southern Mexico	***Ummidia anaya* sp. nov.**

### Nomen dubium

*Ummidia oaxacana* (Chamberlin, 1925). Holotype specimen (MCZ1261), from Oaxacana, Mexico, deposited in MCZ is lost and thus no known specimens can be attributed to this name.

### Eastern United States species

#### Ummidia audouini

Taxon classificationAnimaliaAraneaeHalonoproctidae

(Lucas, 1835)

CCEC59C8-3A66-5AEC-99DF-4D76776EB82B

Figs 4 , 5 , Map 2

Pachyloscelis audouini Lucas, 1835: 5; HOLOTYPE: 1 ♀ (26274) from North America, deposited in the MNHN; examined.
Actinopus audouini Lucas, 1837: 387.
Sphodros audouini Walckenaer, 1841: 436.
Mygale solstitialis Hentz, 1842: 56.
Mygale carolinensis Hentz, 1842: 56.
Pachylomerus carolinensis Ausserer, 1871: 147.
Pachylomerus turris Atkinson, 1886: 136.
Pachylomerus audouini Simon, 1891a: 312.
Pachylomerus tuobitus Chamberlin, 1917: 33; HOLOTYPE: 1 ♀ (IZ30181) from Illinois, deposited in the MCZ, examined, **syn. nov.**
Pachylomerides absolutus Gertsch and Mulaik, 1940: 311; HOLOTYPE: 1 ♀ (IZC327744) from Bandera, Texas vii-viii.1937, coll. B Hale, deposited in the AMNH, examined, **syn. nov.**
Ummidia audouini Roddy, 1957: 286.

##### Diagnosis.

*Ummidia audouini* can be differentiated from all other co-occurring species in the eastern United States by the presence of a brush on the retrolateral face of tarsus IV and being relatively larger and hairier than other species. Females can be differentiated from all other eastern United States species on the basis of the spermathecae, which curve strongly laterally and coil distally; bulbs facing laterally. In males, eye mound relatively low, eyes relatively small and eye group usually more than twice as wide as long. Palpal bulb relatively small, embolus sinuous. Males disperse from June to August.

##### Description of female holotype.

*Specimen preparation and condition*. Specimen preserved in 80% EtOH. Spermathecae removed, cleared, in vial with specimen. Color faded. *General coloration*. Carapace, chelicerae, and legs dark yellowish brown 10YR 4/6. Abdomen brown 10YR 5/3, spinnerets light yellowish brown 2.5YR 6/3. *Cephalothorax*. Carapace 11.39 long, 10.88 wide. Pars cephalica 8.27 long. Foveal groove procurved, 1.13 long, 2.8 wide. Eye tubercle low. AER procurved PER straight. Eye group 1.2 long, 2.41 wide, AME 0.44, PME 0.29, ALE 0.54, PLE 0.37. Sternum sparsely setose around outer edges; thicker anteriorly STRl 7.48, STRw 6.8. Chelicerae with anterior row comprising six teeth, posterior margin with nine teeth. Palpal endites with 26 cuspules spread across proximal half of endite and ~100 short spinules distally, ENDw 2.63, ENDl 4.41. Labium with 17 cuspules, LBw 2.33, LBl 1.86. Rastellum with many strong spines on process continuing up cheliceral face for ~ 3× the length of process. *Abdomen*. Evenly setose. *Legs*. F1 6.64; F1w 2.64; P1 4.32; Ti1 4.21; Mt1 2.63; Tr1 1.82; F3 6.07; F3w 3.2; P3 4.09; Ti3 3.52, Sd3 2.82; Mt3 2.6; Tr3 2.55; F4 7.37; F4w 3.47; P4 4.45; Ti4 4.27; Mt4 4.19; Tr4 2.57. Retrolateral face tarsus IV with thick brush. *Pedipalps*. PF 6.07, PP 3.52, PTi 4.1, PTr 3.23. Spermathecae curved laterally with distal coil, bulbs facing laterally.

**Figure 4.**
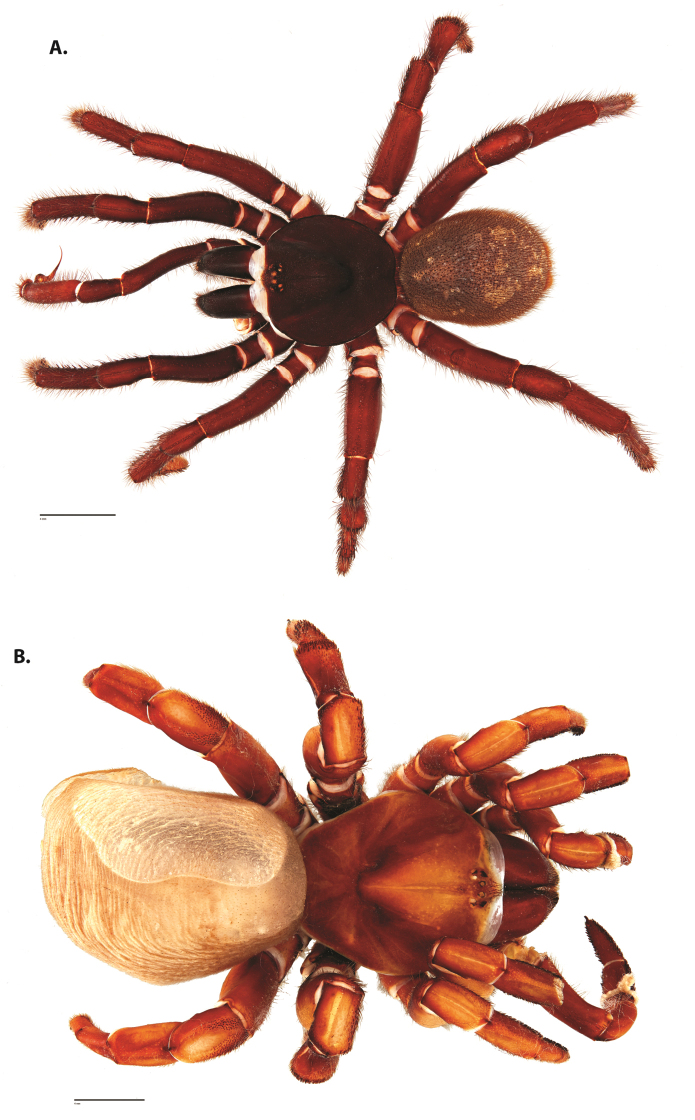
*Ummidia audouini* (Lucas, 1835) from North America. **A** male habitus illustration UMM194 **B** female habitus illustration AR26274. Scale bars: 4.0 mm.

##### Variation, females

**(n = 99).**CL 5.34–13.2, 10.2±0.18; CW 5.04–12.85, 9.58±0.17; Cap 3.5–9.49, 6.96±0.13; ENDl 0.63–1.38, 1.05±0.02; ENDw 1.14–2.96, 2.16±0.04; STRl 3.35–8.68, 6.43±0.12; STRw 3.01–7.73, 5.72±0.1; LBl 0.75–2.23, 1.58±0.03; LBw 1.1–3.22, 2.15±0.04; F1 3.1–7.6, 5.91±0.1; F1w 1.24–3.1, 2.31±0.04; P1 2–4.99, 3.89±0.07; Ti1 1.69–4.84, 3.7±0.07; Mt1 1.12–3.33, 2.42±0.05; Tr1 0.82–2.13, 1.55±0.03; F3 2.73–6.96, 5.37±0.09; F3w 1.62–4, 2.94±0.05; P3 1.91–4.84, 3.66±0.07; Ti3 1.53–4.43, 3.29±0.06; Mt3 0.97–3.84, 2.37±0.05; Tr3 1.06–2.91, 2.07±0.04; F4 3.73–9.27, 7.01±0.12; F4w 1.66–3.89, 3.05±0.05; P4 2–5.15, 4.02±0.07; Ti4 2.02–5.04, 3.91±0.07; Mt4 1.88–5.04, 3.86±0.07; Tr4 1.07–2.88, 2.03±0.03; PF 2.72–7.13, 5.46±0.09; PP 1.68–4.14, 3.24±0.06; PTi 1.68–4.61, 3.54±0.07; PTr 1.3–3.85, 2.93±0.05.

**Figure 5.**
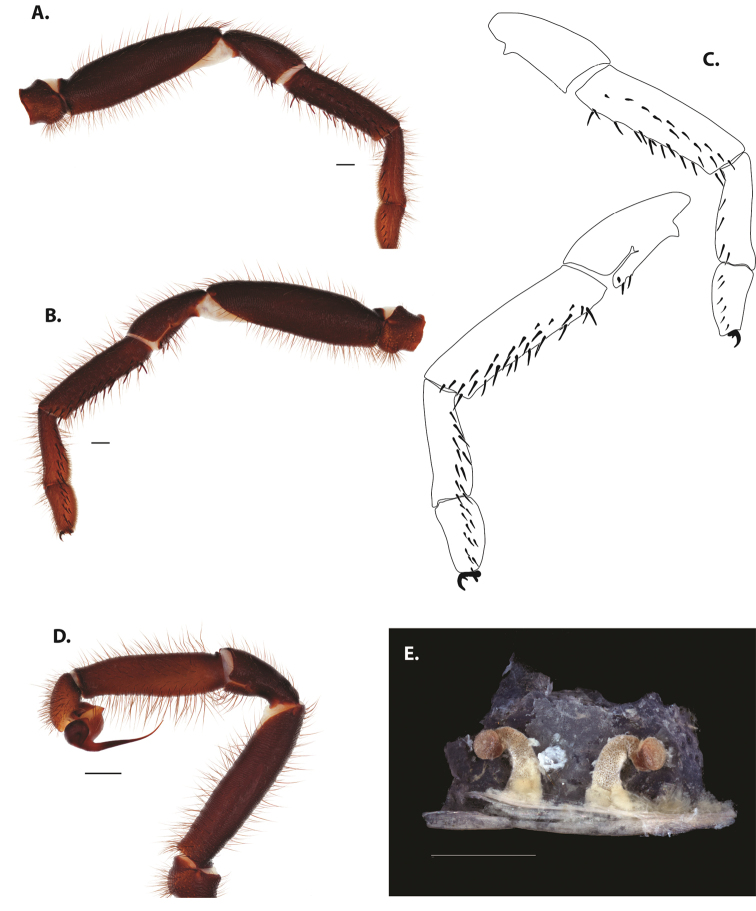
*Ummidia audouini* (Lucas, 1835) from North America **A–D** male exemplar (UMM194) **A** prolateral aspect, leg I **B** retrolateral aspect, leg I **C** line drawings, leg I prolateral and retrolateral aspects **D** retrolateral aspect, pedipalp **E** cleared spermathecae female holotype (AR26274). Scale bars: 1.0 mm.

##### Description of male exemplar.

*Specimen preparation and condition*. Specimen preserved in 80% EtOH. Left palp and legs I removed; in vial with specimen. *General coloration*. Carapace and chelicerae reddish black 2.5YR 2.5/1; legs dark reddish brown 2.5YR 2.5/4. Abdomen black 7.5YR 2.5/1. Cephalothorax. Carapace 7.06 long, 7.23 wide. Pars cephalica 4.78 long. Foveal groove procurved, 0.61 long, 1.46 wide. Tubercle relatively low. AER procurved. PER straight to slightly recurved. Eye group 0.86 long, 1.62 wide, AME 0.37, PME 0.25, ALE 0.4, PLE 0.28. Sternum moderately setose, thicker around outer 1/3, STRl 4.67, STRw 4.12. Chelicerae with anterior tooth row comprising eight teeth, posterior margin with six teeth. Palpal endites with 30 cuspules spread over proximal half of endite face, lacking distal endite cuspules, ENDw 1.59, ENDl 3. Labium with 13 cuspules, LBw 1.36, LBl 1.1. Rastellum with many small spines on process. Abdomen thickly setose. Legs. F1 6.55; F1w 1.87; P1 3.29; Ti1 4.46; Mt1 2.86; Tr1 1.70; F3 5; F3w 2.31; P3 2.66; Ti3 3.02; Sd3 1.6; Mt3 2.7; Tr3 2.34; F4 6.36; F4w 2.16; P4 3.06; Ti4 4.1; Mt4 4.37; Tr4 2.48. Tarsus I with mixed dorsal cluster of clavate/filiform trichobothria. Retrolateral face of tarsus IV with brush of strong spinules. Leg I spination pattern: TSp 12, TSpv 7, TSr 2, TSrv 23, MtSp 4, MtSr 12, TrSp 6, TrSr 12. Pedipalp: PTl 3.96, PTw 1.23, Bl 3.25. Embolus relatively long; sinuous.

##### Variation, males

**(n = 50).**CL 6.09–10.32, 7.77±0.11; CW 3.28–9.53, 7.45±0.16; Cap 3.91–6.61, 5.1±0.08; ENDl 0.54–1.06, 0.82±0.01; ENDw 1.21–1.99, 1.62±0.02; STRl 3.59–6.18, 4.77±0.07; STRw 3.32–5.11, 4.2±0.05; LBl 0.87–1.47, 1.18±0.02; LBw 1.2–1.84, 1.47±0.02; F1 5.31–8.28, 6.56±0.09; F1w 1.43–2.51, 1.95±0.03; P1 1.87–4.46, 3.39±0.06; Ti1 3.45–5.63, 4.36±0.06; Mt1 1.99–3.98, 2.87±0.05; Tr1 1.44–2.21, 1.74±0.02; F3 4.04–6.35, 5.17±0.07; F3w 1.08–2.8, 2.23±0.04; P3 2.21–3.81, 2.86±0.04; Ti3 2.32–3.68, 3.1±0.04; Mt3 2.16–3.74, 2.77±0.04; Tr3 1.82–2.84, 2.29±0.03; F4 3.28–8.96, 6.79±0.11; F4w 1.43–2.89, 2.2±0.03; P4 2.5–4.26, 3.3±0.05; Ti4 3.31–5.65, 4.35±0.06; Mt4 2.55–5.74, 4.33±0.08; Tr4 1.96–2.98, 2.52±0.03; TSp 0–21, 10.66±0.63; TSpv 0–23, 4.56±0.63; TSr 0–5, 1.3±0.18; TSrv 7–32, 17.82±0.78; PTl 3.31–4.83, 4.01±0.04; PTw 1.1–1.64, 1.27±0.02; BL 1.91–4.15, 3.15±0.05.

##### Material examined.

**UNITED STATES: North America** (AR26274, 1♀, M Noisette, MNHN); **Alabama: Bibb Co**: 32.9515 -87.1326^7^, 67 m a.s.l. (UMM0272, 28.vii.1975, 1♀, C Benton, AMNH); Butler: 1.2 mi NW jct AL-55 on US-31 (NW of McKenzie), 31.5664 -86.7402^1^, 93 m a.s.l. (MY0002536, 26.ii.2004, 1♀, Hendrixson, Beamer, BME); McKenzie, 3 mi NW of McKenzie, 31.5739 -86.6779^5^, 91 m a.s.l. (UMM0295, iii.1963, 1♀, BD Valentine, AMNH); S of Georgiana, off hywy 31 (Mobile Rd), just N of Co Rd 8 intersection, 31.5704 -86.7379^1^, 110 m a.s.l. (AUMS005133, 26.v.2013, 1♀, CA Hamilton, JE Bond, WA Shear, BME); 31.6762 -86.6623^7^, 96 m a.s.l. (UMM0462, 10.xi.1957, 1♀, C Benton, AMNH); 32.0896 -88.2219^7^, 55 m a.s.l. (UMM0315, 18.iii.1973, 1juv, C Benton, AMNH); **Calhoun Co**: Jacksonville, 415 High Plateau Drive, 33.4986 -85.7972^1^, 370 m a.s.l. (AUMS016246, 1♂, C Plitt, BME); **Coosa Co**: Alexander City, 4mi from Alexander City, 32.9486 -85.9595^6^, 221 m a.s.l. (UMM0695, 30.v.1974, 1♀, W Baker, BME); Goodwater, 33.0621 -86.0527^5^, 255 m a.s.l. (UMM0732, 15.vi.1980, 1♂, W Baker, BME); **Cullman Co**: Cullman, 34.1791 -86.8113^6^, 255 m a.s.l. (AUMS024769, 9.vii.1991, 1♂, CS Murphree, BME); **Jackson Co**: Mound in Tennessee River near Scottsboro, 34.6522 -85.9622^5^, 197 m a.s.l. (UMM0244, 23.iv.1909, 1♂, AMNH); Scottsboro , 34.6609 -86.0439^5^, 189 m a.s.l. (AUMS24770, 27.viii.2007, 1♀, T Rogers, BME); **Lee Co**: Auburn, 32.6092 -85.4741^5^, 232 m a.s.l. (UMM0731, 7.vii.1996, 1♂, A Teem, BME); Auburn, 32.6001 -85.8425^4^, 146 m a.s.l. (AUMS025725, v.1978, 1♂, F “Sonny”” Eiland III, BME); Auburn, Grove Hill, 31.7062 -87.7778^1^, 151 m a.s.l. (AUMS016098, 30.v.2015, 1♀, NL Garrison, BME); Auburn, Minewa Drive, 32.5476 -85.4248^1^, 182 m a.s.l. (AUMS016247, 13.vii.2015, 1♂, C Guyer, S Herman, BME); (AUMS16343, 29.ix.2015, 1juv, C Guyer, S Herman, BME); Auburn, Wire Road, 32.569 -85.5394^5^, 171 m a.s.l. (UMM0696, vi.1966, 1♀, BME); Darwood Springs, 32.5996 -85.386^7^, 227 m a.s.l. (UMM0736, 7.vii.1999, 1♂, M Patterson, BME); 32.5996 -85.386^7^, 227m (UMM0772, 30.iv.2003, 1♀, B Taylor, BME); 32.5996 -85.386^7^, 227 m a.s.l. (UMM0775, v.2000, 1♀, BME); **Limestone Co**: 34.8486 -86.9965^7^, 234 m a.s.l. (UMM0694, vi.1999, 1♀, Chapman, BME); **Mobile Co**: 30.6968 -88.0444^7^, 3 m a.s.l. (UMM0294, 1941, 1♀, T Johansen , AMNH); 30.6968 -88.0444^7^, 3 m a.s.l. (UMM0287, 1♀, TS Von Allen, AMNH); **Montgomery Co**: Wetumpka, 32.545 -86.2072^4^, 67 m a.s.l. (AUMS018926, 20.v.2003, 1♂, C Gunnels, BME); **Pike Co**: 31.8403 -86.0118^7^, 102 m a.s.l. (UMM0362, 26.vi.1967, 1♂, C Benton, AMNH); **Talladega Co**: 33.4178 -86.1422^7^, 164 m a.s.l. (UMM0697, 8.ix.1999, 1♀, Extension, BME); **Tallapoosa Co**: Eagle Creek, 32.9441 -85.7529^7^, 239 m a.s.l. (AUMS000588, 8.vii.2012, 1♀, W Walker, BME); **Tuscaloosa Co**: Near Holt, 33.234 -87.4844^5^, 115 m a.s.l. (UMM0608, 5.iv.1971, 1♀, M Cameron, AMNH); Tuscaloosa , 33.2094 -87.5694^6^, 60 m a.s.l. (UMM0299, 16.x.1941, 1♀, AMNH); **Wilcox Co**: 32.0075 -87.338^7^, 54 m a.s.l. (UMM0436, 1.vi.1975, 1♀, C Benton, AMNH); Wilson Dam, 34.8004 -87.6256^4^, 154 m a.s.l. (UMM0246, 15.xii.1948, 1♀, AMNH); **Arkansas: Arkansas Co**: DeWhit, 34.2928 -91.3378^6^, 58 m a.s.l. (UMM0320, 28.v.1957, 1♂, AMNH); **Benton Co**: 2 mi N Siloam Springs, Woodland Rd, 36.214 -94.5401^4^, 345 m a.s.l. (UMM0219, 6.vii.1958, 1♂, M Hite, O Hite, MCZ); **Crawford Co**: Ozark National Forest, Fall Creek crossing on AR-220, 35.7135 -94.3082^1^, 249 m a.s.l. (MY0003384, 8.vii.2005, 1juv, B Hendrixson, BME); **Lawrence Co**: Imboden, 36.2025 -91.1745^6^, 95 m a.s.l. (UMM0451, 19.iv.1909, 1♂, 1♀, AMNH); (UMM0335, 22.i.1929, 1♂, BC Marshall, AMNH); **Washington Co**: Cove Creek 15mi S Prairie Grove, 35.8168 -94.2948^6^, 583 m a.s.l. (UMM0607, 1.vii.1960, 1♂, O Hite, M Hite, AMNH); Fayetteville Sequoyah, 36.0657 -94.1372^4^, 417 m a.s.l. (UMM0202, 21.ii.1951, 1juv, Causey, MCZ); Fayetteville, Route 16, 36.0806 -94.3172^1^, 403 m a.s.l. (AUMS016827, 13.vii.2016, 1♂, JR Fisher, BME); **Florida: Alachua Co**: A.M. Laessle place, 29.6573 -82.311^7^, 51 m a.s.l. (UMM0261, 3.i.1947, 1♀, AMNH); Gainesville, 29.6516 -82.3248^6^, 54 m a.s.l. (UMM0177, 13.iv.1982, 1♂, N Backus, UFMNH); Gainesville, Devil’s Mill Hopper, 29.7075 -82.394^4^, 61 m a.s.l. (UMM0255, 1♀, AMNH); Hwy 547, 39.6574 -82.3114^7^, 51 m a.s.l. (UMM0148, 25.iv.1936, 1♀, CUNHC); Rattlesnake Branch, 29.6541 -82.3123^7^, 34 m a.s.l. (UMM0164, 14.vii.1952, 1♂, J McClamroch, AMNH); 29.6573 -82.311^7^, 51 m a.s.l. (UMM0275, 23.x.1947, 1juv, HW, Strawn, AMNH); 39.7799 -82.4793^2^, 1 m a.s.l. (UMM0441, iv.1935, 1♀, HK Wallace, AMNH); **Baker Co**: Glen St. Mary, 30.2761 -82.1614^6^, 39 m a.s.l. (UMM0189, 10.i.1961, 1juv, EW Holder, UFMNH); **Columbia Co**: Itchetucknee Springs State Park, 29.9832 -82.7615^1^, 23 m a.s.l. (AUMS016321, 6.ix.2015, 1♀, RL Godwin, BME); **Flagler Co**: Palm Coast Palm Coast, 29.5724 -81.1964^5^, 3 m a.s.l. (UMM0843, 28.vi.1998, 1♂, D Chicken, TAMU); **Franklin Co**: Dog Island, 29.797 -84.6117^5^, 2 m a.s.l. (UMM0528, 4.iv.1984, 1♂, L Alexander, AMNH); **Gadsden Co**: 30.5578 -84.647^7^, 67 m a.s.l. (UMM0446, 14.iv.1935, 1♀, AMNH); **Leon Co**: Tallahassee , 30.4504 -84.2342^6^, 32 m a.s.l. (UMM0657, 16.vii.2001, 1♂, TD Gowan, UFMNH); **Liberty Co**: Torreya State Park, 30.5697 -84.9476^5^, 77 m a.s.l. (UMM0401, 11.iv.1967, 1♀, FJ Moore, AMNH); (UMM0418, 31.v.1965, 1juv, FJ Moore, AMNH); (UMM0196, 15.vii.1987, 1♂, D Matthews, P Skelley, UFMNH); (UMM0191, 17.vii.1983, 1♂, PM Choate, A Cameron, UFMNH); (UMM0303, 7.ix.1967, 1♀, FJ Moore, AMNH); Torreya State Park, 30.5649 -85.951^4^, 33 m a.s.l. (AUMS016786, iv.2016, 1♂, Alan Jeon, BME); Torreya State Park, Torreya Ravine, 30.5697 -84.9476^5^, 77 m a.s.l. (UMM0184, 10.iii.1963, 1juv, F Hurt, UFMNH); Torreya State Park, Weeping Ridge Trail, 30.5649 -85.951^1^, 33 m a.s.l. (AUMS016217, 1.vii.2015, 1♀, RL Godwin, KF Bourguignon, L Montes de Oca, BME); (AUMS016218, 1♀, BME); (AUMS016216, 30.vi.2015, 1juv, BME); 30.1476 -84.8534^7^, 17 m a.s.l. (UMM0434, 11.iv.1935, 1♀, HKN, AMNH); **Seminole Co**: Long-wood-Markham Rd, 28.8036 -81.4055^5^, 15 m a.s.l. (UMM0173, 1.vii.1963, 1♂, GW Desin, AMNH); **Georgia: Cobb Co**: Marrietta , 33.9524 -84.5477^6^, 344 m a.s.l. (UMM0109, 1.iii.2002, 1♀, TAMU); **Coweta Co**: 35.066 -83.4247^7^, 760 m a.s.l. (AUMS011922, viii.2014, 1♂, B Mount, BME); **Decatur Co**: 30.8721 -84.5213^7^, 44 m a.s.l. (UMM0328, 11.vi.1953, 1♂, TH Hubbell, AMNH); **Fulton Co**: Roswell , 34.0229 -84.3615^6^, 313 m a.s.l. (UMM0220, 1♀, King, MCZ); **Gwinnett Co**: Lawrenceville Lawn, 33.956 -83.9881^6^, 312 m a.s.l. (UMM0116, 25.viii.1967, 1♀, P Wilson, AMNH); **Lee Co**: Bellamy, 31.8126 -84.1432^7^, 80 m a.s.l. (UMM0609, 14.i.1939, 1♀, HKW, AMNH); **Spalding Co**: 33.2637 -84.3121^7^, 271 m a.s.l. (UMM0124, 14.v.1956, 1♀, JE Roberts, AMNH); **Troup Co**: LaGrange , 33.0297 -85.0342^5^, 216 m a.s.l. (UMM0693, 18.viii.2005, 1♀, Z Ogles, BME); **Ware Co**: Okefenokee Swamp Park, nr entrance on Hwy 177, 31.0634 -82.2717^5^, 39 m a.s.l. (UMM0440, 3.vii.1984, 1♂, GL Miller, AMNH); **Illinois: Saline Co**: Harrisburg , 37.7382 -88.5404^6^, 121 m a.s.l. (UMM0433, 23.v.1959, 1♀, RE Sands, AMNH); Murray’s Bluff, 37.7139 -88.5557^7^, 129 m a.s.l. (UMM0382, 9.vii.1969, 1♂, D Evans, AMNH); **Williamson Co**: Little Grassy Lake, 37.6287 -89.1431^5^, 153 m a.s.l. (UMM0431, 27.vi.1970, 1♂, J Hunt, AMNH); (UMM0402, 30.vi.1970, 1♂, AMNH); (UMM0381, 5–23.vii.1971, 1♂, AMNH); (UMM0425, 6.vii.1970, 1♂, AMNH); Touch of Nature, 37.6259 -89.1465^4^, 162 m a.s.l. (UMM0405, 16.vi.1975, 1♂, T Meldau, AMNH); (UMM0453, 24.vi.1975, 1♂, J Culpen, AMNH); **Indiana: Perry Co**: Hoosier National Forest, Morgan Ridge, 38.0556 -86.5332 1, 165 m a.s.l. (UMM0692, 12.vii.2017, 1♂, A Bess, BME); Rome, Tobin Township, 37.9525 -86.589^5^, 223 m a.s.l. (UMM0769, 27.viii.2016, 1♀, H Machen, BME); **Kentucky: Edmonson Co**: Indian Hill , 37.2027 -86.25^4^, 226 m a.s.l. (UMM0221, 22.viii.1881, 1♀, MCZ); **Meade Co**: Brandenburg, Rock Ridge Road, 37.9495 -86.0577^1^, 197 m a.s.l. (AUMS024101, 7.vii.2017, 1♂, D Steenbergen, BME); **Ohio Co**: Olaton, New Baymus Rd, 37.5325 -86.7296^1^, 150 m a.s.l. (MY0002041, 16.vi.2003, 1♂, Canary, Canary, BME); **Louisiana: East Baton Rouge Co**: Highland Rd, Oak Hills Area, 30.3553 -91.0946^4^, 6 m a.s.l. (UMM0290, iv.1975, 1♀, AMNH); Baton Rouge, 30.4579 -91.1405^6^, 15 m a.s.l. (UMM0260, ix.1956, 1♂, AMNH); 30.5696 -91.0981^7^, 22 m a.s.l. (UMM0567, vi.1955, 1♂, LR Roddy, AMNH); **Ouachita Co**: 20mi SW of Monroe, 32.3894 -92.2839^5^, 54m (UMM0312, 24.viii.1940, 1♀, S Mulaik, D Mulaik, AMNH); **Vernon Co**: 31.1306 -93.1781^7^, 97 m a.s.l. (UMM0217, 14.vi.1956, 1♂, T Mitcham, MCZ); **West Feliciano Co**: Tunica Hills Nature Preserve, 2.25 Airline miles W Weyanoke, 30.9467 -91.4985^4^, 73 m a.s.l. (UMM0326, 1.xii.1973, 1♀, Rossman, Erwin, AMNH); (UMM0327, 19.iv.1978, 1♀, Rossman, Vaeth, AMNH); **Maryland: Calvert Co**: Olivet, ca. 2 mi NE of Solomons, 38.3453 -76.4402^5^, 9 m a.s.l. (UMM0250, 16.vi.1966, 1♀, F Younger, AMNH); Conduit Rd, near Cabin John P.O., 39.3875 -76.5623^4^, 121 m a.s.l. (UMM0232, 10.vii.1911, 1♂, EA Schwarz, AMNH); **Mississippi: Choctaw Co**: Tom Bigbee National Forest, nr Hwy-15, Choctaw WMA, campground hiking trail, 33.2733 -89.1449^1^, 131 m a.s.l. (MY0003600, 2.viii.2007, 1juv, BE Hendrixson, AK Stockman, CL Spruill, AL Bailey, BME); (MY0003616, 1juv, BME); **Forrest Co**: Camp Shelby, near Hattiesburg, 31.1805 -89.1984^6^, 73 m a.s.l. (UMM0329, 22.vii.1943, 1♀, Michener, AMNH); Hattiesburg, Mississippi Southern College Campus, 31.3282 -89.3357^4^, 67 m a.s.l. (UMM0245, vii.1957, 1♂, B Valentine, B Valentine, AMNH); **George Co**: 30.8434 -88.6723^7^, 42 m a.s.l. (UMM0319, 4.x.1975, 1♀, C Benton, AMNH); **Grenada Co**: Grenada, 33.7788 -89.8125^5^, 85 m a.s.l. (UMM0730, 24.iv.1991, 1♀, S Winters, MEM); 33.6963 -90.03^1^, 77 m a.s.l. (UMM0720, 19–26.vi.1991, 1♂, T Schiefer, MEM); (UMM0709, 26.vi-2.vii.1991, 1♂, PR Miller, GT Baker, MEM); (UMM0719, 5–11.vi.1991, 1♂, MEM); **Harrison Co**: 40.3222 -93.9889^7^, 303 m a.s.l. (UMM0223, x.1934, 1♀, MCZ); **Hinds Co**: Clinton, 32.3412 -90.3218^6^, 109m (UMM0332, Spring 1926, 1♀, AMNH); Jackson, Mississippi Museum of Nature and Science, 32.3249 -90.1569^4^, 123 m a.s.l. (UMM0773, 18.iii.2005, 1♀, S Payton, BME); Springridge Road, 32.1931 -90.3256^6^, 110 m a.s.l. (UMM0734, 3.vi.2012, 1♂, HJ Ellis, BME); (UMM0737, 24.vi.2005, 1♂, BME); **Lafayette Co**: 1mi SW of Abbeville, 34.4891 -89.5103^4^, 136 m a.s.l. (UMM0723, 21.vi.2002, 1♂, GE Stratton, MEM); (UMM0727, 9.vii.2013, 1♀, MEM); 8 mi SE of Oxford, 34.6 -89.4833^1^, 105 m a.s.l. (UMM0721, 15–22.vii.1992, 1♂, K Kallies, MEM); (UMM0708, 1–8.vii.1992, 1♂, MEM); (UMM0712, 1♂, MEM); (UMM0707, 8–15.vii.1992, 1♂, MEM); (UMM0710, 1♂, MEM); (UMM0711, 1♂, MEM); Burgess, 34.3466 -89.6651^5^, 100 m a.s.l. (UMM0729, 7.vii.1999, 1♀, S Rikard, MEM); Clear Creek Rec. Area, Sardis Lake, Hwy. 314, 34.4333 -89.6666^4^, 131 m a.s.l. (UMM0718, 20.vi.2003, 1♂, K Chatelain, H Bufkin, MEM); **Lauderdale Co**: Meridian, 32.3641 -88.703^6^, 108 m a.s.l. (UMM0578, 17.vi.1975, 1♂, G Bredimeier, AMNH); **Marshall Co**: 3 mi S of Waterford, 34.5974 -89.4723^5^, 107 m a.s.l. (UMM0716, 30.v.1994, 1♂, S Watson, MEM); **Oktibbeha Co**: Starkville, 33.4503 -88.8183^6^, 101 m a.s.l. (UMM0284, 4.vii.1981, 1♂, WE Cross, AMNH); 33.3797 -88.8288^1^, 94 m a.s.l. (UMM0713, 5.vii.1991, 1♂, RL Brown, BB Brown, MEM); **Panola Co**: 11 mi E of Batesville, 34.3337 -89.7208^5^, 145 m a.s.l. (UMM0714, 27.vi.1990, 1♀, L Rutherford, MEM); **Perry Co**: 31.1646 -89.019^7^, 70 m a.s.l. (UMM0420, 15.ii.1976, 1♀, C Benton, AMNH); 31.1646 -89.019^7^, 70 m a.s.l. (UMM0248, 5.xii.1975, 1♀, C Benton, AMNH); **Pontotoc Co**: 1mi SE Ecru, 34.3403 -89.0141^5^, 112 m a.s.l. (UMM0194, 18.vi.1980, 1♂, WH Cross, UFMNH); (UMM0176, 7.vii.1980, 1♂, UFMNH); (UMM0179, 1♂, UFMNH); **Scott Co**: Lake, Scott County line, 32.3424 -89.3224^5^, 144 m a.s.l. (UMM0774, 31.vii.2006, 1♀, BME); **Tate Co**: Sarah, 34.568 -90.2102^5^, 69 m a.s.l. (UMM0728, v.1993, 1♀, MJ Craft, MEM); **Wilkinson Co**: 31.1642 -91.2893^7^, 76 m a.s.l. (UMM0265, 13.vii.1975, 1♂, C Benton, AMNH); (UMM0310, 1♀, AMNH); **Missouri: Franklin Co**: 2 mi S Gray Summit, 38.4897 -90.8168^5^, 195 m a.s.l. (UMM0334, 23.vii.1936, 1♂, L Hubricht, AMNH); St. Clair BSH Camp, 38.3496 -90.9828^6^, 231 m a.s.l. (UMM0123, 1.vii.1971, 1♂, D Gates, AMNH); **Phelps Co**: Rolla , 37.9513 -91.7713^6^, 341 m a.s.l. (UMM0304, 30.v.1958, 1♀, B Russell, AMNH); **Washington Co**: Intersection of Hwy 8 and Huzzah Rd, 37.9178 -91.0935^6^, 246 m a.s.l. (UMM0130, 25.vi.1970, 1♂, AMNH); Lake of the Ozarks, 38.1376 -92.8115^6^, 202 m a.s.l. (UMM0345, 17.vii.1960, 1♂, FE Wood, AMNH); **North Carolina**: Burlington, 36.096 -79.4375^6^, 198 m a.s.l. (UMM0447, Summer 1935, 1♀, KW, AMNH); Chapel Hill, 35.9131 -79.0558^6^, 145 m a.s.l. (UMM0152, 1986, 1♀, DE Woodley, CUNHC); **Chatham Co**: Pittsboro, 35.7201 -79.1772^6^, 120 m a.s.l. (UMM0150, 10.iv.1886, 1♀, C Merrit, CUNHC); (UMM0151, 1♀, C Merrit, CUNHC); **Craven Co**: New Bern, 35.1087 -77.038^3^, 3 m a.s.l. (AUMS016828, 1♂, BME); **Durham Co**: Durham , 35.9933 -78.8977^6^, 124 m a.s.l. (UMM0432, iv, 1♀, AMNH); Durham, Duke Forest, 35.9813 -78.9429^4^, 104 m a.s.l. (UMM0214, 16.ii.1933, 1juv, Chickering, MCZ); (UMM0251, 1936–1937, 1♀, AMNH); Durham, Duke Forest, 36.0106 -78.9664^1^, 110 m a.s.l. (MY0000653, 26.x.2002, 1♀, JE Bond, BE Hendrixson, BME); **Mecklenburg Co**: Charlotte, 35.2265 -80.8428^6^, 213 m a.s.l. (UMM0426, 25.i.1964, 1♀, R Peithman, AMNH); Davidson, 35.4989 -80.8482^6^, 250 m a.s.l. (UMM0435, 14.vi.1951, 1♀, EE Brown, AMNH); Davidson College, 35.5014 -80.8452^4^, 253 m a.s.l. (UMM0293, 19.v.1948, 1♀, RD Barnes, AMNH); Midland, Old Camden, 35.218 -80.5767^1^, 184 m a.s.l. (MY0002042, 21.vi.2003, 1♂, J Johnson, BME); **New Hanover Co**: Wilmington, 34.2233 -77.8834^6^, 11 m a.s.l. (UMM0427, 25.vi.1954, 1♂, AMNH); **Transylvania Co**: Brevard, 35.2334 -82.7341^6^, 675 m a.s.l. (UMM0218, 7.vii.1942, 1♂, Westfall, MCZ); Toxaway River Gorge, 35.1314 -82.9358^5^, 877 m a.s.l. (UMM0477, vii.1961, 1♀, JE Carico, AMNH); **Wake Co**: Raleigh, 35.7713 -78.6383^6^, 96 m a.s.l. (UMM0208, 31.v.1943, 1♀, JA Hoarris, MCZ); Raleigh, 35.7713 -78.6383^6^, 96 m a.s.l. (UMM0142, 1♀, F Sherman, CUNHC); Raleigh, S. King Charles Rd., 35.7772 -78.6056^4^, 92 m a.s.l. (UMM0114, 16.x.1980, 1♀, MG Whonley, NCSM); **Ohio: Adams Co**: Green Township, Rieveschl chalet, 38.6898 -83.4391^1^, 221 m a.s.l. (UMM0768, 19.vi.2007, 1♂, M Zloba, CSNH); (UMM0767, 23.vi.2007, 1♂, CSNH); **Oklahoma: Creek Co**: E. of Oilton, 36.081 -96.5653^5^, 234 m a.s.l. (UMM0813, 30.iv.1986, 1♀, N Rachuk, OSU); **Le Flore Co**: Base of Rich Mtn on N side, 34.7077 -94.4834^6^, 357 m a.s.l. (UMM0413, 27.vi.1972, 1♀, F Coyle, AMNH); **Murray Co**: Platt National Park (Chickasaw National Recreation Area), 34.4611 -97.0065^4^, 295 m a.s.l. (UMM0820, 17.iv.1977, 1♀, J Elsbree, OSU); (UMM0439, 21.viii.1972, 1♀, AMNH); **Muskogee Co**: Greenleaf Lake, 35.6367 -95.1521^5^, 158 m a.s.l. (UMM0819, 30.vi.1967, 1♂, B Clymer, OSU); **Payne Co**: Stillwater, 36.1146 -97.0588^6^, 282m (UMM0825, 27.iv.2004, 1♀, OSU); **Pittsburg Co**: McAlester, 34.9324 -95.7699^5^, 223 m a.s.l. (UMM0829, 5.vi.2006, 1♂, T Evicks, OSU); **Woodward Co**: Boiling Springs State Park, 36.4546 -99.2978^4^, 577 m a.s.l. (UMM0397, 1♂, RJ Ellis, AMNH); 5mi N Tulsa, 36.2524 -95.998^5^, 250 m a.s.l. (UMM0414, 26.vi.1937, 1♂, WF Blair, FA Blair, AMNH); Kiamichi River, 34.3192 -95.2304^7^, 210 m a.s.l. (UMM0213, 24.vi, 1♂, MCZ); **South Carolina: Greenwood Co**: nr Fell Hunt Campground, 34.08 -82.25^1^, 178 m a.s.l. (MY02784, 1.v.2004, 1juv, BE Hendrixson, BME); **Hampton Co**: 32.8781 -81.1273^6^, 33 m a.s.l. (UMM0412, 25.ix.1975, 1♀, C Benton, AMNH); Clemson, 34.6835 -82.837^6^, 215 m a.s.l. (UMM0461, 1978, 1♀, H Douglas, AMNH); **Tennessee: Benton Co**: 36.1153 -88.0521^7^, 168 m a.s.l. (UMM0530, 15.vii.1952, 1♂, TJ Walker, AMNH); **Blount Co**: Montvale Springs , 35.6397 -83.9618^5^, 368 m a.s.l. (UMM0585, 18.xi.1929, 1juv, AMNH); **Knox Co**: Knoxville, Canary Ave, 35.9453 -83.9036^4^, 271 m a.s.l. (UMM0580, 31.iii.1974, 1♀, TB Shepherd, AMNH); **Madison Co**: Jackson, 35.6143 -88.814^6^, 125 m a.s.l. (UMM0330, 15.xi.1954, 1♀, K Hill, AMNH); Jackson, 35.6143 -88.814^6^, 125 m a.s.l. (UMM0301, vi.1953, 1♀, A Young, AMNH); **Overton Co**: Campsite at Standing Stone State Park, 35.4825 -86.1054^5^, 286 m a.s.l. (UMM0593, 5.vii.1964, 1♂, AMNH); **Sequatchie Co**: 206 mi NW TN-28 on Fredonia Rd, 35.4229 -85.4358^7^, 475 m a.s.l. (MY0002716, 6.iv.2004, 1juv, BE Hendrixson, BME); **Sevier Co**: Great Smokey Mtn National Park, Laurel Creek, 35.6782 -83.5941^4^, 864 m a.s.l. (UMM0592, 5.v.1963, 1♂, FJ Moore, AMNH); Great Smokey Mtn National Park, near Sinks Bridge on Little River, 35.6692 -83.662^4^, 547 m a.s.l. (UMM0584, 15.vi.1939, 1♂, J Dodgen, AMNH); **Shelby Co**: Meeman-Shelby Forest State Park, 35.3433 -90.0485^1^, 41 m a.s.l. (UMM0698, 10.iii.2018, 1♀, J Starrett, BME); (UMM0699, 1♀, BME); **Sumner Co**: Gallatin, 36.3469 -86.532^1^, 151 m a.s.l. (AUMS016662, 27.ii.2016, 1♀, RL Godwin, KF Bourguignon, BME); (AUMS016663, 1♀, BME); **Texas**: 31.9572 -99.8965^8^, 567 m a.s.l. (UMM0428, 1♀, AMNH); **Archer Co**: 33.5734 -98.7546^7^, 322 m a.s.l. (UMM0794, 23.vii.1987, 1♂, S DeHaven, MSU); **Bastrop Co**: ½ mi NW of Buescher SP, nr Park Rd #1, 30.0898 -97.1958^5^, 149 m a.s.l. (UMM0776, 31.viii.1974, 1♀, WJ Icenogle, BME); **Collin Co**: McKinney Heard Natural Science Museum and Wildlife Sanctuary, 33.1586 -96.6118^5^, 161 m a.s.l. (UMM0107, vi.2004, 1♂, M Dudas, TAMU); **Denton Co**: Near Creek Woods, 33.2143 -97.1332^6^, 194 m a.s.l. (UMM0333, 18.iii.1942, 1♀, AMNH); **Harris Co**: 29.7758 -95.3102^6^, 11 m a.s.l. (UMM0316, ix.1941, 1♂, R Scott, AMNH); **Knox Co**: 33.6734 -99.8125^7^, 437 m a.s.l. (UMM0792, 26.vi.1984, 1♀, D Patrick, MSU); **Montgomery Co**: 30.3199 -95.4745^7^, 59 m a.s.l. (UMM0790, 15.iii.1985, 1♀, D Russell, MSU); **Val Verde Co**: Seminole Canyon State Park, 29.6924 -101.3198^1^, 420 m a.s.l. (UMM0787, 1.vi.2015, 1♀, BE Hendrixson et al, MSU); **Wichita Co**: Wichita Falls, Sunnyside, 33.8874 -98.4639^4^, 301 m a.s.l. (UMM0805, 30.vii.2018, 1♂, C Loveless, MSU); 33.9142 -98.7506^7^, 308 m a.s.l. (UMM0798, 15.vii.1994, 1♂, K Lindsey, MSU); (UMM0788, 17.vii.1972, 1♂, P Lifsov, MSU); (UMM0793, 17.vii.1989, 1♂, K Ward, MSU); (UMM0809, 22.vii.1996, 1♂, NV Horner, Post Office Cindy, MSU); (UMM0804, 27.vii.1995, 1♂, C Loveless, MSU); (UMM0807, 31.7.2012, 1♂, C Lovelace, MSU); (UMM0806, x.1975, 1♀, T Zupkas, MSU); **Virginia**: Falls Church, 38.8823 -77.171^6^, 101 m a.s.l. (UMM0209, 1♀, N Banks, MCZ); Salem, 37.2933 -80.0546^6^, 325 m a.s.l. (UMM0296, 1927, 1♀, AMNH); **Albemarle Co**: Earlysville, 37.124 -78.465^1^, 172 m a.s.l. (UMM0766, 1.vii.2018, 1♂, BME); **Campbell Co**: Lynchburg, 37.4135 -79.1423^6^, 187 m a.s.l. (UMM0306, 7.vii.1969, 1♀, JE Carico, AMNH); (UMM0309, 8.vii.1968, 1♀, AMNH); (UMM0302, Summer 1966, 1♀, AMNH); (UMM0207, 9.vii.1966, 1♂, J Howell, MCZ); **Chesterfield Co**: Chesterfield, 37.3772 -77.5048^6^, 61 m a.s.l. (UMM0443, 11.v.1967, 1♀, RE Mann, AMNH); **Franklin Co**: Rocky Mount, 36.9973 -79.8921^6^, 342 m a.s.l. (UMM0247, 25.vi.1964, 1♀, AMNH); Salthouse Campground, Philpott Lake, 36.8124 -80.046^5^, 297 m a.s.l. (UMM0027, 2.vi.2004, 1♂, P Katsifos, VMNH); **Henry Co**: Martinsville Inside VMNH Building, 36.6872 -79.8645^4^, 312 m a.s.l. (UMM0039, 21.vii.1996, 1♂, JM Anderson, VMNH); Martinsville Outside VMNH building, 36.6872 -79.8645^4^, 312 m a.s.l. (UMM0051, 24.vii.1997, 1♂, L Hoyt, VMNH); **Isle of Wight Co**: Zuni pine barrens, 3.5 SW of Zuni, 36.8174 -76.8532^5^, 11 m a.s.l. (UMM0099, 27.vii.1985, 2♂, CA Pague, VMNH); **Prince Edward Co**: Farmville, Price Dr, 37.2836 -78.3793^1^, 132m (UMM0083, 15.iv.1993, 1♀, JK Shear, BME); (UMM0770, 14.iv.2010, 1♀, WA Shear, VMNH); (UMM0742, 20.vii.2008, 1♂, BME); (UMM0098, 26.vi.1988, 1♂, VMNH); (UMM0041, 27.v.1978, 1♀, VMNH); (AUMS016826, 9.vii.2016, 1♂, BME); Hampden-Sydney, 37.2397 -78.46^4^, 164 m a.s.l. (UMM0771, 20.vi.2011, 1♀, BME); Hampden-Sydney, 37.2423 -78.4596^5^, 163 m a.s.l. (UMM0059, vii.1976, 1♀, M Farrell, T Martin, VMNH); Rice, Lockett Rd, 37.2751 -78.2913^5^, 138 m a.s.l. (UMM0026, vi.2005, 1♂, Residents, VMNH); **Prince William Co**: Quantico, 38.5206 -77.298^1^, 21 m a.s.l. (UMM0331, 7.v.1918, 1♀, VD Bain, AMNH); **Rockbridge Co**: Natural Bridge, 37.6299 -79.5433^6^, 331 m a.s.l. (UMM0291, v.1884, 1♀, FW Putnam, AMNH); **Russell Co**: 36.9896 -82.0837^7^, 559 m a.s.l. (UMM0072, vii.1998, 1♀, VMNH); **Virginia Beach Co**: Virginia Beach City, Arctic Circle, 36.8414 -75.9754^4^, 0 m a.s.l. (UMM0036, vi.1993, 1♀, M Standing, VMNH).

#### Ummidia neilgaimani sp. nov.

Taxon classificationAnimaliaAraneaeHalonoproctidae

CF42CB5F-26FD-5FED-8717-8DF8CD814467

http://zoobank.org/0C72F9A2-DBF6-4062-8A32-503369919BDD

Figs 6 , 7 , Map 3

##### Type material.

HOLOTYPE: 1 ♂ (UMM016), from Virginia Western Community College, Roanoke County, Virginia, United States, 37.2462 -79.9744^4^, 333 m a.s.l., 26.x.1995, coll. MW Donahue, VMNH. PARATYPE: 1 ♀ (UMM240) from Red Oak, Charlotte County, Virginia, United States, 36.7698 -78.6356^6^, 140 m a.s.l., 2.v.1968, coll. WW Baker, AMNH.

##### Etymology.

The specific epithet is a patronym in honor of Neil Gaiman, author of a number of fantasy and horror books with spider-based characters. In one book, *American Gods*, the main character is tied to the World Tree located “one hour south of Blacksburg,” not far from the type locality of this species.

##### Diagnosis.

*Ummidia neilgaimani* can be differentiated from *U. audouini* by the presence of a comb on the retrolateral face of tarsus IV, which is less defined than the comb in *U. rongodwini* and *U. gingoteague*. Males can further be distinguished from *U. gingoteague* and *U. carabivora* by lacking spines on the prolateral aspect of tibia I and from *U. rongodwini* by lacking a pale dorsal heart patch. Males disperse from September to November.

##### Description of male holotype.

*Specimen preparation and condition*. Specimen preserved in 80% EtOH. Left palp and leg I removed, in vial with specimen. *General coloration*. Carapace and chelicerae very dusky red 10R 2.5/2, legs proximally dark reddish brown 5YR 3/4, Mt and Tr strong brown 7.5YR 5/8. Abdomen dark grayish brown 10YR 4/2. *Cephalothorax*. Carapace 5.71 long, 5.63 wide. Pars cephalica 3.94 long. Foveal groove procurved, 0.5 long, 1.06 wide. ME on moderate tubercle. AER procurved. PER slightly recurved. Eye group 0.85 long, 1.46 wide, AME 0.36, PME 0.25, ALE 0.49, PLE 0.33. Sternum with posterior fringe, sparsely setose anteriorly, STRl 3.18, STRw 3.22. Chelicerae with anterior tooth row comprising seven teeth, posterior margin with five teeth. Palpal endites with 20 cuspules spread over proximal half of endite face, lacking distal endite cuspules, ENDw 1.36, ENDl 2.34. Labium with five cuspules, LBw 1.15, LBl 0.86. Rastellum with many spines on process. Abdomen setose with pale speckles concentrated at apodemes. *Legs*. F1 5.27; F1w 1.28; P1 2.59; Ti1 3.4; Mt1 2.46; Tr1 1.3; F3 4.05; F3w 1.49; P3 2.02; Ti3 2.34; Sd3 1.33; Mt3 2.35; Tr3 1.85; F4 5.37; F4w 1.46; P4 2.28; Ti4 3.44; Mt4 3.62; Tr4 2.18. Retrolateral face of tarsus IV with sparse comb of long heavy setae and three distinct spinules. Leg I spination pattern: TSp 0, TSpv 0, TSrd 0, TSr 0, TSrv 8, MtSp 2, MtSr 6, TrSp 2, TrSr 6. *Pedipalps*. PTl 2.84, PTw 1.07, Bl 2.24. Embolus evenly curved.

**Figure 6.**
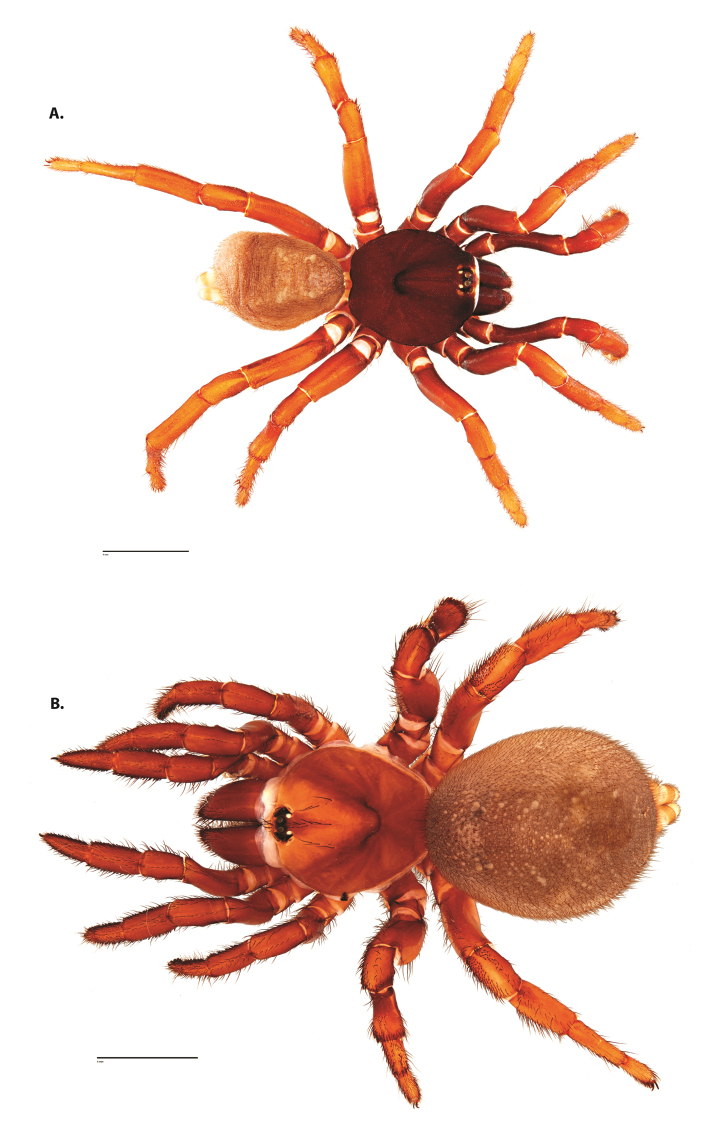
*Ummidia neilgaimani* sp. nov. from Roanoke Co., Virginia. **A** male habitus illustration UMM016 **B** female habitus illustration UMM240. Scale bars: 4.0 mm.

##### Variation, males

**(n = 16).**CL 4.5–6.37, 5.51±0.15; CW 4.66–6.4, 5.51±0.13; Cap 0.9–4.43, 3.58±0.2; ENDl 0.65–1.48, 0.84±0.05; ENDw 1.17–4.03, 1.51±0.17; STRl 2.62–3.6, 3.16±0.07; STRw 2.59–3.41, 3.04±0.06; LBl 0.7–1.01, 0.85±0.03; LBw 0.93–1.3, 1.09±0.03; F1 4.38–5.93, 5.03±0.12; F1w 1.03–1.43, 1.25±0.03; P1 2.06–2.81, 2.47±0.06; Ti1 2.8–3.83, 3.26±0.08; Mt1 1.8–2.69, 2.26±0.06; Tr1 1.02–1.4, 1.26±0.03; F3 3.23–4.46, 3.83±0.09; F3w 1.19–1.68, 1.47±0.04; P3 1.69–2.29, 1.94±0.05; Ti3 1.79–2.64, 2.25±0.06; Mt3 1.83–2.55, 2.11±0.05; Tr3 1.48–1.92, 1.72±0.03; F4 4.12–6.16, 5.04±0.13; F4w 1.16–1.6, 1.38±0.03; P4 1.94–2.62, 2.21±0.05; Ti4 2.59–3.87, 3.26±0.09; Mt4 2.77–3.95, 3.41±0.08; Tr4 1.48–2.4, 2.06±0.06; TSp 0–1, 0.06±0.06; TSpv 0–1, 0.13±0.09; TSr 0–1, 0.13±0.09; TSrv 5–9, 7.5±0.29; PTl 2.38–3.05, 2.68±0.06; PTw 0.9–1.18, 1.04±0.02; BL 1.83–4.41, 2.23±0.15.

##### Description of female paratype.

*Specimen preparation and condition*. Specimen preserved in 80% EtOH. Spermathecae removed, cleared, in vial with specimen. *General coloration*. Carapace, chelicerae, and legs reddish brown 5YR 4/4. Abdomen brown 7.5YR 4/2, spinnerets brownish yellow 10YR 6/6. *Cephalothorax*. Carapace 6.28 long, 5.81 wide. Pars cephalica 4.11 long. Foveal groove procurved, 0.54 long, 1.26 wide. Eye tubercle low, under ME. AER procurved PER slightly procurved. Eye group 0.82 long, 1.46 wide, AME 0.34, PME 0.25, ALE 0.44, PLE 0.31. Sternum sparsely setose around outer edges, STRl 3.53, STRw 3.39. Chelicerae with anterior row comprising eight teeth, posterior margin with seven teeth. Palpal endites with 27 cuspules spread across proximal half of endite and 37 cuspules distally; equal in size to proximal cuspules anteriorly, smaller posteriorly, ENDw 1.29, ENDl 2.37. Labium with row of four cuspules, LBw 1.21, LBl 0.93. Rastellum with many strong spines on process and up cheliceral face. *Abdomen*. Evenly setose with pale speckles concentrated at apodemes. *Legs*. F1 3.72; F1w 1.38; P1 2.32; Ti1 2.17; Mt1 1.43; Tr1 0.92; F3 3.15; F3w 1.71; P3 1.96; Ti3 1.88, Sd3 1.08; Mt3 1.49; Tr3 1.24; F4 4.37; F4w 1.69; P4 2.27; Ti4 2.43; Mt4 2.45; Tr4 1.26. Retrolateral face tarsus IV with poorly defined comb with doubled setae medially and short row of even heavy setae distally. *Pedipalps*. PF 3.34, PP 1.84, PTi 1.95, PTr 1.89. Spermathecae tilted medially with distal medial bend; bulbs facing anteriorly.

**Figure 7.**
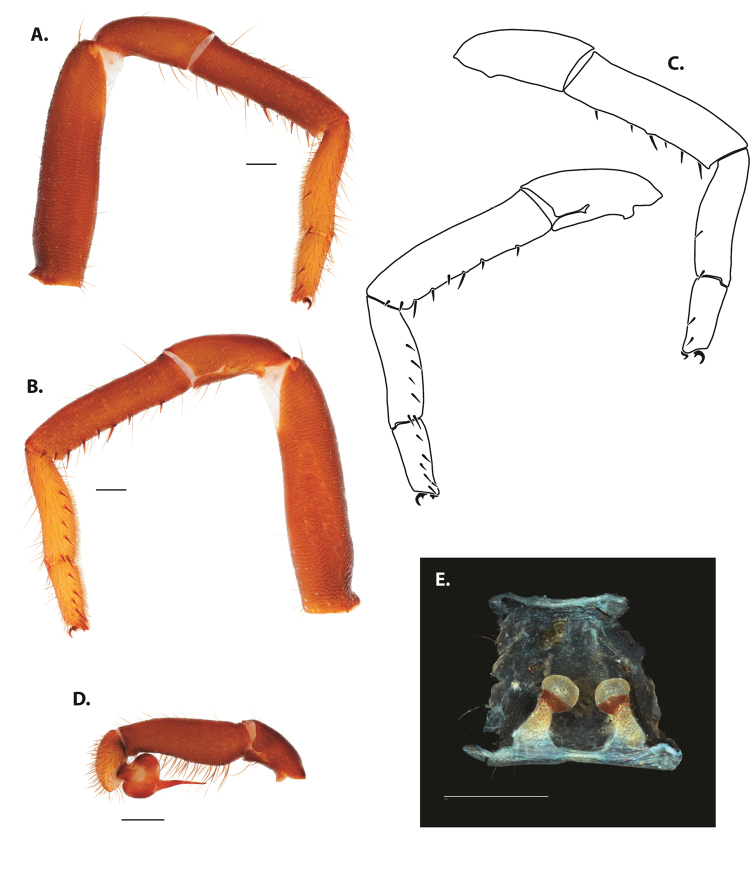
*Ummidia neilgaimani* sp. nov. from Roanoke Co., Virginia **A–D** male holotype (UMM016) **A** prolateral aspect, leg I **B** retrolateral aspect, leg I **C** line drawings, leg I prolateral and retrolateral aspects **D** retrolateral aspect, pedipalp **E** cleared spermathecae female paratype (UMM240). Scale bars: 1.0 mm.

##### Variation, females

**(n = 9).**CL 4.51–7.99, 6.3±0.32; CW 4.24–8.08, 6.27±0.37; Cap 3.17–5.55, 4.41±0.22; ENDl 0.6–1.15, 0.9±0.05; ENDw 1.07–1.94, 1.55±0.08; STRl 2.47–5.13, 3.78±0.28; STRw 2.56–4.96, 3.82±0.23; LBl 0.62–1.37, 1.04±0.07; LBw 1.09–1.88, 1.45±0.08; F1 2.9–4.94, 4.17±0.22; F1w 1.06–1.98, 1.47±0.08; P1 1.72–3.41, 2.6±0.16; Ti1 1.51–3.1, 2.44±0.16; Mt1 1.15–2.31, 1.73±0.12; Tr1 0.65–1.27, 1±0.06; F3 2.38–4.36, 3.58±0.2; F3w 1.26–2.31, 1.85±0.1; P3 1.47–2.86, 2.26±0.14; Ti3 1.28–2.62, 1.99±0.13; Mt3 1.1–2.12, 1.69±0.11; Tr3 0.97–1.83, 1.41±0.09; F4 3.23–5.5, 4.65±0.23; F4w 1.3–2.32, 1.81±0.09; P4 1.69–3.12, 2.48±0.14; Ti4 1.75–3.43, 2.6±0.16; Mt4 1.77–3.3, 2.63±0.15; Tr4 1.01–1.58, 1.35±0.07; PF 2.48–4.65, 3.83±0.24; PP 1.45–2.86, 2.16±0.14; PTi 1.35–3.04, 2.28±0.17; PTr 1.46–2.7, 2.13±0.13.

##### Material examined.

**United States: North Carolina: Clay Co**: Hayesville, 35.0461 -83.8179^6^, 576 m a.s.l. (UMM0277, 20.x.1911, 1♀, AF Archer, AMNH); **Durham Co**: Gate 3, Duke Forest, 1,000ft on N from 751, 35.9813 -78.9429^4^, 104 m a.s.l. (UMM0424, 7.xi.1963, 1♂, JW Berry, AMNH); **Wake Co**: 2.8 mi SSW of Garner, 35.6815 -78.5797^5^, 105 m a.s.l. (UMM0113, 22.xi.1996, 1♂, AL Braswell, NCSM); Raleigh, 35.7713 -78.6383^6^, 96 m a.s.l. (UMM0145, 29.xii.1903, 1♀, CUNHC); Chapel Hill, 35.9131 -79.0558^6^, 145 m a.s.l. (UMM0146, 6.iii.1886, 1♀, GF Atkinson, CUNHC); **South Carolina**: Sumter National Forest, 34.004 -82.2459^7^, 152 m a.s.l. (AUMS24767, 6.x.2017, 1♀, D Hennen, BME); **Virginia: Caroline Co**: Fort A. P. Hill, 38.1183 -77.2759^6^, 41 m a.s.l. (UMM0093, 4–6.x.1997, 1♂, VMNH); **Charlotte Co**: Red Oak, 36.7698 -78.6356^6^, 140 m a.s.l. (UMM0240, 2.v.1968, 1♀, WW Baker, AMNH); **Chesapeake Co**: Chesapeake City, Fentress NALF, Pocaty Creek, 36.7018 -76.1968^6^, 6 m a.s.l. (UMM0005, 13.x.1989, 1♂, KA Buhlmann, VMNH); (UMM0035, 21.ix.1989, 1♂, VMNH); (UMM0086, 5.i.1990, 1♂, VMNH); 36.7018 -76.1968^6^, 6 m a.s.l. (UMM0029, 24.xi.1989, 1♂, VMNH); **Cumberland Co**: 2km SSW of Columbia, 37.7365 -78.1723^5^, 103 m a.s.l. (UMM0069, 16.xi.1989, 1♂, JC Mitchell, VMNH); (UMM0001, 1♂, VMNH); (UMM0092, 19.x.1989, 1♂, VMNH); (UMM0095, 21.ix.1989, 1♂, VMNH); (UMM0078, 1♂, JC Mitchell, VMNH); 5.5 km SSW of Columbia, 37.7051 -78.1812^5^, 104m (UMM0094, 5.x.1989, 1♂, JC Mitchell, VMNH); 7km SSW of Columbia, 37.703 -78.1976^5^, 111m (UMM0033, 16.ix.1990, 1♂, JC Mitchell, VMNH); (UMM0089, 16.xi.1989, 1♂, VMNH); (UMM0057, 19.x.1989, 1♂, VMNH); (UMM0053, 30.ix.1990, 1♂, VMNH); (UMM0079, 5.x.1989, 1♂, VMNH); **Fluvanna Co**: 37.8525 -78.2487^7^, 82 m a.s.l. (UMM0077, 3.xii.1995, 1♂, M Bell, VMNH); **Franklin Co**: Smith Mtn Lake 4-H Center, PF ca 10 mi ENE of Rocky Mount, 37.0405 -79.7046^5^, 242 m a.s.l. (UMM0081, 20.vii.1994, 1♂, VMNH); (UMM0075, 20–24.xi.1994, 1♂, VMNH); **Henrico Co**: 1 mi W of Elko, Elko Natural Area Preserve, 37.4731 -77.2293^5^, 36 m a.s.l. (UMM0068, 22.ix.1989, 1♂, CA Pague, VMNH); (UMM0087, 23.x.1989, 1♂, VMNH); (UMM0101, 6.x.1989, 1♂, VMNH); **Henry Co**: Collinsville, Parkwood Ct. Tennis courts, 36.7388 -79.905^4^, 318m (UMM0066, 23.xi.1992, 1♂, JM Anderson, JL Rolfsmeyer, VMNH); Martinsville, 36.6913 -79.8726^5^, 309 m a.s.l. (UMM0017, xi.2001, 1♂, VMNH); Martinsville, Dundee Lane, 36.6616 -79.8337^4^, 232 m a.s.l. (UMM0008, 11.x.1995, 1♂, P Carter, VMNH); (UMM0063, xi.1988, 1♂, VMNH); Martinsville, Inside VMNH Building, 36.6872 -79.8645^4^, 312 m a.s.l. (UMM0024, 9.x.1995, 1♂, JM Anderson, VMNH); **Isle of Wight Co**: Blackwater Ecological Reserve, 7 km S of Zuni, 36.801 -76.8305^5^, 21 m a.s.l. (UMM0038, 23.xii.1984, 1♂, CA Pague, VMNH); nr Zuni, Blackwater Ecological Reserve, 36.8217 -76.8538^5^, 9 m a.s.l. (UMM0740, 18.ix.2005, 1♂, M Milne, BME); Zuni pine barrens, 36.8185 -76.8541^5^, 11 m a.s.l. (UMM0067, 30.xii.1985, 1♂, CA Pague, JS Foster, VMNH); Zuni pine barrens, 3.5 SW of Zuni, 36.8174 -76.8532^5^, 11 m a.s.l. (UMM0099b, 27.vii.1985, 1♂, CA Pague, VMNH); **King George Co**: Dahlgren, Naval Weapon Laboratory, 38.3333 -77.0391^5^, 6 m a.s.l. (UMM0090, 2.xi.1991, 1♂, KA Buhlmann, VMNH); (UMM0043, 7.x.1991, 1♂, VMNH); (UMM0014, 9.xii.1991, 1♂, VMNH); **Northampton Co**: Savage Neck Dunes Natural Area Preserve, 37.3253 -76.0112^5^, 5 m a.s.l. (UMM0031, 12.xi.2003, 1♂, D Field, VMNH); (UMM0018, 23–28.x.1999, 1♂, AC Chazal, SM Roble, VMNH); (UMM0022, 27.viii–23.ix.1999, 1♂, SM Roble, VMNH); Savage Neck Dunes Natural Area Preserve, 0.26 km WSW Curtis Pond, 37.3281 -76.0114^4^, 4 m a.s.l. (UMM0020, 28.x.2003, 1♂, Erdle & Field, VMNH); **Pittsylvania Co**: Lacy Farm, ca 4 mi ENE of Axton, 36.6764 -79.6415^5^, 227 m a.s.l. (UMM0100, 13.xi–21.xii.1992, 1♂, VMNH); **Prince Edward Co**: Farmville, Price Drive, 37.2852 -78.381^4^, 129 m a.s.l. (UMM0040, 11.xi.1991, 1♂, WA Shear, VMNH); (UMM0054, 9.x.1995, 1♂, VMNH); Hampden Sydney College, Library Parking Lot, 37.241 -78.4631^4^, 164 m a.s.l. (UMM0234, 26.i.1975, 1♂, S Atkinson, AMNH); **Prince William Co**: Prince William Forest Park, 38.5924 -77.3848^6^, 89 m a.s.l. (UMM0065, 15.ii.1989, 1♂, VMNH); (UMM0023, 7.iv.1989, 1♀, VMNH); (UMM0011, 9.i.1989, 1♂, VMNH); **Richmond Co**: Richmond, Willow Oaks Apts at Powhite Pkwy/Forest Hills Ave, 37.5313 -77.5067^4^, 53 m a.s.l. (UMM0096, 1.x.1995, 1♂, D Stevenson, VMNH); **Roanoke Co**: Roanoke, Virginia Western Community College, 37.2462 -79.9744^4^, 333 m a.s.l. (UMM0016, 26.x.1995, 1♂, MW Donahue, VMNH); **Virginia Beach Co**: Fort Story, Hospital Road, 36.9244 -76.0243^4^, 4 m a.s.l. (UMM0050, 22.vi.1995, 1♀, DA Young, VMNH); Pungo, 36.7218 -76.0173^6^, 2 m a.s.l. (UMM0071, 20.v.1990, 1♀, NL Bland, VMNH); Virginia Beach City, Little Creek Amphibious Base, 36.9005 -76.1591^6^, 3 m a.s.l. (UMM0019, 1♂, KA Buhlmann, VMNH); Virginia Beach City, Oceana NAS, Owl Creek, 36.8225 -75.983^5^, 0 m a.s.l. (UMM0032, 1.xi.1989, 1♂, KA Buhlmann, VMNH); (UMM0004, 24.xi.1989, 1♂, VMNH); Virginia Beach City, Seashore State Park, 36.9185 -76.0523^5^, 0 m a.s.l. (UMM0080, 1.xi.1989, 1♂, KA Buhlmann, VMNH); (UMM0097, 1.xi.1989, 1♂, KA Buhlmann, VMNH); (UMM0058, 13.x.1989, 1♂, KA Buhlmann, VMNH); (UMM0061, 13.x.1989, 1♂, KA Buhlmann, VMNH); (UMM0055, 29.ix.1989, 1♂, KA Buhlmann, VMNH); (UMM0013, 5.vii.1989, 1♂, KA Buhlmann, VMNH); (UMM0062, 5.xii.1989, 1♂, KA Buhlmann, VMNH); Virginia Beach City, Seashore State Park, 36.9185 -76.0523^5^, 0 m a.s.l. (UMM0002, 16.xi.1989, 1♂, VMNH); (UMM0074, 29.ix.1989, 1♂, VMNH); **York Co**: Cheatham Annex Naval Supply Base, Jones Mill, 37.2774 -76.6414^4^, 7 m a.s.l. (UMM0037, 2.xi.1989, 1♂, KA Buhlmann, VMNH); (UMM0006, 24.ix.1989, 1♂, VMNH); Cheatham Annex Navy Base, Jones Millpond, Rt 199 and Colonial Parkway, 37.2774 -76.6414^4^, 7 m a.s.l. (UMM0060, 17.xi.1989, 1♂, KA Buhlmann, CA Pague, VMNH); Grafton Ponds, 37.1653 -76.4685^6^, 15 m a.s.l. (UMM0044, 17.viii.1990, 1♀, KA Buhlmann, VMNH); (UMM0045, 30.xi.1989, 1♂, VMNH); (UMM0046, 1♂, VMNH); (UMM0047, 1♂, VMNH); Yorktown Naval Weapons Station, 37.2437 -76.5835^5^, 13 m a.s.l. (UMM0052, 30.xi.1990, 1♂, KA Buhlmann, VMNH); (UMM0070, 1♂, VMNH); (UMM0091, 1♂, VMNH).

#### Ummidia gingoteague sp. nov.

Taxon classificationAnimaliaAraneaeHalonoproctidae

4DA3045F-4AED-5007-9D7C-19D4C9695731

http://zoobank.org/93A1A87C-0A44-46DC-AD11-1A3D5A9D7A56

Figs 8 , 9 , Map 3

##### Type material.

HOLOTYPE: 1 ♂ (UMM003) from Chincoteague National Wildlife Refuge, White Hills Ridge, 1.3 km N toll booth. Accomack County, Virginia, United States, 37.9083 -75.3568^5^, 2 m a.s.l., 26.vi–10.vii.1998, coll. SM Roble, VMNH. PARATYPE: 1 ♀ (AUMS16243) from Newport News, Mariner’s Museum Park, Newport News County, Virginia, United States, 37.0588 -76.4846^1^, 18 m a.s.l., 9.vii.2015, coll. RL Godwin, BME.

##### Etymology.

The specific epithet is a noun taken in apposition and is in reference to the Native American name for the type locality.

##### Diagnosis.

*Ummidia gingoteague* can be differentiated from all other eastern United States species on the basis of its small size. *Ummidia gingoteague* can be differentiated from *U. audouini* by the presence of a comb on the retrolateral face of tarsus IV, which is more defined than the comb in *U. neilgaimani*. Males can further be distinguished from *U. neilgaimani*, *U. rongodwini*, and *U. okefenokee* by the presence of spines on the prolateral aspect of tibia I and from *U. rongodwini* by lacking a pale dorsal heart patch. Males disperse from May to July.

**Figure 8.**
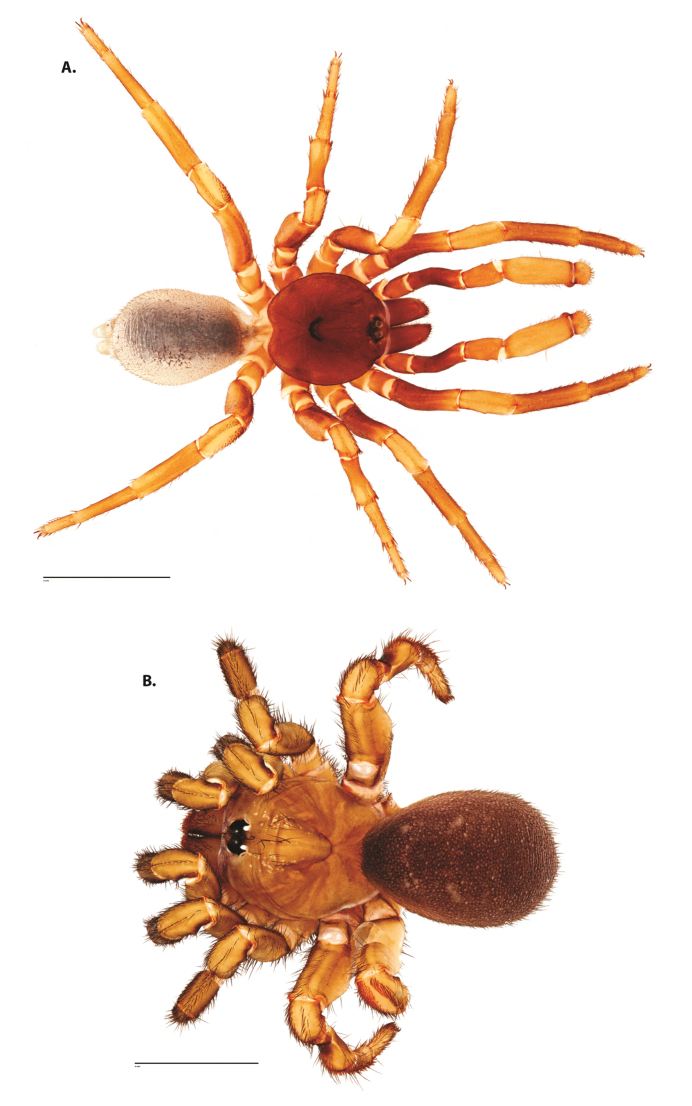
*Ummidia gingoteague* sp. nov. from Accomack Co. Virginia **A** male habitus illustration UMM003 **B** female habitus illustration AUMS16243. Scale bars: 4.0 mm.

##### Description of male holotype.

*Specimen preparation and condition*. Specimen preserved in 80% EtOH. Left palp, leg I removed, in vial with specimen. *General coloration*. Carapace dark reddish brown 2.5YR 2.5/3, legs brown 7.5YR 5/6. Abdomen very dark gray GLEY 1 3/N. *Cephalothorax*. Carapace 3.48 long, 3.49 wide. Pars cephalica 2.51 long. Foveal groove procurved, 0.3 long, 0.71 wide. All eyes on moderate tubercle. AER procurved. PER straight. Eye group 0.94 long, 2.39 wide, AME 0.26, PME 0.15, ALE 0.31, PLE 0.18. Sternum sparsely setose anteriorly with posterior fringe, STRl 2.01, STRw 1.99. Chelicerae with anterior tooth row comprising six teeth, posterior margin with five teeth. Palpal endites with 15 long, thin cuspules over proximal half of endite face, and three long, thin cuspules distally, ENDw 0.87, ENDl 1.42. Labium with six cuspules, LBw 0.79, LBl 0.56. Rastellum with many spines on process. Abdomen setose. *Legs*. F1 3.66; F1w 0.75; P1 1.74; Ti1 2.49; Mt1 1.62; Tr1 0.78; F3 2.46; F3w 0.95; P3 1.31; Ti3 1.48; Sd3 0.87; Mt3 1.33; Tr3 1.18; F4 3.54; F4w 0.96; P4 1.46; Ti4 2.28; Mt4 2.36; Tr4 1.22. Retrolateral face of tarsus IV with defined comb over length of tarsus. Leg I spination pattern: TSp 4, TSpv 5, TSrd 0, TSr 0, TSrv 19, MtSp 3, MtSr 6, TrSp 3, TrSr 6. *Pedipalps*. PTl 2.05, PTw 0.84, Bl 1.71. Embolus evenly curved.

##### Variation, males

**(n = 12).**CL 3.48–5.24, 4.12±0.14; CW 3.43–5.04, 4.11±0.14; Cap 0.59–3.69, 2.65±0.21; ENDl 0.49–0.94, 0.64±0.03; ENDw 0.92–2.39, 1.17±0.11; STRl 2.01–2.82, 2.43±0.08; STRw 1.06–2.61, 2.18±0.12; LBl 0.46–0.76, 0.63±0.03; LBw 0.63–1.14, 0.89±0.04; F1 3.33–4.46, 3.95±0.1; F1w 0.75–1.14, 0.92±0.03; P1 1.64–2.18, 1.91±0.05; Ti1 2.19–3.1, 2.7±0.08; Mt1 1.47–2.1, 1.82±0.06; Tr1 0.78–1.03, 0.92±0.02; F3 2.26–3.17, 2.79±0.08; F3w 0.95–1.35, 1.12±0.03; P3 1.1–1.69, 1.46±0.05; Ti3 1.33–1.81, 1.61±0.04; Mt3 1.33–1.86, 1.62±0.05; Tr3 1.09–1.56, 1.34±0.04; F4 3.16–4.19, 3.71±0.1; F4w 0.92–1.27, 1.1±0.03; P4 1.36–1.96, 1.65±0.05; Ti4 2.02–2.88, 2.5±0.08; Mt4 2.08–2.95, 2.64±0.08; Tr4 1.21–1.77, 1.48±0.05; TSp 1–8, 4.92±0.51; TSpv 2–10, 6.17±0.66; TSr 0–3, 1.25±0.28; TSrv 12–28, 20.75±1.26; PTl 1.96–2.76, 2.27±0.07; PTw 0.78–1, 0.88±0.02; BL 1.65–1.93, 1.78±0.03.

##### Description of female paratype.

*Specimen preparation and condition*. Specimen preserved in 80% EtOH. Spermathecae removed and cleared, in vial with specimen. Right leg III and IV in 100% EtOH. *General coloration*. Carapace, chelicerae, and legs dark yellowish brown 10YR 4/6. Abdomen very dark gray 7.5YR 3/1, spinnerets yellowish brown 10YR 5/6. *Cephalothorax*. Carapace 4.88 long, 4.53 wide. Pars cephalica 3.27 long. Foveal groove procurved, 0.49 long, 1.0 wide. Eye tubercle very low under AME only. AER procurved PER straight. Eye group 0.7 long, 1.26 wide, AME 0.28, PME 0.19, ALE 0.42, PLE 0.29. Sternum sparsely setose around outer edges, STRl 2.95, STRw 2.93. Chelicerae with anterior row comprising seven teeth, posterior margin with five teeth. Palpal endites with 24 cuspules spread across proximal half of endite and 58 cuspules distally; equal in size to proximal cuspules anteriorly; smaller posteriorly, ENDw 1.2, ENDl 1.89. Labium with three cuspules, LBw 1.16, LBl 0.86. Rastellum with many strong spines on process, along distal cheliceral margin, and up cheliceral face for ~ 3× length of process. *Abdomen*. Evenly setose with patches of pale speckles at dorsal apodemes. *Legs*. F1 3.48; F1w 1.24; P1 2.23; Ti1 2.11; Mt1 1.47; Tr1 0.91; F3 2.9; F3w 1.46; P3 1.64; Ti3 1.64, Sd3 1.03; Mt3 1.29; Tr3 1.84; F4 3.77; F4w 1.58; P4 1.83; Ti4 2.09; Mt4 2.06; Tr4 1.23. Retrolateral face tarsus IV with defined comb over 2/3 of article. *Pedipalps*. PF 2.78, PP 1.77, PTi 1.7, PTr 1.84. Spermathecae bent medially, bulbs facing anteromedially.

**Figure 9.**
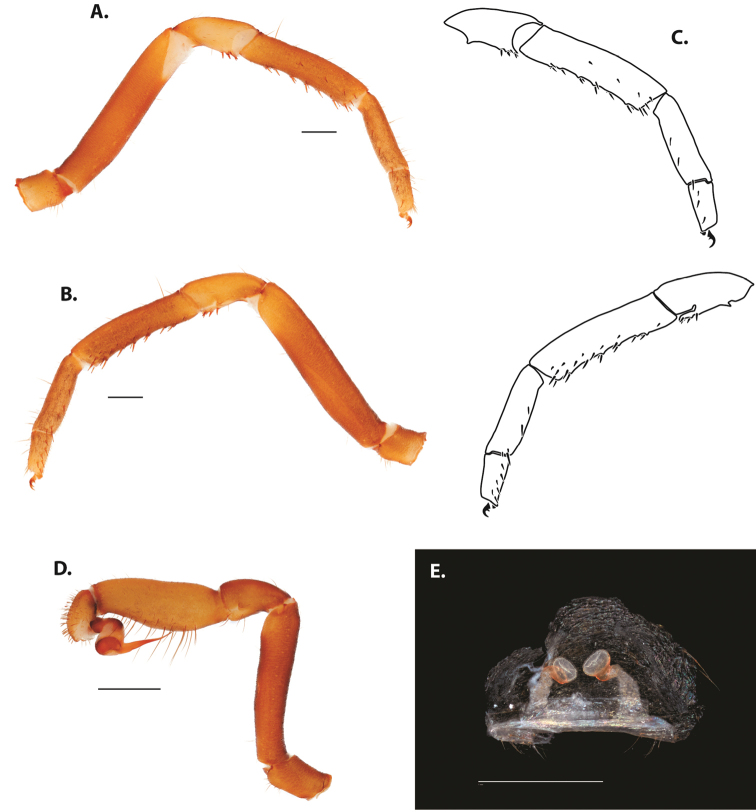
*Ummidia gingoteague* sp. nov. from Accomack Co. Virginia **A–D** male holotype (UMM003) **A** prolateral aspect, leg I **B** retrolateral aspect, leg I **C** line drawings, leg I prolateral and retrolateral aspects **D** retrolateral aspect, pedipalp **E** cleared spermathecae female paratype (AUMS16243). Scale bars: 1.0 mm.

##### Variation, females

**(n = 4).**CL 4.45–4.88, 4.68±0.12; CW 4.21–4.55, 4.43±0.08; Cap 3.01–3.27, 3.15±0.07; ENDl 0.52–0.7, 0.63±0.04; ENDw 0.92–1.26, 1.09±0.07; STRl 2.65–2.95, 2.8±0.07; STRw 2.52–2.97, 2.76±0.11; LBl 0.63–0.86, 0.76±0.06; LBw 0.98–1.16, 1.07±0.05; F1 2.69–3.48, 3.01±0.19; F1w 0.89–1.24, 1.07±0.08; P1 1.72–2.23, 1.95±0.11; Ti1 1.53–2.11, 1.76±0.13; Mt1 1.12–1.47, 1.3±0.09; Tr1 0.61–1.66, 1±0.23; F3 2.24–2.9, 2.53±0.15; F3w 1.26–1.46, 1.36±0.05; P3 1.34–1.64, 1.5±0.07; Ti3 1.23–1.64, 1.4±0.09; Mt3 1.07–1.3, 1.2±0.06; Tr3 0.99–1.23, 1.11±0.06; F4 2.85–3.77, 3.25±0.22; F4w 1.27–1.58, 1.39±0.07; P4 1.57–1.84, 1.71±0.07; Ti4 1.7–2.09, 1.89±0.1; Mt4 1.74–2.06, 1.89±0.08; Tr4 0.9–1.23, 1.07±0.08; PF 2.42–2.82, 2.62±0.11; PP 1.43–1.77, 1.6±0.08; PTi 1.4–1.7, 1.57±0.07; PTr 1.44–1.84, 1.58±0.09.

##### Material examined.

**United States: Virginia: Accomack Co**: Chincoteague National Wildlife Refuge, White Hills Ridge, 1.3km N toll booth, 37.9083 -75.3568^5^, 2 m a.s.l. (UMM0003, 26.vi-10.vii.1998, 1♂, SM Roble, VMNH); **Greensville Co**: end of Va. 666, 1mi NE Claresville, 36.6259 -77.4422^5^, 23 m a.s.l. (UMM0085, 21.vi-14.vii.1993, 1♂, VMNH); **Isle of Wight Co**: Blackwater Ecological Reserve, 4mi S of Zuni, Rt 614, 36.8121 -76.8535^5^, 9 m a.s.l. (UMM0009, 26.v.1995, 1♂, SM Roble, VMNH); **Newport News Co**: Newport News, Mariner’s Museum Park, 37.0588 -76.4846^1^, 18 m a.s.l. (AUMS016243, 9.vii.2015, 1♀, RL Godwin, BME); (AUMS016244, 1♀, BME); (AUMS016245, 1juv, BME); **Prince Edward Co**: Twin Lakes State Park, 37.1748 -78.2732^5^, 134 m a.s.l. (UMM0049, viii-ix.1997, 1juv, VMNH); **Virginia Beach Co**: Virginia Beach City, First Landing State Park, 36.9198 -76.0436^1^, 11 m a.s.l. (AUMS016242, 7.vii.2015, 1♀, RL Godwin, BME); (UMM0088, 21.vi.1989, 1♂, VMNH); (UMM0084, 21.vi.1989, 1♂, KA Buhlmann, VMNH); (UMM0056, 26.vii.1989, 1♂, VMNH); (UMM0012, 1♂, VMNH); (UMM0076, 29.ix.1989, 1♀, VMNH); (UMM0231, 1♀, AMNH); (UMM0021, 5.vii.1989, VMNH); (UMM0034, 5.vii.1989, 1♂, VMNH); (UMM0007, 1♂, VMNH); **York Co**: Cheatham Annex, US Naval Supply Center, 6mi NW of Yorktown, 37.279 -76.609^5^, 7 m a.s.l. (UMM0048, 6.vi.1989, 1♂, KA Buhlmann, VMNH); Cheatham Annex, US Naval Supply Center, Cheatham Pond, 37.2951 -76.6185^4^, 0 m a.s.l. (UMM0030, 19.vi.1989, 1♂, VMNH); Cheatham Annex, US Naval Supply Center, Cheatham Pond, 37.2951 -76.6185^4^, 0 m a.s.l. (UMM0015, 30.v.1990, 1♂, KA Buhlmann, VMNH).

#### Ummidia carabivora

Taxon classificationAnimaliaAraneaeHalonoproctidae

(Atkinson, 1886)

40C8996B-A931-5FD1-AE27-95D249D5CED4

**Figure 10.**
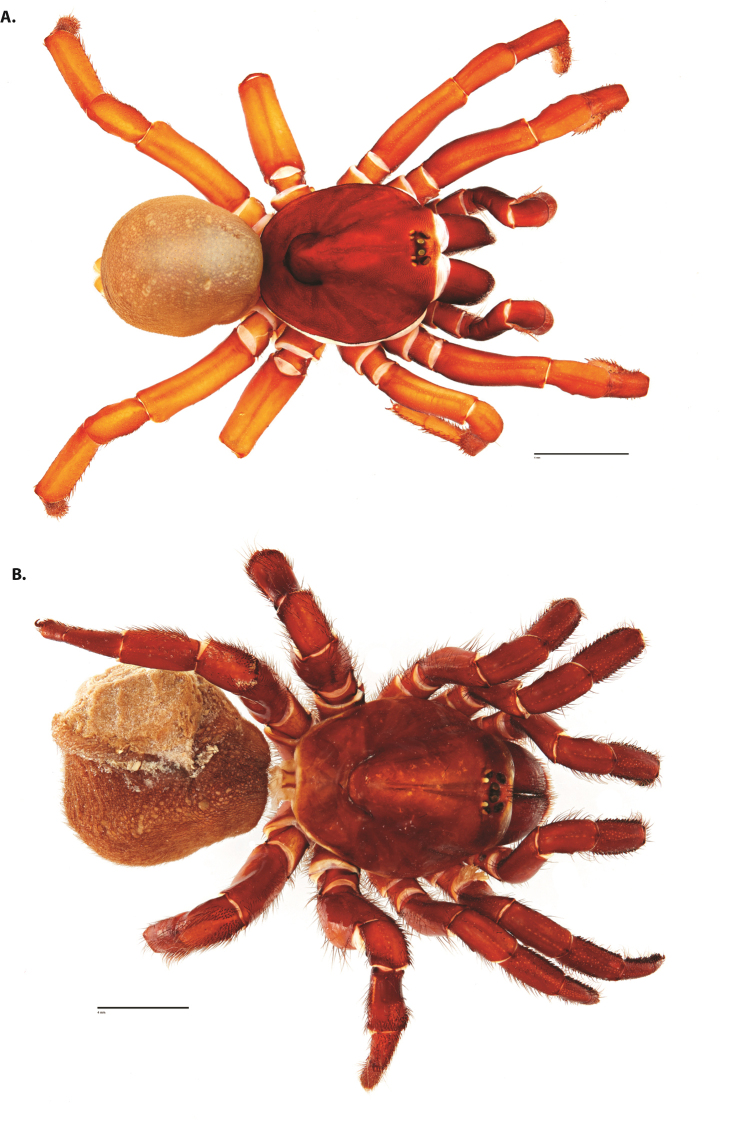
*Ummidia carabivora* (Atkinson, 1886) from Chapel Hill, North Carolina **A** male habitus illustration UMM073 **B** female habitus illustration A47. Scale bars: 4.0 mm.

**Figure 11.**
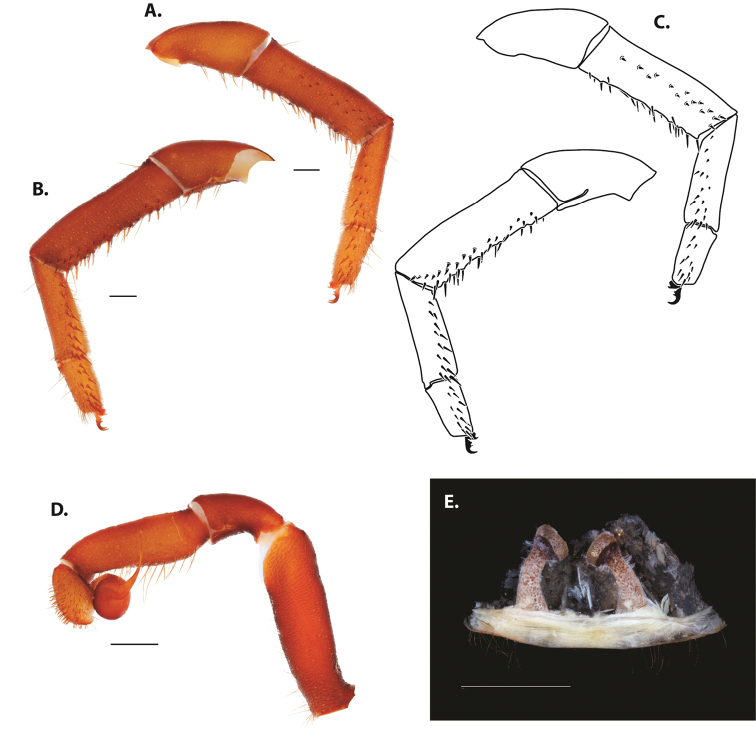
*Ummidia carabivora* (Atkinson, 1886) from Chapel Hill, North Carolina **A–D** male exemplar (UMM073) **A** prolateral aspect, leg I **B** retrolateral aspect, leg I **C** line drawings, leg I prolateral and retrolateral aspects **D** retrolateral aspect, pedipalp **E** cleared spermathecae female holotype (A47). Scale bars: 1.0 mm.

Figs 10 , 11 , Map 3

Pachylomerus carabivorus Petrunkevitch, 1929: 511; HOLOTYPE: 1 ♀ (A-47) from Chapel Hill, North Carolina, United States, 35.9131 -79.0557^6^, 147 m a.s.l., deposited in USNM Marx Collection, examined.
Pachylomerus carabivorus emarginatus Atkinson, 1886: 134, **syn. nov.**

##### Diagnosis.

*Ummidia carabivora* can be differentiated from all other species of *Ummidia* by the pars cephalica which extends 4/5 (7/10 in other species) the length of the carapace and raised dorsally at the fovea which is 1/3 (1/4–1/5 in other species) the width of the carapace. Males can further be distinguished from all other eastern United States species with tarsal combs by tibia I being relatively more spinose both on the prolateral and retrolateral aspects. Males disperse from June to July.

##### Description of female holotype.

*Specimen preparation and condition*. Specimen preserved in 80% EtOH. Spermathecae removed, cleared, in vial with specimen. *General coloration*. Carapace, chelicerae, and legs very dusky red 10R 2.5/2. Abdomen very dark brown 7.5YR 2.5/3, spinnerets dark brown 7.5YR 3/4. *Cephalothorax*. Carapace 9.07 long, 8.3 wide. Pars cephalica 7.21 long. Foveal groove procurved and extremely broad, 1.11 long, 2.81 wide. Eye tubercle low. AER very procurved PER very slightly procurved. Eye group 1.11 long, 2.1 wide, AME 0.46, PME 0.31, ALE 0.56, PLE 0.37. Sternum sparsely setose around anterior edges with posterior fringe, STRl 5.41, STRw 5.3. Chelicerae with anterior row comprising six teeth, posterior margin with six teeth. Palpal endites with 25 cuspules spread across proximal half of endite and 37 cuspules distally, ENDw 1.98, ENDl 3.38. Labium with five cuspules, LBw 1.93, LBl 1.36. Rastellum with many strong spines on process. *Abdomen*. Evenly setose. *Legs*. F1 5.54; F1w 1.91; P1 3.37; Ti1 3.41; Mt1 2.34; Tr1 1.32; F3 4.73; F3w 2.51; P3 3.02; Ti3 2.74, Sd3 1.77; Mt3 2.15; Tr3 2.11; F4 5.99; F4w 2.38; P4 3.53; Ti4 3.52; Mt4 3.13; Tr4 1.71. Retrolateral face tarsus IV with defined comb. *Pedipalps*. PF 4.8, PP 2.67, PTi 3.1, PTr 2.68. Spermathecae straight with slight medial tilt, bulbs facing anteromedially.

##### Variation, females.

Known only from female holotype specimen.

##### Description of male exemplar.

*Specimen preparation and condition*. Specimen preserved in 80% EtOH. Left palp, leg I removed, in vial with specimen. *General coloration*. Carapace and chelicerae dusky red 10R ¾, legs strong brown 7.5YR 4/6. Abdomen dark brown 10YR 3/3. *Cephalothorax*. Carapace 8.05 long, 7.16 wide. Pars cephalica 6.85 long. Foveal groove broadly procurved, 1.48 long, 2.34 wide. ME on moderate tubercle. AER procurved. PER straight. Eye group 0.93 long, 1.66 wide, AME 0.48, PME 0.24, ALE 0.47, PLE 0.25. Sternum sparsely setose anteriorly with posterior fringe, STRl 3.98, STRw 4.04. Chelicerae with anterior tooth row comprising four teeth, posterior margin with six teeth. Palpal endites with 19 small cuspules across proximal half of endite face, lacking distal endite cuspules, ENDw 1.58, ENDl 2.86. Labium with five cuspules, LBw 1.47, LBl 1.11. Rastellar process narrow and extended medially; covered in small spines. Abdomen setose, setae very short. *Legs*. F1 5.71; F1w 1.84; P1 2.92; Ti1 3.68; Mt1 2.61; Tr1 1.31; F3 4.57; F3w 2.05; P3 2.56; Ti3 2.48; Sd3 1.49; Mt3 2.52; Tr3 2.1; F4 5.49; F4w 2.1; P4 2.71; Ti4 3.73; Mt4 3.66; Tr4 1.82. Retrolateral face of tarsus IV with defined comb over central half of tarsus. Leg I spination pattern: TSp 15, TSpv 4, TSrd 0, TSr 3, TSrv 31, MtSp 17, MtSr 14, TrSp 12, TrSr 13. *Pedipalps*. PTl 3.05, PTw 1.16, Bl 2.3. Embolus evenly curved.

##### Variation, males

**(n = 3).**CL 6.16–8.85, 7.69±0.8; CW 5.54–8.23, 6.98±0.78; Cap 4.95–6.85, 6.15±0.6; ENDl 0.59–1.07, 0.86±0.14; ENDw 1.34–1.75, 1.58±0.12; STRl 3.17–4.28, 3.81±0.33; STRw 3.05–4.75, 3.95±0.49; LBl 0.77–1.11, 0.94±0.17; LBw 1.08–1.47, 1.28±0.2; F1 4.35–5.79, 5.28±0.47; F1w 1.38–2.03, 1.75±0.19; P1 2.26–3.18, 2.78±0.27; Ti1 2.92–3.68, 3.38±0.23; Mt1 2.02–2.81, 2.48±0.24; Tr1 1.08–1.48, 1.29±0.12; F3 3.37–5.06, 4.33±0.5; F3w 1.44–2.14, 1.88±0.22; P3 1.84–2.56, 2.31±0.24; Ti3 1.88–2.81, 2.39±0.27; Mt3 1.86–2.75, 2.38±0.27; Tr3 1.61–2.25, 1.98±0.19; F4 4.46–6.37, 5.44±0.55; F4w 1.46–2.2, 1.92±0.23; P4 2.09–3.18, 2.66±0.32; Ti4 2.79–3.85, 3.46±0.34; Mt4 3.13–4.08, 3.62±0.27; Tr4 1.82–2.28, 1.98±0.15; TSp 4–17, 9.33±3.93; TSpv 2–6, 4±1.15; TSr 1–3, 2±0.58; TSrv 25–31, 28.33±1.76; PTl 2.62–6.05, 4.02±1.04; PTw 0.98–1.29, 1.14±0.09; BL 1.96–2.3, 2.13±0.1.

##### Material examined.

**United States: North Carolina**: Chapel Hill, 35.9131 -79.0557^6^, 147 m a.s.l. (A-47, 1♀, USNM); **Cumberland Co**: Cool Springs, 35.845 -80.7321^5^, 272 m a.s.l. (UMM0691, 1♂, BME); **Virginia: Virginia Beach Co**: Virginia Beach City, Seashore State Park, 36.9185 -76.0523^5^, 0 m a.s.l. (UMM0073, 21.vi.1989, 1♂, VMNH); (UMM0025, 23.vi-6.vii-2003, 1♂, R Vigneault, VMNH).

#### Ummidia rongodwini sp. nov.

Taxon classificationAnimaliaAraneaeHalonoproctidae

419374FC-ED6C-5491-80B9-272BCD7E8857

http://zoobank.org/11514E3A-93CF-4CFC-943E-7AC879BBE784

**Figure 12.**
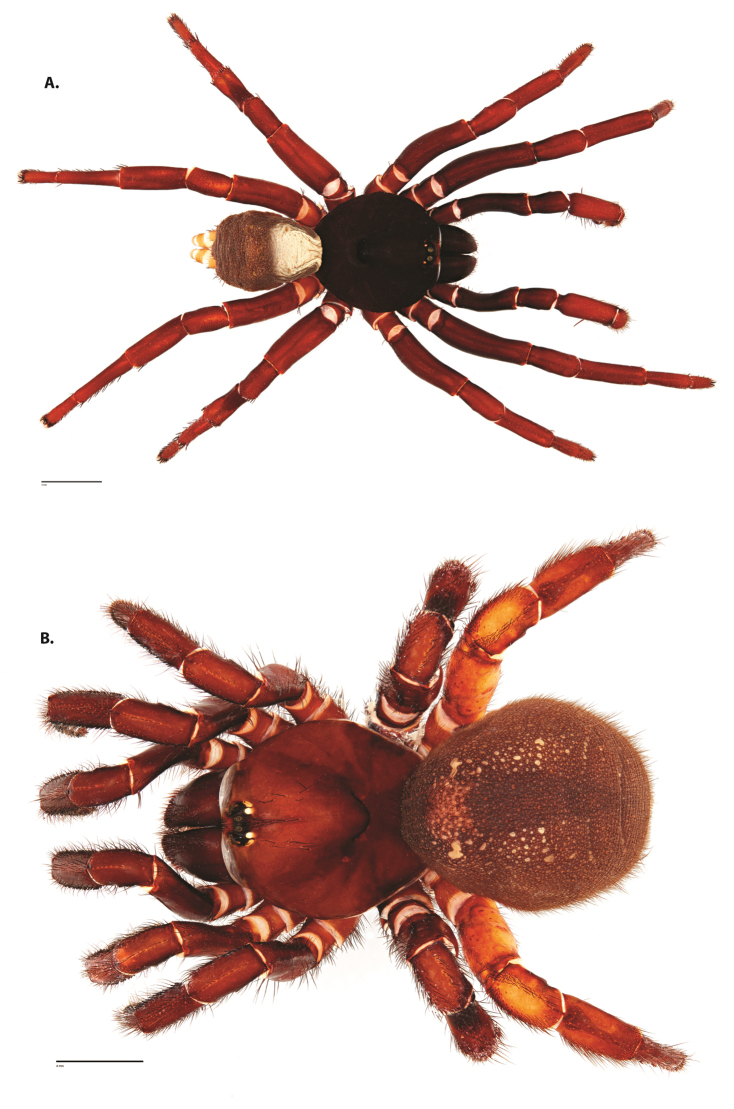
*Ummidia rongodwini* sp. nov. **A** male habitus illustration MY2313b **B** female habitus illustration AUMS4565. Scale bars: 4.0 mm.

**Figure 13.**
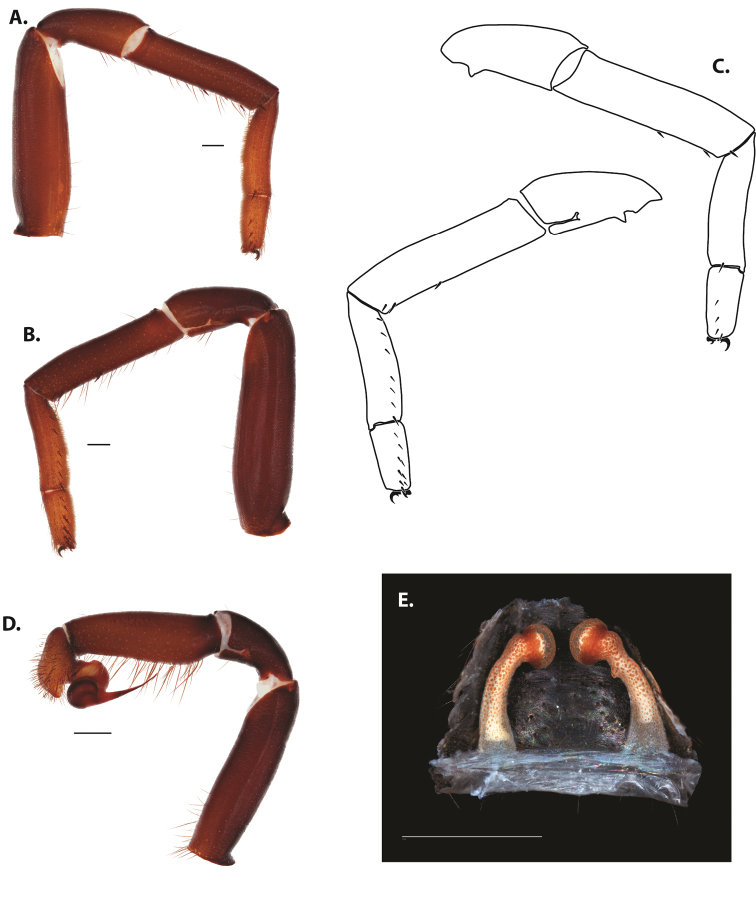
*Ummidia rongodwini* sp. nov. from Calhoun Co., Alabama **A–D** male holotype (UMM367) **A** prolateral aspect, leg I **B** retrolateral aspect, leg I **C** line drawings, leg I prolateral and retrolateral aspects **D** retrolateral aspect, pedipalp **E** cleared spermathecae female paratype (AUMS4565). Scale bars: 1.0 mm.

Figs 12 , 13 , Map 3

##### Type material.

HOLOTYPE: 1 ♂ (UMM367) from 7mi N Jacksonville, on Nisbet Lake Rd, Calhoun County, Alabama, United States, 33.8795 -85.7889^4^, 233 m a.s.l., 29.viii.1979, coll. A Boozer, AMNH. PARATYPE: 1 ♀ (AUMS4565) from off Hwy 39 east of Walter F. George Reservoir, Quitman County, Georgia, United States, 31.7902 -85.0916^1^, 104 m a.s.l., 24.iii.2013, coll. CA Hamilton, BME.

##### Etymology.

The specific epithet is a patronym named for the first author’s husband and partner Ron Godwin as an expression of gratitude for his love and support throughout her graduate career. The second author likes Ron but not as much as the first author.

##### Diagnosis.

*Ummidia rongodwini* males can be differentiated from *U. audouini*, *U. neilgaimani*, *U. gingoteague*, *U. carabivora*, and *U. okefenokee* by the presence of a white to opalescent dorsal heart patch and from *U. richmond* by having eyes relatively larger and closer to the anterior margin of the carapace. Males disperse from September to November.

##### Description of male holotype.

*Specimen preparation and condition*. Specimen preserved in 80% EtOH. Left palp and leg I removed, in vial with specimen. *General coloration*. Carapace and chelicerae reddish black 2.5YR 2.5/1, legs very dark brown 7.5YR 2.5/3. Abdomen black 5YR 2.5/1 with heart patch light gray 2.5Y 7/2. *Cephalothorax*. Carapace 7.91 long, 7.68 wide. Pars cephalica 5.27 long. Foveal groove procurved, 0.75 long, 1.68 wide. All eyes on moderate tubercle. AER procurved. PER straight. Eye group 0.95 long, 1.68 wide, AME 0.42, PME 0.22, ALE 0.44, PLE 0.27. Sternum sparsely setose with fringe around margin, more setose anteriorly, STRl 4.54, STRw 4.75. Chelicerae with anterior tooth row comprising five teeth, posterior margin with five teeth. Palpal endites with 35 tiny cuspules, lacking distal endite cuspules, ENDw 1.84, ENDl 3.24. Labium with six cuspules, LBw 1.56, LBl 1.32. Rastellum reduced with only small denticles on process. Abdomen sparsely setose; setae short. *Legs*. F1 6.79; F1w 1.86; P1 3.47; Ti1 4.81; Mt1 3.27; Tr1 1.65; F3 5.1; F3w 2.12; P3 2.64; Ti3 2.89; Sd3 1.53; Mt3 2.96; Tr3 2.11; F4 6.38; F4w 1.97; P4 3.14; Ti4 4.28; Mt4 4.54; Tr4 2.44. Retrolateral face of tarsus IV with defined comb of heavy setae over proximal 2/3 transitioning to spinules over distal 1/3. Leg I spination pattern: TSp 0, TSpv 0, TSrd 0, TSr 0, TSrv 4, MtSp 1, MtSr 7, TrSp 4, TrSr 8. *Pedipalps*. PTl 4.02, PTw 1.39, Bl 2.73. Embolus evenly curved.

##### Variation, males

**(n = 47).**CL 3.72–9.23, 7.18±0.2; CW 3.49–9.02, 7.06±0.2; Cap 2.33–7.7, 4.9±0.15; ENDl 0.51–1.19, 0.84±0.02; ENDw 1.01–2.08, 1.63±0.04; STRl 2.16–5.2, 4.21±0.11; STRw 2–5.55, 4.08±0.11; LBl 0.58–1.57, 1.12±0.04; LBw 0.49–2.1, 1.47±0.05; F1 3.82–7.82, 6.03±0.15; F1w 0.75–2.14, 1.69±0.05; P1 1.78–3.96, 3.11±0.08; Ti1 2.52–5.02, 4.09±0.1; Mt1 1.7–3.81, 2.94±0.07; Tr1 0.84–1.86, 1.47±0.03; F3 2.63–5.78, 4.52±0.12; F3w 0.96–2.42, 1.94±0.05; P3 1.32–3.18, 2.46±0.07; Ti3 0.39–3.56, 2.61±0.08; Mt3 1.42–3.43, 2.58±0.07; Tr3 1.19–2.72, 1.99±0.05; F4 3.57–7.32, 5.89±0.15; F4w 0.95–2.65, 1.87±0.05; P4 1.51–3.7, 2.8±0.08; Ti4 2.34–4.86, 3.92±0.09; Mt4 2.37–5.01, 3.99±0.1; Tr4 1.33–3.06, 2.16±0.05; TSp 0–6, 0.19±0.13; TSpv 0–7, 0.36±0.16; TSr 0–11, 0.62±0.35; TSrv 0–13, 3.7±0.37; PTl 2.47–4.62, 3.54±0.08; PTw 0.78–4.49, 1.37±0.07; BL 1.41–3.32, 2.72±0.06.

##### Description of female paratype.

*Specimen preparation and condition*. Specimen preserved in 80% EtOH. Spermathecae removed and cleared, in vial with specimen. Right leg III and IV in 100% EtOH. *General coloration*. Carapace, chelicerae, and legs very dusky red 10R 2.5/2. Abdomen black 5YR 2.5/1, spinnerets dark yellowish brown 10YR 4/6. *Cephalothorax*. Carapace 8.93 long, 9.32 wide. Pars cephalica 6.41 long. Foveal groove procurved, 0.72 long, 2.1 wide. Eye tubercle low, under ME. AER procurved. PER straight to slightly recurved. Eye group 1.24 long, 2.08 wide, AME 0.43, PME 0.36, ALE 0.7, PLE 0.51. Sternum sparsely setose around outer edges, STRl 5.7, STRw 5.92. Chelicerae with anterior row comprising six teeth, posterior margin with seven teeth. Palpal endites with 31 cuspules spread across proximal half of endite and 33 smaller cuspules distally, ENDw 2.45, ENDl 3.96. Labium with nine cuspules, LBw 2.3, LBl 1.61. Rastellum with many strong spines on process continuing up cheliceral face. *Abdomen*. Evenly setose with pale speckles, darker along dorsal midline. *Legs*. F1 5.6; F1w 2.07; P1 3.6; Ti1 3.54; Mt1 2.71; Tr1 1.56; F3 4.78; F3w 2.6; P3 3.17; Ti3 2.79, Sd3 2.15; Mt3 2.32; Tr3 2.07; F4 5.97; F4w 2.43; P4 3.46; Ti4 3.85; Mt4 3.65; Tr4 1.99. Retrolateral face tarsus IV with defined tight comb of long spinules. *Pedipalps*. PF 5.31, PP 3.33, PTi 3.45, PTr 3.07. Spermathecae curved medially with large medial facing bulb.

##### Variation, females

**(n = 23).**CL 4.58–12.14, 7.75±0.37; CW 4.28–10.98, 7.61±0.38; Cap 2.76–8.64, 5.26±0.29; ENDl 0.19–1.24, 0.92±0.05; ENDw 1.04–2.36, 1.8±0.08; STRl 2.71–6.94, 4.74±0.22; STRw 2.67–7.07, 4.58±0.24; LBl 0.73–2.05, 1.33±0.07; LBw 0.97–2.53, 1.77±0.08; F1 2.75–6.8, 4.86±0.21; F1w 1.09–2.83, 1.82±0.09; P1 1.7–4.56, 3.11±0.15; Ti1 1.58–4.48, 3.03±0.15; Mt1 1.09–2.98, 2.15±0.11; Tr1 0.74–1.69, 1.22±0.05; F3 2.56–6.07, 4.21±0.19; F3w 1.34–3.6, 2.25±0.11; P3 1.42–4.16, 2.66±0.14; Ti3 1.21–3.54, 2.35±0.12; Mt3 1.07–2.65, 1.94±0.09; Tr3 1.13–2.56, 1.78±0.08; F4 3.04–7.79, 5.24±0.24; F4w 1.4–3.48, 2.21±0.11; P4 1.61–4.66, 2.95±0.16; Ti4 1.88–4.67, 3.18±0.15; Mt4 1.72–4, 3.04±0.14; Tr4 1.02–2.32, 1.64±0.07; PF 2.48–6.31, 4.38±0.2; PP 1.01–3.87, 2.61±0.14; PTi 1.46–4.01, 2.78±0.14; PTr 1.44–3.36, 2.61±0.12.

##### Material examined.

**United States: Alabama: Calhoun Co**: 7 mi N Jacksonville, on Nisbet Lake Rd., 33.8795 -85.7889^4^, 233 m a.s.l. (UMM0367, 29.viii.1979, 1♂, A Boozer, AMNH); Jacksonville, 33.8139 -85.7612^6^, 209 m a.s.l. (UMM0363, 30.ix.1979, 1♂, KE Landers, AMNH); **Cleburne Co**: 33.6273 -85.5277^7^, 307 m a.s.l. (UMM0388, 18.xi.1979, 1♀, C Benton, AMNH); **Covington Co**: Solon Dixon, 31.1426 -86.6962^5^, 88 m a.s.l. (AUMS016199, 1♂, 20.vi.2015, C Sanspree, BME); **Jackson Co**: Skyline Wilderness Area, Bishop Cove, 34.9073 -86.1289^1^, 325 m a.s.l. (AUMS016344, 10.x.2015, 1♀, KF Bourguignon, RL Godwin, BME); **Lee Co**: Auburn, 32.5976 -85.479^5^, 218 m a.s.l. (UMM0733, 24.ix.1979, 1♂, ML Williams, BME); Auburn, 32.6106 -85.4803^6^, 212 m a.s.l. (UMM0442, 1♀, N Banks, AMNH); **Tuscaloosa Co**: 33.2094 -87.5694^7^, 60 m a.s.l. (UMM0307, 11.vi.1974, 1♀, C Benton, AMNH) ; **Florida: Alachua Co**: Gainesville, 29.6673 -82.3507^6^, 49 m a.s.l. (UMM0659, 10.ix.2012, 1♂, L Buss, UFMNH); Gainesville, 29.6516 -82.3248^6^, 54 m a.s.l. (UMM0183, 18.x.1980, 1♂, GB Edwards, RE Williams, UFMNH); Gainesville, DPI, 29.6342 -82.3722^4^, 24 m a.s.l. (UMM0197, 18.x.1983, 1♂, J Wiley, UFMNH); (UMM0198, 24.x.1983, 1♂, SW Dunkle, UFMNH); Gainesville, San Felasco Hammock, off Hwy 232, Sandhill Community, 29.714 -82.4529^5^, 50 m a.s.l. (UMM0190, 25.xi.1986, 1♂, DT Corey, UFMNH); Gainesville, western Gainesville, 29.6735 -82.3845^6^, 49 m a.s.l. (UMM0655, 16–17.xi.2001, 1♂, R Wallbrunn, UFMNH); NW Gainesville, end of Millhopper Rd, 29.7175 -82.4824^4^, 41 m a.s.l. (UMM0649, iii.1993, 1♀, J Knott, UFMNH); 29.6573 -82.311^7^, 51 m a.s.l. (UMM0283, 12.i.1938, 1♀, BJ Kaston, AMNH); **Citrus Co**: 28.8848 -82.5187^7^, 16 m a.s.l. (UMM0423, 26.ii.1937, 1♀, HKN, AMNH); **Columbia Co**: High Springs, O’leno State Park, off Hwy 441, 29.9165 -82.5819^4^, 17 m a.s.l. (UMM0408, 22.i.1987, 1juv, D Corey, AMNH); **Franklin Co**: Dog Island, 29.797 -84.6117^5^, 2 m a.s.l. (UMM0280, 4.vi.1984, 1♂, L Alexander, AMNH); **Highlands Co**: Archbold Biological Station, Lake Placid, 27.2007 -81.3553^3^, 38 m a.s.l. (UMM0195, 24.ix.1984, 1♂, M Deyrup, UFMNH); (UMM0656, 27.ix.1984, 1♂, UFMNH); **Hillsborough Co**: 7.9 air miles W Plant City, 28.0325 -82.3232^6^, 17 m a.s.l. (AUMS024771, 8.x.1991, 1♂, JC Godwin, BME); **Indian River Co**: Sebastian, 27.8163 -80.4706^6^, 7 m a.s.l. (UMM0216, 6.iv.1909, 1♀, G Nelson, MCZ); **Levy Co**: Cedar Key Scrub State Park, 29.2095 -82.9868^1^, 20 m a.s.l. (AUMS016397, 20.xi.2015, 1♀, RL Godwin, BME); (AUMS016396, 1juv, BME); Sea Horse Key, 29.0979 -83.0649^4^, 0 m a.s.l. (UMM0411, 30.x.1955, 1♂, C Wharton, AMNH); **Liberty Co**: nr. Bristol, Apalachicola Bluffs Ravines Preserve, Bluff’s Tract, 30.4532 -84.9713^4^, 52 m a.s.l. (UMM0658, 10.x.2002, 1♂, C Porter, L Stange, UFMNH); Torreya State Park, 30.5697 -84.9476^5^, 77 m a.s.l. (UMM0398, 7.ix.1967, 1♂, FJ Moore, AMNH); **Marion Co**: Belleview, 29.0643 -82.0593^6^, 23 m a.s.l. (UMM0399, 22.ii.1989, 1♀, JS Kutis, AMNH); Ocala National Forest, Riverside Island, 29.4782 -81.8206^1^, 17 m a.s.l. (UMM0653, 19.v.1975, 1♂, CR Smith, UFMNH); **Orange Co**: Orlando, 28.538 -81.3785^6^, 31 m a.s.l. (UMM0193, 15.xi.1987, 1♀, K Chubb, UFMNH); Orlando, 4200 block of Waternill Ave off Univ Blvd, 28.6014 -81.2582^4^, 17 m a.s.l. (UMM0785, 21.ii.1999, 1♀, A Thaxton, AMNH); Wekiwa State Park, Sandhill community, 28.7127 -81.4627^6^, 16 m a.s.l. (UMM0180, 6.xi.1987, 1♂, DT Corey, UFMNH); (UMM0192, 1♂, UFMNH); (UMM0188, 7.xi.1987, 1♂, UFMNH); **Pasco Co**: Dade City, 28.3614 -82.1961^5^, 39 m a.s.l. (UMM0650, 16.v.1984, 1♀, T Weeks, AMNH); **Putnam Co**: Cowpen Lake, 29.604 -82.0011^4^, 27 m a.s.l. (UMM0281, ix.1946, 1♀, FN Young, AMNH); Interlachen, off HWY 315, Sandhill Community, 29.6238 -81.8903^6^, 31 m a.s.l. (UMM0187, 10.vi.1988, 1♂, D Corey, UFMNH); Mannville, E. of Interlachen, 29.6284 -81.8679^5^, 28 m a.s.l. (UMM0651, 13.v.1999, 1♀, J Litchfield, AMNH); **Santa Rosa Co**: E of Escambia River on FL-4, 30.9562 -87.2146^1^, 16 m a.s.l. (MY02549, 27.ii.2004, 1juv, BE Hendrixson, A Beamer, BME); (MY02550, 1♀, BME); Milton, 30.6321 -87.0529^4^, 27 m a.s.l. (UMM0652, 13.xii.2011, 1♂, M Donahoe, UFMNH); Milton, Trail Lane, 30.647 -86.844^3^, 40 m a.s.l. (UMM0660, 30.xi.2011, 1♂, E Nelson, UFMNH); **Volusia Co**: Benson Springs, 28.8658 -81.2544^5^, 17 m a.s.l. (UMM0777, 23.ix.1933, 1juv, HW, AMNH); **Escambia Co**: Hwy 488, 30.642 -87.3375^7^, 35 m a.s.l. (UMM0407, 15.vii.1935, 1juv, Canrall, AMNH); **Georgia: Glynn Co**: Jekyll Island, 31.0702 -81.4199^5^, 1 m a.s.l. (UMM0456, 22.x.1970, 1♂, C Benton, AMNH); St. Simons Island, 31.161 -81.3875^5^, 3 m a.s.l. (UMM0227, 10.x.1984, 1♂, GJ Magnon, AMNH); **Liberty Co**: Catherine’s Island, 31.703 -81.275^6^, 0 m a.s.l. (UMM0534, 20.iv.1973, 1♀, A Zweifel, AMNH); **Long Co**: 8.2km SW Ludowici, Griffin Ridge WMA, 31.6796 -81.8216^1^, 19 m a.s.l. (UMM0700, 1.iii.2018, 1♀, D Stevenson, BME); **Marion Co**: 13.4 airmiles NNW of Buena Vista, 32.4722 -84.6151^6^, 162 m a.s.l. (UMM0735, 19.xi.1979, 1♂, W Seyle, BME); **Quitmen Co**: 31.7902 -85.0916^1^, 104 m a.s.l. (AUMS004564, 24.iii.2013, 1♀, CA Hamilton, BME); 31.7902 -85.0916^1^, 104 m a.s.l. (AUMS004565, 24.iii.2013, 1♀, CA Hamilton, BME); **Screven Co**: 9 mi S Sylvania, 32.6197 -81.6358^5^, 59 m a.s.l. (UMM0229, 4.x.1984, 1♂, JB Cole, AMNH); **Louisiana: Grant Co**: Kisatchie National Forest, 31.3555 -92.4344^5^, 45 m a.s.l. (UMM0454, vi.1941, 1♀, Jones, Archer, AMNH); **Mississippi: George Co**: Lucedale, 30.925 -88.59^6^, 91 m a.s.l. (UMM0531, 1.ix.1931, 1♀, Dietrich, AMNH); **North Carolina: Brunswick Co**: Boiling Springs Lakes, Cedar Rd, 34.0507 -78.0423^4^, 16 m a.s.l. (UMM0112, 3.x.2006, 1♂, CL Hasvold, NCSM); **Carteret Co**: Beaufort, 34.7182 -76.6638^5^, 2 m a.s.l. (UMM0561, 17.ix.1951, 1♂, RD Barnes, AMNH); **Iredell Co**: Weyerhauser, Cool Springs, 35.1825 -77.0754^1^, 3 m a.s.l. (MY0002311, 1♂, JE Bond, BME); (MY0002312, 1♂, BME); (MY0002313, 1♂, BME); **Moore Co**: Manley, 35.3667 -79.4693^7^, 98 m a.s.l. (UMM0633, x.1955, 1♀, A Twombly, AMNH); **New Hanover Co**: Wilmington, 34.1855 -77.894^6^, 40 m a.s.l. (AUMS004566, 11.x.2012, 1♂, JE Bond, BME); (AUMS004567, 1♂, BME); (AUMS004568, 1♂, BME); (AUMS004569, 1♂, BME); (AUMS004570, 1♂, BME); (AUMS004571, 1♂, BME); (AUMS004572, 1♂, BME); (AUMS004573, 1♂, BME); **Virginia: Isle of Wight Co**: Pine Barrens, 3.5 mi SSW of Zuni, 36.8185 -76.8541^5^, 11 m a.s.l. (UMM0042, i.1985, 1♂, CA Pague, VMNH); **Virginia Beach Co**: Virginia Beach City, Seashore State Park, 36.9185 -76.0523^5^, 0 m a.s.l. (UMM0064, 13.x.1989, 1♂, KA Buhlmann, VMNH); (UMM0010, 29.ix.1989, 1♂, VMNH).

#### Ummidia okefenokee sp. nov.

Taxon classificationAnimaliaAraneaeHalonoproctidae

08DFAEB3-19EE-5642-8254-F2EAF3BE058A

http://zoobank.org/59BE8AFD-DB43-4FAD-98CB-6299176274D6

Fig. 14 , Map 3

##### Type material.

HOLOTYPE: 1 ♂ (UMM163) from Billy’s Island, Okefenokee Swamp, Charlton County, Georgia, United States, 30.8056 -82.3371^5^, 36 m a.s.l., 17.v.1921, AMNH.

##### Etymology.

The specific epithet is a noun taken in apposition and refers to the type locality.

##### Diagnosis.

*Ummidia okefenokee* can be differentiated from *U. audouini* by the presence of a comb on the retrolateral face of tarsus IV, which is more defined than the comb in *U. neilgaimani*. Males can further be distinguished from *U. gingoteague* and *U. carabivora* by lacking spines on the prolateral aspect of tibia I and from *U. rongodwini* by lacking a pale dorsal heart patch. Males disperse from May to June.

##### Description of male holotype.

*Specimen preparation and condition*. Specimen preserved in 80% EtOH. Specimen has been dried at some point; many leg articles broken, in vial with specimen. *General coloration*. Carapace and chelicerae very dusky red 10R 2.5/2, legs dark brown 7.5YR 3/4. Abdomen black 10YR 2/1. *Cephalothorax*. Carapace 5.26 long, 4.86 wide. Pars cephalica 3.51 long. Foveal groove procurved with posterior pointed extension, 0.18 long, 0.88 wide. All eyes on moderate tubercle. AER procurved. PER straight. Eye group 0.63 long, 1.2 wide, AME 0.24, PME 0.17, ALE 0.32, PLE 0.25. Sternum with outer fringe of setae, STRl 2.83, STRw 2.81. Chelicerae with anterior tooth row comprising three teeth, posterior margin with five teeth. Palpal endites with mix of 28 hastate and small/thin cuspules over proximal half of endite face and lacking distal endite cuspules, ENDw 1.18, ENDl 2.07. Labium with five cuspules, LBw 1.11, LBl 0.71. Rastellum reduced with only small denticles on small process. Abdomen setose with pale speckles. *Legs*. F1 4.19; F1w 1.22; P1 2.16; Ti1 2.84; Mt1 1.82; Tr1 1.09; F3 3.25; F3w 1.28; P3 1.74; Ti3 1.69; Sd3 1; Mt3 1.74; Tr3 1.61; F4 4.07; F4w 1.25; P4 1.94; Ti4 2.64; Mt4 2.62; Tr4 1.57. Retrolateral face of tarsus IV with defined comb over length of tarsus. Leg I spination pattern: TSp 0, TSpv 0, TSrd 0, TSr 0, TSrv 19, MtSp 3, MtSr 8, TrSp 3, TrSr 9. *Pedipalps*. PTl 2.38, PTw 0.89, Bl 1.84. Embolus evenly curved.

**Figure 14.**
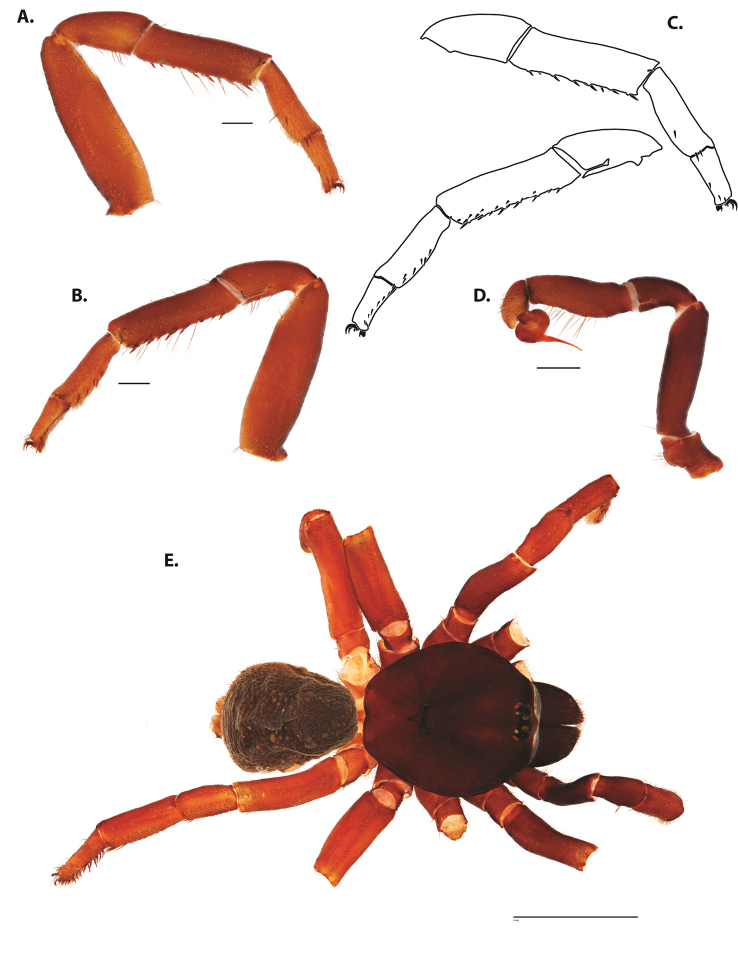
*Ummidia okefenokee* sp. nov. male holotype specimen (UMM163) from Okefenokee Swamp, Charlton Co., Georgia. **A** retrolateral aspect, leg I **B** prolateral aspect, leg I **C** line drawings, leg I retrolateral and prolateral aspects **D** retrolateral aspect, pedipalp **E** habitus illustration. Scale bars: 1.0 mm (**A–D**), 4.0 mm (**E**).

##### Variation, males

**(n = 2).**CL 5.26–5.66, 5.46±0.2; CW 4.86–5.55, 5.21±0.34; Cap 3.51–4.11, 3.81±0.3; ENDl 0.63–0.7, 0.67±0.04; ENDw 1.2–1.33, 1.27±0.07; STRl 2.83–3.18, 3.01±0.17; STRw 2.81–3.05, 2.93±0.12; LBl 0.71–0.75, 0.73±0.02; LBw 1.11–1.11, 1.11±0; F1 4.19–4.72, 4.46±0.27; F1w 1.22–1.32, 1.27±0.05; P1 2.16–2.36, 2.26±0.1; Ti1 2.84–3.15, 2.99±0.16; Mt1 1.82–1.97, 1.9±0.07; Tr1 1.09–1.12, 1.1±0.02; F3 3.25–3.63, 3.44±0.19; F3w 1.28–1.5, 1.39±0.11; P3 1.74–1.96, 1.85±0.11; Ti3 1.69–1.99, 1.84±0.15; Mt3 1.74–1.8, 1.77±0.03; Tr3 1.61–1.74, 1.68±0.06; F4 4.07–4.55, 4.31±0.24; F4w 1.25–1.48, 1.36±0.12; P4 1.94–1.97, 1.96±0.01; Ti4 2.64–2.98, 2.81±0.17; Mt4 2.62–2.81, 2.71±0.1; Tr4 1.47–1.57, 1.52±0.05; TSp 0–3, 1.5±1.5; TSpv 0–0, 0±0; TSr 0–0, 0±0; TSrv 15–19, 17±2; PTl 2.38–2.72, 2.55±0.17; PTw 0.89–0.97, 0.93±0.04; BL 1.72–1.84, 1.78±0.06.

##### Females.

Unknown.

##### Material examined.

**United States: Florida: Alachua Co**: 36 mi W Melrose, 29.7135 -82.1526^7^, 42 m a.s.l. (UMM0532, 13.vi.1962, 1♂, JA Beatty, AMNH); **Georgia: Charlton Co**: Billy’s Island, Okefenokee Swamp, 30.8056 -82.3371^5^, 36 m a.s.l. (UMM0163, 17.v.1921, 1♂, AMNH).

#### Ummidia richmond sp. nov.

Taxon classificationAnimaliaAraneaeHalonoproctidae

4B8C2ACF-1CDF-5A65-950A-2C1B83C9FD35

http://zoobank.org/3792FE9A-475D-4EEE-9DE8-F27CA03F326D

Fig. 15 , Map 3

##### Type material.

HOLOTYPE: 1 ♂ (UMM159) from 6 mi SSW of Perrine, Miami-Dade County, Florida, United States, 25.605 -80.3566^6^, 4 m a.s.l., 16.v.1958, coll. HF Strohecker, AMNH.

##### Etymology.

The specific epithet is a noun taken in apposition and refers to Richmond, originally a small sawmill camp, then the Richmond Naval Air Base, and now known as the Richmond Tract. It contains the most diverse and second largest remaining fragment of critically endangered pine rockland habitat (the largest is in Everglades National Park but is threatened by rising sea levels and will likely lose the title by the end of the century), the habitat in which the spiders were found.

##### Diagnosis.

*Ummidia richmond* can be differentiated from *U. audouini* by the presence of a comb on the retrolateral face of tarsus IV which is more defined than the comb in *U. neilgaimani*. Males can further be distinguished from *U. gingoteague* and *U. carabivora* by lacking spines on the prolateral aspect of tibia I and from *U. rongodwini* by lacking a pale dorsal heart patch. It can be differentiated from *U. rongodwini* by having eyes relatively smaller and more removed from the anterior margin of the carapace. Males disperse from May to June.

**Figure 15.**
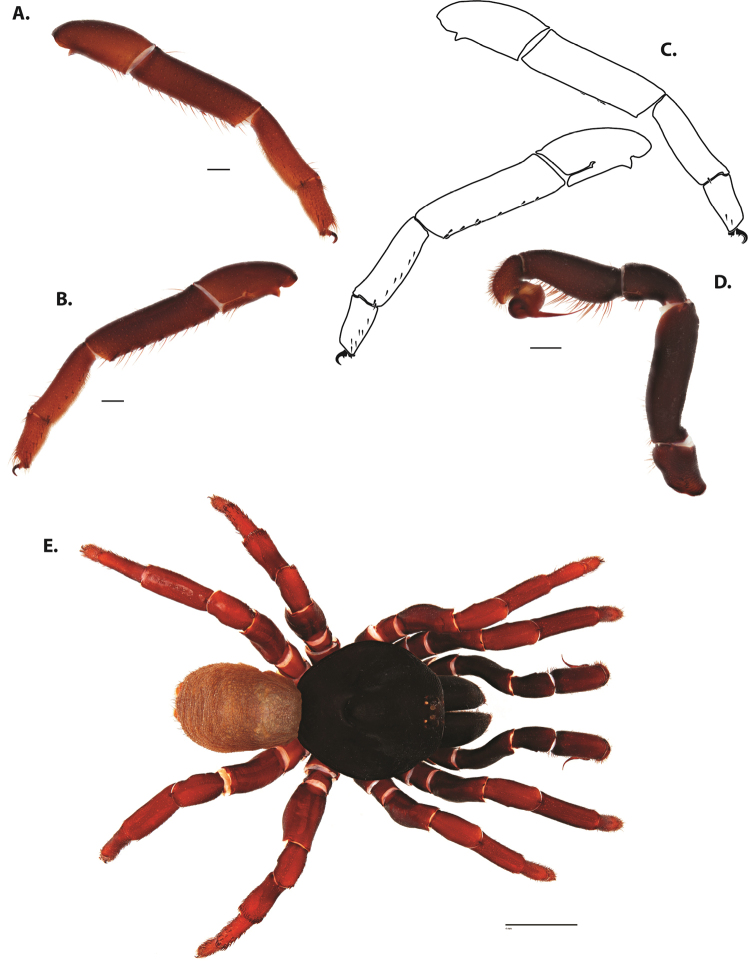
*Ummidia richmond* sp. nov. male holotype (UMM159) from Perrine, Florida **A** retrolateral aspect, leg I **B** prolateral aspect, leg I **C** line drawings, leg I retrolateral and prolateral aspects **D** retrolateral aspect, pedipalp **E** habitus illustration. Scale bars: 1.0 mm (**A–D**), 4.0 mm (**E**).

##### Description of male holotype.

*Specimen preparation and condition*. Specimen preserved in 80% EtOH. Left palp, leg I removed, in vial with specimen. *General coloration*. Carapace and chelicerae reddish black 2.5YR 2.5/1, legs dark reddish brown 5YR 3/4. Abdomen dark brown 7.5YR 3/2. *Cephalothorax*. Carapace 7.9 long, 7.63 wide. Pars cephalica 5.32 long. Foveal groove procurved, 0.43 long, 1.36 wide. All eyes on defined moderate tubercle. AER procurved. PER slightly procurved. Eye group 0.87 long, 1.82 wide, AME 0.38, PME 0.2, ALE 0.4, PLE 0.21. Sternum sparsely setose anteriorly with posterior fringe, STRl 4.35, STRw 4.07. Chelicerae with anterior tooth row comprising five teeth, posterior margin with five teeth. Palpal endites with 21 small, non-hastate cuspules over proximal half of endite face, and seven small non-hastate cuspules distally, ENDw 1.73, ENDl 3.0. Labium with six small, non-hastate cuspules, LBw 1.44, LBl 1.16. Rastellum with many small teeth on process. Abdomen setose with dorsal opalescence. *Legs*. F1 5.58; F1w 1.69; P1 3.11; Ti1 4.02; Mt1 2.66; Tr1 1.45; F3 4.59; F3w 2.07; P3 2.56; Ti3 2.66; Sd3 1.4; Mt3 2.6; Tr3 2.11; F4 5.69; F4w 2.07; P4 2.78; Ti4 3.84; Mt4 3.7; Tr4 1.94. Retrolateral face of tarsus IV with defined comb over length of tarsus. Leg I spination pattern: TSp 0, TSpv 0, TSrd 0, TSr 0, TSrv 8, MtSp 1, MtSr 7, TrSp 2, TrSr 6. *Pedipalps*. PTl 3.14, PTw 1.33, Bl 2.69. Embolus evenly curved.

##### Variation, males

**(n = 8).**CL 6.15–8.36, 7.29±0.28; CW 5.87–8.39, 7.19±0.33; Cap 4.22–6.16, 5.16±0.25; ENDl 0.66–0.96, 0.81±0.04; ENDw 1.42–1.86, 1.68±0.06; STRl 3.16–4.58, 3.96±0.2; STRw 3.01–4.35, 3.75±0.19; LBl 0.87–1.29, 1.12±0.06; LBw 1.19–1.87, 1.51±0.08; F1 4.65–6.39, 5.46±0.22; F1w 1.42–2.69, 1.75±0.14; P1 2.42–3.26, 2.97±0.12; Ti1 3.28–4.35, 3.78±0.14; Mt1 2.11–2.97, 2.6±0.12; Tr1 1.12–1.51, 1.34±0.05; F3 3.48–4.92, 4.3±0.2; F3w 1.5–2.17, 1.86±0.09; P3 2.08–2.7, 2.46±0.09; Ti3 2.09–2.9, 2.51±0.11; Mt3 1.94–2.84, 2.46±0.12; Tr3 1.5–2.2, 1.87±0.1; F4 4.37–6.29, 5.35±0.26; F4w 1.44–2.07, 1.78±0.09; P4 2.23–3.12, 2.69±0.11; Ti4 3.07–4.27, 3.64±0.15; Mt4 2.82–4.04, 3.52±0.17; Tr4 1.56–2.19, 1.86±0.08; TSp 0–5, 0.88±0.64; TSpv 0–4, 0.5±0.5; TSr 0–0, 0±0; TSrv 2–17, 8.38±1.95; PTl 2.56–3.74, 3.11±0.14; PTw 1.13–1.42, 1.28±0.04; BL 2.27–2.9, 2.62±0.08.

##### Females.

Unknown.

##### Material examined.

**United States: Florida: Collier Co**: Fort Myers, 26.6405 -81.8717^6^, 3 m a.s.l. (UMM0160, 3.vi.1958, 1♂, N Causey, AMNH); **Miami-Dade Co**: Everglades National Park, Long Pine Key, 25.4088 -80.6861^5^, 1 m a.s.l. (UMM0288, 28.v-8.vi.1986, 1♂, S Peck, J Peck, AMNH); (UMM0429, 8.vi.1926, 1♂, AMNH); Perrine, 25.605 -80.3566^6^, 4 m a.s.l. (UMM0159, 16.v.1958, 1♂, HF Strohecker, AMNH); Zoo Miami, 25.602 -80.3983^1^, 2 m a.s.l. (AUMS016784, 18.v.2016, 1♂, F Ridgely, BME); (AUMS016785, 1♂, BME); **Monroe Co**: Big Pine Key, Key Deer Refuge, Palm Avenue, 24.699 -81.3761^4^, 1 m a.s.l. (UMM0654, 1♂, AMNH).

#### Ummidia macarthuri sp. nov.

Taxon classificationAnimaliaAraneaeHalonoproctidae

2B704AA1-C045-5281-9CA3-271050C13564

http://zoobank.org/B99B1CA8-5BCB-4F70-8757-7B8FEDFE5241

**Figure 16.**
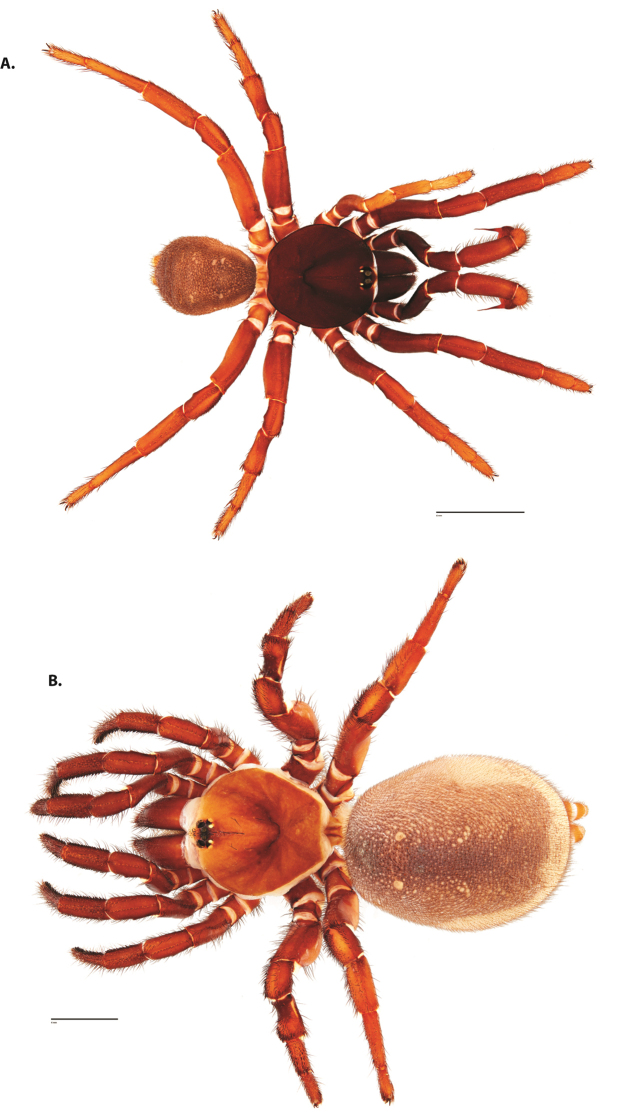
*Ummidia macarthuri* sp. nov. from Washington Co. Arkansas **A** male habitus illustration UMM286 **B** female habitus illustration UMM647. Scale bars: 4.0 mm.

**Figure 17.**
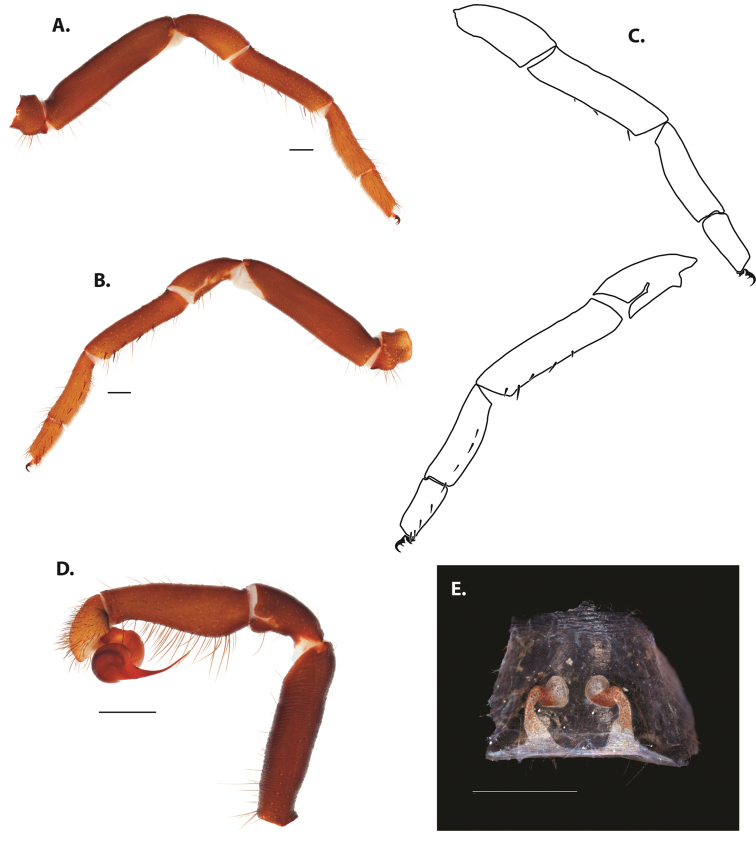
*Ummidia macarthuri* sp. nov. from Washington Co. Arkansas **A–D** male holotype (UMM286) **A** prolateral aspect, leg I **B** retrolateral aspect, leg I **C** line drawings, leg I prolateral and retrolateral aspects **D** retrolateral aspect, pedipalp **E** cleared spermathecae female exemplar (UMM647). Scale bars: 1.0 mm.

Figs 16 , 17 , Map 3

##### Type material.

HOLOTYPE: 1 ♂ (UMM286) from Cove Creek, Washington County Arkansas, United States, 35.8167 -94.395^6^, 583 m a.s.l., 13.x.1962, coll. O Hite, M Hite, AMNH.

##### Etymology.

The specific epithet is a patronym named in honor of Arkansas native General Douglas MacArthur.

##### Diagnosis.

*Ummidia macarthuri* males can be differentiated from *U. rongodwini* by lacking a pale dorsal heart patch and from *U. audouini* and *U. rosillos* by lacking a brush on the retrolateral face of tarsus IV. Comb on tarsus IV is less distinct than that of *U. funerea* and *U. colemanae*. Males can be differentiated from all geographically proximate species other than *U. rongodwini* by the lack of prolateral spines on tibia I. Males disperse from September to October.

##### Description of male holotype.

*Specimen preparation and condition*. Specimen preserved in 80% EtOH. Left palp, leg I removed, in vial with specimen. *General coloration*. Carapace and chelicerae dark reddish brown 2.5YR 2.5/2, legs yellowish red 5YR 4/6. Abdomen very dark brown 7.5YR 2.5/3. *Cephalothorax*. Carapace 4.91 long, 4.88 wide. Pars cephalica 3.24 long. Foveal groove procurved, 0.38 long, 0.92 wide. All eyes on moderate tubercle. AER procurved. PER straight. Eye group 0.64 long, 1.14 wide, AME 0.32, PME 0.17, ALE 0.38, PLE 0.22. Sternum sparsely setose around outer 1/3, thicker anteriorly, STRl 2.77, STRw 2.77. Chelicerae with anterior tooth row comprising five teeth, posterior margin with five teeth. Palpal endites with 18 small hastate cuspules spread across proximal half of endite face, lacking distal endite cuspules, ENDw 1.3, ENDl 2.01. Labium with six cuspules, LBw 1.01, LBl 0.71. Rastellum with many spines on process. Abdomen setose with pale speckles. *Legs*. F1 4.36; F1w 1.05; P1 2.13; Ti1 3.04; Mt1 2.05; Tr1 1.14; F3 3.12; F3w 1.32; P3 1.75; Ti3 2.01; Sd3 1.35; Mt3 1.8; Tr3 1.44; F4 4.31; F4w 1.28; P4 1.97; Ti4 2.66; Mt4 3; Tr4 1.79. Retrolateral face of tarsus IV with indistinct comb surrounded in setae. Leg I spination pattern: TSp 0, TSpv 0, TSrd 0, TSr 0, TSrv 5, MtSp 0, MtSr 4, TrSp 0, TrSr 4. *Pedipalps*. PTl 2.5, PTw 0.96, Bl 1.9. Embolus evenly curved.

##### Variation, males

**(n = 9).**CL 4.58–5.92, 5.21±0.18; CW 4.67–5.94, 5.27±0.17; Cap 3.09–3.96, 3.55±0.12; ENDl 0.61–0.82, 0.73±0.02;ENDw1.14–1.42, 1.27±0.03; STRl 2.64–3.36, 3±0.09; STRw 2.54–3.31, 2.9±0.09; LBl 0.68–0.96, 0.82±0.04; LBw 0.88–1.25, 1.06±0.05; F1 4.31–5.39, 4.82±0.15; F1w 1.05–1.4, 1.2±0.04; P1 2.02–2.6, 2.32±0.07; Ti1 2.73–3.61, 3.13±0.11; Mt1 1.73–2.44, 2.1±0.08; Tr1 1.06–1.28, 1.17±0.02; F3 3.12–4.05, 3.55±0.13; F3w 1.2–1.61, 1.4±0.05; P3 1.57–2.08, 1.83±0.06; Ti3 1.84–2.48, 2.09±0.08; Mt3 1.66–2.18, 1.92±0.06; Tr3 1.28–1.74, 1.59±0.05; F4 4.3–5.36, 4.81±0.16; F4w 1.16–1.56, 1.32±0.05; P4 1.9–2.34, 2.1±0.06; Ti4 2.66–3.39, 2.99±0.11; Mt4 2.85–3.67, 3.21±0.12; Tr4 1.59–2.14, 1.86±0.06; TSp 0–1, 0.11±0.11; TSpv 0–0, 0±0; TSr 0–1, 0.11±0.11; TSrv 4–8, 6±0.47; PTl 2.48–2.95, 2.68±0.07; PTw 0.93–1.13, 1.01±0.02; BL 1.87–2.21, 2.04±0.05.

##### Description of female exemplar.

*Specimen preparation and condition*. Specimen preserved in 80% EtOH. Spermathecae removed, cleared, in vial with specimen. *General coloration*. Carapace and posterior legs dark reddish brown 5YR 3/4, chelicerae and anterior legs dark reddish brown 2.5YR 2.5/4. Abdomen dark brown 10YR 3/3, spinnerets dark yellowish brown 10YR 4/4. *Cephalothorax*. Carapace 7.03 long, 7.57 wide. Pars cephalica 4.85 long. Foveal groove procurved, 0.59 long, 1.56 wide. Eye tubercle low. AER procurved. PER procurved. Eye group 1.08 long, 1.79 wide, AME 0.33, PME 0.3, ALE 0.58, PLE 0.32. Sternum sparsely setose around edges, thicker anteriorly, STRl 4.34, STRw 4.63. Chelicerae with anterior row comprising six teeth, posterior margin with six teeth. Palpal endites with 23 cuspules spread across proximal half of endite and 29 cuspules distally, larger anteriorly but smaller than proximal cuspules, ENDw 1.83, ENDl 3.07. Labium with five cuspules, LBw 1.76, LBl 1.37. Rastellum with many strong spines on process and up cheliceral face. *Abdomen*. Evenly setose with pale speckles creating light chevrons at apodemes. *Legs*. F1 4.83; F1w 1.76; P1 3.16; Ti1 3.01; Mt1 2.09; Tr1 1.22; F3 4.1; F3w 2.21; P3 2.56; Ti3 2.28, Sd3 1.45; Mt3 1.98; Tr3 1.89; F4 5.24; F4w 2.17; P4 2.79; Ti4 2.93; Mt4 3.26; Tr4 1.58. Retrolateral face tarsus IV with tight comb over middle half of article. *Pedipalps*. PF 4.37, PP 2.5, PTi 2.8, PTr 2.52. Spermathecae sinuous, curving strongly medially and posteriorly then anteriorly; bulbs facing medioanteriorly.

##### Variation, females

**(n = 6).**CL 3.85–8.12, 6.57±0.62; CW 3.65–8.38, 6.53±0.66; Cap 2.68–5.59, 4.53±0.43; ENDl 0.55–1.19, 0.97±0.1;ENDw0.92–2.05, 1.63±0.16; STRl 2.08–4.77, 3.98±0.41; STRw 2.25–5.3, 4.2±0.46; LBl 0.66–1.63, 1.26±0.15; LBw 0.87–1.91, 1.54±0.16; F1 2.47–5.43, 4.51±0.44; F1w 0.94–1.88, 1.58±0.14; P1 1.53–3.34, 2.81±0.27; Ti1 1.36–3.39, 2.7±0.3; Mt1 0.98–2.41, 1.87±0.21; Tr1 0.61–1.33, 1.05±0.1; F3 2.18–4.46, 3.76±0.34; F3w 1.13–2.61, 2.04±0.21; P3 1.29–2.71, 2.32±0.22; Ti3 1.12–2.64, 2.11±0.22; Mt3 0.89–2.17, 1.74±0.19; Tr3 0.99–2.09, 1.63±0.16; F4 2.89–5.81, 4.89±0.44; F4w 1.16–2.46, 1.94±0.18; P4 1.42–3.1, 2.57±0.25; Ti4 1.46–3.2, 2.67±0.26; Mt4 1.47–3.53, 2.79±0.31; Tr4 0.85–1.81, 1.44±0.15; PF 2.19–4.79, 4.01±0.39; PP 1.24–2.7, 2.31±0.22; PTi 1.21–3.07, 2.47±0.28; PTr 1.29–2.85, 2.32±0.23.

##### Material examined.

**United States: Arkansas: Washington Co**: Cove Creek, 35.8167 -94.395^6^, 583 m a.s.l. (UMM0259, 13.x.1962, 1♂, O Hite, M Hite, AMNH); (UMM0318, 3.xi.1962, 1♂, Hite, AMNH); (UMM0140, 3.xi.1962, 1♂, Exline, Peck, AMNH); Cove Creek, 35.8167 -94.395^6^, 583 m a.s.l. (UMM0286, 13.x.1962, 1♂, AMNH); **Illinois: Jackson Co**: Carbondale, Union Hills, 37.6886 -89.2478^5^, 145 m a.s.l. (UMM0269, 7.x.1969, 1♂, JKV Adams, AMNH); Little Grand Canyon, ca. 5.8 mi SW of Murphysboro, 37.6803 -89.3933^5^, 209 m a.s.l. (UMM0264, 17.x.1970, 1♂, JA Beatty, AMNH); **Indiana: Morgan Co**: Morgan, Monroe State Forest, 39.3335 -86.4283^1^, 275 m a.s.l. (UMM0739, 18.ix.2016, 1♂, M Milne, BME); **Kentucky: Edmonson Co**: Mammoth Cave National Park, 37.1852 -86.1001^6^, 222 m a.s.l. (UMM0268, 16.ix.1966, 1♀, F Coyle, AMNH); **Mississippi: Grenada Co**: Grenada Ravine, 33.8966 -90.0294^1^, 77 m a.s.l. (UMM0703, 23.x.1991, 1♂, GL Snodgrass, MEM); 33.6963 -90.03^1^, 77 m a.s.l. (UMM0705, 28.viii.1991, 1♂, GT Baker, MEM); (UMM0704, 4.ix.1991, 1♂, M MacGown, J MacGown, MEM); **Marshall Co**: 3 mi S of Waterford, 35.5844 -89.4738^5^, 96 m a.s.l. (UMM0722, 1.vi.1994, 1♀, PR Miller, MEM); **Tate Co**: Senatobia, NWCC, 34.6254 -89.9695^5^, 91 m a.s.l. (UMM0725, 2.x.2006, 1♀, D Jones, MEM); **Tennessee: Chester Co**: Chickasaw State Park, 35.3606 -88.8618^5^, 173 m a.s.l. (UMM0647, 9.ix.1966, 1♀, Coyle, AMNH); **Davidson Co**: Nashville, 36.1658 -86.7844^6^, 165 m a.s.l. (UMM0621, 20.vi.1955, 1♀, AR Laskey, AMNH); **Shelby Co**: Cordova, 35.1558 -89.7774^5^, 112 m a.s.l. (UMM0472, 3.v.1956, 1♀, AMNH).

#### Ummidia beatula

Taxon classificationAnimaliaAraneaeHalonoproctidae

(Gertsch & Mulaik, 1940)

AE502721-A3AF-5FC5-B168-4F1404D0CCBF

**Figure 18.**
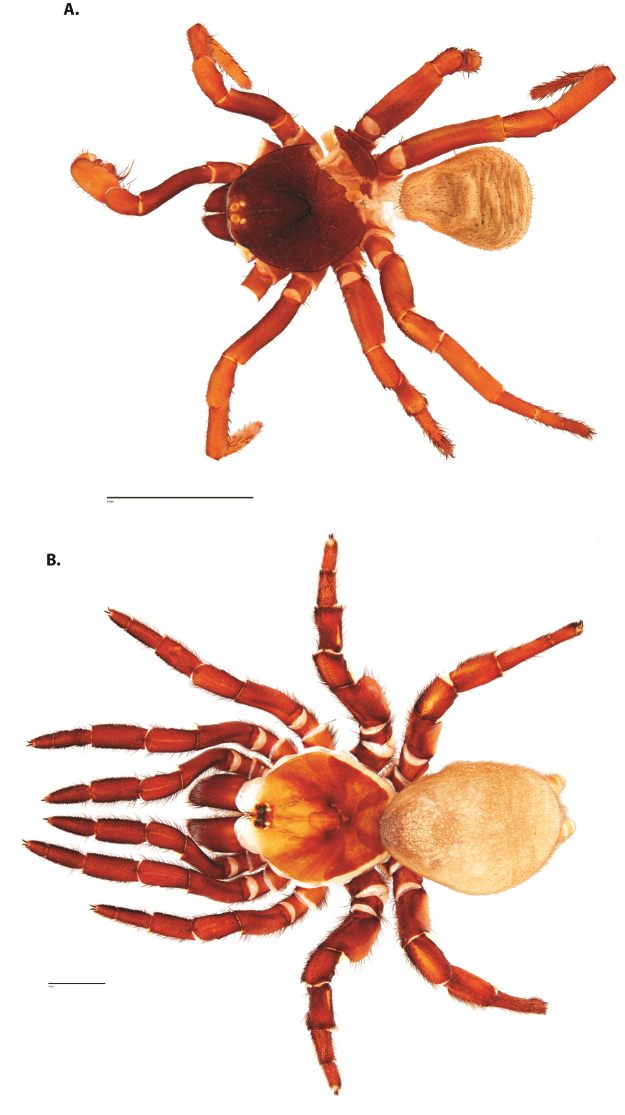
*Ummidia beatula* (Gertsch & Mulaik, 1940) from Dallas, Texas **A** male habitus illustration IZC327745 **B** female habitus illustration IZC327743. Scale bars: 4.0 mm.

**Figure 19.**
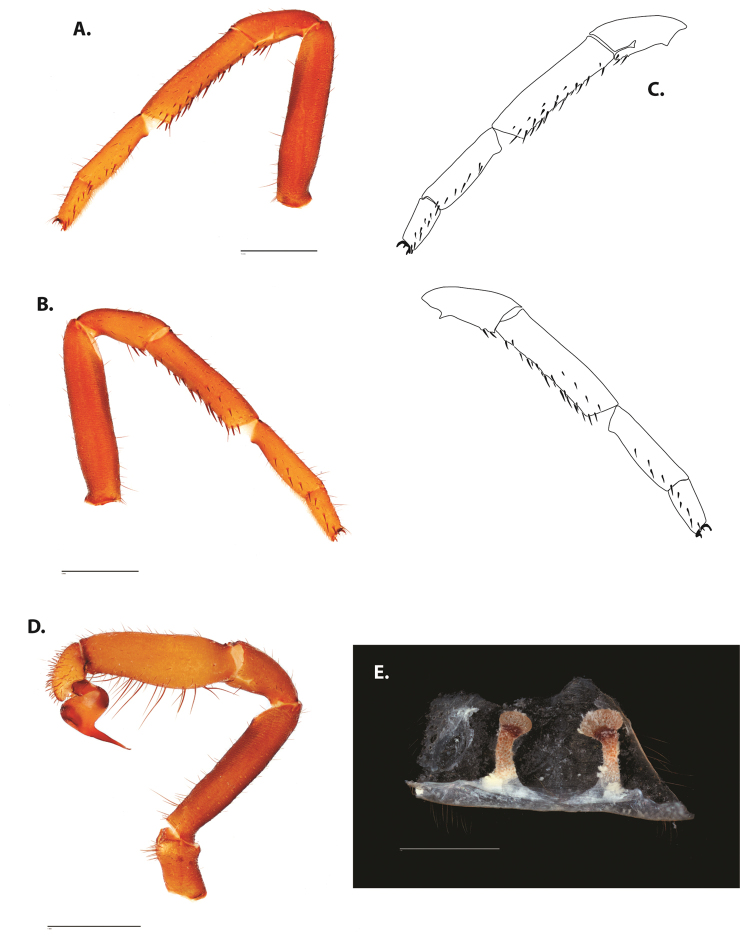
*Ummidia beatula* (Gertsch & Mulaik, 1940) from Dallas, Texas **A–D** male exemplar (IZC327745) **A** retrolateral aspect, leg I **B** prolateral aspect, leg I **C** line drawings, leg I retrolateral and prolateral aspects **D** retrolateral aspect, pedipalp **E** cleared spermathecae female holotype (IZC327743). Scale bars: 1.0 mm.

Figs 18 , 19 , Map 3

Pachylomerides beatulus Gertsch & Mulaik, 1940: 312; female HOLOTYPE (AMNH_IZC327743) from 5–6 miles S Dallas, Texas, United States, 32.6472 -96.7913^6^, 189 m a.s.l., xii.1937, coll. J.C. Sanders, deposited in AMNH, examined.
Ummidia beatula Brignoli, 1983: 117.
Pachylomerides pygmaeus Chamberlin and Ivie, 1945: 558; female HOLOTYPE from Eagletown, Oklahoma, United States, 34.0333 -94.6167^1^, 121 m a.s.l., 28.vi.1937, coll. Standish-Kaiser, deposited in AMNH, examined, **syn. nov.**
Ummidia pygmaea Brignoli, 1983: 117.

##### Diagnosis.

*Ummidia beatula* are relatively small spiders which can be differentiated from *U. rongodwini* by males lacking a pale dorsal heart patch and from *U. audouini* and *U. rosillos* by lacking a brush on the retrolateral face of tarsus IV. In males, the comb on tarsus IV is less distinct than that of *U. funerea* and *U. colemanae*. Males can be differentiated from *U. macarthuri* and *U. rongodwini* by the presence of prolateral spines on tibia I. Males disperse from April to June.

##### Description of female holotype.

*Specimen preparation and condition*. Specimen preserved in 80% EtOH. Spermathecae removed, cleared, in vial with specimen. *General coloration*. Carapace and chelicerae strong brown 7.5YR 4/6. Legs dark reddish brown 2.5YR 2.5/4. Abdomen yellowish brown 10YR 5/6, spinnerets strong brown 7.5YR 4/6. *Cephalothorax*. Carapace 9.22 long, 9.33 wide. Pars cephalica 6.3 long. Foveal groove procurved with posterior pointed extension, 0.88 long, 1.97 wide. Eye tubercle low. AER procurved. PER procurved. Eye group 2.5 long, 3.6 wide, AME 0.96, PME 0.79, ALE 1.34, PLE 0.8. Sternum sparsely setose around outer margin, thicker anteriorly, STRl 10.17, STRw 11.23. Chelicerae with anterior row comprising eight teeth, posterior margin with six teeth. Palpal endites with 24 large cuspules across proximal half of endite face and 28 smaller cuspules distally, ENDw 2.1, ENDl 3.59. Labium with seven cuspules, LBw 4.21, LBl 3.15. Rastellum with many strong spines on process. *Abdomen*. Evenly setose. *Legs*. F1 6.42; F1w 2.14; P1 3.92; Ti1 3.75; Mt1 3.74; Tr1 1.59; F3 7.07; F3w 3.7; P3 3.6; Ti3 3.4, Sd3 2.71; Mt3 2.75; Tr3 2.3; F4 7.44; F4w 2.84; P4 4.24; Ti4 4.06; Mt4 4.35; Tr4 1.97. Retrolateral face tarsus IV with defined comb over length of tarsus. *Pedipalps*. PF 5.71, PP 3.03, PTi 3.36, PTr 3.17. Spermathecae simple and straight, bulbs facing anteriorly.

##### Variation, females

**(n = 20).**CL 3.25–8.48, 6.63±0.33; CW 3.03–8.33, 6.5±0.34; Cap 2.29–5.85, 4.54±0.22; ENDl 0.47–1.19, 0.94±0.04; ENDw 0.82–2.03, 1.61±0.08; STRl 2.02–5.56, 4.18±0.22; STRw 1.32–5.61, 4.11±0.28; LBl 0.56–1.93, 1.21±0.08; LBw 0.76–2.02, 1.55±0.08; F1 2.09–5.52, 4.49±0.22; F1w 0.83–2.56, 1.65±0.09; P1 1.32–3.61, 2.84±0.15; Ti1 1.14–3.51, 2.63±0.14; Mt1 0.77–2.62, 1.9±0.1; Tr1 0.59–1.2, 0.99±0.04; F3 1.92–4.8, 3.74±0.18; F3w 0.88–2.65, 2.03±0.11; P3 1.16–3, 2.33±0.12; Ti3 0.98–2.84, 2.18±0.12; Mt3 0.85–2.19, 1.74±0.08; Tr3 0.82–1.9, 1.52±0.06; F4 2.41–5.9, 4.82±0.23; F4w 1.03–2.68, 2.04±0.1; P4 1.31–3.35, 2.64±0.14; Ti4 1.38–3.33, 2.7±0.13; Mt4 1.16–3.47, 2.75±0.15; Tr4 0.92–1.93, 1.48±0.06; PF 1.83–4.97, 4±0.2; PP 1.12–3.04, 2.31±0.12; PTi 0.33–3.08, 2.32±0.17; PTr 1.06–2.7, 2.25±0.1.

##### Description of male exemplar.

*Specimen preparation and condition*. Specimen preserved in 80% EtOH. Color faded, cephalothorax broken. Left palp, leg I, and right leg I removed, in vial with specimen. *General coloration*. Carapace and chelicerae very dusky red 10R 2.5/2, legs strong brown 7.5YR 4/6. Abdomen dark brown 10YR 3/3. *Cephalothorax*. Carapace 3.73 long, 3.6 wide. Pars cephalica 2.32 long. Foveal groove procurved, 0.26 long, 0.68 wide. Tubercle moderate. AER procurved. PER straight. Eye group 0.92 long, 0.52 wide, AME 0.26, PME 0.2, ALE 0.29, PLE 0.2. Sternum sparsely setose around outer 1/3, STRl 2.17, STRw 1.99. Chelicerae with anterior tooth row comprising five teeth, posterior margin with five teeth. Palpal endites with 14 cuspules spread over proximal half of endite face, lacking distal endite cuspules, ENDw 0.89, ENDl 1.42. Labium with six cuspules, LBw 0.71, LBl 0.43. Rastellum with many spines. Abdomen setose. *Legs*. F1 3.55; F1w 0.74; P1 1.72; Ti1 2.29; Mt1 1.68; Tr1 0.8; F3 2.37; F3w 0.94; P3 1.22; Ti3 1.44; Sd3 0.82; Mt3 1.47; Tr3 1.05; F4 3.12; F4w 0.91; P4 1.38; Ti4 2.18; Mt4 2.18; Tr4 1.22. Retrolateral face of tarsus IV with sparse/indistinct comb. Leg I spination pattern: TSp 0, TSpv 10, TSrd 0, TSr 0, TSrv 21, MtSp 0, MtSr 8, TrSp 0, TrSr 8. *Pedipalps*. PTl 2.27, PTw 0.82, Bl 1.76. Embolus evenly curved, bulb relatively large.

##### Variation, males

**(n = 26).**CL 3.02–4.84, 4±0.1; CW 2.92–4.73, 3.95±0.1; Cap 2.04–3.1, 2.59±0.06; ENDl 0.41–0.73, 0.59±0.02; ENDw 0.77–1.5, 1.06±0.03; STRl 1.81–2.81, 2.39±0.06; STRw 1.66–2.93, 2.39±0.07; LBl 0.48–0.83, 0.63±0.02; LBw 0.67–1.1, 0.87±0.02; F1 3.11–4.79, 3.99±0.09; F1w 0.65–1.08, 0.9±0.02; P1 1.39–2.26, 1.91±0.05; Ti1 1.98–3.28, 2.71±0.07; Mt1 1.37–2.27, 1.82±0.04; Tr1 0.72–1.08, 0.93±0.02; F3 2.02–3.26, 2.76±0.07; F3w 0.87–1.4, 1.12±0.03; P3 1.06–1.72, 1.44±0.04; Ti3 1.18–1.98, 1.65±0.04; Mt3 1.14–1.95, 1.62±0.04; Tr3 0.92–1.45, 1.23±0.03; F4 2.9–4.38, 3.8±0.08; F4w 0.82–1.35, 1.08±0.03; P4 1.3–2.01, 1.71±0.04; Ti4 1.9–2.95, 2.48±0.06; Mt4 1.94–3.61, 2.61±0.07; Tr4 0.36–1.8, 1.32±0.05; TSp 0–11, 4.76±0.58; TSpv 1–15, 7.56±0.7; TSr 0–8, 1.56±0.42; TSrv 15–36, 23.12±0.94; PTl 0.8–2.78, 2.35±0.08; PTw 0.74–2.15, 0.98±0.05; BL 1.19–1835, 75.17±73.33.

##### Material examined.

**United States: Arkansas: Bradley Co**: 33.5087 -92.2236^7^, 51 m a.s.l. (UMM0282, vi–vii.1964, 1♂, AMNH); **Washington Co**: Fayetteville, 36.0625 -94.1571^6^, 406 m a.s.l. (UMM0263, 11.vi.1961, 1♂, Whitcomb, AMNH); (UMM0205, 14–31.vii.1961, 1♂, N Causey, MCZ); University of Arkansas, 33.591 -91.8117^4^, 81 m a.s.l. (UMM0285, 23.vi.1965, 1♂, Dodge, AMNH); Fayetteville, 36.0625 -94.1571^6^, 406 m a.s.l. (UMM0134, 23.vi.1967, 1♂, WB Peck, AMNH); **Kansas: Cowley Co**: Winfield, 37.2398 -96.995^6^, 344 m a.s.l. (UMM0170, Summer 1935, 1♂, AMNH); **Douglas Co**: 5 mi S of K10 on Hwy59, 38.846 -95.3659^5^, 332 m a.s.l. (UMM0830, 13.vii.1990, 1♂, Blackwood, OSU); Fitch Natural History Reserve, 39.0635 -95.196^5^, 301 m a.s.l. (UMM0615, 4.vi.1988, 1♂, Guarisco, AMNH); **Jefferson Co**: 39.2739 -95.311^7^, 335 m a.s.l. (UMM0827, 18.vi.1989, 1♂, OSU); **Montgomery Co**: Elk City, Elk City Lake, 37.2621 -95.7816^5^, 287 m a.s.l. (UMM0826, 7.vii.1997, 1♂, E Miller, OSU); **Mississippi: Grenada Co**: 33.6963 -90.03^1^, 76 m a.s.l. (UMM0700, 21–27.viii.1991, 1♂, GT Baker, MEM); **Lafayette Co**: 1 mi SW of Abbeville, 34.4891 -89.5103^4^, 135 m a.s.l. (UMM0715, 17.x.2017, 1♂, PR Miller, MEM); 5 mi NW Oxford, 34.4477 -89.6499^6^, 102 m a.s.l. (UMM0717, 1♂, TG Forrest, MEM); **Oktibbeha Co**: 7 mi S of Starkville, 33.3485 -88.8178^5^, 81 m a.s.l. (UMM0175, 7.vi.1983, 1♀, G Miller, UFMNH); Osborn Prairie, 33.5113 -88.7355^1^, 99 m a.s.l. (UMM0701, 11.v-1.vi.1992, 1♂, JA MacGown, MEM); (UMM0706, 22.vii.1991, 1♂, DM Pollock, MEM); Starkville, 33.4503 -88.8183^6^, 101 m a.s.l. (UMM0174, 3.vi.1981, 1♂, WH Cross, UFMNH); **Missouri: Benton Co**: 4mi W of Warsaw, 38.2437 -93.4539^5^, 254 m a.s.l. (UMM0182, 29.iv.1973, 1♀, RS Frank, UFMNH); **Iron Co**: Graniteville, 37.9939 -91.8407^8^, 318 m a.s.l. (UMM0278, 10.x.1949, 1♀, Smith, Berger, AMNH); **Johnson Co**: Knob Noster State Park, 38.7496 -93.6065^5^, 246 m a.s.l. (UMM0128, 11–19.vi.1979, 1♂, Peck, Peaslee, AMNH); (UMM0127, 19–26.vi.1979, 1♂, AMNH); (UMM0136, 19–27.vi.1978, 1♂, AMNH); (UMM0126, 26.vi-2.vii.1979, 1♂, AMNH); **Vernon Co**: Nevada, 37.8391 -94.3546^6^, 264 m a.s.l. (UMM0204, 7.vi.1962, 1♂, JW McReynolds, MCZ); **Oklahoma: Carter Co**: 34.1875 -97.3484^7^, 273 m a.s.l. (UMM0833, 12.vi.1992, 1♂, L McDaniel, OSU); **Coal Co**: Lehigh, 34.4692 -96.2164^6^, 189 m a.s.l. (UMM0613, 1♀, AMNH); **Garvin Co**: Paul’s Valley, 34.7394 -97.222^5^, 267 m a.s.l. (UMM0821, 12.i.1987, 1♀, OSU); **Kay Co**: Ponca City, 36.7063 -97.0856^6^, 308 m a.s.l. (UMM0828, 2.vi.2006, 1♂, B Parker, OSU); **Logan Co**: Guthrie, 35.8783 -97.4253^6^, 292 m a.s.l. (UMM0125, 21.iv.1962, 1♀, RC Harrel, AMNH); **McCurtain Co**: Beaver’s Bend State Park, 34.1308 -94.6905^5^, 196 m a.s.l. (UMM0838, 14.vi.1977, 1♂, DC Arnold, OSU); **Oklahoma Co**: Oklahoma City, 35.4555 -97.5107^7^, 365 m a.s.l. (UMM0817, 12.ix.1990, 1♀, OSU); **Payne Co**: 1.6 mi W, 1 mi N Stillwater, 36.1376 -97.0972^5^, 279 m a.s.l. (UMM0238, 10.iv.1965, 1♂, VV Barrd, AMNH); Oklahoma State University Campus, 36.1275 -97.0794^5^, 292 m a.s.l. (UMM0235, 12.xi.1964, 1♀, RC Harrel, AMNH); (UMM0132, x.1964, 1♀, AMNH); Stillwater, 36.1142 -97.0551^6^, 270 m a.s.l. (UMM0815, 10.i.1975, 1♀, DC Arnold, OSU); (UMM0834, 10.ii.1974, 1♂, DC Arnold, OSU); (UMM0548, 4.vi.1973, 1♂, DC Arnold, AMNH); (UMM0617, 14.iv.1958, 1♀, B Branson, AMNH); (UMM0818, 14.v.1968, 1♀, JT Pitts, OSU); (UMM0601, 15.vi.1973, 1♂, Arnold, AMNH); (UMM0837, 24.vi.1972, 1♂, DR Moiner, OSU); (UMM0814, 26.ii.1992, 1♀, WA Drew, OSU); (UMM0824, 7.x.1979, 1♀, B Ree, OSU); (UMM0812, 9.xi.1967, 1♀, WA Dres, OSU); (UMM0836, Summer 1973, 1♂, S Miller, OSU); Stillwater, Lake Carl Blackwell, 36.1299 -97.2341^5^, 299 m a.s.l. (UMM0822, vii.1974, 1♀, R Wall, OSU); Stillwater, OSU forestry farm, 36.1142 -97.0551^6^, 270 m a.s.l. (UMM0816, 28.ix.1967, 1♀, L Johnson, OSU); **Tulsa Co**: Bixby, 35.9447 -35.9447^5^, 183 m a.s.l. (UMM0835, 27.vi.1978, 1♂, JM Nelson, OSU); (UMM0832, 5.vi.1978, 1♂, OSU); (UMM0839, 1♂, OSU); (UMM0831, 9.vi.1973, 1♂, OSU); Tulsa, 36.151 -95.9945^7^, 223 m a.s.l. (UMM0823, 8.iii.1974, 1♀, D Ringer, OSU); **Texas**: 31.6485 -99.7837^8^, 480 m a.s.l. (UMM0791, 7.x.1925, 1♂, R Watterschoid, MSU); **Anderson Co**: 11 mi ENE center of Palestine, 31.8178 -95.4144^1^, 92 m a.s.l. (UMM0105, 15–24.vi.2002, 1♂, J Yantis, TAMU); **Angelina Co**: 5.9 air miles SE Zavalla, 31.1186 -94.3366^1^, 82 m a.s.l. (UMM0851, 18.v.2013, 1♂, S Clarke, TAMU); (UMM0852, 26.iv-9.v.2012, 1♂, TAMU); 6 air miles SE Zavalla, 31.1164 -94.3345^1^, 84 m a.s.l. (UMM0856, 18.v.2013, 1♂, S Clarke, TAMU); Angelina National Forest, 4 mi SE Zavalla, 31.1278 -94.3658^1^, 81 m a.s.l. (UMM0846, 7–30.v.1996, 1♂, Clarke, Menard, Riley, TAMU); **Bastrop Co**: 30.1105 -97.3153^6^, 111 m a.s.l. (UMM0502, 23.iv.1977, 1♀, C Benton, AMNH); **Bell Co**: 3 mi S of Belton, 31.0125 -97.4613^5^, 147 m a.s.l. (UMM0271, 28.xii.1941, 1♀, AMNH); **Bexar Co**: 10 mi S of San Antonio at jct Hwy 16 and Medina River, 29.2614 -98.5823^4^, 166 m a.s.l. (UMM0786, 1.ix.1974, 1♀, W Icenogle, AMNH); **Bowie Co**: Texarkana, 33.4256 -94.0475^6^, 95 m a.s.l. (UMM0108, 20.xii.1993, 1♀, V Akins, TAMU); **Brazos Co**: Bryan, 30.6385 -96.3355^1^, 86 m a.s.l. (UMM0842, 5.viii.2012, 1♀, PC Krauter, TAMU); College Station, 30.6239 -96.3342^6^, 105 m a.s.l. (UMM0841, 27.iv.1979, 1♀, P Menefee, TAMU); College Station, Lick Creek Park, 30.5677 -96.2132^4^, 74 m a.s.l. (UMM0102, 30.vi-15.vii.2005, 1♂, T Henderson, TAMU); **Cameron Co**: Harlingen, 26.1911 -97.6976^5^, 12 m a.s.l. (UMM0840, 30.viii.2009, 1♀, M Wilson, TAMU); **Comanche Co**: 8mi E of Rising Star, 32.0957 -98.8281^5^, 460 m a.s.l. (UMM0110, 15.iv.1983, 1♀, CW Agnew, TAMU); **Coryell Co**: 31.4804 -97.8683^7^, 286 m a.s.l. (UMM0409, 29.vi.1948, 1♂, JH Robinson, AMNH); **Dallas**: 5–6 mi S Dallas, 32.6472 -96.7913^6^, 189 m a.s.l. (IZC327743, xii.1937 , 1♀, JC Sanders, AMNH); **Fayette Co**: 29.8901 -96.8351^7^, 109 m a.s.l. (UMM0560, 15.v.1977, 1♀, C Benton, AMNH); **Gregg Co**: Longview, 32.5003 -94.7398^6^, 96 m a.s.l. (UMM0600, 24.ix.1960, 1♀, N Brachi, AMNH); **Harris Co**: Houston, Rice Institute, 29.7166 -95.4026^4^, 33 m a.s.l. (UMM0410, 5.x.1954, 1♀, RD Barnes, AMNH); **Hays Co**: 6 mi NW of Dripping Springs, 30.3803 -98.21^1^, 408 m a.s.l. (UMM0850, 2.ix-5.x.2006, 1♂, EG Riley et al, TAMU); **Hidalgo Co**: Lower Rio Grande Valley National Wildlife Refuge, McManus Unit, 26.0538 -98.0499^1^, 32 m a.s.l. (UMM0853, 27.v-6.vi.2009, 1♂, J King, E Riley, TAMU); **Houston Co**: 19 mi NE of Crockett, 31.497 -95.2166^1^, 109 m a.s.l. (UMM0106, 27.v-6.vi.2002, 1♂, J Yantis, TAMU); 4 mi NNE center of Kennard, 31.4064 -95.1592^1^, 117 m a.s.l. (UMM0103, 2–11.vi.2002, 1♂, J Yantis, TAMU); 6 mi N of center of Radcliff, 31.4783 -95.1335^1^, 66 m a.s.l. (UMM0104, 3–12.vi.2002, 1♂, J Yantis, TAMU); **Kerr Co**: 6.5 mi SW Hunt, 29.0969 -99.0622^1^, 597 m a.s.l. (UMM0848, 2.ix-5.x.2006, 1♂, EG Riley et al, TAMU); **Lubbock Co**: Lubbock Lake, 33.6222 -101.8896^6^, 978 m a.s.l. (UMM0506, 31.vii.1979, 1♂, JC Cokendolpher, AMNH); **Madison Co**: 5 mi S of center of Madisonville, 30.8763 -95.9102^5^, 74 m a.s.l. (UMM0111, 1–10.x.2001, 1♂, J Yantis, TAMU); **McMullen Co**: 28.3104 -98.5721^7^, 69 m a.s.l. (UMM0573, 31.vii.1977, 1♀, C Benton, AMNH); **Newton Co**: Bon Wier, 30.7397 -93.6429^6^, 24 m a.s.l. (UMM0564, 1♂, AMNH); **Robertson Co**: Mumford, Holmes Pecan Orchard, 8 mi N Hwy 21 on FM 50, 30.7433 -96.56^1^, 79 m a.s.l. (UMM0855, 28.vi-5.vii.2001, 1♂, A Calixto, TAMU); **San Patricio Co**: Welder Wildlife Refuge, 28.113 -97.4164^5^, 12 m a.s.l. (UMM0854, 19.vi.1989, 1♂, JM Mora, TAMU); (UMM0857, 1♂, TAMU); **San Saba Co**: 3 mi E McCulloch, San Saba Co line on Hwy 190, 31.2479 -99.041^1^, 480 m a.s.l. (UMM0844, 23.vi.2008, 1♀, A Dean, S Dean, TAMU); **Travis Co**: Austin, 30.2671 -97.743^6^, 148 m a.s.l. (UMM0421, 26.v.1946, 1♂, Brown, HE Frizzell, AMNH); Austin, 30.2671 -97.743^6^, 148 m a.s.l. (UMM0568, ix.1941, 1♀, AMNH); Austin, 30.2675 -97.7438^6^, 161 m a.s.l. (UMM0233, viii.1956, 1♀, G Henderson Jr, AMNH); **Wichita Co**: Midwestern State University Farm, 33.931 -98.7481^7^, 313 m a.s.l. (UMM0803, 13.ii.1975, 1♀, T Emsoff, MSU); 33.931 -98.7481^7^, 313 m a.s.l. (UMM0801, 1.v.1995, 1♀, C Davis, MSU); (UMM0808, 11.viii.1996, 1♂, NV Horner, MSU); (UMM0802, 13.x.1997, 1♂, MSU); (UMM0797, 6.vii.2003, 1♀, MSU); (UMM0800, 15.xi.2001, 1♂, R Lowery, MSU); (UMM0799, 17.x.1988, 1♂, LJ Nessel, MSU); (UMM0795, 2.viii.1984, 1♀, TA Austin, MSU); (UMM0789, 29.vii.1997, 1♂, W Hanss, MSU); (UMM0796, 9.xii.1990, 1♂, J Mitchell, MSU).

#### Ummidia colemanae sp. nov.

Taxon classificationAnimaliaAraneaeHalonoproctidae

90025ED5-039B-579D-BFB7-225BCD8FB979

http://zoobank.org/5C68EB61-8B25-4F20-80CE-2795303743D1

Fig. 20 , Map 3

##### Type material.

HOLOTYPE: 1 ♂ (UMM504) from 8 miles NE Sinton, San Patricio County, Texas, 28.0852 -97.2647^1^, 2 m a.s.l., 28.iv.1960, coll. HE Laughlin, AMNH.

##### Etymology.

The specific epithet is a patronym named in honor of Texas native Bessie Coleman (1892–1926), the first African American and Native American woman to obtain her pilot’s license.

##### Diagnosis.

*Ummidia colemanae* is an extremely small species found in southern Texas. Males can be differentiated from *U. rongodwini* by the absence a pale dorsal heart patch and from *U. audouini* and *U. rosillos* by lacking a brush on the retrolateral face of tarsus IV. Comb on prolateral face of tarsus IV is more distinct than that of *U. beatula*, *U. mercedesburnsae*, and *U. macarthuri*. Males disperse from April to June.

**Figure 20.**
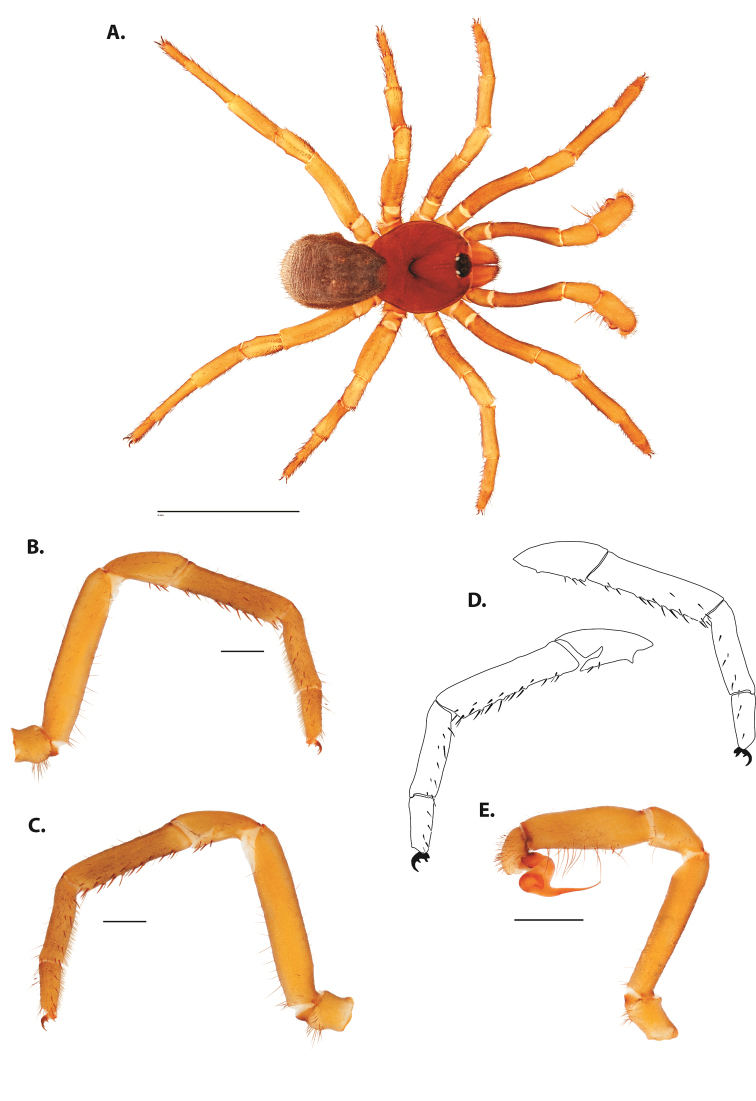
*Ummidia colemanae* sp. nov. male holotype specimen (UMM504) from Sinton, Texas **A** habitus illustration **B** prolateral aspect, leg I **C** retrolateral aspect, leg I **D** line drawings, leg I prolateral and retrolateral aspects **E** retrolateral aspect, pedipalp. Scale bars: 4.0 mm (**A**), 1.0 mm (**B–E**).

##### Description of male holotype.

*Specimen preparation and condition*. Specimen preserved in 80% EtOH. Left palp, leg I removed, in vial with specimen. Left leg II missing. *General coloration*. Carapace and chelicerae dark red 2.5YR 3/6, leg dark yellowish brown 10YR 4/6. Abdomen dark reddish gray 2.5YR 3/1. *Cephalothorax*. Carapace 2.73 long, 2.56 wide. Pars cephalica 1.76 long. Foveal groove procurved, 0.16 long, 0.43 wide. All eyes on dark, high tubercle. AER procurved. PER straight. Eye group 0.38 long, 0.73 wide, AME 0.19, PME 0.17, ALE 0.22, PLE 0.15. Sternum sparsely setose around outer 1/3, thicker anteriorly, STRl 1.61, STRw 1.5. Chelicerae with anterior tooth row comprising two teeth, posterior margin with four teeth. Palpal endites with 13 cuspules spread over proximal half of endite face, lacking distal endite cuspules, ENDw 0.63, ENDl 1.07. Labium with three cuspules, LBw 0.58, LBl 0.37. Rastellum with many spines on process. Abdomen setose with pale speckles concentrated into faint chevrons at apodemes. *Legs*. F1 2.73; F1w 0.54; P1 1.28; Ti1 1.78; Mt1 1.23; Tr1 0.72; F3 1.84; F3w 0.74; P3 0.93; Ti3 1.02; Sd3 0.53; Mt3 1.05; Tr3 0.81; F4 2.54; F4w 0.71; P4 1.11; Ti4 1.64; Mt4 1.75; Tr4 0.93. Retrolateral face of tarsus IV with defined comb over length of tarsus. Leg I spination pattern: TSp 2, TSpv 4, TSrd 0, TSr 0, TSrv 18, MtSp 6, MtSr 8, TrSp 2, TrSr 5. *Pedipalps*. PTl 1.8, PTw 0.65, Bl 1.4. Embolus evenly curved.

##### Females.

Unknown.

#### Ummidia funerea

Taxon classificationAnimaliaAraneaeHalonoproctidae

(Gertsch, 1936)

968D77F1-BF5F-5AB1-A5B5-B05051A95373

**Figure 21.**
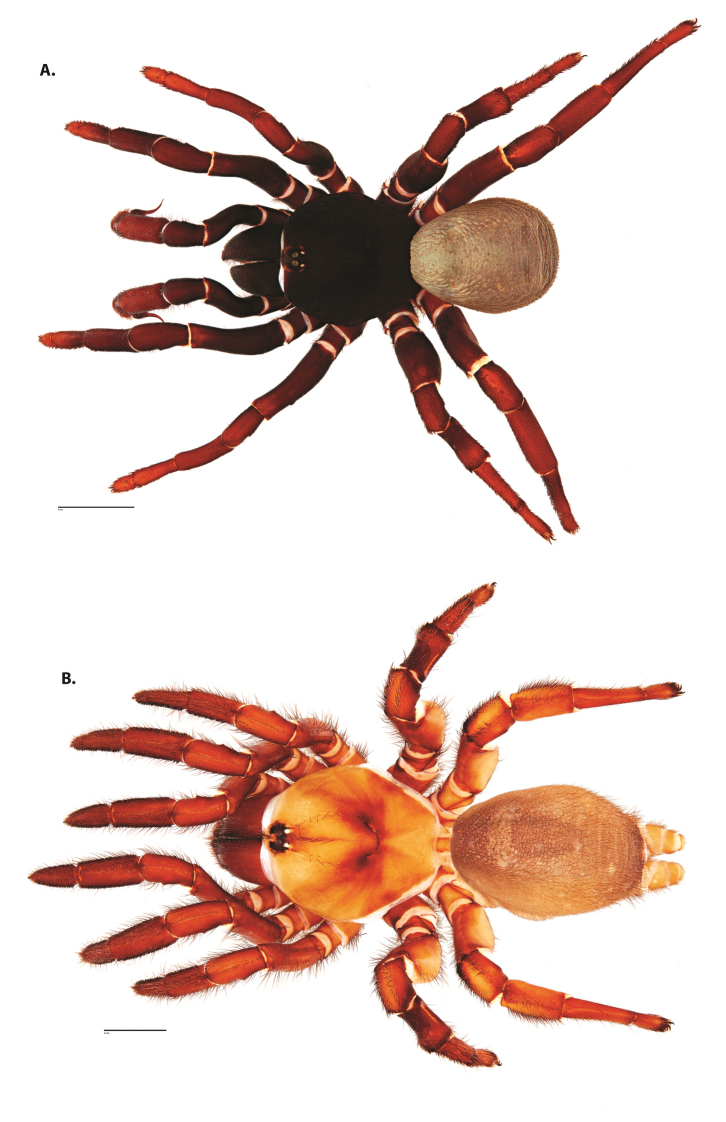
*Ummidia funerea* (Gertsch, 1936) from Edinburg, Texas. **A** male habitus illustration ICZ327741 **B** female habitus illustration UMM430. Scale bars: 4.0 mm.

**Figure 22.**
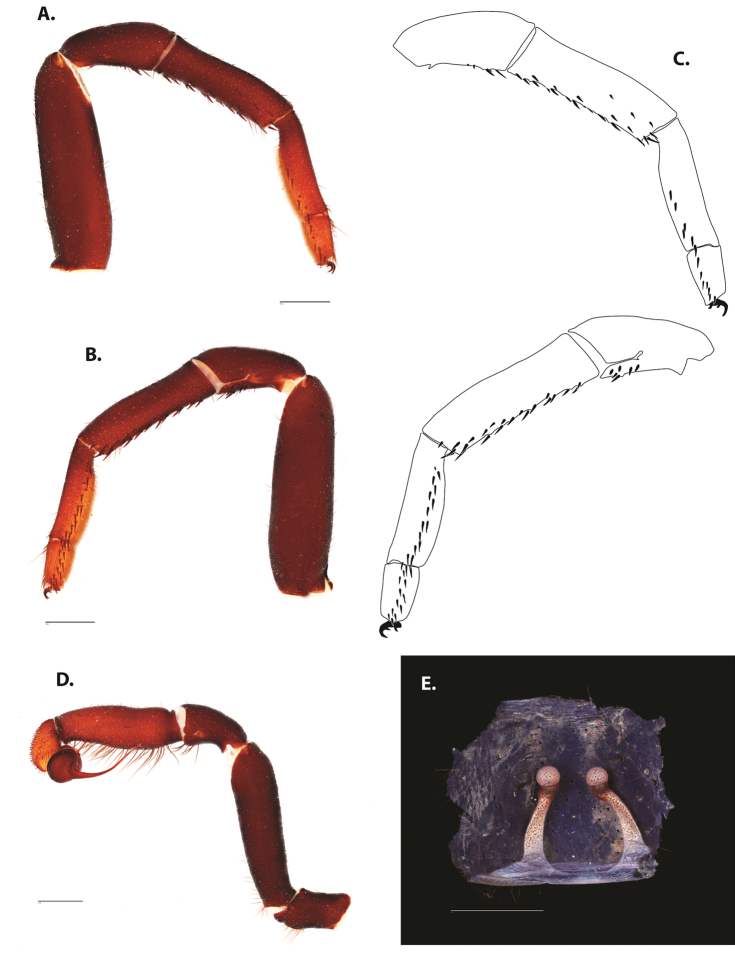
*Ummidia funerea* (Gertsch, 1936) from Edinburg, Texas. A-D male holotype (ICZ327741) **A** prolateral aspect, leg I **B** retrolateral aspect, leg I **C** line drawings, leg I prolateral and retrolateral aspects **D** retrolateral aspect, pedipalp **E** cleared spermathecae female exemplar (UMM430). Scale bars: 1.0 mm.

Figs 21 , 22 , Map 3

Pachylomerus funereus Gertsch, 1936, 1; HOLOTYPE: 1 ♂ (IZC00327741) from Edinburg, Texas 26.3005 -98.1623^6^, 25 m a.s.l., 1.vi.1935, coll. S Mulaik, deposited in AMNH, examined.
Pachylomerides celsus Gertsch & Mulaik, 1940, 313; HOLOTYPE: 1 ♂ (IZC327741) from 32miles SW of Laredo, Texas, United States, 5.viii.1935, coll. S Mulaik, deposited in AMNH, examined, **syn. nov.**
Ummidia celsa Brignoli, 1983, 117.

##### Diagnosis.

*Ummidia funerea* can be differentiated from all other species of *Ummidia* by having an anterior eye row which is extremely procurved, such that the posterior margin of the ALEs is even with the anterior margin of the AMEs and the ALEs are 2× as large as the PLEs.

##### Description of male holotype.

*Specimen preparation and condition*. Specimen preserved in 80% EtOH. Left palp, leg I and II Pt-Tr removed, in vial with specimen. *General coloration*. Carapace and chelicerae reddish black 10R 2.5/1, legs dark reddish brown 2.5YR 2.5/3. Abdomen very dark gray 10YR 3/1. *Cephalothorax*. Carapace 7.38 long, 7.17 wide. Pars cephalica 4.9 long. Foveal groove procurved, 0.5 long, 1.67 wide. Tubercle high. AER extremely procurved. PER slightly procurved. Eye group 1.14 long, 1.44 wide, AME 0.42, PME 0.22, ALE 0.62, PLE 0.34. Sternum with marginal fringe and few scattered setae, STRl 4.62, STRw 4.31. Chelicerae with anterior tooth row comprising six teeth, posterior margin with six teeth. Palpal endites with 22 cuspules spread over proximal half of endite face, lacking distal endite cuspules, ENDw 1.73, ENDl 3.05. Labium with nine cuspules, LBw 1.58, LBl 1.73. Rastellum with many spines. Abdomen setose, dorsally lighter with dorsodistal opalescence. *Legs*. F1 6.26; F1w 1.79; P1 3.37; Ti1 3.93; Mt1 2.93; Tr1 1.13; F3 4.61; F3w 2.12; P3 2.55; Ti3 2.48; Sd3 1.65; Mt3 2.99; Tr3 2.01; F4 6.01; F4w 2.02; P4 2.87; Ti4 3.78; Mt4 4.58; Tr4 2.03. Retrolateral face of tarsus IV with defined comb over proximal 2/3 of tarsus and paired spinules over distal third. Leg I spination pattern: TSp 0, TSpv 11, TSrd 1, TSr 0, TSrv 22, MtSp 6, MtSr 17, TrSp 7, TrSr 11. *Pedipalps*. PTl 3.38, PTw 1.37, Bl 3.26. Embolus relatively long, sinuous.

##### Variation, males

**(n = 6).**CL 6.08–9.1, 7.34±0.45; CW 5.56–8.21, 6.84±0.39; Cap 4.01–5.84, 4.82±0.27; ENDl 0.83–1.19, 1.03±0.07; ENDw 1.14–1.52, 1.37±0.06; STRl 3.62–4.98, 4.31±0.22; STRw 2.87–4.58, 3.82±0.26; LBl 0.91–1.27, 1.08±0.06; LBw 1.23–1.84, 1.5±0.09; F1 5.22–7.31, 6.03±0.32; F1w 1.54–2.02, 1.74±0.08; P1 2.54–3.89, 3.12±0.2; Ti1 3.2–4.79, 3.81±0.22; Mt1 2.38–3.06, 2.7±0.12; Tr1 0.87–1.34, 1.11±0.06; F3 3.57–5.53, 4.47±0.27; F3w 1.61–2.26, 1.95±0.1; P3 2.02–3.08, 2.48±0.15; Ti3 1.35–2.95, 2.27±0.23; Mt3 2.35–3.27, 2.73±0.15; Tr3 1.48–2.31, 1.86±0.12; F4 4.78–7.14, 5.72±0.35; F4w 1.53–2.18, 1.9±0.1; P4 2.36–3.39, 2.79±0.15; Ti4 3.07–4.26, 3.63±0.16; Mt4 3.61–5.05, 4.24±0.21; Tr4 1.57–2.24, 1.94±0.09; TSp 0–8, 3.5±1.31; TSpv 4–11, 6.67±0.95; TSr 0–0, 0±0; TSrv 19–28, 21.67±1.36; PTl 3–4.19, 3.44±0.16; PTw 1.09–1.52, 1.33±0.06; BL 2.66–3.26, 3.06±0.1.

##### Description of female exemplar.

*Specimen preparation and condition*. Specimen preserved in 80% EtOH. Spermathecae removed, cleared, in vial with specimen. Left leg II and abdomen removed, in vial with specimen *General coloration*. Carapace and posterior legs dark yellowish brown 10YR 4/6, chelicerae and anterior legs dark reddish brown 2.5YR 2.5/4. Abdomen dark brown 10YR 3/3, spinnerets strong brown 7.5YR 4/6. *Cephalothorax*. Carapace 10.43 long, 9.83 wide. Pars cephalica 6.96 long. Foveal groove procurved, 0.78 long, 2.22 wide. Eye tubercle low, under ME. AER extremely procurved PER procurved. Eye group 1.42 long, 1.84 wide, AME 0.35, PME 0.42, ALE 0.82, PLE 0.34. Sternum sparsely setose around outer edges, thicker anteriorly, STRl 6.15, STRw 6.07. Chelicerae with anterior row comprising six teeth, posterior margin with six teeth. Palpal endites with 47 cuspules spread across proximal half of endite and 53 cuspules distally; equal in size to proximal cuspules anteriorly, smaller posteriorly, ENDw 2.2, ENDl 4.16. Labium with 15 cuspules, LBw 2.19, LBl 1.67. Rastellum with many strong spines on process and up cheliceral face. *Abdomen*. Evenly setose. *Legs*. F1 6.73; F1w 2.32; P1 4.15; Ti1 4.19; Mt1 2.72; Tr1 1.11; F3 5.26; F3w 2.88; P3 3.44; Ti3 3.2, Sd3 1.86; Mt3 2.93; Tr3 2.18; F4 6.55; F4w 2.72; P4 3.99; Ti4 3.95; Mt4 4.2; Tr4 1.97. Retrolateral face tarsus IV with defined tight comb with heavy setae ventrally. *Pedipalps*. PF 6.09, PP 3.36, PTi 3.9, PTr 3.27. Spermathecae curved medially, bulbs facing anteriorly.

##### Variation, females

**(n = 7).**CL 3.81–10.43, 7.35±0.9; CW 3.28–9.83, 6.76±0.88; Cap 2.5–6.96, 4.96±0.62; ENDl 0.64–1.69, 1.14±0.13; ENDw 0.76–1.84, 1.28±0.13; STRl 2.32–6.18, 4.5±0.56; STRw 2.18–6.07, 4.3±0.55; LBl 0.55–1.67, 1.15±0.16; LBw 0.87–2.19, 1.6±0.18; F1 2.4–6.73, 4.77±0.62; F1w 0.86–2.32, 1.66±0.21; P1 1.54–4.15, 2.87±0.37; Ti1 1.39–4.19, 2.95±0.41; Mt1 1.06–2.98, 2.07±0.27; Tr1 0.57–1.19, 0.92±0.08; F3 1.19–5.4, 3.81±0.59; F3w 1.1–2.91, 2.13±0.26; P3 1.23–3.52, 2.52±0.33; Ti3 1.09–3.2, 2.24±0.31; Mt3 1.01–2.93, 2.06±0.27; Tr3 0.79–2.18, 1.52±0.19; F4 2.35–6.55, 4.79±0.59; F4w 1.08–2.83, 2.06±0.24; P4 1.46–3.99, 2.72±0.35; Ti4 1.51–3.95, 2.85±0.34; Mt4 1.35–4.24, 3.04±0.42; Tr4 0.68–1.97, 1.37±0.18; PF 2.13–6.09, 4.32±0.57; PP 1.35–3.44, 2.48±0.3; PTi 1.25–3.9, 2.73±0.37; PTr 1.23–3.43, 2.39±0.33.

##### Material examined.

**United States: Texas: Brooks Co**: 8 mi N Cantu, 31.9799 -99.9018^8^, 540 m a.s.l. (UMM0313, 29.xii.1949, 1juv, AMNH); **Cameron Co**: Laguna Atascosa National Wildlife Refuge, 26.2238 -97.3345^1^, 5 m a.s.l. (UMM0845, 15.v-4.vi.2010, 1♂, J King, E Riley, TAMU); Port Isabel, 26.0738 -97.207^5^, 3 m a.s.l. (UMM0847, 8.iv.1992, 1♂, S Sweeten, TAMU); **Hidalgo Co**: Edinburg, 10 mi NW Edinburg, 26.3005 -98.1623^6^, 25 m a.s.l. (IZC00327741, 1.vi.1935 , 1♂, S Mulaik, AMNH); Texas, 34.9197 -99.9195^8^, 540 m a.s.l. (UMM0262, 24.xii.1949, 1juv, Mulaik, AMNH); Edinburg, 26.3015 -98.1634^6^, 30 m a.s.l. (UMM0267, 29.xii.1949, 1♀, S Mulaik, D Mulaik, AMNH); (UMM0321, ix.1936, 1♂, S Mulaik, AMNH); (UMM0614, ix.1936, 1♂, Mulaik, AMNH); (UMM0416, 1♂, Mulaik, AMNH); **Starr Co**: Just S of Hwy 83, 2.5 mi W of Sullivan City, 26.3015 -98.6334^5^, 64 m a.s.l. (UMM0782, 6.ix.1974, 1juv, W Icenogle, AMNH); (UMM0783, 7.ix.1974, 1♀, AMNH); (UMM0784, 1juv, AMNH); **Webb Co**: Laredo, 27.5062 -99.5072^6^, 128 m a.s.l. (UMM0422, 1♂, R Baird, AMNH); Near Hwy 83, 1.8 mi N junction with Hwy 35, 15 mi N Laredo, 27.7567 -99.4406^5^, 212 m a.s.l. (UMM0430, 8.ix.1974, 1♀, W Icenogle, AMNH); (UMM0780, 9.ix.1974, 1♀, AMNH); (UMM0781, 1♀, AMNH).

### Western United States and northern Mexico

#### Ummidia rosillos sp. nov.

Taxon classificationAnimaliaAraneaeHalonoproctidae

4E53C54C-68C2-5BEA-A6F3-77A3E4A5A468

http://zoobank.org/A6AC84CB-73BE-4246-A05E-9CBAA8A7B969

Fig. 23 , Map 4

##### Type material.

HOLOTYPE: 1 ♂ (UMM810) from Rosillos Mountains, Big Bend National Park, Brewster County, Texas, United States, 29.5385 -103.2323^5^, 1386 m a.s.l., 12.vii.1991, coll. R Vogtsberger, DMNS.

##### Etymology.

The specific epithet is a noun taken in apposition and is in reference to the type locality.

##### Diagnosis.

*Ummidia rosillos* males can be differentiated from all geographically proximate species except *U. audouini* by the presence of a brush on the retrolateral face of tarsus IV and from all geographically proximate species by the presence of a pale dorsal heart patch. Males dispersing July to September.

##### Description of male holotype.

*Specimen preparation and condition*. Specimen preserved in 80% EtOH. Left palp and legs I removed; in vial with specimen. *General coloration*. Carapace and chelicerae reddish blank 7.5YR 2.5/1. Abdomen very dark brown 10YR 2/2 with heart patch light gray 10YR 7/2. Cephalothorax. Carapace 7.17 long, 6.87 wide. Pars cephalica 4.51 long. Foveal groove procurved, 0.45 long, 1.38 wide. Tubercle moderate; only under AME. AER procurved. PER straight. Eye group 0.7 long, 1.58 wide, AME 0.29, PME 0.16, ALE 0.4, PLE 0.16. Sternum sparsely setose, STRl 4.28, STRw 4.13. Chelicerae with anterior tooth row comprising five teeth, posterior margin with six teeth. Palpal endites with 13 cuspules spread over proximal half of endite face, lacking distal endite cuspules, ENDw 1.49, ENDl 2.85. Labium with five cuspules, LBw 1.49, LBl 1.04. Rastellum with several small teeth on process. Abdomen setose with irregular pale heart patch. Legs. F1 6.75; F1w 1.55; P1 3; Ti1 4.33; Mt1 2.69; Tr1 1.38; F3 4.95; F3w 1.95; P3 2.73; Ti3 2.98; Sd3 1.83; Mt3 2.61; Tr3 2.1; F4 6.6; F4w 1.91; P4 2.84; Ti4 4.13; Mt4 4.23; Tr4 2.13. Tarsus I with mixed dorsal cluster of clavate/filiform trichobothria. Retrolateral face of tarsus IV with brush. Leg I spination pattern: TSp 0, TSpv 0, TSrd 0, TSr 0, TSrv 2, MtSp 0, MtSr 0, TrSp 0, TrSr 0. Pedipalp: PTl 4.44, PTw 1.31, Bl 3.58. Embolus relatively long; sinuous.

**Figure 23.**
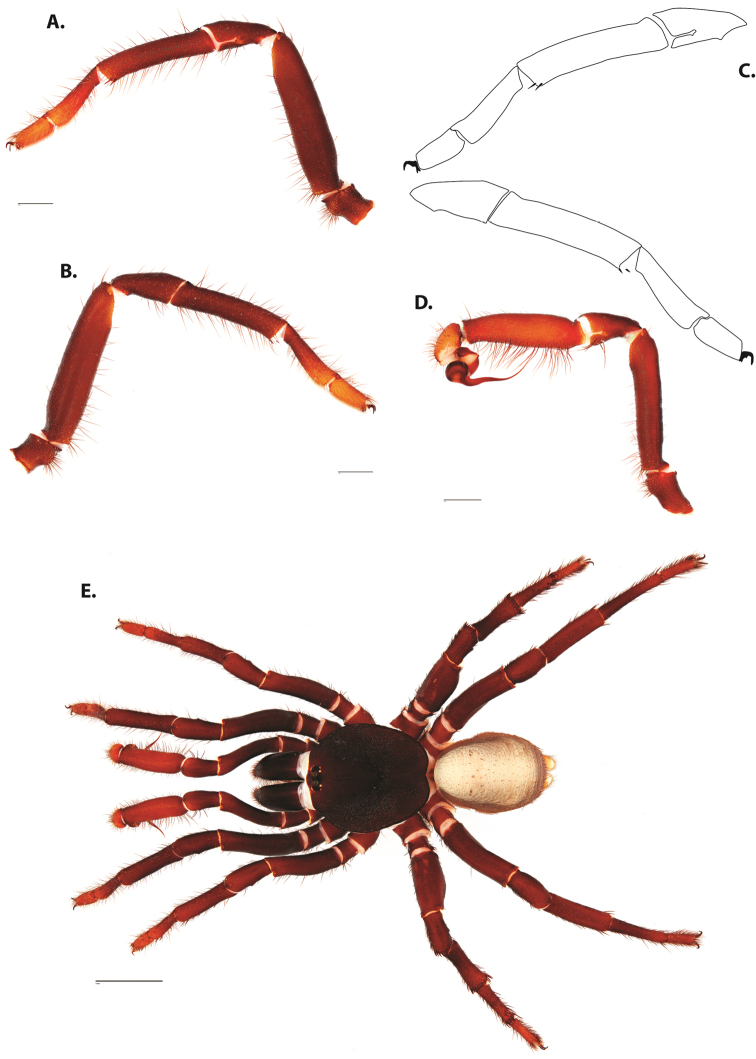
*Ummidia rosillos* sp. nov. male holotype (UMM810) from Brewster Co., Texas **A** retrolateral aspect, leg I **B** prolateral aspect, leg I **C** line drawings, leg I retrolateral and prolateral aspects **D** retrolateral aspect, pedipalp **E** habitus illustration. Scale bars: 1.0 mm (**A–D**), 4.0 mm (**E**).

##### Variation, males

**(n = 2).**CL 7.03–7.17, 7.1±0.07; CW 6.75–6.87, 6.81±0.06; Cap 4.51–4.61, 4.56±0.05; ENDl 0.7–0.79, 0.75±0.04; ENDw 1.49–1.58, 1.54±0.05; STRl 4.17–4.28, 4.23±0.05; STRw 3.7–4.13, 3.91±0.22; LBl 1.03–1.04, 1.03±0.01; LBw 1.41–1.49, 1.45±0.04; F1 6.07–6.75, 6.41±0.34; F1w 1.55–1.57, 1.56±0.01; P1 2.9–3, 2.95±0.05; Ti1 4.11–4.33, 4.22±0.11; Mt1 2.6–2.69, 2.64±0.04; Tr1 1.38–1.4, 1.39±0.01; F3 4.61–4.95, 4.78±0.17; F3w 1.89–1.95, 1.92±0.03; P3 2.6–2.73, 2.66±0.06; Ti3 2.91–2.98, 2.94±0.04; Mt3 2.61–2.9, 2.75±0.14; Tr3 2.09–2.1, 2.09±0.01; F4 5.98–6.6, 6.29±0.31; F4w 1.83–1.91, 1.87±0.04; P4 2.77–2.84, 2.81±0.03; Ti4 4.04–4.13, 4.09±0.05; Mt4 3.91–4.23, 4.07±0.16; Tr4 2.13–2.15, 2.14±0.01; TSp 0–0, 0±0; TSpv 0–5, 2.5±2.5; TSr 0–0, 0±0; TSrv 2–9, 5.5±3.5; PTl 4.02–4.44, 4.23±0.21; PTw 1.29–1.31, 1.3±0.01; BL 2.58–3.29, 2.94±0.36.

##### Females.

Unknown.

##### Material examined.

**United States: Texas: Brewster Co**: Big Bend Natl Park, at junction of US385 and St Rd 118, 29.3172 -103.3925^5^, 1006 m a.s.l. (UMM0625, 2.ix.1968, 1♂, JA Brubaker, FJ Moore, AMNH); Rosillos Mountains, Big Bend National Park, 29.5385 -103.2323^5^, 1386 m a.s.l. (UMM0810, 12.vii.1991, 1♂, R Vogtsberger, MSU).

#### Ummidia mercedesburnsae sp. nov.

Taxon classificationAnimaliaAraneaeHalonoproctidae

CC524BBB-6FD7-5C10-9F04-317B19A73E30

http://zoobank.org/8C1AC96D-255B-451D-BFB4-0FD047305AEA

**Figure 24.**
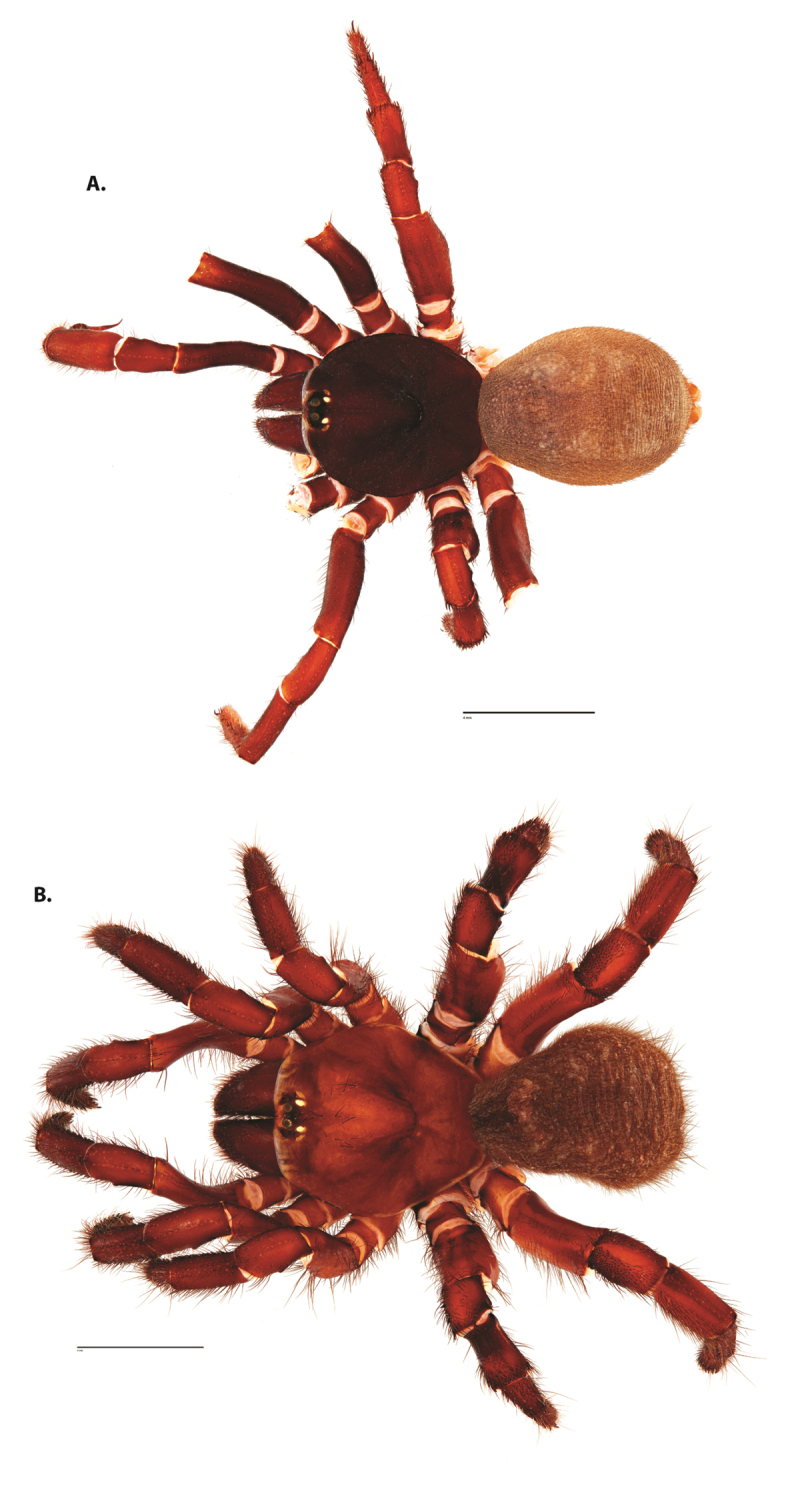
*Ummidia mercedesburnsae* sp. nov. from Culberson Co., Texas. **A** male habitus illustration UMM162 **B** female habitus illustration UMM644. Scale bars: 4.0 mm.

**Figure 25.**
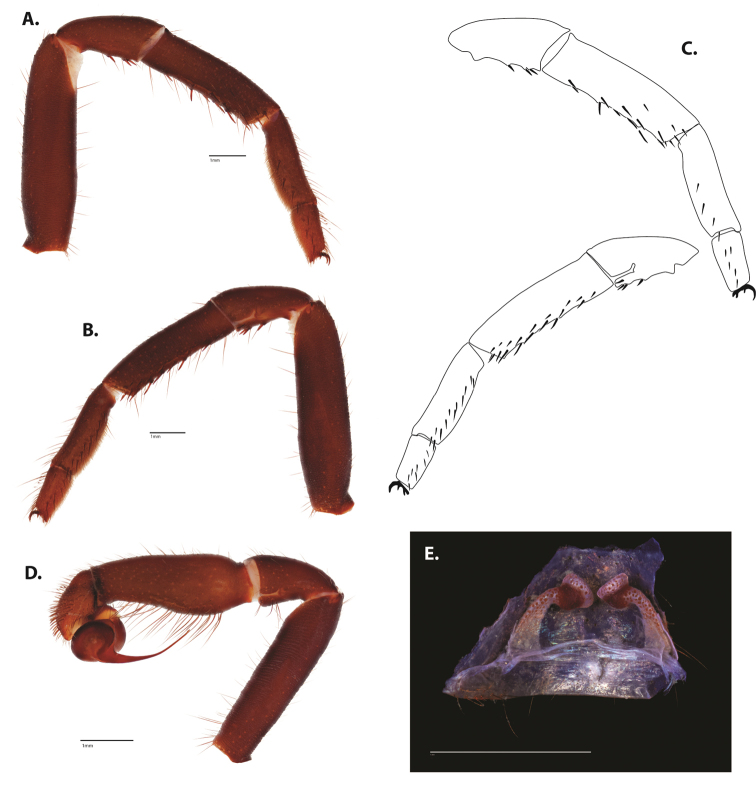
*Ummidia mercedesburnsae* sp. nov. from Culberson Co., Texas. **A–D** male holotype (UMM162) **A** prolateral aspect, leg I **B** retrolateral aspect, leg I **C** line drawings, leg I prolateral and retrolateral aspects **D** retrolateral aspect, pedipalp **E** cleared spermathecae female paratype (UMM644). Scale bars: 1.0 mm.

Figs 24 , 25 , Map 4

##### Type material.

HOLOTYPE: 1 ♂ (UMM162) from 2mi E of Nickel Creek Station, Culberson County, Texas, United States, 31.8945 -104.6331^6^, 1332 m a.s.l., 2.ix.1952, coll. B Malkin, AMNH. PARATYPE: 1 ♀ (UMM644) from Limpia Creek, 10mi N Fort Davis, 30.7368 -103.8969^6^, 1689 m a.s.l., 28.viii.1971, J Bull, AMNH.

##### Etymology.

The specific epithet is a patronym in honor of Dr. Mercedes Burns the first female African American arachnologist (to our knowledge).

##### Diagnosis.

*Ummidia mercedesburnsae* can be differentiated from *U. rosillos* by the lack of a pale dorsal heart patch and brush on the retrolateral face of tarsus IV. Males can be differentiated from *U. rosillos*, *U. modesta*, *U. timcotai*, *U. gabrieli*, and *U. rodeo* by having an embolus with an even curve and from *U. paulacushingae*, *U. waunekaae*, *U. gertschi*, *U. timcotai*, and *U. rodeo* by having relatively fewer spines on the retrolateral face of tibia I. Females can be differentiated from all other geographically proximate species except *U. modesta* by having spermathecae with strong medial bend. Males disperse in August.

##### Description of male holotype.

*Specimen preparation and condition*. Specimen preserved in 80% EtOH. Specimen with a number of leg articles removed, in vial with specimen. *General coloration*. Carapace and chelicerae reddish black 2.5YR 2.5/1, legs dark reddish brown 5YR 3/3. Abdomen dark grayish brown 10YR 4/2. *Cephalothorax*. Carapace 5.04 long, 4.95 wide. Pars cephalica 3.39 long. Foveal groove procurved, 0.39 long, 1.01 wide. All eyes on moderate tubercle. AER procurved. PER straight. Eye group 0.74 long, 1.25 wide, AME 0.36, PME 0.24, ALE 0.39, PLE 0.27. Sternum sparsely setose anteriorly with posterior fringe, STRl 2.94, STRw 3.02. Chelicerae with anterior tooth row comprising three teeth, posterior margin with three teeth. Palpal endites with 15 long, thin cuspules over proximal half of endite face, lacking distal endite cuspules, ENDw 1.24, ENDl 2.12. Labium with five long, thin cuspules, LBw 1.13, LBl 0.79. Rastellum with many spines on process. Abdomen setose. *Legs*. F1 4.92; F1w 1.24; P1 2.36; Ti1 3.09; Mt1 2.13; Tr1 1.01; F3 3.41; F3w 1.5; P3 1.84; Ti3 1.97; Sd3 1.07; Mt3 1.92; Tr3 1.44; F4 4.54; F4w 1.44; P4 2.1; Ti4 2.94; Mt4 2.98; Tr4 1.49. Retrolateral face of tarsus IV with indistinct comb. Leg I spination pattern: TSp 3, TSpv 4, TSrd 0, TSr 0, TSrv 14, MtSp 3, MtSr 11, TrSp 4, TrSr 6. *Pedipalps*. PTl 2.93, PTw 1.18, Bl 2.43. Embolus evenly curved.

##### Description of female paratype.

*Specimen preparation and condition*. Specimen preserved in 80% EtOH. Spermathecae removed, cleared, in vial with specimen. *General coloration*. Carapace, chelicerae, and legs dark reddish brown 2.5YR 2.5/4. Abdomen black 5YR 2.5/1, spinnerets dark reddish brown 2.5YR 2.5/2. *Cephalothorax*. Carapace 6.5 long, 6.15 wide. Pars cephalica 4.51 long. Foveal groove procurved, 0.64 long, 1.51 wide. Eye tubercle low. AER procurved. PER straight. Eye group 0.96 long, 1.67 wide, AME 0.37, PME 0.3, ALE 0.54, PLE 0.38. Sternum sparsely setose around edges, thicker anteriorly, STRl 3.95, STRw 4.19. Chelicerae with anterior row comprising six teeth, posterior margin with five teeth. Palpal endites with 27 cuspules spread across proximal half of endite and 40 cuspules distally, equal in size to proximal cuspules anteriorly, smaller posteriorly, ENDw 1.68, ENDl 2.78. Labium with six cuspules, LBw 1.64, LBl 1.12. Rastellum with many strong spines on process and up cheliceral face. *Abdomen*. *Legs*. F1 4.29; F1w 1.58; P1 2.69; Ti1 2.64; Mt1 1.92; Tr1 0.9; F3 3.67; F3w 1.96; P3 2.3; Ti3 2.08, Sd3 1.11; Mt3 1.69; Tr3 1.63; F4 4.36; F4w 1.93; P4 2.38; Ti4 2.52; Mt4 2.65; Tr4 2.52. Retrolateral face tarsus IV with defined tight comb over length of tarsus. *Pedipalps*. PF 3.83, PP 2.25, PTi 2.38, PTr 2.17. Spermathecae with medial bend, bulbs facing anteriorly.

##### Known only from type material.

#### Ummidia paulacushingae sp. nov.

Taxon classificationAnimaliaAraneaeHalonoproctidae

C902FE90-25ED-53AE-859F-C74A96415606

http://zoobank.org/7E09E4EF-34C2-44DB-9083-D70911DA816A

Fig. 26 , Map 4

##### Type material.

HOLOTYPE: 1 ♂ (DMNS_ZA40253) from Grand Junction, Mesa County, Colorado, United States 39.0636 -108.5518^6^, 1399 m a.s.l., 14.ix.2009, RW Hammon, DMNS.

##### Etymology.

The specific epithet is a patronym in honor of arachnologist Dr. Paula Cushing who is also the curator of arachnids for the Denver Museum of Natural History and first female president of the American Arachnological Society. The second author is generally afraid of her.

**Figure 26.**
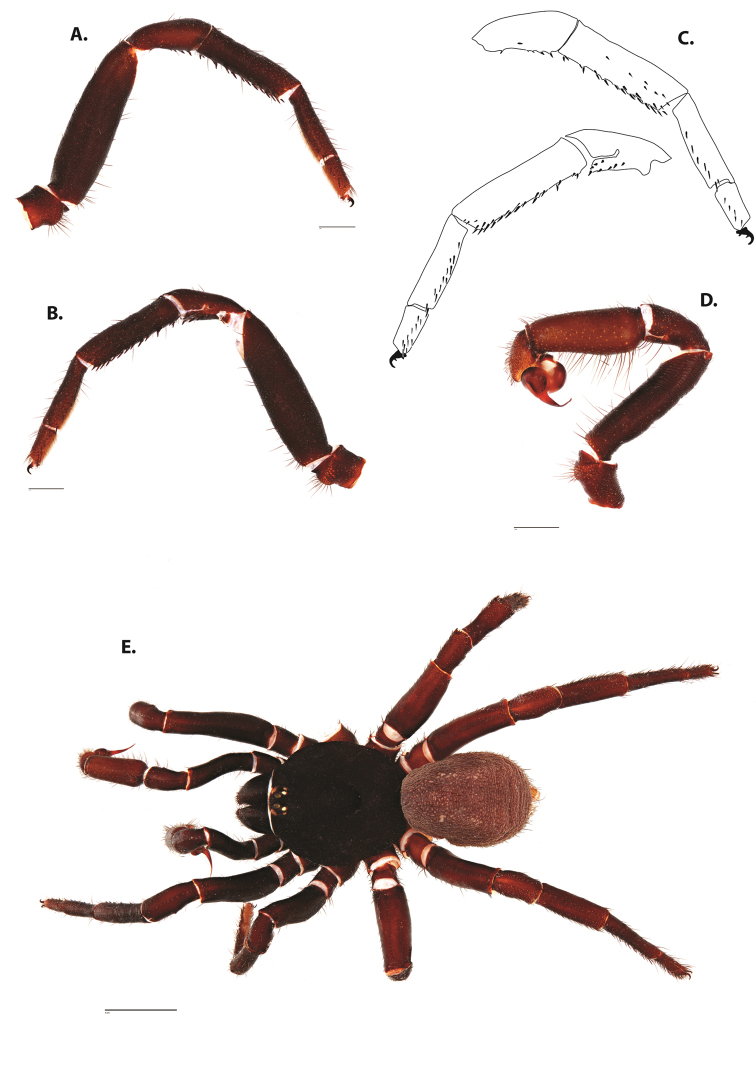
*Ummidia paulacushingae* sp. nov. male holotype specimen (CMNS40253) from Mesa Co., Colorado **A** retrolateral aspect, leg I **B** prolateral aspect, leg I **C** line drawings, leg I retrolateral and prolateral aspects **D** retrolateral aspect, pedipalp **E** habitus illustration. Scale bars: 1.0 mm (**A–D**), 4.0 mm (**E**).

##### Diagnosis.

*Ummidia paulacushingae* can be differentiated from *U. rosillos* by the lack of a pale dorsal heart patch and brush on the retrolateral face of tarsus IV. Males can be differentiated from *U. rosillos*, *U. modesta*, *U. timcotai*, *U. gabrieli*, and *U. rodeo* by having an embolus with an even curve and from *U. mercedesburnsae* by having relatively more spines on the retrolateral face of tibia I. Males can also be differentiated from all other geographically proximal species except *U. gertschi* by having a distinct comb comprising close set heavy setae on the retrolateral face of tarsus IV.

##### Description of male holotype.

*Specimen preparation and condition*. Specimen preserved in 80% EtOH. Right leg II removed, in vial with specimen. *General coloration*. Carapace, chelicerae, and legs black 5YR 2.5/1. Abdomen reddish black 2.5YR 2.5/1. *Cephalothorax*. Carapace 7.35 long, 7.13 wide. Pars cephalica 4.94 long. Foveal groove procurved, 0.49 long, 1.62 wide. Tubercle moderate with all eyes on tubercle. AER procurved. PER straight. Eye group 0.92 long, 1.64 wide, AME 0.33, PME 0.29, ALE 0.53, PLE 0.24. Sternum sparsely setose around outer 1/3, thicker anteriorly, STRl 4.32, STRw 4.42. Chelicerae with anterior tooth row comprising seven teeth, posterior margin with four teeth. Palpal endites with 33 cuspules spread over proximal half of endite face, lacking distal endite cuspules, ENDw 1.71, ENDl 2.96. Labium with 13 cuspules, LBw 1.56, LBl 1.08. Rastellum with many spines. Abdomen setose. *Legs*. F1 6.61; F1w 1.86; P1 3.33; Ti1 4.03; Mt1 3; Tr1 1.31; F3 4.77; F3w 2.02; P3 2.55; Ti3 3; Sd3 2.06; Mt3 3.17; Tr3 1.95; F4 6.27; F4w 1.93; P4 2.95; Ti4 3.86; Mt4 4.21; Tr4 2.14. Retrolateral face of tarsus IV with defined comb over proximal half of tarsus and paired spinules distally. Leg I spination pattern: TSp 6, TSpv 7, TSrd 0, TSr 2, TSrv 24, MtSp 5, MtSr 12, TrSp 5, TrSr 13. *Pedipalps*. PTl 3.52, PTw 1.51, Bl 3.27. Embolus evenly curved.

##### Variation, males

**(n = 2).**CL 6.69–7.35, 7.02±0.33; CW 6.63–7.13, 6.88±0.25; Cap 4.35–4.94, 4.64±0.3; ENDl 0.89–0.92, 0.91±0.01; ENDw 1.49–1.64, 1.56±0.07; STRl 3.73–4.32, 4.03±0.29; STRw 4.01–4.42, 4.21±0.2; LBl 1.03–1.08, 1.05±0.03; LBw 1.56–1.58, 1.57±0.01; F1 6.54–6.61, 6.57±0.04; F1w 1.68–1.86, 1.77±0.09; P1 3.12–3.33, 3.22±0.11; Ti1 4.03–4.2, 4.11±0.09; Mt1 3–3.2, 3.1±0.1; Tr1 1.29–1.31, 1.3±0.01; F3 4.66–4.77, 4.72±0.06; F3w 1.85–2.02, 1.94±0.08; P3 2.38–2.55, 2.46±0.08; Ti3 2.81–3, 2.9±0.1; Mt3 2.82–3.17, 2.99±0.17; Tr3 1.84–1.95, 1.89±0.06; TSp 0–6, 3±3; TSpv 5–7, 6±1; TSr 0–2, 1±1; TSrv 12–24, 18±6; PTl 3.52–3.54, 3.53±0.01; PTw 1.39–1.51, 1.45±0.06; BL 3.03–3.27, 3.15±0.12.

##### Females.

Unknown.

##### Material examined.

**United States: Colorado: Delta Co**: F Street, Crawford, 38.7058 -107.61^1^, 2000 m a.s.l. (DMNS_ZA37484, 17.x.2001, 1♂, B McCullaugh, DMNS); **Mesa Co**: Grand Junction, 39.0636 -108.5518^6^, 1399 m a.s.l. (DMNS_ZA40253, 14.ix.2009, 1♂, RW Hammon, DMNS).

#### Ummidia modesta

Taxon classificationAnimaliaAraneaeHalonoproctidae

(Banks, 1901)

FB740DAF-C66D-555E-B1C9-2AF01B59A78B

**Figure 27.**
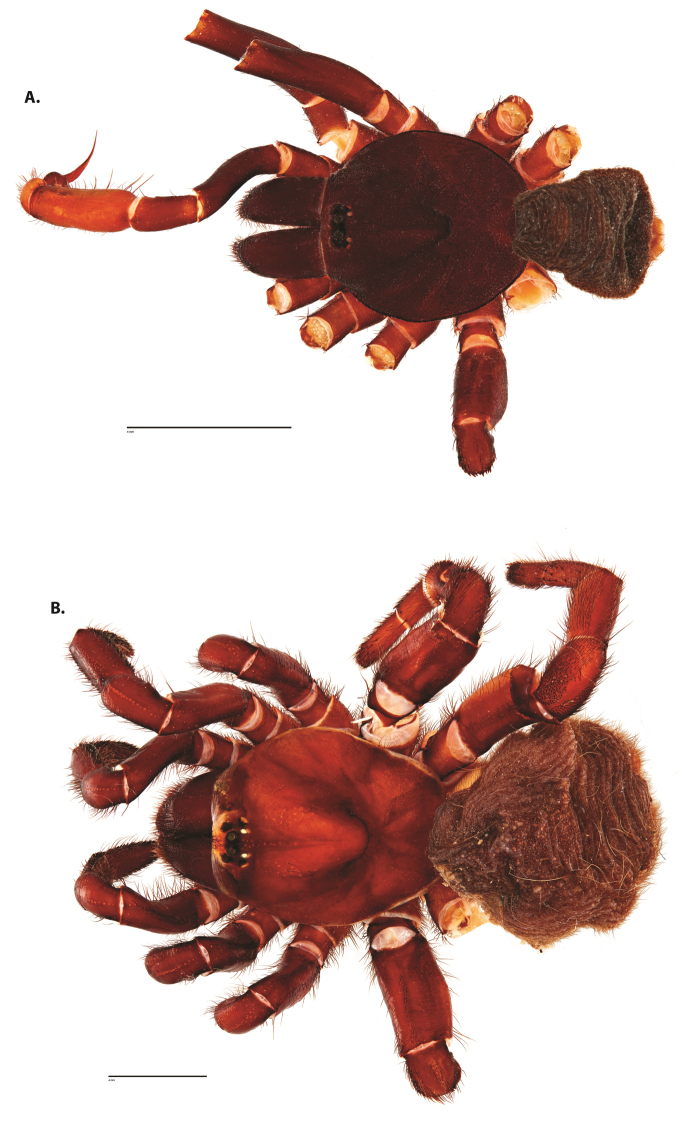
*Ummidia modesta* (Banks, 1901) from New Mexico **A** male habitus illustration MCZ22137 **B** female habitus illustration UMM586. Scale bars: 4.0 mm.

**Figure 28.**
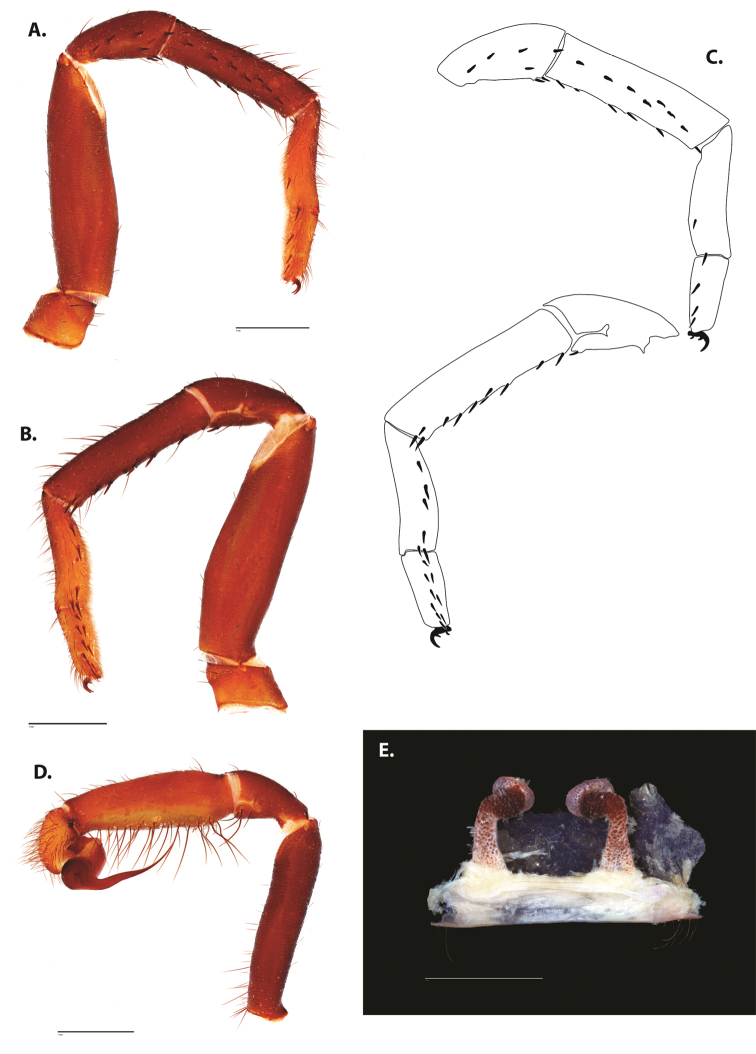
*Ummidia modesta* (Banks, 1901) from Las Cruces, New Mexico. male holotype (MCZ22137) **A** prolateral aspect, leg I **B** retrolateral aspect, leg I **C** line drawings, leg I prolateral and retrolateral aspects **D** retrolateral aspect, pedipalp **E** cleared spermathecae female paratype (UMM586). Scale bars: 1.0 mm (**A–D**).

Figs 27 , 28 , Map 4

Pachylomerus modestus Banks, 1901b: 570; HOLOTYPE: 1 ♂ (IZ22137) from Las Cruces, Dona Ana County, New Mexico, 32.338 -106.7661^5^, 1238 m a.s.l. coll. Nathan Banks, deposited in MCZ, examined. PARATYPE: 1 ♀ (UMM586, AMNH) from Las Cruces, Dona Ana County, New Mexico, 32.338 -106.7661^5^, 1238 m a.s.l., 23–27.ii.1937, coll. W.L. Chapel, AMNH.

##### Diagnosis.

*Ummidia modesta* can be differentiated from *U. rosillos* by the lack of a pale dorsal heart patch and brush on the retrolateral face of tarsus IV. Males can be differentiated from *U. mercedesburnsae*, *U. paulacushingae*, *U. waunekaae*, and *U. gertschi* by having a sinuous embolus and from *U. paulacushingae*, *U. waunekaae*, *U. gertschi*, *U. timcotai*, and *U. rodeo* by having relatively fewer spines on tibia I. Females can be differentiated from all other geographically proximate species except *U. mercedesburnsae* by having spermathecae with strong medial bend.

##### Description of male holotype.

*Specimen preparation and condition*. Specimen preserved in 80% EtOH. In poor condition, specimen in many pieces. *General coloration*. Carapace and chelicerae reddish black 10R 2.5/1, legs dark reddish brown 5YR 3/4. Abdomen reddish black 2.5YR 2.5/1. *Cephalothorax*. Carapace 5.05 long, 4.79 wide. Pars cephalica 3.18 long. Foveal groove procurved, 0.33 long, 0.88 wide. Tubercle moderate. AER procurved. PER slightly procurved. Eye group 0.63 long, 1.12 wide, AME 0.26, PME 0.17, ALE 0.35, PLE 0.16. Sternum with marginal fringe, STRl 2.89, STRw 2.68. Chelicerae with anterior tooth row comprising five teeth, posterior margin with five teeth. Palpal endites with 13 cuspules spread over proximal half of endite face, lacking distal endite cuspules, ENDw 1.12, ENDl 2. Labium with five cuspules, LBw 0.91, LBl 0.77. Rastellum with many spines. Abdomen setose. *Legs*. F1 4.68; F1w 1.21; P1 2.14; Ti1 3.12; Mt1 2.05; Tr1 1.23; F3 3.47; F3w 1.41; P3 1.85; Ti3 1.94; Sd3 1.34; Mt3 2.07; Tr3 1.63; F4 4.62; F4w 1.32; P4 2.11; Ti4 2.96; Mt4 3.17; Tr4 1.59. Retrolateral face of tarsus IV with distinct comb of spinules which is surrounded in setae. Leg I spination pattern: TSp 7, TSpv 1, TSrd 0, TSr 0, TSrv 10, MtSp 2, MtSr 6, TrSp 3, TrSr 9. *Pedipalps*. PTl 3.03, PTw 1.04, Bl 2.58. Embolus relatively long, sinuous.

##### Description of female paratype.

*Specimen preparation and condition*. Specimen preserved in 80% EtOH. Spermathecae removed, cleared, and in vial with specimen. Specimen in poor condition, many limbs broken, in vial with specimen; right leg III missing. *General coloration*. Carapace, chelicerae, and legs dark reddish brown 2.5YR 2.5/4. Abdomen reddish black 10R 2.5/1, spinnerets strong brown 7.5YR 5/6. *Cephalothorax*. Carapace 9.86 long, 8.8 wide. Pars cephalica 6.74 long. Foveal groove procurved, 0.78 long, 2.17 wide. Eye tubercle moderate under median eyes. AER procurved PER straight. Eye group 1.47 long, 2.26 wide, AME 0.49, PME 0.38, ALE 0.6, PLE 0.42. Sternum sparsely setose around edges, thicker anteriorly, STRl 5.84, STRw 6.15. Chelicerae with anterior row comprising six teeth, posterior margin with five teeth. Palpal endites with 34 cuspules spread across proximal half of endite and 40 smaller cuspules distally, ENDw 2.25, ENDl 3.87. Labium with seven cuspules, LBw 2.3, LBl 1.6. Rastellum with many strong spines on process and up cheliceral face. *Abdomen*. Evenly setose. *Legs*. F1 6.14; F1w 2.26; P1 3.34; Ti1 3.94; Mt1 2.86; Tr1 1.4; F3 5.24; F3w 2.75; P3 3.49; Ti3 3.19, Sd3 2.08; Mt3 2.52; Tr3 2.36; F4 6.8; F4w 2.71; P4 3.9; Ti4 3.79; Mt4 4.07; Tr4 2.31. Retrolateral face tarsus IV with defined, tight comb with clusters of spinules and proximal and distal ends. *Pedipalps*. PF 5.44, PP 3.26, PTi 3.74, PTr 3.12. Spermathecae with medial bend, bulbs facing ventrally.

##### Material examined.

Known only from type material.

#### Ummidia waunekaae sp. nov.

Taxon classificationAnimaliaAraneaeHalonoproctidae

13C3A966-9E53-5E72-8FDA-5B6A7E8D76CA

http://zoobank.org/7C7F8491-084E-4CE7-A695-B765DF297BFF

Fig. 29 , Map 4

##### Type material.

HOLOTYPE: 1 ♂ (UMM559) from northeast of Albuquerque, Bernalillo County, New Mexico, United States, 35.1303 -106.5521^5^, 1667 m a.s.l., 30.x.1963, coll. L. Oakley, AMNH.

##### Etymology.

The specific epithet is a patronym in honor of Annie Dodge Wauneka (1910–1997), influential member of the Navajo Nation who worked tirelessly to improve education and health of the Navajo. Among other awards, she was bestowed the United States Presidential Medal of Freedom in 1963 by President Lyndon B. Johnson.

##### Diagnosis.

*Ummidia waunekaae* can be differentiated from *U. rosillos* by the lack of a pale dorsal heart patch and brush on the retrolateral face of tarsus IV. Males can be differentiated from *U. rosillos*, *U. modesta*, *U. timcotai*, *U. gabrieli*, and *U. rodeo* by having an embolus with an even curve and from *U. mercedesburnsae* by having relatively more spines on the retrolateral face of tibia I. Males can also be differentiated from *U. gertschi* and *U. modesta* by lacking a distinct comb on the retrolateral face of tarsus IV. Males disperse in October.

**Figure 29.**
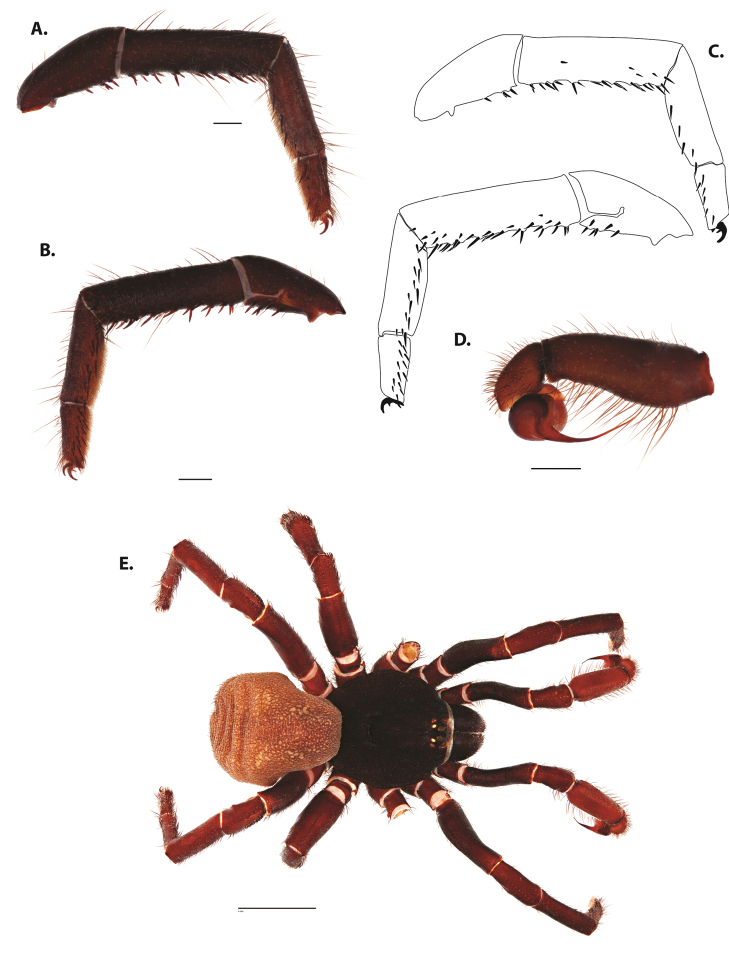
*Ummidia waunekaae* sp. nov. male holotype (UMM559) from Bernalillo Co., New Mexico. **A** prolateral aspect, leg I **B** retrolateral aspect, leg I **C** line drawings, leg I prolateral and retrolateral aspects **D** retrolateral aspect, pedipalp **E** habitus illustration. Scale bars: 1.0 mm (**A–D**), 4.0 mm (**E**).

##### Description of male holotype.

*Specimen preparation and condition*. Specimen preserved in 80% EtOH. Specimen in poor condition. *General coloration*. Carapace, chelicerae, and legs very dusky red 10R 2.5/1. Abdomen dark reddish brown 5YR 2.5/2. *Cephalothorax*. Carapace 6.16 long, 6.35 wide. Pars cephalica 4.29 long. Foveal groove procurved, 0.47 long, 1.24 wide. All eyes on moderate tubercle. AER procurved. PER straight. Eye group 0.92 long, 1.49 wide, AME 0.35, PME 0.33, ALE 0.5, PLE 0.28. Sternum sparsely setose around outer 1/3, thicker anteriorly, STRl 3.51, STRw 3.96. Chelicerae with anterior tooth row comprising five teeth, posterior margin with six teeth. Palpal endites with 17 cuspules spread over proximal half of endite face, and 12 cuspules distally, ENDw 1.59, ENDl 2.74. Labium with nine cuspules, LBw 1.69, LBl 1.1. Rastellum with many spines on process. Abdomen setose with pale speckles. *Legs*. F1 5.71; F1w 1.6; P1 2.89; Ti1 3.54; Mt1 2.39; Tr1 1.3; F3 4.16; F3w 1.86; P3 2.19; Ti3 2.46; Sd3 1.36; Mt3 2.19; Tr3 1.79; F4 5.44; F4w 1.79; P4 2.67; Ti4 3.56; Mt4 3.63; Tr4 1.91. Retrolateral face of tarsus IV with indistinct irregular row of spinules, no defined comb. Leg I spination pattern: TSp 3, TSpv 8, TSrd 0, TSr 1, TSrv 17, MtSp 5, MtSr 13, TrSp 6, TrSr 10. *Pedipalps*. PTl 3.36, PTw 1.39, Bl 2.77. Embolus evenly curved.

##### Variation, males

**(n = 2).**CL 5.99–6.16, 6.08±0.09; CW 5.71–6.35, 6.03±0.32; Cap 3.8–4.29, 4.04±0.24; ENDl 0.79–0.92, 0.86±0.06; ENDw 1.25–1.49, 1.37±0.12; STRl 3.19–3.51, 3.35±0.16; STRw 3.23–3.96, 3.59±0.37; LBl 0.9–1.1, 1±0.1; LBw 1.18–1.69, 1.44±0.25; F1 4.87–5.71, 5.29±0.42; F1w 1.37–1.6, 1.49±0.12; P1 2.56–2.89, 2.72±0.17; Ti1 3.33–3.54, 3.43±0.1; Mt1 2.17–2.39, 2.28±0.11; Tr1 1.26–1.3, 1.28±0.02; F3 3.59–4.16, 3.87±0.28; F3w 1.57–1.86, 1.71±0.14; P3 1.47–2.19, 1.83±0.36; Ti3 2.12–2.46, 2.29±0.17; Mt3 2.19–2.19, 2.19±0; Tr3 1.47–1.79, 1.63±0.16; F4 4.67–5.44, 5.06±0.39; F4w 1.57–1.79, 1.68±0.11; P4 2.25–2.67, 2.46±0.21; Ti4 3.22–3.56, 3.39±0.17; Mt4 3.21–3.63, 3.42±0.21; Tr4 1.58–1.91, 1.75±0.17; TSp 3–5, 4±1; TSpv 3–8, 5.5±2.5; TSr 0–1, 0.5±0.5; TSrv 16–17, 16.5±0.5; PTl 2.78–3.36, 3.07±0.29; PTw 1.3–1.39, 1.34±0.04; BL 2.58–2.77, 2.67±0.09.

##### Females.

Unknown.

##### Material examined.

**United States: New Mexico: Bernalillo Co**: NE Albuquerque, 35.1303 -106.5521^5^, 1667 m a.s.l. (UMM0559, 30.x.1963, 1♂, L Oakley, AMNH); La Tremen Fina, 34.9672 -105.0323^8^, 1734 m a.s.l. (UMM0224, 1♂, MCZ).

#### Ummidia gertschi sp. nov.

Taxon classificationAnimaliaAraneaeHalonoproctidae

446E51CB-A247-5A2B-82BA-30D3C6C37A16

http://zoobank.org/CBA55F87-B442-4A85-B770-80FA3765D4D2

**Figure 30.**
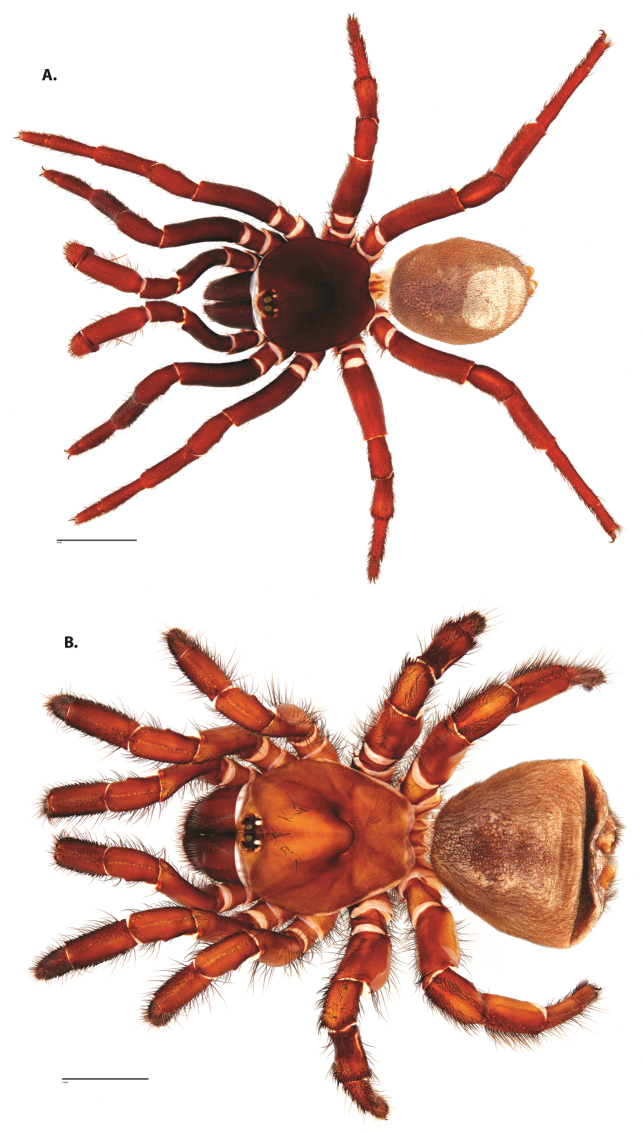
*Ummidia gertschi* sp. nov. from Cochise Co., Arizona. **A** male habitus illustration UMM165 **B** female habitus illustration UMM664. Scale bars: 4.0 mm.

**Figure 31.**
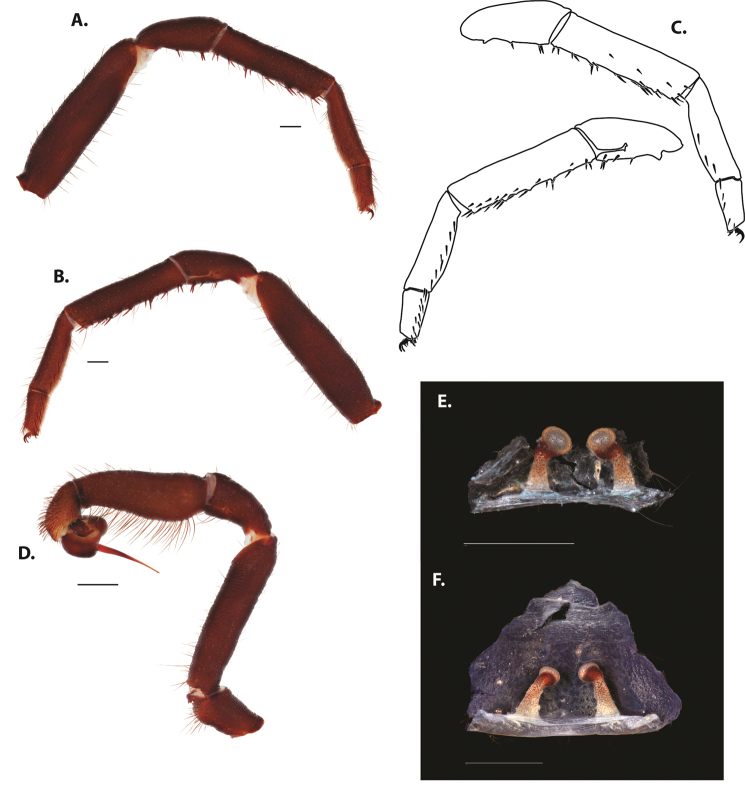
*Ummidia gertschi* sp. nov. from Cochise Co., Arizona **A–D** male holotype (UMM165) **A** prolateral aspect, leg I **B** retrolateral aspect, leg I **C** line drawings, leg I prolateral and retrolateral aspects **D** retrolateral aspect, pedipalp **E** cleared spermathecae female exemplar (UMM664) **F** cleared spermathecae female paratype (UMM572). Scale bars: 1.0 mm.

Figs 30 , 31 , Map 4

##### Type material.

HOLOTYPE: 1 ♂ (UMM165) and PARATYPE: 1 ♀ (UMM572) from 5 miles west of Portal, Southwestern Research Station, Cochise County, Arizona, 31.8842 -109.206^5^, 1645 m a.s.l., 10–20.vii.1955, coll. WJ Gertsch, AMNH.

##### Etymology.

The specific epithet is a patronym named for Dr. Willis J. Gertsch, famous arachnologist who put decades of work into a revision of *Ummidia* and the then Ctenizidae but was unable to finish before his passing; and whose notes, stored in the archives of the American Museum of Natural History, proved invaluable in the completion of this revision.

##### Diagnosis.

*Ummidia gertschi* can be differentiated from *U. rosillos* by the lack of a pale dorsal heart patch and brush on the retrolateral face of tarsus IV. Males can be differentiated from *U. rosillos*, *U. modesta*, *U. timcotai*, *U. gabrieli*, and *U. rodeo* by having an embolus with an even curve and from *U. mercedesburnsae* by having relatively more spines on the retrolateral face of tibia I. Males can also be differentiated from all other geographically proximal species except *U. paulacushingae* by having a distinct comb on the retrolateral face of tarsus IV. Females can be distinguished from all other geographically proximal species by possessing simple, straight spermathecae. Males disperse from July to August.

##### Description of male holotype.

*Specimen preparation and condition*. Specimen preserved in 80% EtOH. Specimen in poor condition. *General coloration*. Carapace and chelicerae very dusky red 10R 2.5/2, legs dark reddish brown 2.5YR 2.5/4. Abdomen dark reddish brown 5YR 2.5/2. *Cephalothorax*. Carapace 6.04 long, 5.9 wide. Pars cephalica 3.91 long. Foveal groove procurved, 0.49 long, 1.22 wide. All eyes on moderate tubercle. AER procurved. PER procurved. Eye group 0.88 long, 1.42 wide, AME 0.38, PME 0.25, ALE 0.39, PLE 0.22. Sternum sparsely setose around outer 1/3, thicker anteriorly, STRl 3.48, STRw 3.69. Chelicerae with anterior tooth row comprising seven teeth, posterior margin with five teeth. Palpal endites with 27 long, thin cuspules over proximal half of endite face, lacking distal endite cuspules, ENDw 1.39, ENDl 2.51. Labium with six long, thin cuspules, LBw 1.44, LBl 0.99. Rastellum with many teeth on process. Abdomen setose. *Legs*. F1 5.73; F1w 1.42; P1 2.79; Ti1 3.86; Mt1 2.96; Tr1 1.23; F3 4.1; F3w 1.63; P3 2.17; Ti3 2.45; Sd3 1.32; Mt3 2.4; Tr3 1.62; F4 5.25; F4w 1.58; P4 2.5; Ti4 3.46; Mt4 3.87; Tr4 1.75. Retrolateral face of tarsus IV with proximal paired setae and with comb of spinules over distal half. Leg I spination pattern: TSp 4, TSpv 8, TSrd 0, TSr 0, TSrv 21, MtSp 4, MtSr 9, TrSp 4, TrSr 9. *Pedipalps*. PTl 3.17, PTw 1.25, Bl 2.78. Embolus evenly curved.

##### Varia tion, males

**(n = 17).**CL 4.04–7.65, 5.92±0.26; CW 4.1–7.5, 5.83±0.23; Cap 2.76–5.11, 3.93±0.17; ENDl 0.59–1.07, 0.8±0.04; ENDw 1.09–1.84, 1.39±0.05; STRl 2.56–4.41, 3.44±0.14; STRw 2.46–4.51, 3.47±0.14; LBl 0.67–1.2, 0.95±0.04; LBw 0.92–1.56, 1.29±0.05; F1 4.18–6.85, 5.53±0.18; F1w 0.84–1.87, 1.41±0.07; P1 1.97–3.46, 2.73±0.1; Ti1 2.79–4.51, 3.7±0.12; Mt1 1.94–2.99, 2.59±0.09; Tr1 0.99–3.8, 1.36±0.16; F3 2.94–4.92, 3.98±0.14; F3w 1.18–2.22, 1.63±0.07; P3 1.48–2.69, 2.1±0.09; Ti3 1.74–3.12, 2.41±0.1; Mt3 1.77–2.69, 2.27±0.07; Tr3 1.23–2.15, 1.73±0.07; F4 3.75–6.23, 5.16±0.19; F4w 1.1–2.64, 1.63±0.09; P4 1.72–3.12, 2.44±0.09; Ti4 2.53–4.18, 3.48±0.12; Mt4 1.76–4.37, 3.58±0.16; Tr4 1.19–2.49, 1.87±0.08; TSp 0–10, 4.76±0.66; TSpv 0–17, 7.59±0.88; TSr 0–7, 0.65±0.41; TSrv 15–39, 24.06±1.32; PTl 2.53–3.93, 3.24±0.1; PTw 0.95–1.55, 1.25±0.04; BL 1.97–3.18, 2.63±0.08.

##### Description of female paratype.

*Specimen preparation and condition*. Specimen preserved in 80% EtOH. Spermathecae removed, cleared, in vial with specimen. Left leg IV Mt-Tr and abdomen removed, in vial with specimen. *General coloration*. Carapace, chelicerae, and legs dark reddish brown 5YR 3/4. Abdomen very dark brown 7.5YR 2.5/3, spinnerets dark yellowish brown 10YR 4/6. *Cephalothorax*. Carapace 8.08 long, 7.93 wide. Pars cephalica 5.55 long. Foveal groove procurved, 0.65 long, 1.7 wide. Eye tubercle low under ME. AER procurved PER straight. Eye group 1.0 long, 1.87 wide, AME 0.44, PME 0.33, ALE 0.56, PLE 0.34. Sternum with posterior fringe, sparsely setose anteriorly STRl 5.01, STRw 5.28. Chelicerae with anterior row comprising eight teeth, posterior margin with six teeth. Palpal endites with 40 cuspules spread across proximal half of endite and 40 cuspules distally, equal in size to proximal cuspules anteriorly, smaller posteriorly, ENDw 2.04, ENDl 3.22. Labium with five cuspules, LBw 1.99, LBl 1.35. Rastellum with many strong spines on process and up cheliceral face. *Abdomen*. Evenly setose with pale speckles creating light chevrons at apodemes. *Legs*. F1 5.32; F1w 1.78; P1 3.31; Ti1 3.01; Mt1 2.27; Tr1 1.04; F3 4.37; F3w 2.25; P3 2.88; Ti3 2.33, Sd3 1.55; Mt3 2.2; Tr3 1.86; F4 5.5; F4w 2.31; P4 3.11; Ti4 3.15; Mt4 3.39; Tr4 1.89. Retrolateral face tarsus IV with defined comb over length of tarsus with proximal cluster of spinules. *Pedipalps*. PF 4.59, PP 2.62, PTi 2.93, PTr 2.78. Spermathecae simple with medial tilt, bulbs facing medioventrally.

##### Variation, females

**(n = 10)**CL 6.23–9.72, 7.9±0.32; CW 6.23–9.06, 7.51±0.31; Cap 4.18–6.78, 5.54±0.24; ENDl 0.92–1.2, 1.05±0.03; ENDw 1.55–2.2, 1.83±0.06; STRl 3.83–5.7, 4.77±0.2; STRw 4.02–6.12, 5±0.24; LBl 1–1.62, 1.32±0.06; LBw 1.39–2.36, 1.83±0.09; F1 4.14–6.27, 5.11±0.21; F1w 1.55–2.27, 1.89±0.08; P1 2.71–4, 3.28±0.15; Ti1 2.49–3.98, 3.12±0.15; Mt1 1.91–2.92, 2.34±0.12; Tr1 0.98–1.63, 1.21±0.07; F3 3.61–5.25, 4.36±0.17; F3w 1.88–2.78, 2.28±0.1; P3 2.31–3.54, 2.84±0.13; Ti3 2.09–3.17, 2.54±0.12; Mt3 1.75–2.62, 2.05±0.09; Tr3 1.52–2.18, 1.78±0.08; F4 4.58–6.66, 5.46±0.22; F4w 1.93–2.83, 2.31±0.1; P4 2.55–3.79, 3.1±0.13; Ti4 2.67–3.64, 3.11±0.12; Mt4 2.75–3.87, 3.22±0.13; Tr4 1.41–2.27, 1.8±0.09; PF 4.06–5.59, 4.66±0.17; PP 2.29–3.27, 2.73±0.1; PTi 2.34–3.65, 2.92±0.14; PTr 2.15–3.26, 2.6±0.11.

##### Material examined.

**United States: Arizona: Cochise Co**: 5 mi W of Portal, 31.9142 -109.226^5^, 1907 m a.s.l. (UMM0165, 10–20.vii.1955, 1♂, WJ Gertsch, AMNH); 5 mi W Portal, Southwestern Research Station, 31.8842 -109.206^5^, 1645 m a.s.l. (UMM0348, 1.viii.1955, 1♂, WJ Gertsch, AMNH); (UMM0380, 10.ix.1970, 1♂, WJ Gertsch, AMNH); (UMM0351, 10.viii.1956, 1♂, E Ordway, AMNH); (UMM0356, 10–20.vii.1955, 1♂, WJ Gertsch, AMNH); (UMM0572, 1♀, AMNH); (UMM0254, 12.vii.1992, 1♂, S Lingafelter, AMNH); (UMM0350, 15–20.viii.1968, 1♂, V Roth, AMNH); (UMM0358, 19.viii.1965, 1♂, Mrs Anderson, AMNH); (UMM0266, 19.viii.1988, 1♂, 1♀, J Rozen, E Brewster, AMNH); (UMM0360, 20.vii.1962, 1♂, WJ Gertsch, AMNH); (UMM0470, 20.vii.1976, 1♂, S Johnson, AMNH); (UMM0344, 21.viii.1966, 1♂, B Rozen, AMNH); (UMM0324, 23.viii.1974, 1♂, AMNH); (UMM0359, 25.viii.1966, 1♂, Rozens, AMNH); (UMM0395, 26.viii.1955, 1♂, WJ Gertsch, AMNH); (UMM0550, 29.vii.1976, 1♂, S Johnson, AMNH); (UMM0385, 30.viii.2003, 1♂, JG Rozch, AMNH); (UMM0386, 31.vii.1956, 1♂, E Ordway, AMNH); (UMM0612, 31.vii.1968, 1♀, SE Roth, AMNH); (UMM0618, 7.vii.1966, 1♂, B Nockumas, AMNH); (UMM0595, 7.vii.1972, 1juv, Riechart, AMNH); (UMM0361, 7.viii.1963, 1♂, K Muma, AMNH); (UMM0394, iv.1956, 1♀, WJ Gertsch, E Ordway, AMNH); 5 mi W Portal, Southwestern Research Station, 31.8842 -109.206^5^, 1645 m a.s.l. (UMM0661, 1♂, AMNH); Chiricahua Mountains, 31.8831 -109.2829^6^, 2751 m a.s.l. (UMM0387, 15.vii.1961, 1♂, DJ Knull, JN Knull, AMNH); (UMM0355, vii-viii.1972, 1♂, JAL Cooke, AMNH); Chiricahua Mountains, Southwestern Research Station, 31.8842 -109.206^5^, 1645 m a.s.l. (UMM0336, 10.ix.1979, 1♂, V Roth, AMNH); (UMM0276, 14.ix.1967, 1♂, AMNH); (UMM0375, 14.viii.1969, 1♂, AMNH); (UMM0337, 17.viii.1981, 1♂, AMNH); (UMM0376, 18.viii.1982, 1♂, AMNH); (UMM0357, 2.viii.1971, 1♂, AMNH); (UMM0372, 21.vii.1981, 1♂, AMNH); (UMM0378, 25.viii.1974, 1♂, AMNH); (UMM0352, 28.vii.1970, 1♂, AMNH); (UMM0342, 28.viii.1979, 1♂, AMNH); (UMM0379, 31.viii.1968, 1♂, AMNH); (UMM0374, 4.ix.1967, 1♂, AMNH); (UMM0373, 8.vii.1984, 1♂, AMNH); (UMM0338, 22.vii.1971, 1♂, T Haley, AMNH); (UMM0393, 22–24.viii.1980, 1♂, D Hawks, AMNH); (UMM0343, 23.vii.1964, 1♂, SE Roth, AMNH); (UMM0368, 5.ix.1978, 1♀, D Hawks, AMNH); (UMM0370, 7.viii.1967, 1♂, D Rich , AMNH); Chiricahua Mountains, Southwestern Research Station, 31.8842 -109.206^5^, 1645 m a.s.l. (UMM0391, 30.vii.1968, 1♂, AMNH); Huachuca Mountains, Miller Canyon, 31.4177 -110.2775^6^, 2059 m a.s.l. (UMM0185, 27.ii.1972, 1♀, DB Richman, MS Richman, UFMNH); Huachucha Mountains, Ramsey Canyon, 31.4486 -110.3087^4^, 1710 m a.s.l. (UMM0346, 20–25.viii.1932, 1♂, WS Creighton, AMNH); 1mi S Portal, 31.899 -109.1492^4^, 1833m (UMM0620, 23.viii.1966, 1♂, AMNH); Portal, 31.9125 -109.1421^5^, 1457 m a.s.l. (UMM0690, 15.vii.1965, 1♂, M Muma, AMNH); 31.9125 -109.1421^6^, 1457 m a.s.l. (UMM0688, 28.ix.1983, 1♂, M Muma, AMNH); (UMM0390, 15.vii.1965, 1♂, WJ Gertsch, AMNH); (UMM0349, 19.viii.1967, 1♂, AMNH); (UMM0389, 25.vii.1970, 1♂, AMNH); (UMM0354, vii.1968, 1♂, AMNH); (UMM0371, 20.vii.1980, 1♂, V Roth, H Joboff, AMNH); (UMM0549, 22.viii.1970, 1♂, L Anderson, AMNH); (UMM0317, 24.viii.1964, 1♂, J Rozen, AMNH); (UMM0353, 30.vi.1967, 1♂, T Smell, AMNH); Portal, Jensen’s Store, 31.9137 -109.1414^6^, 1453 m a.s.l. (UMM0347, 1.viii.1971, 1♂, S Roth, AMNH); 5mi W Portal, Southwestern Research Station, 31.8842 -109.206^5^, 1645 m a.s.l. (UMM0206, 13–22.viii.1972, 1♂, N Platnick, MCZ); Coconino Co: Jct I-40 and Meteor Crater Road, 35.1105 -111.0316^4^, 1639 m a.s.l. (UMM849, 1♂, E Riley, M Yoder, TAMU); **Graham Co**: Galiura Mountains, 10 mi W Sunset, 32.6011 -110.3533^6^, 1753 m a.s.l. (UMM0181, 18.vii.1956, 1juv, MJ Westfall, UFMNH); Pinaleno Mountains, 32.641 -109.8436^6^, 2824 m a.s.l. (UMM0153, 15.vii.1917, 1♀, CUNHC); **Maricopa Co**: Phoenix, South Mountain, 33.3304 -112.092^5^, 560 m a.s.l. (UMM0417, 20.viii.1966, 1♂, SC Williams, AMNH); **Pima Co**: Santa Catalina Mountains, Bear Canyon Picnic Area, 32.3154 -110.791^4^, 934 m a.s.l. (UMM0455, 3.vii.1961, 1♂, FG Werner, AMNH); Santa Catalina Mountains, Molina Canyon, 31.3022 -110.7178^5^, 871 m a.s.l. (UMM0168, 25.vii.1970, 1♂, K Stephens, AMNH); Tucson, 32.222 -110.9262^6^, 759 m a.s.l. (UMM0157, 15.vii.1953, 1♂, GM Bradt, AMNH); (UMM0616, 15.vii.1953, 1♂, GM Bradt, AMNH); (UMM0438, 9.v.1909, 1♀, G Bradt, AMNH); (UMM0468, vii.1950, 1♂, RE Crandall, AMNH); **Pinal Co**: San Tan Valley, 33.1788 -111.4942^1^, 474 m a.s.l. (UMM0662, 7.ii.2016, 1♀, TA Cota, BME); (UMM0664, 1♀, BME); (UMM0665, 1♀, BME); **New Mexico: Grant Co**: Fort Bayard, 32.7961 -108.1502^5^, 1867 m a.s.l. (UMM0689, 20.viii.1976, 1♂, M Muma, AMNH); **Hidalgo Co**: off Hwy #9, 7 mi W Aimes, 31.9406 -108.7077^7^, 1372 m a.s.l. (UMM0508, 1♂, B Tomberlin, T Snell, AMNH).

#### Ummidia timcotai sp. nov.

Taxon classificationAnimaliaAraneaeHalonoproctidae

FAFDB716-8F53-5649-B316-0356217DAEBA

http://zoobank.org/642975B5-66C0-44F7-BD83-40D0E3214E98

**Figure 32.**
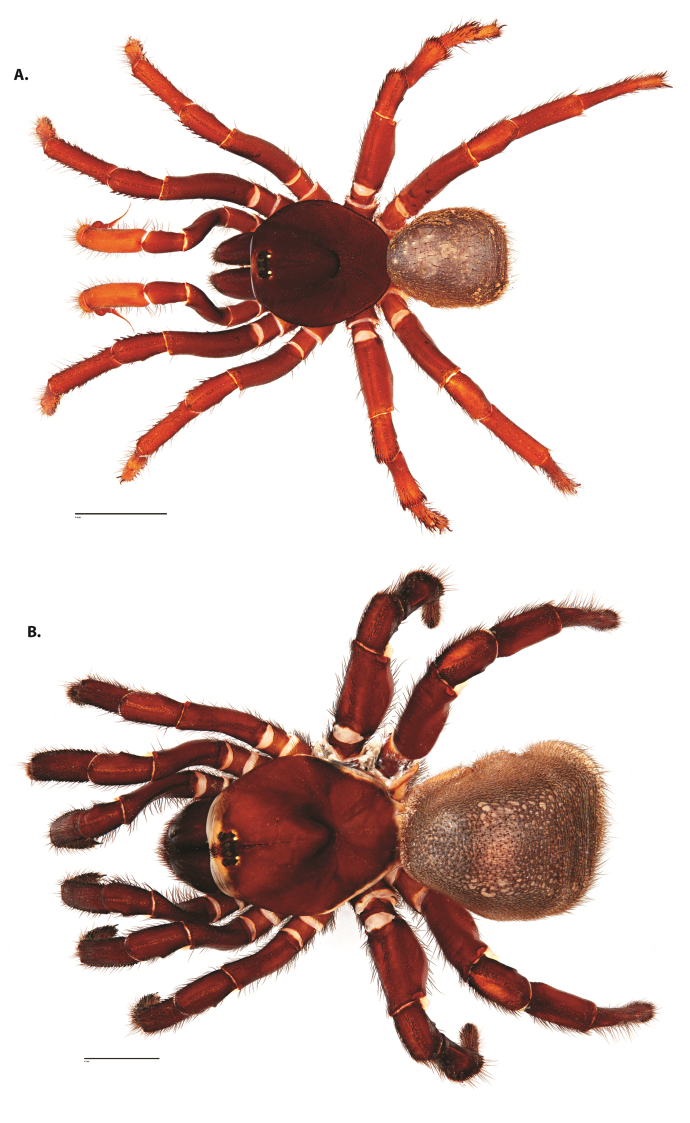
*Ummidia timcotai* sp. nov. from Gila Co., Arizona **A** male habitus illustration UMM161 **B** female habitus illustration UMM666. Scale bars: 4.0 mm.

**Figure 33.**
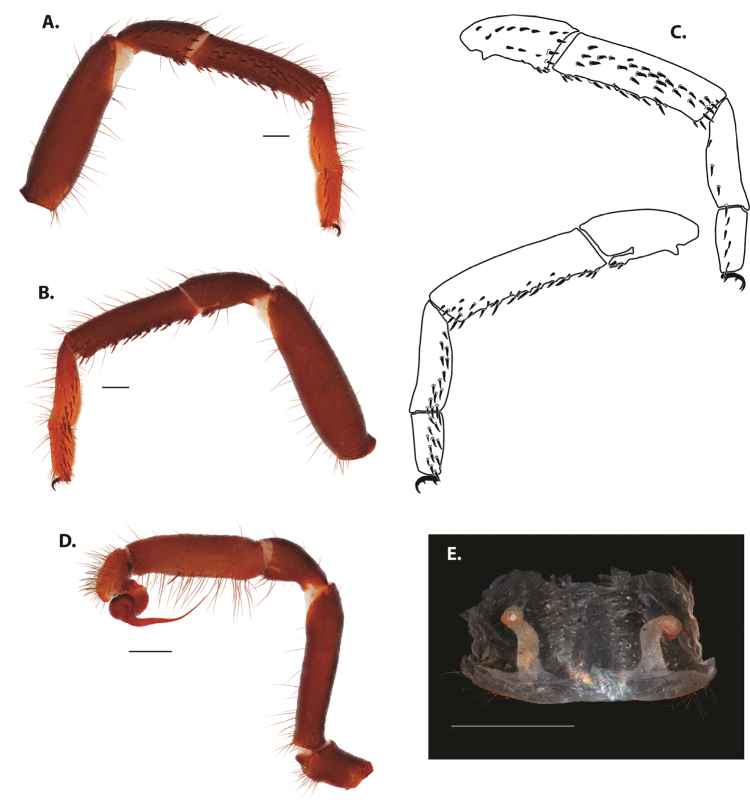
*Ummidia timcotai* sp. nov. from Gila Co., Arizona **A–D** male holotype (UMM161) **A** prolateral aspect, leg I **B** retrolateral aspect, leg I **C** line drawings, leg I prolateral and retrolateral aspects **D** retrolateral aspect, pedipalp **E** cleared spermathecae female paratype (UMM666). Scale bars: 1.0 mm.

Figs 32 , 33 , Map 4

##### Type material.

HOLOTYPE ♂ (UMM161) from Globe, Gila County, Arizona, United States, 33.3940 -110.7864^6^, 1072 m a.s.l., 17.vii.1967, coll. MA Cazier, AMNH. PARATYPE: 1 ♀ (UMM666) from San Tan Valley, Pinal County, Arizona, United States, 33.1788 -111.4942^1^, 474 m a.s.l., 8.ii.2016, coll. TA Cota, BME.

##### Etymology.

The specific epithet is a patronym named for Timothy Cota who has collected many specimens used in this revision to include the female paratype of this species and has studied the group’s natural history for many years.

##### Diagnosis.

*Ummidia timcotai* can be differentiated from *U. rosillos* by the lack of a pale dorsal heart patch and from all geographically proximal species except *U. rosillos* by the presences of a brush on the retrolateral face of tarsus IV. Males can be differentiated from *U. mercedesburnsae*, *U. paulacushingae*, *U. waunekaae*, and *U. gertschi* by having a sinuous embolus and from all geographically proximal species by tibia I being very spinose. Females can be differentiated from *U. gertschi*, *U. modesta*, and *U. mercedesburnsae* by having spermathecae that curve laterally. Males disperse from July to August.

##### Description of male holotype.

*Specimen preparation and condition*. Specimen preserved in 80% EtOH. Left palp and leg I removed, in vial with specimen. *General coloration*. Carapace and chelicerae very dusky red 10R 2.5/2, legs dark reddish brown 2.5YR 2.5/4. Mt and Tr yellowish red 5YR 4/6. Abdomen dark reddish gray 2.5YR 3/1. *Cephalothorax*. Carapace 6.1 long, 5.72 wide. Pars cephalica 3.97 long. Foveal groove procurved, 0.41 long, 1.31 wide. AME on low tubercle. AER procurved. PER straight. Eye group 0.71 long, 1.33 wide, AME 0.31, PME 0.19, ALE 0.41, PLE 0.2. Sternum sparsely setose, STRl 3.5, STRw 3.09. Chelicerae with anterior tooth row comprising eight teeth, posterior margin with eight teeth. Palpal endites with 14 cuspules spread over proximal half of endite face, lacking distal endite cuspules, ENDw 1.34, ENDl 2.31. Labium with five cuspules, LBw 1.2, LBl 0.91. Rastellum with many spines on process. Abdomen setose with dorsal opalescence. *Legs*. F1 4.96; F1w 1.37; P1 2.45; Ti1 3.41; Mt1 2.25; Tr1 1.26; F3 3.82; F3w 1.6; P3 2.08; Ti3 2.5; Sd3 1.41; Mt3 2.17; Tr3 1.7; F4 5.04; F4w 1.49; P4 2.33; Ti4 3.43; Mt4 3.27; Tr4 1.79. Retrolateral face of tarsus IV with loose brush. Leg I spination pattern: TSp 26, TSpv 4, TSrd 0, TSr 0, TSrv 30, MtSp 4, MtSr 14, TrSp 6, TrSr 12. *Pedipalps*. PTl 3.13, PTw 1.04, Bl 2.64. Embolus relatively long and sinuous.

##### Variation, males

**(n = 21).**CL 4.34–6.82, 5.75±0.13; CW 4.25–7.73, 5.73±0.17; Cap 2.89–4.66, 3.78±0.1; ENDl 0.51–0.96, 0.72±0.03; ENDw 0.96–1.63, 1.31±0.04; STRl 2.75–3.98, 3.37±0.08; STRw 2.44–4.25, 3.29±0.11; LBl 0.69–1.79, 0.96±0.05; LBw 0.89–1.65, 1.2±0.04; F1 4.35–6.33, 5.33±0.11; F1w 0.92–1.68, 1.37±0.04; P1 1.88–3.18, 2.56±0.07; Ti1 3.01–4.19, 3.66±0.06; Mt1 1.92–3.05, 2.46±0.05; Tr1 1.13–1.49, 1.31±0.02; F3 3.27–4.64, 4.07±0.07; F3w 1.25–1.98, 1.63±0.04; P3 1.62–2.51, 2.13±0.05; Ti3 2.01–2.96, 2.52±0.05; Mt3 1.96–2.83, 2.39±0.04; Tr3 1.36–2.21, 1.81±0.04; F4 4.14–6.16, 5.29±0.1; F4w 1.14–2.1, 1.56±0.05; P4 1.78–2.81, 2.37±0.06; Ti4 2.82–3.98, 3.56±0.06; Mt4 3.04–4.27, 3.65±0.07; Tr4 1.53–2.3, 1.96±0.05; TSp 0–23, 9.9±1.44; TSpv 0–28, 4.71±1.54; TSr 0–12, 0.9±0.61; TSrv 9–33, 18.29±1.46; PTl 2.71–3.61, 3.27±0.05; PTw 0.89–1.36, 1.12±0.03; BL 2.42–3.01, 2.65±0.04.

##### Description of female paratype.

*Specimen preparation and condition*. Specimen preserved in 80% EtOH. Spermathecae removed and cleared, in vial with specimen. Right leg III and IV in 100% EtOH. *General coloration*. Carapace, chelicerae, and legs dark reddish brown 2.5YR 2.5/3. Abdomen black 7.5YR 2.5/1, spinnerets yellowish brown 10YR 5/8. *Cephalothorax*. Carapace 10.08 long, 9.04 wide. Pars cephalica 6.75 long. Foveal groove procurved, 0.91 long, 2 wide. Eye tubercle low under ME only. AER procurved. PER straight. Eye group 1.04 long, 2.01 wide, AME 0.45, PME 0.25, ALE 0.49, PLE 0.36. Sternum sparsely setose, STRl 6.19, STRw 5.38. Chelicerae with anterior row comprising five teeth, posterior margin with six teeth. Palpal endites with 24 cuspules spread across proximal half of endite and six reduced cuspules distally, ENDw 2.19, ENDl 3.71. Labium with six cuspules, LBw 2.13, LBl 1.43. Rastellum with many strong spines on process and up cheliceral face. *Abdomen*. Evenly setose with slight dorsal opalescence. *Legs*. F1 5.63; F1w 2.08; P1 3.69; Ti1 3.77; Mt1 2.58; Tr1 1.4; F3 5.29; F3w 2.7; P3 3.37; Ti3 3.04, Sd3 1.66; Mt3 2.04; Tr3 1.87; F4 6.8; F4w 2.7; P4 3.63; Ti4 3.89; Mt4 3.6; Tr4 2.01. Retrolateral face tarsus IV with brush of spinules, prolaterally and ventrally very setose. *Pedipalps*. PF 5.68, PP 3.29, PTi 3.87, PTr 3.12. Spermathecae curved laterally and then coiled dorsally, bulbs facing dorsally.

##### Variation, females

**(n = 8).**CL 7.57–10.8, 9.6±0.43; CW 7.14–10.35, 8.85±0.4; Cap 4.84–6.95, 6.37±0.29; ENDl 0.88–1.16, 1.03±0.03; ENDw 1.59–2.91, 2.05±0.14; STRl 4.75–6.75, 5.96±0.26; STRw 4.29–6.33, 5.38±0.27; LBl 1.16–1.67, 1.43±0.06; LBw 1.56–2.44, 2.04±0.11; F1 4.41–6.49, 5.44±0.23; F1w 1.7–2.38, 2.13±0.07; P1 2.76–4.11, 3.52±0.15; Ti1 2.99–4.23, 3.58±0.15; Mt1 1.93–2.95, 2.47±0.12; Tr1 1.07–1.48, 1.32±0.05; F3 4.36–5.51, 5.03±0.15; F3w 2.17–3.06, 2.71±0.11; P3 2.56–3.95, 3.28±0.15; Ti3 2.39–3.31, 2.91±0.13; Mt3 1.56–2.51, 2.05±0.11; Tr3 1.38–1.9, 1.7±0.07; F4 5.46–7.41, 6.42±0.23; F4w 2.34–3.19, 2.76±0.1; P4 2.81–4.13, 3.49±0.16; Ti4 3.09–4.18, 3.61±0.14; Mt4 3.11–4.27, 3.6±0.14; Tr4 1.47–2.43, 1.84±0.1; PF 4.52–5.86, 5.26±0.19; PP 2.57–3.81, 3.08±0.15; PTi 2.96–4.07, 3.53±0.14; PTr 2.43–3.29, 2.95±0.1.

##### Material examined.

**United States: Arizona**: 34.0529 -111.0937^8^, 1126 m a.s.l. (UMM0552, 1♂, RV Chamberlin, AMNH); **Cochise Co**: Bisbee, 31.4489 -109.9283^6^, 1700 m a.s.l. (UMM0403, 24.v.1909, 1♀, CL Edmunson, AMNH); **Gila Co**: Globe, 33.394 -110.7864^6^, 1072 m a.s.l. (UMM0161, 17.vii.1967, 1♂, MA Cazier, AMNH); **Madera Co**: Santa Rita Mtns, 31.7251 -110.8799^5^, 1499 m a.s.l. (UMM0339, 25.vii-5.viii.1965, 1♂, RH Crandall, AMNH); (UMM0571, 1♂, AMNH); **Maricopa Co**: 2.5 mi N of Sentinel, jct Hwy I-8 and road to Agua Caliente, 32.9142 -113.2852^4^, 173 m a.s.l. (UMM0778, 20.xi.1989, 1♀, W Icenogle, T Prentice, AMNH); **Pima Co**: 100 yds NW entrance to Organ Pipe National Monument, 32.2079 -112.7639^5^, 534 m a.s.l. (UMM0779, 19.xi.1989, 1♀, W Icenogle, T Prentice, AMNH); Black Pt Picnic Area, Santa Rita Mtns, Madera Canyon, 31.7251 -110.8799^5^, 1499 m a.s.l. (UMM0507, 15.vii.1971, 1♂, DB Ekkens, AMNH); Catalina Mtns, 32.4732 -110.7817^7^, 1827 m a.s.l. (UMM0594, 23.vii.1917, 1♂, AMNH); Tucson, 32.222 -110.9262^6^, 759 m a.s.l. (UMM0527, 3.viii.1950, 1♀, GM Bradt, AMNH); (UMM0383, 1♂, O Bryant, AMNH); Tucson, between Deaf and Dumb School and golf course, 32.222 -110.9262^6^, 759 m a.s.l. (UMM0450, 16.xi.1946, 1♀, GD Morris, AMNH); **Pinal Co**: Florence, 32.9818 -111.362^1^, 503 m a.s.l. (UMM0667, 13.ii.2016, 1♀, TA Cota, BME); San Tan Valley, 33.1788 -111.4942^1^, 474 m a.s.l. (UMM0663, 7.ii.2016, 1♀, TA Cota, BME); (UMM0666, 8.ii.2016, 1♀, BME); **Santa Cruz Co**: Elgin, 31.6597 -110.5253^6^, 1441 m a.s.l. (UMM0598, 17.vii.1948, 1♂, C Vaurie, P Vaurie, AMNH); Madera Canyon, 31.7251 -110.8799^5^, 1499 m a.s.l. (UMM0539, 14.vii.1971, 1♂, M Van Buskirk, A Jung, AMNH); Madera Canyon, 31.7187 -110.8896^5^, 1905 m a.s.l. (UMM0726, 24.vii.1985, 1♂, PK Lago, MEM); Pena Blanca Lake, 31.3887 -111.0936^1^, 1184 m a.s.l. (UMM0741, 23.vii.2009, 1♂, P Bollinger, BME); (AUMS019104, 8.viii.2016, 1♂, A Jeon, BME); Santa Rita Mtns, Madera Canyon, 31.7251 -110.8799^5^, 1499 m a.s.l. (UMM0503, 14.vii.1971, 1♂, M Van Buskirk, A Jung, AMNH); (UMM0495, 17.vii.1971, 1♂, DG Marqua, AMNH); (UMM0505, 19.vii.1971, 1♂, AMNH); (UMM0627, 1♂, AMNH); (UMM0628, 1♂, AMNH); (UMM0501, 20.vii.1971, 1♂, AMNH); (UMM0366, 8.vii.1971, 1♂, AMNH); (UMM0645, 4.viii.1956, 1♂, D Verity, AMNH); Santa Rita Mtns, Madera Canyon, 31.7251 -110.8799^5^, 1499 m a.s.l. (UMM0566, 1♂, AMNH).

#### Ummidia gabrieli sp. nov.

Taxon classificationAnimaliaAraneaeHalonoproctidae

A31E2B13-1A7F-595D-925D-216C52F505E3

http://zoobank.org/74AD7E87-7007-41AD-85BE-17D0896526FA

**Figure 34.**
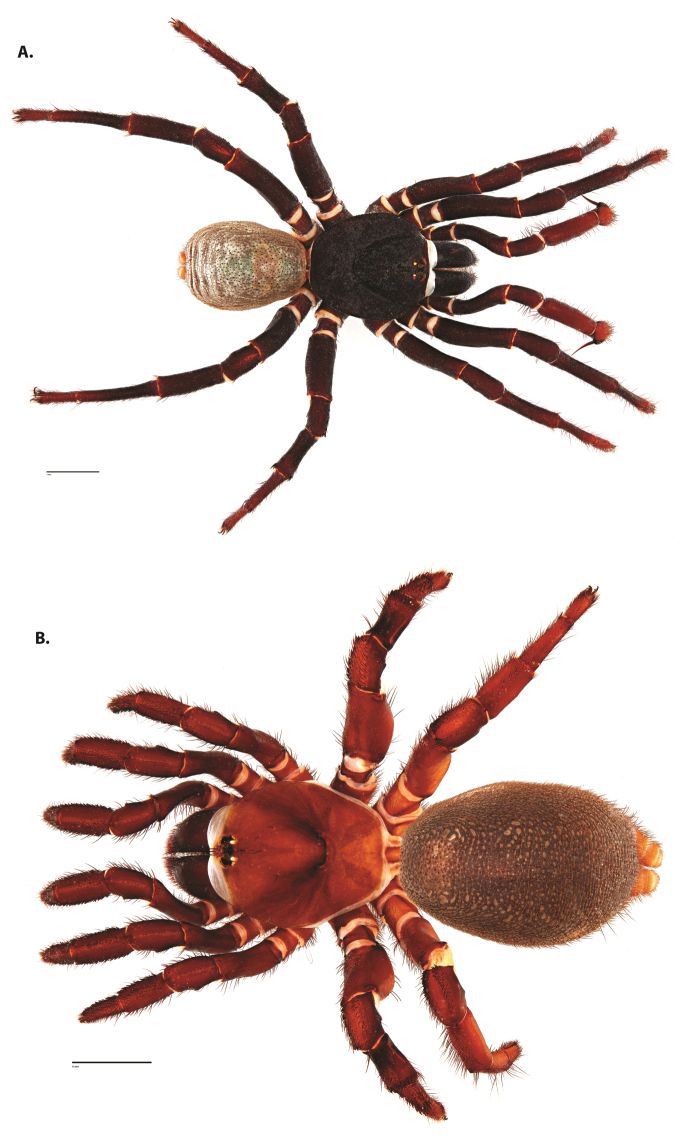
*Ummidia gabrieli* sp. nov. from Baja California Sur, Mexico. **A** male habitus illustration UMM341 **B** female habitus illustration UMM392. Scale bars: 4.0 mm.

**Figure 35.**
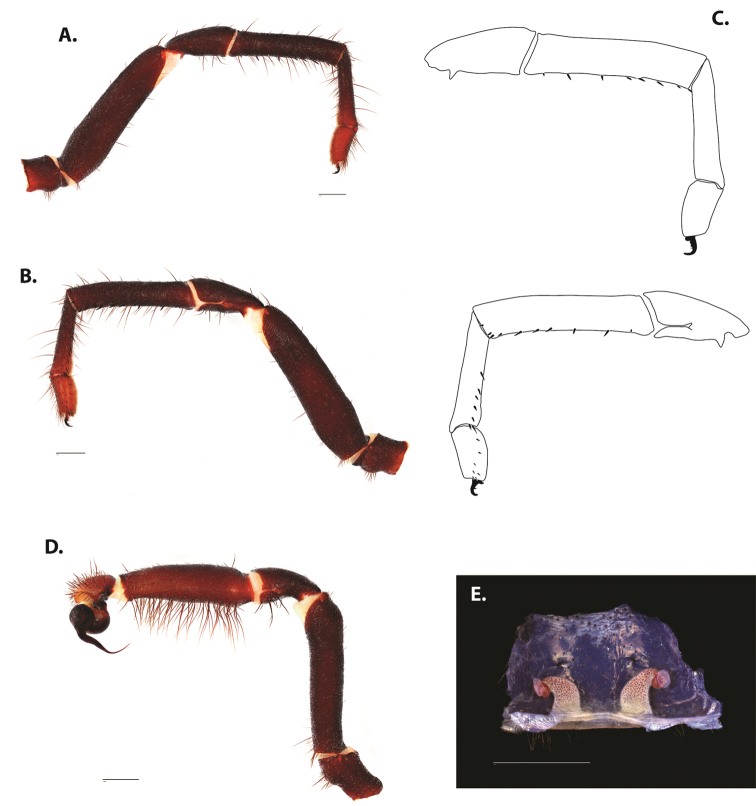
*Ummidia gabrieli* sp. nov. from Baja California Sur, Mexico **A–D** male holotype (UMM341) **A** prolateral aspect, leg I **B** retrolateral aspect, leg I **C** line drawings, leg I prolateral and retrolateral aspects **D** retrolateral aspect, pedipalp **E** cleared spermathecae female paratype (UMM392). Scale bars: 1.0 mm.

Figs 34 , 35 , Map 4

##### Type material.

HOLOTYPE ♂ (UMM341) from 8 miles southeast of La Paz, La Paz, Baja California Sur, Mexico, 24.0345 -110.227^4^, 304 m a.s.l., 13.x.1968, coll. EL Sleeper, FJ Moore, AMNH. PARATYPE: 1 ♀ (UMM392) from 5 miles north of San Ignacio, Mulegé, Baja California Sur, Mexico, 26.735 -111.7012^5^, 210 m a.s.l., 20.i.1965, coll. V. Roth, AMNH.

##### Etymology.

The specific epithet is a patronym in honor of musician and human rights activist Peter Gabriel.

##### Diagnosis.

*Ummidia gabrieli* are relatively large spiders (males CL 8.6–10.6, other species CL 4–7.5) and can be differentiated from males of all other geographically proximal species by the lack of prolateral spines on metatarsus and tibia I, having a palpal tibia that is > 3 ´ long as wide, by having tarsi which are relatively short and tapered distally, and by the presence of a dorsal opalescence on the abdomen. Males can be differentiated from *U. mercedesburnsae*, *U. paulacushingae*, *U. waunekaae*, and *U. gertschi* by having a sinuous embolus. Females can be differentiated from *U. gertschi*, *U. modesta*, and *U. mercedesburnsae* by having spermathecae that curve laterally. Females can be differentiated from *U. pesiou* by having significantly fewer endite cuspules (28 vs 59) and having rastellar spines not extending up cheliceral face, and from *U. pustulosa* by having the sternum roughly as wide as long.

##### Description of male holotype.

*Specimen preparation and condition*. Specimen preserved in 80% EtOH. Left palp and leg I removed, in vial with specimen. *General coloration*. Carapace, chelicerae, and legs reddish black 2.5YR 2.5/1. Abdomen black 2.5Y 2.5/1. *Cephalothorax*. Carapace 9.0 long, 8.39 wide. Pars cephalica 5.49 long. Foveal groove procurved, 0.61 long, 1.71 wide. All eyes on defined moderate tubercle. AER procurved. PER straight. Eye group 1.09 long, 1.75 wide, AME 0.47, PME 0.31, ALE 0.69, PLE 0.22. Sternum sparsely setose, STRl 5.09, STRw 4.41. Chelicerae with anterior tooth row comprising eight teeth, posterior margin with eight8 teeth. Palpal endites with eleven small, non-hastate cuspules over proximal 1/4 of endite face, lacking distal endite cuspules, ENDw 1.89, ENDl 3.35. Labium with four cuspules, LBw 1.77, LBl 1.39. Rastellum with many small, rounded teeth on process. Abdomen setose. *Legs*. F1 8.04; F1w 1.94; P1 3.59; Ti1 5.82; Mt1 3.92; Tr1 1.79; F3 6.11; F3w 2.32; P3 3.23; Ti3 3.53; Sd3 2.71; Mt3 3.79; Tr3 2.59; F4 7.87; F4w 2.22; P4 3.44; Ti4 5.06; Mt4 6; Tr4 2.67. Retrolateral face of tarsus IV lacking distinct comb or brush, but very setose ventrally. Leg I spination pattern: TSp 0, TSpv 0, TSrd 0, TSr 0, TSrv 11, MtSp 0, MtSr 7, TrSp 0, TrSr 7. *Pedipalps*. PTl 4.84, PTw 1.51, Bl 3.82. Embolus relatively long; sinuous.

##### Variation, males

**(n = 7).**CL 8.59–10.61, 9.24±0.26; CW 7.84–9.92, 8.5±0.25; Cap 5.28–6.41, 5.65±0.14; ENDl 0.91–1.09, 0.98±0.02; ENDw 1.65–1.88, 1.75±0.04; STRl 4.41–5.97, 5.17±0.17; STRw 4.27–5.08, 4.52±0.1; LBl 1.15–1.6, 1.38±0.05; LBw 1.46–4.77, 2.13±0.44; F1 6.92–9.06, 7.86±0.24; F1w 1.75–2.32, 2±0.07; P1 3.21–4.41, 3.77±0.14; Ti1 4.69–6.52, 5.7±0.21; Mt1 3.31–4.57, 3.89±0.14; Tr1 1.62–2.01, 1.82±0.05; F3 5.2–6.84, 6.02±0.19; F3w 2.18–2.74, 2.37±0.07; P3 2.78–3.65, 3.18±0.1; Ti3 3.36–4, 3.61±0.08; Mt3 3.03–4.34, 3.7±0.16; Tr3 2.23–2.96, 2.52±0.09; F4 7.01–8.91, 7.77±0.23; F4w 1.92–2.48, 2.16±0.06; P4 3.1–3.82, 3.43±0.08; Ti4 4.32–5.65, 5.07±0.15; Mt4 4.74–6.23, 5.56±0.19; Tr4 2.36–3.06, 2.63±0.1; TSp 0–0, 0±0; TSpv 0–3, 0.86±0.46; TSr 0–0, 0±0; TSrv 5–17, 8.71±1.63; PTl 3.91–5.48, 4.83±0.19; PTw 1.45–1.62, 1.52±0.02; BL 3.6–4.31, 3.78±0.09.

##### Description of female paratype.

*Specimen preparation and condition*. Specimen preserved in 80% EtOH. Spermathecae removed, cleared, in vial with specimen. Right palp removed at patella, in vial with specimen. *General coloration*. Carapace, chelicerae, and legs dark reddish brown 5YR 3/1. Abdomen black 7.5YR 2.5/1, spinnerets dark yellowish brown 10YR 4/6. *Cephalothorax*. Carapace 8.25 long, 7.45 wide. Pars cephalica 5.48 long. Foveal groove procurved, 0.47 long, 1.67 wide. Eye tubercle low. AER procurved. PER recurved. Eye group 0.95 long, 1.63 wide, AME 0.35, PME 0.26, ALE 0.49, PLE 0.31. Sternum sparsely setose, STRl 4.81, STRw 4.25. Chelicerae with anterior row comprising seven teeth, posterior margin with seven teeth. Palpal endites with 18 cuspules spread across proximal half of endite and ten reduced cuspules distally, ENDw 1.97, ENDl 3.08. Labium with eight cuspules, LBw 1.75, LBl 1.23. Rastellum with many strong spines along cheliceral margin and on rastellar process. *Abdomen*. Evenly setose with pale speckles and slight dorsal opalescence. *Legs*. F1 4.62; F1w 1.77; P1 2.92; Ti1 2.84; Mt1 1.97; Tr1 0.95; F3 4.45; F3w 2.35; P3 2.94; Ti3 2.49, Sd3 1.63; Mt3 1.7; Tr3 1.54; F4 5.68; F4w 2.26; P4 2.99; Ti4 2.94; Mt4 2.94; Tr4 1.66. Retrolateral face tarsus IV with defined comb over length of tarsus. *Pedipalps*. PF 4.35, PP 2.5, PTi 2.6, PTr 2.16. Spermathecae curved laterally with distal coil, bulbs facing laterally.

##### Variation, females

**(n = 3).**CL 5.4–8.3, 7.32±0.96; CW 4.27–7.58, 6.43±1.08; Cap 3.55–5.56, 4.86±0.66; ENDl 0.65–1.11, 0.9±0.14; ENDw 0.88–1.64, 1.38±0.25; STRl 3.22–5.21, 4.41±0.61; STRw 2.6–4.63, 3.83±0.62; LBl 0.82–1.48, 1.18±0.19; LBw 1.15–1.75, 1.55±0.2; F1 2.94–5.32, 4.3±0.71; F1w 1.18–1.82, 1.59±0.21; P1 1.82–3.18, 2.64±0.42; Ti1 1.78–3.31, 2.64±0.45; Mt1 1.22–2.28, 1.82±0.32; Tr1 0.84–1.3, 1.03±0.14; F3 2.55–4.71, 3.9±0.68; F3w 1.29–2.4, 2.01±0.36; P3 1.66–2.94, 2.48±0.41; Ti3 1.46–2.51, 2.15±0.35; Mt3 1.1–2.01, 1.61±0.27; Tr3 1.2–1.63, 1.46±0.13; F4 3.31–5.68, 4.88±0.79; F4w 1.47–2.26, 1.97±0.25; P4 1.9–3.31, 2.73±0.43; Ti4 1.84–3.14, 2.64±0.4; Mt4 1.78–3.4, 2.71±0.48; Tr4 1.14–1.66, 1.46±0.16; PF 2.71–5, 4.02±0.68; PP 1.62–2.89, 2.34±0.38; PTi 1.81–3.43, 2.62±0.47; PTr 1.31–2.8, 2.09±0.43.

##### Material examined.

**Mexico: Baja California: Sur**: 22 mi NE of Santo Domingo, 28.5223 -113.7724^6^, 786 m a.s.l. (UMM0155, 15.ii.1966, 1♂, V Roth, AMNH); 8 mi SE La Paz, 24.0345 -110.227^4^, 304 m a.s.l. (UMM0340, 13.x.1968, 1♂, EL Sleeper, FJ Moore, AMNH); (UMM0341, 1♂, AMNH); (UMM0596, 1♂, AMNH); (UMM0637, 1♂, AMNH); (UMM0400, 1♂, AMNH); El Triunfo watercourse, 23.8099 -110.1241^1^, 457 m a.s.l. (MY02696, 16.iii.2004, 1♀, M Hedin, BME); Hwy 286 (to San Juan de los Planes), 24.1003 -110.2692^1^, 110 m a.s.l. (MY02695, 17.iii.2004, 1♀, M Hedin, BME); near La Paz, 24.1263 -110.2935^7^, 11 m a.s.l. (UMM0687, vii.1990, 1♂, T Jackson, AMNH); 3 mi N Santa Anita, 23.2612 -109.7155^6^, 110 m a.s.l. (UMM0445, 1.vii.1968, 1♀, AMNH); 5 mi N San Ignacio (mission), 26.735 -111.7012^5^, 210 m a.s.l. (UMM0392, 20.i.1965, 1♀, V Roth, AMNH).

#### Ummidia pesiou sp. nov.

Taxon classificationAnimaliaAraneaeHalonoproctidae

AF1C3C7B-3B5A-5B9A-9968-35246E95BA9E

http://zoobank.org/E4DF1B16-B890-45AD-99A6-7FDDCD9BEA60

Fig. 36 , Map 4

##### Type material.

HOLOTYPE: 1 ♀ (UMM464, AMNH) from 16 miles east of Hermosillo, Sonora, Mexico, 29.0894 -110.6947^5^, 371 m a.s.l., 20.viii.1960, coll. Zweifel et. al., AMNH.

##### Etymology.

The specific epithet is a noun taken in apposition and is in reference to the original Yaqui name for Hermosillo.

##### Diagnosis.

*Ummidia pesiou* can be differentiated from all other geographically proximal species by having a well-developed rastellum with spines extending up the cheliceral face 3 ´ the length of the rastellar process. Females can be differentiated from *U. gabrieli* by having significantly more endite cuspules (59 vs 28) and from *U. pustulosa* by having the sternum roughly as wide as long.

**Figure 36.**
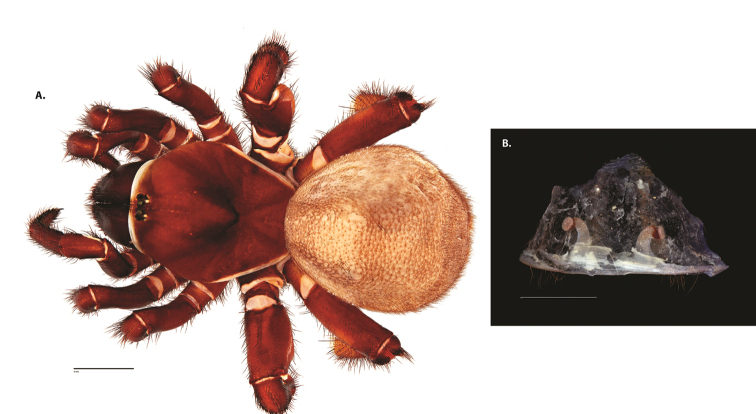
*Ummidia pesiou* sp. nov. female holotype specimen (UMM464) from Sonora, Mexico. **A** female habitus illustration **B** cleared spermathecae. Scale bars: 4.0 mm (**A**), 1.0 mm (**B**).

##### Description of female holotype.

*Specimen preparation and condition*. Specimen preserved in 80% EtOH. Spermathecae removed, cleared, in vial with specimen. Right leg II missing. Left legs IV and II removed, in vial with specimen. *General coloration*. Carapace, chelicerae, and legs dark reddish brown 2.5YR 2.5/3. Abdomen brown 10YR 4/3, spinnerets strong brown 7.5YR 4/6. *Cephalothorax*. Carapace 10.45 long, 9.5 wide. Pars cephalica 6.88 long. Foveal groove procurved, 0.5 long, 1.92 wide. Eye tubercle low under ME. AER procurved PER straight. Eye group 1.02 long, 2.51 wide, AME 0.49, PME 0.32, ALE 0.51, PLE 0.39. Sternum sparsely setose, STRl 6.1, STRw 6.05. Chelicerae with anterior row comprising seven teeth, posterior margin with five teeth. Palpal endites with 32 cuspules spread across proximal half of endite and 27 smaller cuspules distally, ENDw 2.51, ENDl 3.94. Labium with seven cuspules, LBw 2.41, LBl 1.39. Rastellum with many strong spines on process and up cheliceral face for ~ 3 ´ length of process. *Abdomen*. Evenly setose; and lighter dorsally. *Legs*. F1 5.8; F1w 2.16; P1 3.58; Ti1 3.64; Mt1 2.43; Tr1 1.43; F3 4.96; F3w 2.98; P3 3.75; Ti3 3.11, Sd3 2.1; Mt3 1.92; Tr3 2.1; F4 6.96; F4w 2.91; P4 3.58; Ti4 3.63; Mt4 3.69; Tr4 1.95. Retrolateral face tarsus IV with longitudinal cluster of spinules over central half of tarsus surrounded in setae. *Pedipalps*. PF 5.48, PP 3.08, PTi 3.69, PTr 3.01. Spermathecae tilted laterally with distal coil, bulbs face laterally.

##### Variation, females

**(n = 2).**CL 6.14–10.45, 8.29±2.16; CW 5.93–9.5, 7.71±1.79; Cap 4.22–6.88, 5.55±1.33; ENDl 0.66–1.02, 0.84±0.18; ENDw 1.43–1.86, 1.65±0.22; STRl 3.63–6.1, 4.87±1.23; STRw 3.49–6.05, 4.77±1.28; LBl 1–1.39, 1.19±0.19; LBw 1.46–2.41, 1.94±0.47; F1 3.47–5.8, 4.63±1.16; F1w 1.38–2.16, 1.77±0.39; P1 2.29–3.58, 2.94±0.65; Ti1 2.2–3.64, 2.92±0.72; Mt1 1.29–2.42, 1.85±0.57; Tr1 0.95–1.43, 1.19±0.24; F3 3.09–4.96, 4.03±0.93; F3w 1.87–2.98, 2.43±0.56; P3 2.13–3.75, 2.94±0.81; Ti3 1.7–3.11, 2.4±0.7; Mt3 1.25–1.92, 1.59±0.33; Tr3 1.21–2.1, 1.66±0.45; F4 4.21–6.96, 5.58±1.37; F4w 1.8–2.91, 2.36±0.55; P4 2.22–3.58, 2.9±0.68; Ti4 2.1–3.63, 2.87±0.77; Mt4 2.14–3.69, 2.92±0.77; Tr4 1.43–1.95, 1.69±0.26; PF 3.19–5.48, 4.33±1.15; PP 1.86–3.08, 2.47±0.61; PTi 2.19–3.69, 2.94±0.75; PTr 1.55–3.01, 2.28±0.73.

##### Males.

Unknown.

##### Material examined.

**Mexico: Sonora**: 16 mi E of Hermosillo, 29.0894 -110.6947^5^, 371 m a.s.l. (UMM0464, 20.viii.1960, 1♀, Zweifel et al, AMNH); Guaymas, 27.914 -110.902^6^, 17 m a.s.l. (UMM0533, 20.viii.1964, 1♀, WJ Gertsch, JM Woods, AMNH).

#### Ummidia rodeo sp. nov.

Taxon classificationAnimaliaAraneaeHalonoproctidae

7D466154-16AA-5B06-BC1E-3CA5B3E857B2

http://zoobank.org/97E05AB6-D099-4706-A23A-CE56E7F90FC6

**Figure 37.**
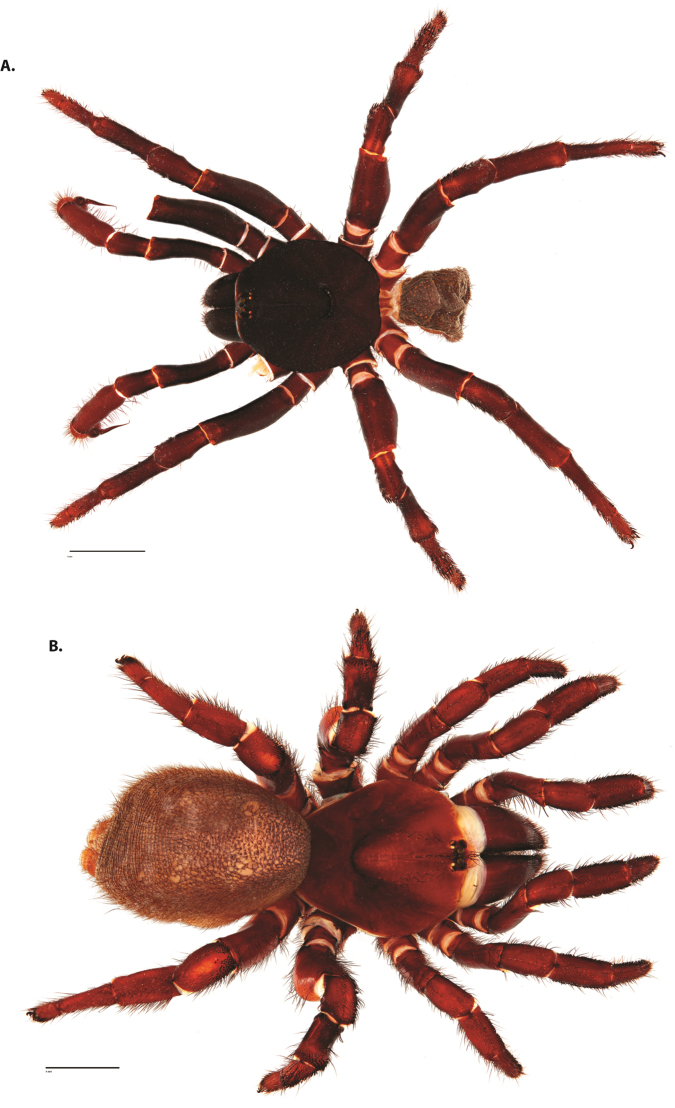
*Ummidia rodeo* sp. nov. from Durango, Mexico **A** male habitus illustration UMM325 **B** female habitus illustration UMM166. Scale bars: 4.0 mm.

**Figure 38.**
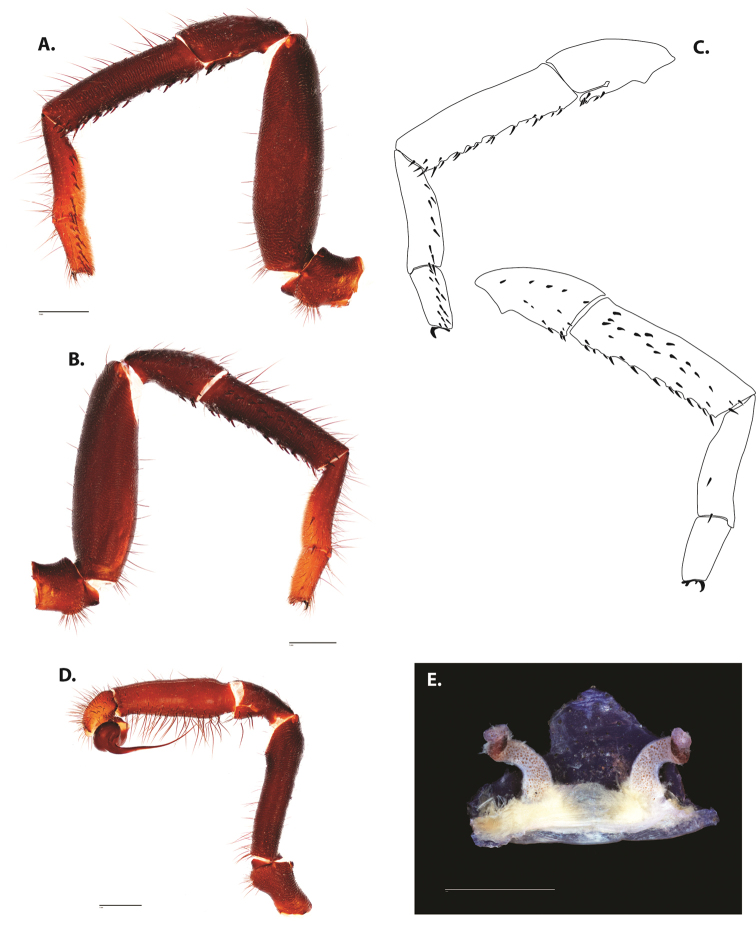
*Ummidia rodeo* sp. nov. from Durango, Mexico **A–D** male holotype (UMM325) **A** prolateral aspect, leg I **B** retrolateral aspect, leg I **C** line drawings, leg I prolateral and retrolateral aspects **D** retrolateral aspect, pedipalp **E** cleared spermathecae female paratype (UMM166). Scale bars: 1.0 mm.

Figs 37 , 38 , Map 4

##### Type material.

HOLOTYPE ♂ (UMM325) from 5 miles south of Rodeo, Durango, Mexico, 25.1081 -104.5556^5^, 1365 m a.s.l., 17.vii.1975, coll. D Ubick, AMNH. PARATYPE: 1 ♀ (UMM166) from Yerbanís, 80 miles northeast of Durango, 24.7356 -103.8364 6, 1905 m a.s.l., 1.viii.1947, coll. WJ Gertsch, AMNH.

##### Etymology.

The specific epithet is a noun taken in apposition and is in reference to the type locality.

##### Diagnosis.

*Ummidia rodeo* males can be differentiated from *U. gabrieli* by the presence of prolateral spines and by having relatively more retrolateral spines (24 vs 11) on tibia I. Females can be differentiated from *U. gabrieli* by having relatively more distal endite cuspules (59 vs 28), from *U. pustulosa* by have the sternum and carapace roughly as wide as long, and from all geographically proximate species by having the labium 2× wide as long (1.5× wide as long in other species). Males disperse in July.

##### Description of male holotype.

*Specimen preparation and condition*. Specimen preserved in 80% EtOH. Left palp and legs I&II removed; in vial with specimen. *General coloration*. Carapace and chelicerae reddish black 7.5YR 2.5/1, legs very dusky red 2.5YR 2.5/2. Abdomen black 10YR 2/1. *Cephalothorax*. Carapace 7.20 long, 6.95 wide. Pars cephalica 5.05 long. Foveal groove procurved, 0.46 long, 1.55 wide. Tubercle moderate, only under AME. AER slightly procurved. PER relatively straight. Eye group 0.74 long, 1.46 wide, AME 0.38, PME 0.17, ALE 0.36, PLE 0.19. Sternum sparsely setose, STRl 3.92, STRw 3.84. Chelicerae with anterior tooth row comprising six teeth, posterior margin with four teeth. Palpal endites with 13 non-hastate cuspules over proximal 1/4 of endite face, lacking distal endite cuspules, ENDw 1.64, ENDl 2.88. Labium with seven small cuspules, LBw 1.48, LBl 1.23. Rastellum with five small spines along distal margin of process. Abdomen setose. *Legs*. F1 6.42; F1w 1.88; P1 3.01; Ti1 4.28; Mt1 2.97; Tr1 1.53; F3 4.84; F3w 2.16; P3 2.55; Ti3 2.86; Sd3 2.11; Mt3 2.7; Tr3 1.74; F4 6.45; F4w 2.03; P4 2.92; Ti4 3.99; Mt4 4.33; Tr4 1.85. Retrolateral face of tarsus IV lacking distinct comb or brush. Leg I spination pattern: TSp 16, TSpv 10, TSrd 0, TSr 0, TSrv 24, MtSp 2, MtSr 9, TrSp 1, TrSr 13. *Pedipalps*. PTl 3.83, PTw 1.36, Bl 3.43. Embolus sinuous.

##### Variation, males

**(n = 2).**CL 6.17–7.2, 6.68±0.52; CW 6.22–6.95, 6.58±0.36; Cap 4.19–5.05, 4.62±0.43; ENDl 0.65–0.74, 0.69±0.04; ENDw 1.46–1.48, 1.47±0.01; STRl 3.59–3.92, 3.75±0.17; STRw 3.33–3.84, 3.58±0.26; LBl 0.77–1.64, 1.21±0.44; LBw 1.06–1.48, 1.27±0.21; F1 5.79–6.42, 6.1±0.32; F1w 1.46–1.88, 1.67±0.21; P1 2.64–3.01, 2.83±0.18; Ti1 3.48–4.28, 3.88±0.4; Mt1 2.52–2.97, 2.75±0.23; Tr1 1.52–1.53, 1.53±0; F3 4.13–4.84, 4.48±0.35; F3w 1.68–2.16, 1.92±0.24; P3 2.24–2.55, 2.39±0.16; Ti3 2.11–2.24, 2.17±0.06; Mt3 2.49–2.7, 2.6±0.1; Tr3 1.74–1.83, 1.78±0.04; F4 5.23–6.45, 5.84±0.61; F4w 1.73–2.03, 1.88±0.15; P4 2.47–2.92, 2.7±0.23; Ti4 3.35–3.99, 3.67±0.32; Mt4 3.5–4.33, 3.92±0.41; Tr4 0–1.94, 0.97±0.97; TSp 10–16, 13±3; TSpv 5–10, 7.5±2.5; TSr 0–0, 0±0; TSrv 0–23, 11.5±11.5; PTl 3.74–3.83, 3.78±0.05; PTw 1.23–1.36, 1.29±0.06; BL 2.95–3.43, 3.19±0.24.

##### Description of female paratype.

*Specimen preparation and condition*. Specimen preserved in 80% EtOH. Spermathecae removed, cleared, in vial with specimen. *General coloration*. Carapace, chelicerae, and legs very dusky red 10R 2.5/2. Abdomen reddish black 2.5YR 2.5/1, spinnerets dark yellowish brown 10YR 3/6. *Cephalothorax*. Carapace 9.26 long, 8.57 wide. Pars cephalica 6.39 long. Foveal groove procurved, 0.67 long, 2.39 wide. Eye tubercle low. AER procurved. PER straight to slightly recurved. Eye group 1.02 long, 2.08 wide, AME 0.43, PME 0.22, ALE 0.52, PLE 0.33. Sternum sparsely setose, STRl 5.95, STRw 5.24. Chelicerae with anterior row comprising seven teeth, posterior margin with six teeth. Palpal endites with 15 cuspules spread across proximal half of endite and 42 smaller cuspules distally, ENDw 2.08, ENDl 3.45. Labium with three cuspules, LBw 2.07, LBl 1.08. Rastellum with many strong spines on process and up cheliceral face for ~ 1× length of process. *Abdomen*. Evenly setose with pale speckles clustered at apodemes. *Legs*. F1 5.1; F1w 1.9; P1 3.2; Ti1 3.04; Mt1 2.3; Tr1 1.25; F3 4.7; F3w 2.6; P3 2.85; Ti3 2.56, Sd3 1.55; Mt3 2.03; Tr3 1.86; F4 6.12; F4w 2.65; P4 3.08; Ti4 3.12; Mt4 3.33; Tr4 1.82. Retrolateral face tarsus IV with defined comb over length of tarsus. *Pedipalps*. PF 4.75, PP 2.54, PTi 3.1, PTr 2.6. Spermathecae curved laterally with distal coil, bulbs face anterolaterally.

##### Variation, females

**(n = 5).**CL 8.23–9.26, 8.72±0.17; CW 7.74–8.57, 8.15±0.14; Cap 5.77–6.39, 6.02±0.11; ENDl 0.86–1.04, 0.98±0.03; ENDw 1.17–1.87, 1.65±0.13; STRl 5.05–5.95, 5.37±0.16; STRw 4.58–5.24, 5.01±0.13; LBl 1.08–1.37, 1.22±0.05; LBw 1.81–2.07, 1.96±0.04; F1 4.69–5.1, 4.85±0.07; F1w 1.73–1.9, 1.83±0.03; P1 2.97–3.2, 3.1±0.04; Ti1 2.88–3.06, 2.96±0.04; Mt1 1.96–2.3, 2.08±0.07; Tr1 1.15–1.44, 1.27±0.05; F3 4.28–4.75, 4.55±0.09; F3w 2.35–2.62, 2.48±0.05; P3 2.85–2.95, 2.89±0.02; Ti3 2.48–2.7, 2.59±0.04; Mt3 1.77–2.03, 1.88±0.05; Tr3 1.72–1.95, 1.84±0.05; F4 5.59–6.12, 5.78±0.1; F4w 2.41–2.65, 2.52±0.05; P4 2.81–3.31, 3.04±0.08; Ti4 2.92–3.26, 3.08±0.06; Mt4 2.59–3.33, 3.06±0.13; Tr4 1.63–1.91, 1.75±0.05; PF 4.54–4.75, 4.63±0.04; PP 2.54–2.78, 2.63±0.04; PTi 2.93–3.19, 3.06±0.05; PTr 2.44–2.69, 2.57±0.04.

##### Material examined.

**Mexico: Chihuahua**: 5 mi E Parral, 26.9323 -105.5847^5^, 548 m a.s.l. (UMM0626, 15.vii.1947, 1♀, WJ Gertsch, AMNH); Summit NE of San José Babícora, 29.2979 -107.537^5^, 1158 m a.s.l. (UMM0629, 3.vii.1947, 1♂, WJ Gertsch, AMNH); **Durango**: San Lucas Ocampo, 24.0072 -104.6662^6^, 1887 m a.s.l. (UMM0298, 1.viii.1947, 1♀, WJ Gertsch, AMNH); Yerbanís, 80 mi NE Durango, 24.7356 -103.8364^6^, 1905 m a.s.l. (UMM0444, 19.viii.1947, 1♀, WJ Gertsch, AMNH); (UMM0308, 1♀, AMNH); (UMM0448, 1♀, AMNH); (UMM0449, 1♀, AMNH); (UMM0581, 1♀, AMNH); (UMM0619, 1♀, AMNH); (UMM0166, 1♀, AMNH); 5 mi S Rodeo, 25.1081 -104.5556^5^, 1365 m a.s.l. (UMM0325, 17.vii.1975, 1♂, D Ubick, AMNH).

#### Ummidia pustulosa

Taxon classificationAnimaliaAraneaeHalonoproctidae

(Becker, 1879)

8C5B1055-42CD-5ED3-A542-9B0572CBABAE

Fig. 39 , Map 4

Pachylomerus pustulosus Becker, 1879: 140.
Pachylomerus pustulosus Becker, 1881: 44.

##### Type material.

Female holotype presumed lost. NEOTYPE 1♀ designated here (NHMUK1071) from Amula (now Jalisco), Mexico, 20.6934 -103.356^7^, 1550 m a.s.l., H.H. Smith 1898, NHMUK. The original type locality is simply listed as “Guanajuato, Mexico” in the original description which neighbors the locality of the neotype designated, Jalisco, Mexico. Given the carapace length to width ratio noted in Becker’s (1879) description and the illustration of the sternum (Becker 1881, fig. 2b) we can be reasonably confident that designation of this specimen as the neotype is consistent with what is known of the originally described specimen.

##### Diagnosis.

Females of *U. pustulosa* can be differentiated from *U. pesiou* and *U. rodeo* by having the carapace and sternum 1.2× long as wide (roughly long as wide in other species) and from *U. gabrieli* by having relatively more endite cuspules (43 vs 28).

##### Female neotype.

*Specimen preparation and condition*. Specimen preserved in 80% EtOH. Spermathecae removed, cleared, in vial with specimen. *General coloration*. Carapace, chelicerae, and legs dark reddish brown 2.5YR 3/4. Abdomen brown 7.5YR 4/3, spinnerets strong brown 7.5YR 4/6. *Cephalothorax*. Carapace 9.44 long, 7.99 wide. Pars cephalica 6.36 long. Foveal groove slightly procurved, 0.21 long, 1.7 wide. Eye tubercle low. AER procurved. PER recurved. Eye group 0.93 long, 1.59 wide, AME 0.34, PME 0.19, ALE 0.49, PLE 0.32. Sternum moderately setose, STRl 5.92, STRw 4.85. Chelicerae with anterior row comprising seven teeth, posterior margin with seven teeth. Palpal endites with 20 cuspules spread across proximal half of endite and 23 smaller cuspules distally, ENDw 2.08, ENDl 3.5. Labium with four cuspules, LBw 2.04, LBl 1.48. Rastellum with very many strong spines on process and along margin continuing up cheliceral face approx. length of rastellar process. *Abdomen*. Evenly setose. *Legs*. F1 5.66; F1w 1.8; P1 3.52; Ti1 3.3; Mt1 2.46; Tr1 1.43; F3 4.93; F3w 2.57; P3 3.16; Ti3 2.91, Sd3 1.84; Mt3 2.06; Tr3 1.98; F4 5.75; F4w 2.66; P4 3.35; Ti4 3.33; Mt4 3.31; Tr4 1.82. Retrolateral face tarsus IV with very defined comb over length of tarsus. *Pedipalps*. PF 5.32, PP 2.99, PTi 3.29, PTr 3.1. Spermathecae curved laterally with distal coil, bulbs facing laterally.

**Figure 39.**
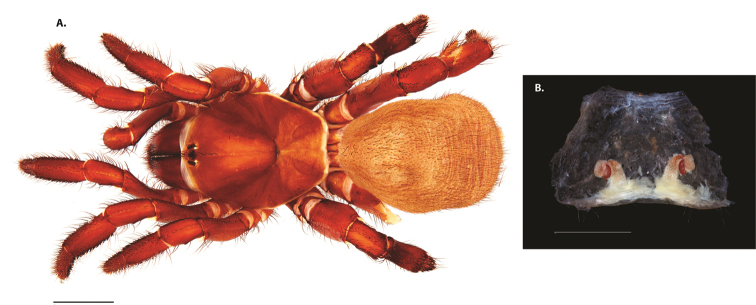
*Ummidia pustulosa* (Becker, 1879) from Sonora, Mexico. Female neotype (BMNH1071) **A** female habitus illustration **B** cleared spermathecae. Scale bars: 4.0 mm (**A**), 1.0 mm (**B**).

##### Variation, females

**(n = 3).**CL 8.65–9.53, 9.21±0.28; CW 7.5–8.62, 8.04±0.32; Cap 5.63–6.36, 6.05±0.22; ENDl 0.93–1, 0.96±0.02; ENDw 1.59–1.84, 1.68±0.08; STRl 5.27–5.92, 5.65±0.19; STRw 4.31–4.85, 4.64±0.17; LBl 1.48–1.65, 1.56±0.05; LBw 1.62–2.04, 1.87±0.13; F1 3.38–5.66, 4.63±0.67; F1w 1.89–2.06, 1.97±0.05; P1 3.03–3.58, 3.38±0.18; Ti1 2.96–3.33, 3.2±0.12; Mt1 2.17–2.59, 2.4±0.12; Tr1 1.28–1.43, 1.34±0.05; F3 4.39–4.93, 4.71±0.16; F3w 2.31–2.65, 2.51±0.1; P3 2.9–3.5, 3.19±0.17; Ti3 2.78–2.97, 2.89±0.06; Mt3 1.96–2.06, 2.03±0.03; Tr3 1.88–1.99, 1.95±0.03; F4 5.59–6.48, 5.94±0.28; F4w 2.33–2.75, 2.58±0.13; P4 3–3.55, 3.3±0.16; Ti4 3.33–3.57, 3.45±0.07; Mt4 3.16–3.47, 3.31±0.09; Tr4 1.57–1.93, 1.77±0.11; PF 4.33–5.32, 4.98±0.32; PP 2.72–3.17, 2.96±0.13; PTi 2.88–3.51, 3.23±0.18; PTr 2.49–3.1, 2.82±0.18.

##### Males.

Unknown.

##### Material examined.

**Mexico: Jalisco**: Amula (now Jalisco), Mexico, 20.6934 -103.356^7^, 1550 m a.s.l. (NHMUK1071, 1898, 1♀, HH Smith, NHMUK); Chamela Biological Station, 19.5307 -105.0828^6^, 13 m a.s.l. (UMM0465, 21.vii.1989, 1♀, JG Rozen, AMNH); **Sinaloa**: 35 mi N of Los Mochis, former desert floor within 0.5 mi of the ocean, 26.2772 -109.1947^6^, 15 m a.s.l. (UMM0222, iii.1961, 1♀, WW Gibson, MCZ).

### Southern Mexico And Central America

#### Ummidia huascazaloya sp. nov.

Taxon classificationAnimaliaAraneaeHalonoproctidae

111C66DE-ED86-5C08-BA7E-45C85566D8A0

http://zoobank.org/9409326F-C38C-4ED9-AEC4-29FDD72D18D3

**Figure 40.**
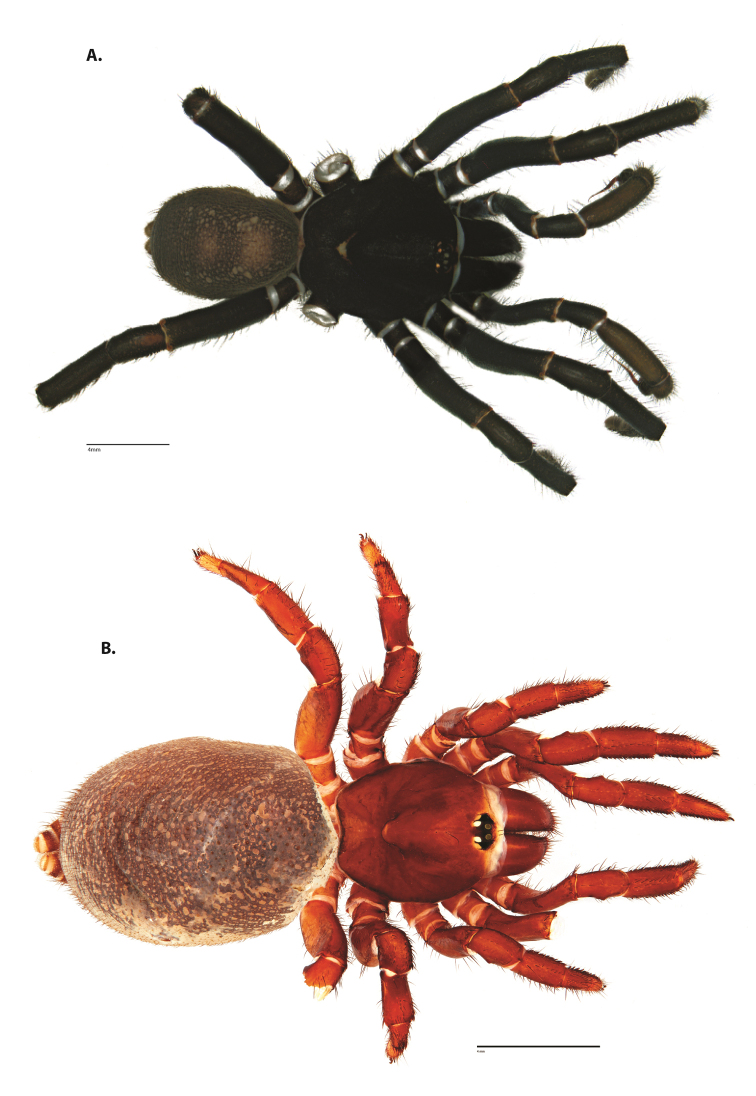
*Ummidia huascazaloya* sp. nov. from Hidalgo, Mexico **A** male habitus illustration CNAN-T01384 **B** female habitus illustration UMM171. Scale bars: 4.0 mm.

**Figure 41.**
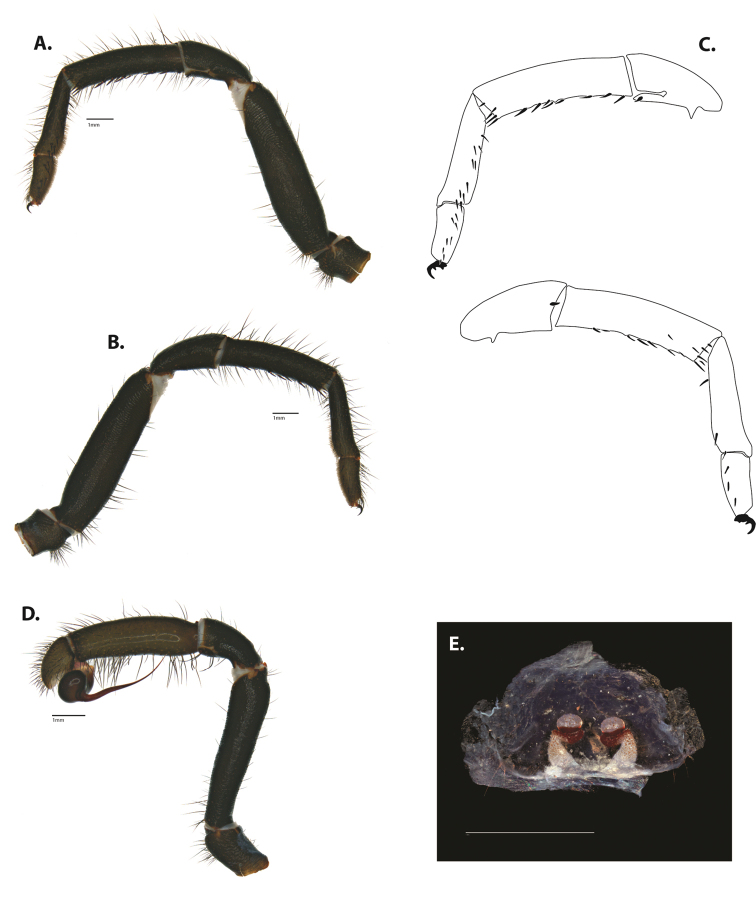
*Ummidia huascazaloya* sp. nov. from Hidalgo, Mexico **A–D** male holotype (CNAN-T01384) **A** retrolateral aspect, leg I **B** prolateral aspect, leg I **C** line drawings, leg I retrolateral and prolateral aspects **D** retrolateral aspect, pedipalp **E** cleared spermathecae female paratype (UMM171). Scale bars: 1.0 mm.

Figs 40 , 41 , Map 5

##### Type material.

HOLOTYPE ♂ (CNAN-T01384) from just outside Peña del Aire Park, outside Huasca de Ocampo, Hidalgo, Mexico, 20.2762 -98.5169^1^, 2046 m a.s.l., 6–7.vi.2019, coll. PE Cushing, H Carmona, DL Batista Perales, J Rojas Castillo, CNAN. PARATYPE: 1 ♀ (UMM171) from Chapulhuacán, Hidalgo, Mexico, 21.1569 -98.9026^6^, 967 m a.s.l.. coll. F Bonet, AMNH.

##### Etymology.

The specific epithet is a noun taken in apposition and is in reference to the original Nahuatl name for Huasca de Ocampo.

##### Diagnosis.

*Ummidia huascazaloya* can be differentiated from *U. anaya* by the presence of a defined comb on the retrolateral face of tarsus IV in males and females. *U. huascazaloya* males can be further differentiated from *U. anaya* by having a palpal tibia (3.25× long as wide vs 1.85×) and tarsus IV (3.6× long as wide vs 2.9×) which are more elongate as well as having fewer, larger endite cuspules (10 vs 22). Males can be differentiated from *U. cuicatec* and *U. zilchi* by having a sinuous embolus without a strong bend and from *U. cuicatec*, *U. zebrina*, *U. brandicarlileae*, and *U. yojoa* by having more spines on the prolateral face of tibia I (11 vs 0-2). Males can be differentiated from *U. riverai* by lacking a pale dorsal heart patch on the abdomen. Females can be differentiated from *U. gabrieli*, *U. pesiou*, *U. rodeo*, *U. pustulosa*, and *U. frankellerae* by having spermathecae that bend medially then anteriorly. Males disperse in June.

##### Description of male holotype.

*Specimen preparation and condition*. Specimen preserved in 80% EtOH. Right and left leg III missing. *Cephalothorax*. Carapace 7.36 long, 7.12 wide. Pars cephalica 5.25 long. Foveal groove procurved, 0.48 long, 1.76 wide. Tubercle moderate, mostly restricted to ME. AER procurved. PER recurved. Eye group 0.88 long, 1.62 wide, AME 0.34, PME 0.24, ALE 0.51, PLE 0.3. Sternum moderately setose, STRl 4.31, STRw 3.88. Chelicerae with anterior tooth row comprising six teeth, posterior margin with six teeth. Palpal endites with ten relatively long non-haste spinules over proximal half of endite face, lacking distal endite cuspules, ENDw 1.59, ENDl 2.72. Labium with three relatively long, non-hastate cuspules, LBw 1.44, LBl 1.12. Rastellum with many spines on process. Abdomen setose with pale speckles concentrated at apodemes. *Legs*. F1 6.56; F1w 1.68; P1 3.12; Ti1 4.4; Mt1 3.2; Tr1 1.52; F4 6.48; F4w 1.96; P4 2.88; Ti4 3.92; Mt4 4.4; Tr4 2.24. Retrolateral face of tarsus IV with defined comb with heavier spinules over distal half. Leg I spination pattern: TSp 6, TSpv 5, TSrd 0, TSr 1, TSrv 17, MtSp 3, MtSr 12, TrSp 2, TrSr 8. *Pedipalps*. PTl 3.9, PTw 1.2, Bl 3.7. Embolus long; sinuous, bulb relatively small.

##### Variation, males.

Known only from male holotype specimen.

##### Description of female paratype.

*Specimen preparation and condition*. Specimen preserved in 80% EtOH. Spermathecae removed, cleared, in vial with specimen. *General coloration*. Carapace, chelicerae, and legs dark reddish brown 2.5YR 2.5/4, Tr IV strong brown 7.5YR 5/8. Abdomen black 7.5YR 2.5/1, spinnerets yellowish brown 10YR 5/6. *Cephalothorax*. Carapace 4.99 long, 4.71 wide. Pars cephalica 3.69 long. Foveal groove procurved, 1.09 long, 0.47 wide. All eyes on moderate tubercle. AER procurved. PER straight. Eye group 0.78 long, 1.22 wide, AME 0.3, PME 0.3, ALE 0.41, PLE 0.34. Sternum very sparsely setose around edges, thicker anteriorly STRl 3.03, STRw 2.97. Chelicerae with anterior row comprising five teeth, posterior margin with six teeth. Palpal endites with 30 very large cuspules spread across proximal half of endite face and 15 smaller cuspules distally, ENDw 1.23, ENDl 2.09. Labium with 13 very large cuspules, LBw 1.19, LBl 0.91. Rastellum with large blunt spines along rastellar margin and several smaller blunt spines on rastellar process. *Abdomen*. Evenly setose with pale speckles. *Legs*. F1 3.36; F1w 1.27; P1 2.09; Ti1 1.98; Mt1 1.33; Tr1 1.72; F3 2.86; F3w 1.57; P3 1.67; Ti3 1.61, Sd3 1.09; Mt3 1.24; Tr3 1.12; F4 3.59; F4w 1.5; P4 2.04; Ti4 1.92; Mt4 1.9; Tr4 1.02. Retrolateral face tarsus IV with comb of alternating long and short spinules. *Pedipalps*. PF 3.25, PP 1.72, PTi 1.81, PTr 1.67. Spermathecae bent medially then folded anteriorly, bulbs facing anteriorly.

##### Variation, females.

Known only from female paratype specimen.

##### Known only from type material.

#### Ummidia anaya sp. nov.

Taxon classificationAnimaliaAraneaeHalonoproctidae

B7EEA986-699F-5FAF-964C-A99A8AAFAFE0

http://zoobank.org/851CAC8A-F15B-4DEF-BE0A-5B9A6248E754

Fig. 42 , Map 5

##### Type material.

HOLOTYPE ♂ (CNAN-T01385) from Municipio de Santiago de Anaya Hidalgo, Grutas Xoxafi, Hidalgo, Mexico, 20.3884 -99.0275^1^, 2020 m a.s.l., coll. 7–8.vi.2019, PE Cushing, E García, E González Santillán, DL Batista Perales, J Rojas Castillo, CNAN.

##### Etymology.

The specific epithet is a noun taken in apposition and is in reference to the type locality, the town of Santiago de Anaya.

##### Diagnosis.

*Ummidia anaya* can be differentiated from *U. huascazaloya* by lacking a defined comb on the retrolateral face of tarsus IV and by having a palpal tibia (1.85× long as wide vs 3.25×) and tarsus IV (2.9× long as wide vs 3.6×) which are less elongate as well as having more, smaller endite cuspules (22 vs 10). Males can be differentiated from *U. cuicatec* and *U. zilchi* by having a sinuous embolus without a strong bend and from *U. cuicatec*, *U. zebrina*, *U. brandicarlileae*, and *U. yojoa* by having relatively more spines on the prolateral face of tibia I (11 vs 0-2). Males can be differentiated from *U. riverai* by lacking a pale dorsal heart patch on the abdomen. Males disperse in June.

**Figure 42.**
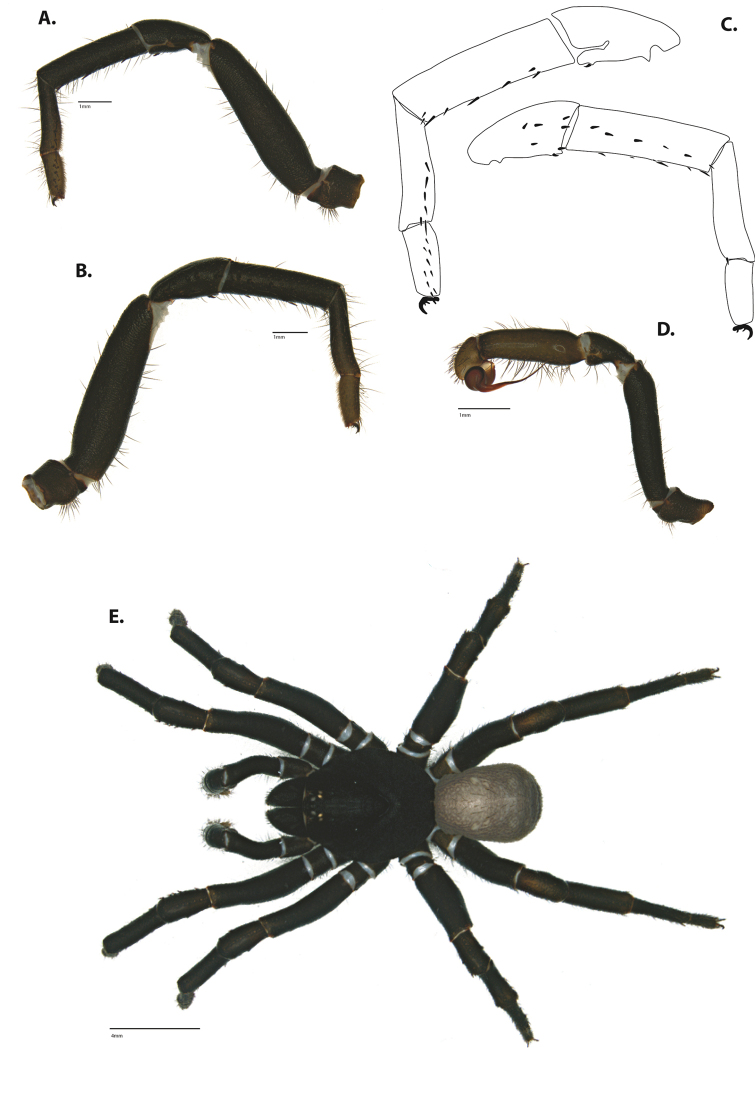
*Ummidia anaya* sp. nov. male holotype specimen (CNAN-T01385) from Hidalgo, Mexico. **A** retrolateral aspect, leg I **B** prolateral aspect, leg I **C** line drawings, leg I retrolateral and prolateral aspects **D** retrolateral aspect, pedipalp **E** habitus illustration. Scale bars: 1.0 mm (**A–D**), 4.0 mm (**E**).

##### Description of male holotype.

*Specimen preparation and condition*. Specimen preserved in 80% EtOH. *Cephalothorax*. Carapace 5.88 long, 5.5 wide. Pars cephalica 3.75 long. Foveal groove procurved, 0.5 long, 1.13 wide. ME on moderate tubercle. AER procurved. PER straight. Eye group 0.66 long, 1.28 wide, AME 0.28, PME 0.18, ALE 0.36, PLE 0.2. Sternum moderately setose, STRl 3.4, STRw 3.1. Chelicerae with anterior tooth row comprising four teeth, posterior margin with four teeth. Palpal endites with 22 small, subhastate spinules over proximal half of endite face, lacking distal endite cuspules, ENDw 1.16, ENDl 2.16. Labium with seven small cuspules, LBw 1.12, LBl 0.88. Rastellum many spines on process. Abdomen setose with pale speckles. *Legs*. F1 5.5; F1w 1.44; P1 2.56; Ti1 3.13; Mt1 2.44; Tr1 1.25; F3 4.05; F3w 1.6; P3 2.09; Ti3 2.52; Sd3 1.52; Mt3 2.2; Tr3 1.4; F4 5.38; F4w 1.5; P4 2.31; Ti4 3.2; Mt4 3.45; Tr4 1.6. Retrolateral face of tarsus IV lacking distinct comb or brush, with two loose rows of spinules. Leg I spination pattern: TSp 7, TSpv 0, TSrd 0, TSr 0, TSrv 15, MtSp 0, MtSr 8, TrSp 0, TrSr 9. *Pedipalps*. PTl 1.96, PTw 1.06, Bl 2.81. Embolus long, sinuous, bulb relatively small.

##### Variation, males.

Known only from male type specimen.

##### Females.

Unknown.

#### Ummidia cuicatec sp. nov.

Taxon classificationAnimaliaAraneaeHalonoproctidae

237473CD-CBAF-5472-87D7-CE0F52106898

http://zoobank.org/82916089-EA27-4062-9DD7-D4ADDF91A3D0

**Figure 43.**
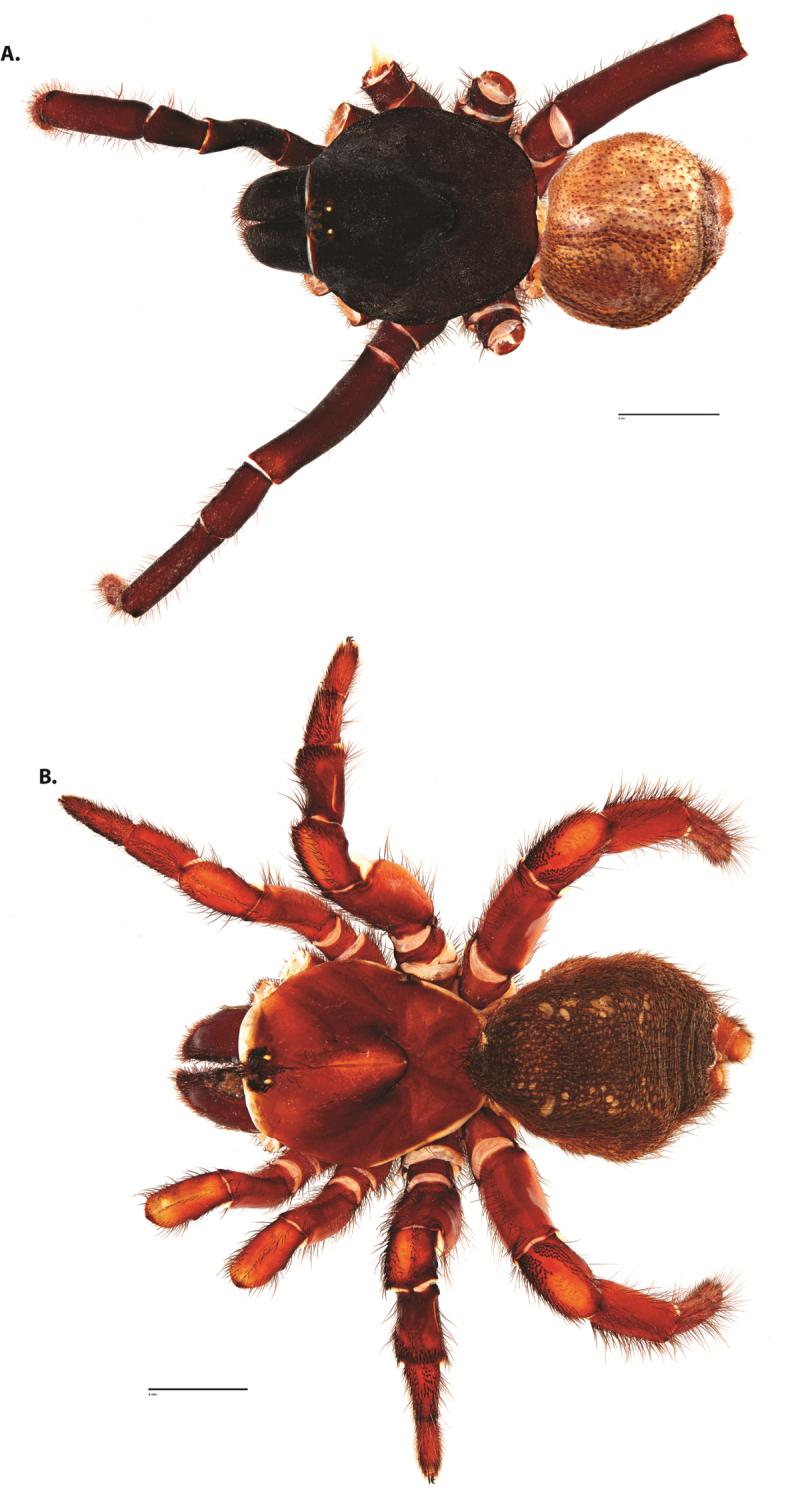
*Ummidia cuicatec* sp. nov. from Oaxaca, Mexico. **A** male habitus illustration UMM154 **B** female habitus illustration UMM415. Scale bars: 4.0 mm.

**Figure 44.**
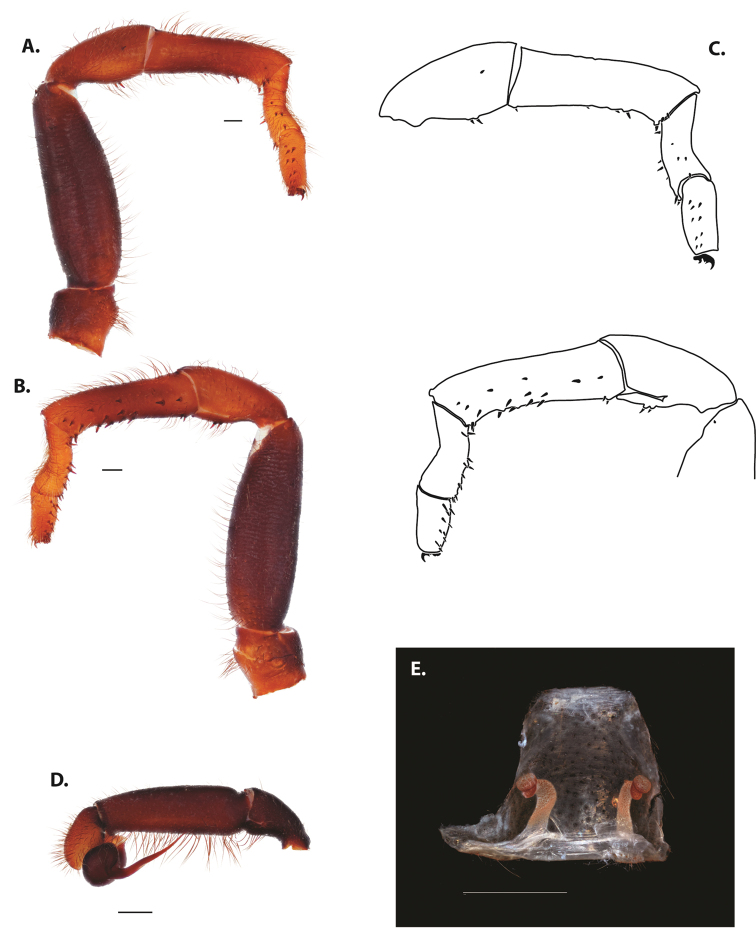
*Ummidia cuicatec* sp. nov. from Oaxaca, Mexico. **A–D** male holotype (UMM154) **A** prolateral aspect, leg I **B** retrolateral aspect, leg I **C** line drawings, leg I prolateral and retrolateral aspects **D** retrolateral aspect, pedipalp **E** cleared spermathecae female paratype (UMM415). Scale bars: 1.0 mm.

Figs 43 , 44 , Map 5

##### Type material.

HOLOTYPE ♂ (UMM154) from Cuicatlán, Oaxaca, Mexico, 17.7984 -96.9519^6^, 752 m a.s.l., 13.vi.1944, coll. H Wagner, AMNH. PARATYPE: 1 ♀ (UMM415, AMNH) from Portillo de Nejapa, Oaxaca, Mexico, 16.061 -95.9797^6^, 635 m a.s.l., 20.vii.1966, coll. CM Bogert, AMNH.

##### Etymology.

The specific epithet is a noun taken in apposition and is in reference to the indigenous people group for whom the locality is named, the Cuicatec.

##### Diagnosis.

*Ummidia cuicatec* is a relatively large species in which males can be differentiated from all other species by having a long, sinuous embolus with an extreme distal curve. Males can be differentiated from *U. anaya*, *U. huascazaloya*, *U. zilchi*, *U. riverai*, and *U. hondurena* by having fewer spines on prolateral (2 vs 7-28) and retrolateral (10 vs 15-42) faces of tibia I and from *U. riverai* by lacking a pale dorsal heart patch on the abdomen. Females can be differentiated from all other geographically proximal species except *U. pustulosa* and *U. frankellerae* by having spermathecae that curve laterally and end in a distal coil with bulbs facing laterally. Males disperse in June.

##### Description of male holotype.

*Specimen preparation and condition*. Specimen preserved in 80% EtOH. Specimen in poor condition, metatarsus I appears to have been damaged. *General coloration*. Carapace and chelicerae reddish black 10R 2.5/1, legs very dusky red 10R 2.5/2. Abdomen very dark brown 7.5YR 2.5/3. *Cephalothorax*. Carapace 8.96 long, 8.53 wide. Pars cephalica 5.83 long. Foveal groove procurved, 0.78 long, 1.63 wide. All eyes on low tubercle. AER procurved. PER straight. Eye group 0.97 long, 1.64 wide, AME 0.37, PME 0.26, ALE 0.58, PLE 0.31. Sternum sparsely setose, STRl 5.21, STRw 4.86. Chelicerae with anterior tooth row comprising six teeth, posterior margin with four teeth. Palpal endites with 19 cuspules spread over proximal half of endite face, lacking distal endite cuspules, ENDw 1.83, ENDl 3.25. Labium with two cuspules, LBw 1.72, LBl 1.33. Rastellum with many small teeth on process. Abdomen setose. *Legs*. F1 7.78; F1w 2.12; P1 3.7; Ti1 5.62; Mt1 4.24; Tr1 2.02; F3 5.8; F3w 2.39; P3 3.01; Ti3 3.52; Sd3 2.06; Mt3 3.22; Tr3 2.55; F4 7.7; F4w 2.41; P4 3.45; Ti4 4.93; Mt4 5.51; Tr4 3.1. Retrolateral face of tarsus IV with distinct comb of spinules, which is surrounded in setae. Leg I spination pattern: TSp 0, TSpv 2, TSrd 0, TSr 0, TSrv 10, MtSp 3, MtSr 7, TrSp 3, TrSr 4. *Pedipalps*. PTl 4.28, PTw 1.5, Bl 3.63. Embolus relatively long and sinuous with strong bend distally.

##### Variation, males.

Known only from male type specimen.

##### Description of female paratype.

*Specimen preparation and condition*. Specimen preserved in 80% EtOH. Spermathecae removed, cleared, in vial with specimen. Right palp and leg IV and left leg II Ti-TR removed and in vial. Left palp missing. *General coloration*. Carapace, chelicerae, and legs dark reddish brown 2.5YR 2.5/4. Abdomen reddish black 2.5YR 2.5/1, spinnerets dark reddish brown 5YR 3/4. *Cephalothorax*. Carapace 8.98 long, 8.54 wide. Pars cephalica 6.4 long. Foveal groove procurved, 0.84 long, 2.27 wide. All eyes on moderate tubercle. AER procurved. PER procurved. Eye group 1.15 long, 1.93 wide, AME 0.43, PME 0.31, ALE 0.65, PLE 0.43. Sternum very setose around outer third and thicker posteriorly, STRl 5.83, STRw 5.54. Chelicerae with anterior row comprising four teeth, posterior margin with seven teeth. Palpal endites with 24 cuspules spread across proximal half of endite and 78 cuspules distally, ENDw 1.88, ENDl 3.42. Labium with seven cuspules, LBw 1.87, LBl 1.33. Rastellum with many strong spines on process and up cheliceral face for ~ 3 ´ length of process. *Abdomen*. Evenly setose with pale speckles clustered at apodemes. *Legs*. F1 5.17; F1w 2.09; P1 3.53; Ti1 3.09; Mt1 2.24; Tr1 1.29; F3 4.83; F3w 2.58; P3 3.26; Ti3 2.93, Sd3 2.3; Mt3 2.26; Tr3 2.04; F4 6.36; F4w 2.76; P4 3.45; Ti4 3.67; Mt4 3.85; Tr4 1.91. Retrolateral face tarsus IV with brush of spinules, retrolaterally and ventrally setose. *Pedipalps*. PF 4.62, PP 2.91, PTi 3.01, PTr 2.56. Spermathecae curved laterally with distal coil, bulbs facing laterally.

##### Variation, females.

Known only from female type specimen.

#### Ummidia brandicarlileae sp. nov.

Taxon classificationAnimaliaAraneaeHalonoproctidae

CBFC6C49-1369-5E4C-B92F-8EF54C82ADF5

http://zoobank.org/2F411436-55E0-4C1F-B797-0DF43ABC6B9D

**Figure 45.**
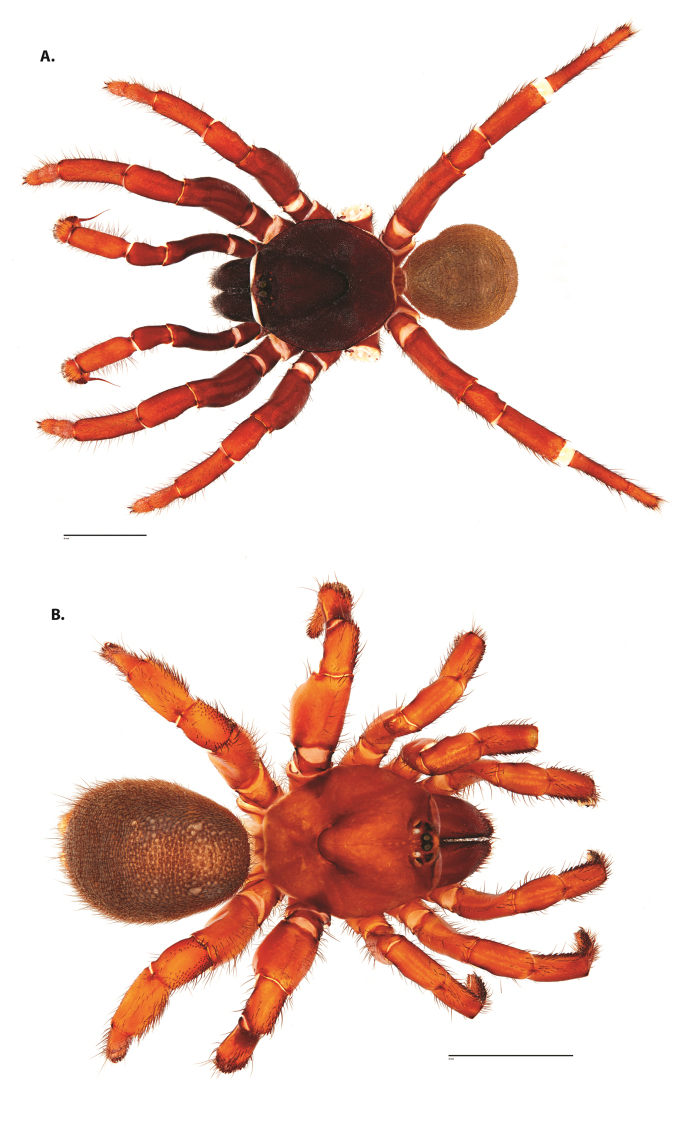
*Ummidia brandicarlileae* sp. nov. from Yucatán, Mexico. **A** male habitus illustration UMM158 **B** female habitus illustration UMM576. Scale bars: 4.0 mm.

**Figure 46.**
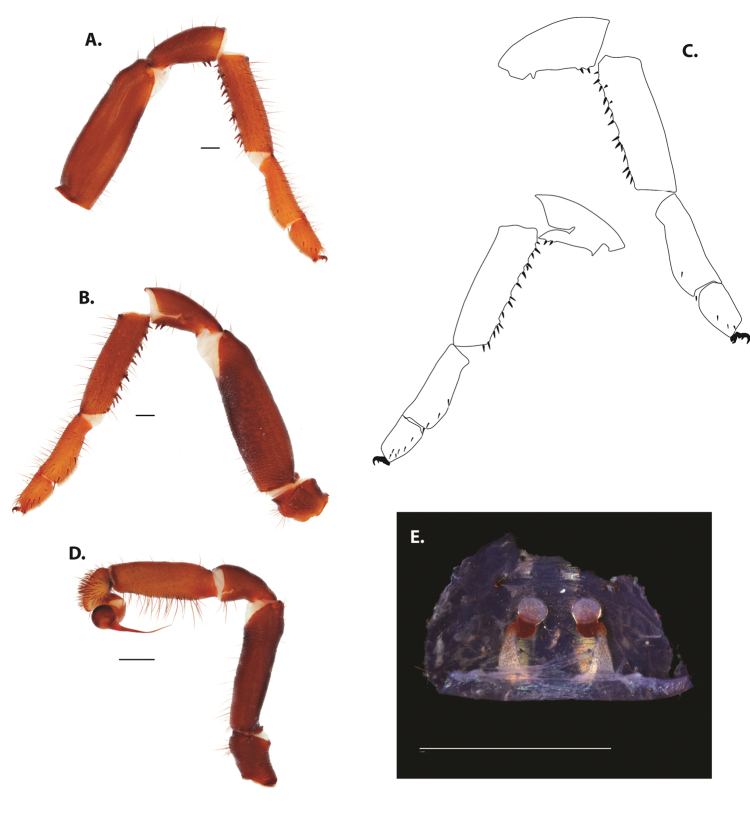
*Ummidia brandicarlileae* sp. nov. from Yucatán, Mexico. A-D male holotype (UMM158) **A** prolateral aspect, leg I **B** retrolateral aspect, leg I **C** line drawings, leg I prolateral and retrolateral aspects **D** retrolateral aspect, pedipalp **E** cleared spermathecae female paratype (UMM576). Scale bars: 1.0 mm.

Figs 45 , 46 , Map 5

##### Type material.

HOLOTYPE ♂ (UMM158) from Tekom, Yucatán, Mexico, 20.0603 -88.2654^1^, 96 m a.s.l., 17.iv.1940, coll. T. Sanderson, AMNH. PARATYPE: 1 ♀ (UMM576) from Chichén Itzá, Yucatán, Mexico, 20.6826 -88.5686^5^, 38 m a.s.l., 16.vii.1952, coll. J Ballister, D Ballister AMNH.

##### Etymology.

The specific epithet is in honor of Brandi Carlile, an American singer-songwriter who, along with Tim and Phil Hanseroth, established the Looking Out Foundation, which raises money and awareness for numerous causes to include supporting child victims of war, world hunger, LGBT rights, and the empowerment of women. Carlile founded the annual Girls Just Wanna Weekend Festival to counter the lack of female representation at mainstream music festivals, which takes place near the type locality for this species.

##### Diagnosis.

*U. brandicarlileae* can be distinguished from all other geographically proximal species except *U. yojoa* by lacking either a defined brush or comb on the retrolateral face of tarsus IV. Males can be differentiated from *U. cuicatec* and *U. zilchi* by having a sinuous embolus without a strong bend, from *U. huascazaloya*, *U. anaya*, *U. zilchi*, *U. riverai*, and *U. hondurena* by having fewer spines on prolateral (2 vs 7-28) face of tibia I and from *U. riverai* by lacking a pale dorsal heart patch on the abdomen. Males can be further differentiated from all other geographically proximate species by having tarsi I relatively short and thickened proximally. Females can be differentiated from *U. cuicatec*, *U. frankellerae* and *U. matagalpa* by having spermathecae that bend medially then anteriorly. Males disperse from April to July.

##### Description of male holotype.

*Specimen preparation and condition*. Specimen preserved in 80% EtOH. *General coloration*. Carapace and chelicerae very dusky red 10R 2.5/2, legs dark reddish brown 2.5YR 2.5/3, Mt and Tr strong brown 7.5YR 4/6. Abdomen very dark gray 7.5YR 3/1. *Cephalothorax*. Carapace 6.78 long, 6.65 wide. Pars cephalica 4.77 long. Foveal groove procurved, 0.59 long, 1.55 wide. Tubercle moderate, only under AME. AER procurved. PER straight. Eye group 0.79 long, 1.83 wide, AME 0.33, PME 0.12, ALE 0.46, PLE 0.23. Sternum with posterior fringe, sparsely setose anteriorly, STRl 4.37, STRw 3.69. Chelicerae with anterior tooth row comprising seven teeth, posterior margin with eight teeth. Palpal endites with 16 small cuspules across proximal half of endite face, lacking distal endite cuspules, ENDw 1.45, ENDl 2.71. Labium with four cuspules, LBw 1.32, LBl 1.14. Rastellum with many small spines on process. Abdomen setose. *Legs*. F1 5.61; F1w 1.57; P1 2.87; Ti1 3.75; Mt1 2.34; Tr1 1.18; F3 4.4; F3w 1.87; P3 1.98; Ti3 2.41; Sd3 1.45; Mt3 2.07; Tr3 1.61; F4 6.05; F4w 1.79; P4 2.55; Ti4 3.52; Mt4 3.63; Tr4 1.98. Retrolateral face of tarsus IV with brush. Leg I spination pattern: TSp 0, TSpv 2, TSrd 0, TSr 0, TSrv 15, MtSp 1, MtSr 4, TrSp 3, TrSr 6. *Pedipalps*. PTl 2.91, PTw 1.1, Bl 2.52. Embolus sinuous.

##### Variation, males

**(n = 2).**CL 6.78–6.92, 6.85±0.07; CW 6.65–6.8, 6.73±0.08; Cap 4.77–4.87, 4.82±0.05; ENDl 0.79–0.89, 0.84±0.05; ENDw 1.79–1.83, 1.81±0.02; STRl 4.15–4.37, 4.26±0.11; STRw 3.69–3.74, 3.72±0.03; LBl 1.13–1.14, 1.14±0.01; LBw 1.32–1.32, 1.32±0; F1 5.61–5.92, 5.77±0.15; F1w 1.57–1.78, 1.68±0.11; P1 2.87–3.09, 2.98±0.11; Ti1 3.73–3.75, 3.74±0.01; Mt1 2.34–2.65, 2.5±0.15; Tr1 1.18–1.43, 1.3±0.12; F3 4.4–4.47, 4.43±0.03; F3w 1.87–2.18, 2.03±0.15; P3 1.35–1.98, 1.66±0.32; Ti3 2.32–2.41, 2.37±0.04; Mt3 2.07–2.28, 2.17±0.1; Tr3 1.61–1.93, 1.77±0.16; F4 5.78–6.05, 5.92±0.13; F4w 1.79–1.88, 1.83±0.04; P4 2.55–2.68, 2.62±0.07; TSp 0–0, 0±0; TSpv 2–8, 5±3; TSr 0–0, 0±0; TSrv 10–15, 12.5±2.5; PTl 2.91–3.47, 3.19±0.28; PTw 1.1–1.19, 1.15±0.05; BL 2.52–2.73, 2.62±0.11.

##### Description of female paratype.

*Specimen preparation and condition*. Specimen preserved in 80% EtOH. Spermathecae removed; cleared; in vial with specimen. *General coloration*. Carapace, chelicerae, and legs yellowish red 5YR 4/6. Abdomen reddish black 2.5YR 2.5/1. spinnerets yellowish brown 10YR 5/8. *Cephalothorax*. Carapace 5.37 long, 4.94 wide. Pars cephalica 3.74 long. Foveal groove procurved, 0.56 long, 1.29 wide. Eye tubercle lacking. AER procurved. PER straight. Eye group 0.7 long, 1.52 wide, AME 0.31, PME 0.25, ALE 0.36, PLE 0.32. Sternum sparsely setose, STRl 3.51, STRw 3.1. Chelicerae with anterior row comprising five teeth, posterior margin with seven teeth. Palpal endites with 15 large cuspules across proximal half of endite and 25 smaller cuspules distally, ENDw 1.22, ENDl 1.98. Labium with two large cuspules, LBw 1.12, LBl 0.87. Rastellum with many strong spines on process and up cheliceral face for ~ 1× length of process. *Abdomen*. Evenly setose with pale speckles clustered at two anterior apodeme pairs. *Legs*. F1 3.07; F1w 1.28; P1 2.13; Ti1 1.82; Mt1 1.13; Tr1 0.88; F3 2.89; F3w 1.77; P3 1.78; Ti3 1.64, Sd3 1.04; Mt3 1.15; Tr3 1.19; F4 3.65; F4w 1.84; P4 1.89; Ti4 1.92; Mt4 1.85; Tr4 1.27. Retrolateral face tarsus IV with lacking defined comb or brush. *Pedipalps*. PF 2.75, PP 1.72, PTi 1.55, PTr 1.26. Spermathecae bent medially then anteriorly, bulbs facing anteriorly.

##### Variation, females

**(n = 2).**CL 5.37–5.44, 5.41±0.04; CW 4.94–5.31, 5.13±0.18; Cap 3.74–4.03, 3.89±0.14; ENDl 0.7–0.88, 0.79±0.09; ENDw 1.44–1.52, 1.48±0.04; STRl 3.31–3.51, 3.41±0.1; STRw 3.1–3.15, 3.13±0.02; LBl 0.87–0.93, 0.9±0.03; LBw 1.12–1.26, 1.19±0.07; F1 3.07–3.86, 3.46±0.4; F1w 1.28–1.39, 1.34±0.05; P1 2.09–2.13, 2.11±0.02; Ti1 1.82–2.34, 2.08±0.26; Mt1 1.13–1.56, 1.34±0.22; Tr1 0.88–0.93, 0.91±0.03; F3 2.89–3.24, 3.06±0.17; F3w 1.49–1.77, 1.63±0.14; P3 1.78–1.86, 1.82±0.04; Ti3 1.64–1.81, 1.72±0.08; Mt3 1.15–1.35, 1.25±0.1; Tr3 1.19–1.23, 1.21±0.02; F4 3.65–4.06, 3.86±0.2; F4w 1.56–1.84, 1.7±0.14; P4 1.89–2.15, 2.02±0.13; Ti4 1.92–2.14, 2.03±0.11; Mt4 1.85–2.07, 1.96±0.11; Tr4 1.07–1.27, 1.17±0.1; PF 2.75–3.62, 3.18±0.44; PP 1.72–1.93, 1.82±0.1; PTi 1.55–2.18, 1.87±0.31; PTr 1.26–2.06, 1.66±0.4.

##### Material examined.

**Guatemala**: 15.2812 -90.342^9^, 1836 m a.s.l. (UMM0156, 1♂, AMNH); **Mexico: Chiapas**: 5 mi SE Palenque, Mizola Waterfall, 17.4593 -91.975^5^, 264 m a.s.l. (UMM0536, 14.i.1980, 1♀, B Roth, V Roth, AMNH); **Yucatán**: Chichén Itzá, 20.6826 -88.5686^5^, 38 m a.s.l. (UMM0576, 16.vii.1952, 1♀, J Pallister, D Pallister, AMNH); Tekom, 20.0603 -88.2645^1^, 96 m a.s.l. (UMM0158, 17.iv.1940, 1♂, T Sanderson, AMNH).

#### Ummidia zebrina

Taxon classificationAnimaliaAraneaeHalonoproctidae

(F.O. Pickard-Cambridge, 1897)

A32F71F4-0F24-59A9-BF23-FAF026C36F31

Fig. 47 , Map 5

Pachylomerus zebrinus F. O. Pickard-Cambridge, 1897: 9; male holotype (NHMUK1249) from Guatemala, 15.2812 -90.3420^9^, 1836 m a.s.l., coll. FD Godman 1898, deposited in NHMUK, examined.
Ummidia zebrina Ibarra-Núñez et al., 2011: 1186.

##### Diagnosis.

*Ummidia zebrina* can be distinguished from all other species by the presence of pale horizontal bands on the abdomen. Males can be differentiated from all geographically proximate species by having a single spine on tibia I located on the retroventral face of the article and no spines on metatarsus or tarsus I. Males can be further distinguished from all other species by having the embolus distinctly sigmoid, having a strong S shape.

**Figure 47.**
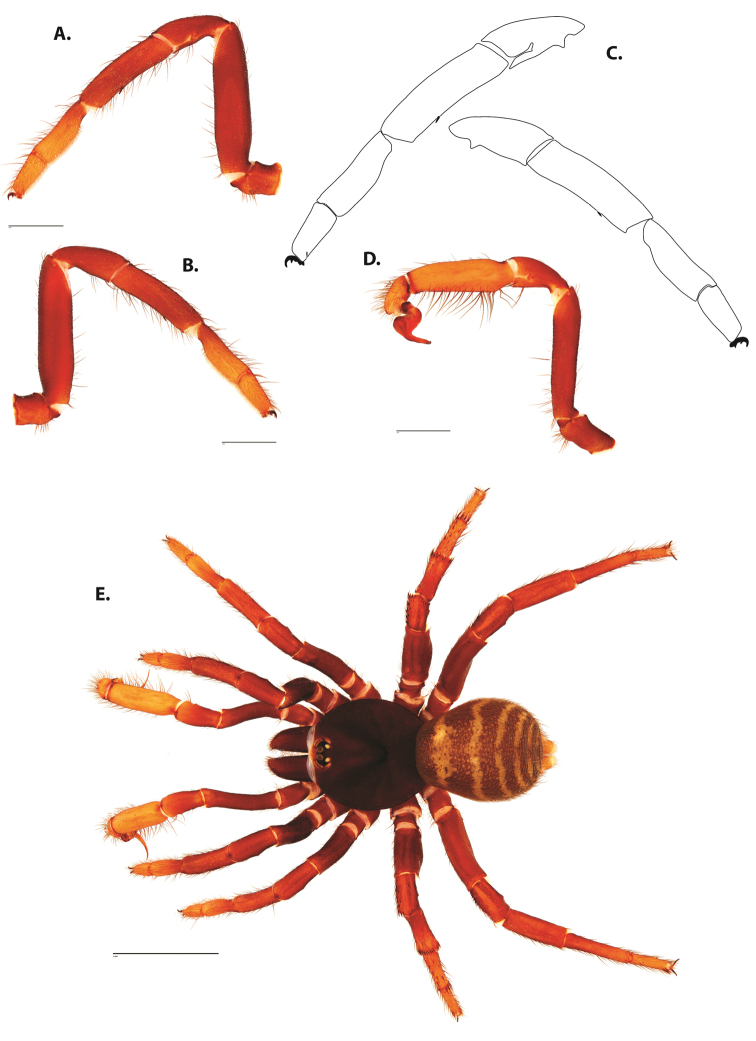
*Ummidia zebrina* (F.O. Pickard-Cambridge, 1897) male holotype (NHMUK1249) from Guatemala. **A** retrolateral aspect, leg I **B** prolateral aspect, leg I **C** line drawings, leg I retrolateral and prolateral aspects **D** retrolateral aspect, pedipalp **E** habitus illustration. Scale bars: 1.0 mm (**A–B**), 4.0 mm (**E**).

##### Description of male holotype.

*Specimen preparation and condition*. Specimen preserved in 80% EtOH. Right leg II Ti-Tr removed, in vial with specimen. *General coloration*. Carapace and chelicerae very dusky red 10R 2.5/2, legs yellowish red 5YR 4/6. Abdomen very dark brown 7.5YR 2.5/3. *Cephalothorax*. Carapace 4.34 long, 4.35 wide. Pars cephalica 2.9 long. Foveal groove procurved, 0.37 long, 0.91 wide. Tubercle moderate. AER procurved. PER straight. Eye group 0.69 long, 1.17 wide, AME 0.29, PME 0.25, ALE 0.41, PLE 0.31. Sternum moderately setose, STRl 2.74, STRw 2.44. Chelicerae with anterior tooth row comprising four teeth, posterior margin with six teeth. Palpal endites with eleven cuspules spread over proximal half of endite face, lacking distal endite cuspules, ENDw 0.97, ENDl 1.86. Labium with six cuspules, LBw 1, LBl 0.65. Rastellum with many spines. Abdomen setose. *Legs*. F1 3.88; F1w 0.95; P1 2.04; Ti1 2.54; Mt1 1.74; Tr1 1.04; F3 3.01; F3w 1.15; P3 1.54; Ti3 1.59; Sd3 1.07; Mt3 1.59; Tr3 1.25; F4 3.9; F4w 1.17; P4 1.94; Ti4 2.46; Mt4 2.56; Tr4 1.42. Retrolateral face of tarsus IV with defined comb over length of tarsus. Leg I spination pattern: TSp 0, TSpv 0, TSrd 0, TSr 0, TSrv 1, MtSp 0, MtSr 0, TrSp 0, TrSr 0. *Pedipalps*. PTl 2.75, PTw 0.82, Bl 2.2. Embolus long, strongly sinuous, bulb relatively small.

##### Variation, males

**(n = 2).**CL 4.3–4.34, 4.32±0.02; CW 3.87–4.35, 4.11±0.24; Cap 2.83–2.9, 2.86±0.04; ENDl 0.53–1.86, 1.19±0.67; ENDw 0.95–0.97, 0.96±0.01; STRl 2.71–2.74, 2.72±0.01; STRw 2.09–2.44, 2.27±0.17; LBl 0.51–0.65, 0.58±0.07; LBw 0.8–1, 0.9±0.1; F1 3.65–3.88, 3.76±0.11; F1w 0.95–0.99, 0.97±0.02; P1 1.78–2.04, 1.91±0.13; Ti1 2.17–2.54, 2.35±0.18; Mt1 1.41–1.74, 1.57±0.16; Tr1 0.86–1.04, 0.95±0.09; F3 2.68–3.01, 2.84±0.16; F3w 1.06–1.15, 1.1±0.05; P3 1.38–1.54, 1.46±0.08; Ti3 1.42–1.59, 1.5±0.09; Mt3 1.31–1.59, 1.45±0.14; Tr3 1.12–1.25, 1.19±0.07; F4 3.39–3.9, 3.64±0.25; F4w 1.1–1.17, 1.14±0.04; P4 1.62–1.94, 1.78±0.16; Ti4 2.21–2.46, 2.33±0.13; Mt4 2.02–2.56, 2.29±0.27; Tr4 1.12–1.42, 1.27±0.15; TSp 0–0, 0±0; TSpv 0–0, 0±0; TSr 0–0, 0±0; TSrv 1–1, 1±0; PTl 1.98–2.75, 2.36±0.39; PTw 0.79–0.82, 0.8±0.01; BL 1.98–2.2, 2.09±0.11.

##### Females.

Unknown.

##### Material examined.

**Guatemala**: 15.2812 -90.3420^9^, 1836m (NHMUK1249, 1898, 1♂, FD Godman, NHMUK); **Jalapa**: Mataquescuintla, El Carrizal, 14.6665 -90.0998^4^, 1741m (UMM0516, 1.x.1982, 1imm, Fend-Renkes, AMNH); **Mexico: Chiapas**: Cañon del Sumidero National Park, 16.859 -93.0921^7^, 1000m (UMM0686, 1.vi.1990, 1♂, H Howden, A Howden, AMNH).

#### Ummidia riverai sp. nov.

Taxon classificationAnimaliaAraneaeHalonoproctidae

C03A668C-7E79-560D-A185-BF0FAB13AB2E

http://zoobank.org/F68B4F7A-368E-440E-956A-6E5E7B3A5412

Fig. 48 , Map 5

##### Type material.

HOLOTYPE: 1 ♂ (UMM496) from 8 kilometers south of Purulhá, Baja Verapaz, Guatemala, 15.1631 -90.2316^5^, 2167 m a.s.l., 29.v.1991, coll. HF Howden, AMNH.

##### Etymology.

The specific epithet is a patronym in honor of Mario Dary Rivera, and environmentalist and patriarch of Guatemala’s environmental movement. Rivera was instrumental in the creation of the Quetzal Biotope (Biotopo de Mario Dary Rivera) near where the type specimen was collected.

##### Diagnosis.

*Ummidia riverai* males can be distinguished from all other geographically proximate males except *U. rugosa* by the presence of a pale dorsal heart patch on the abdomen and from all geographically proximate species by the presence of a defined comb of alternating long and short spinules on the retrolateral face of tarsus I. Males can be further differentiated from *U. huascazaloya*, *U. anaya*, *U. cuicatec*, *U. brandicarlileae*, *U. zebrina*, *U. yojoa*, and *U. varablanca* by having relatively more spines on the prolateral (19 vs 0-11) and retrolateral (22 vs 1-17) faces of tibia I.

**Figure 48.**
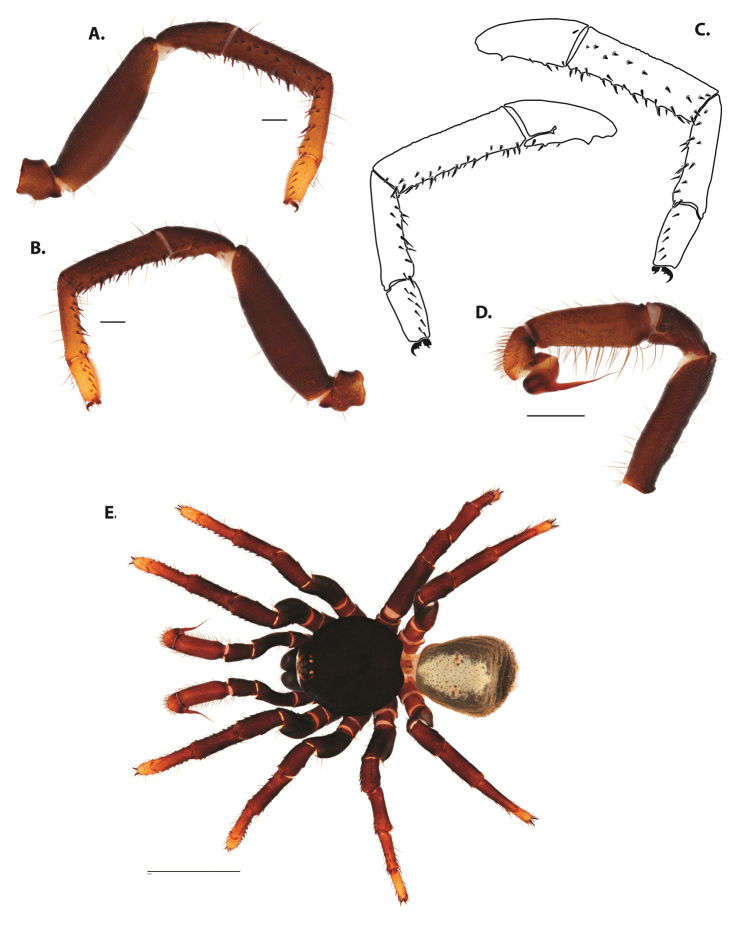
*Ummidia riverai* sp. nov. male holotype (UMM496) from Baja Verapaz, Guatemala. **A** retrolateral aspect, leg I **B** prolateral aspect, leg I **C** line drawings, leg I retrolateral and prolateral aspects **D** retrolateral aspect, pedipalp **E** habitus illustration. Scale bars: 1.0 mm (**A–B**), 4.0 mm (**E**).

##### Description of male holotype.

*Specimen preparation and condition*. Specimen preserved in 80% EtOH. Left palp and leg I removed, in vial with specimen. *General coloration*. Carapace, chelicerae, legs reddish black 10R 2.5/1, tarsi story brown 7.5YR 5/8. Abdomen black 7.5YR 2.5/1 with dorsal heart patch gray 7.5YR 5/1. *Cephalothorax*. Carapace 4.21 long, 4.21 wide. Pars cephalica 2.94 long. Foveal groove procurved, 0.24 long, 0.78 wide. Tubercle high. AER procurved. PER straight. Eye group 0.6 long, 1.06 wide, AME 0.27, PME 0.19, ALE 0.37, PLE 0.2. Sternum sparsely setose around outer 1/3, STRl 2.41, STRw 2.44. Chelicerae with anterior tooth row comprising four teeth, posterior margin with six teeth. Palpal endites with 29 medium and large cuspules spread over proximal 3/4 of endite face, lacking distal endite cuspules, ENDw 1, ENDl 1.54. Labium with eight cuspules, LBw 0.91, LBl 0.66. Rastellum reduced with seven small spines on distal cheliceral margin. Abdomen setose with dorsal heart patch. *Legs*. F1 4.21; F1w 1.16; P1 1.97; Ti1 2.51; Mt1 2.07; Tr1 1.02; F3 2.86; F3w 1.04; P3 1.46; Ti3 1.73; Sd3 1.01; Mt3 1.53; Tr3 1.09; F4 3.63; F4w 1.16; P4 1.7; Ti4 2.1; Mt4 2.24; Tr4 1.14. Retrolateral face of tarsus IV with comb of interspersed long and short hairs. Leg I spination pattern: TSp 10, TSpv 9, TSrd 0, TSr 1, TSrv 22, MtSp 13, MtSr 11, TrSp 5, TrSr 9. *Pedipalps*. PTl 2.06, PTw 0.77, Bl 1.89. Embolus relatively long, slightly sinuous.

##### Variation, males.

Known only from male type specimen.

##### Females.

Unknown.

#### Ummidia frankellerae sp. nov.

Taxon classificationAnimaliaAraneaeHalonoproctidae

07907DF5-CE5B-540A-9952-00C2EB98E2D0

http://zoobank.org/3EB3652D-A200-4C84-91EC-5EEFA5DE771C

Fig. 49 , Map 5

##### Type material.

HOLOTYPE: 1 ♀ (BMEA101781) from Las Cuevas Field Station, 15 kilometers east of Caracol, Cayo, Belize, 16.733 -88.986^1^, 650 m a.s.l., 29.vi-3.vii.2019, coll. JE Bond, BME.

##### Etymology.

The specific epithet is a patronym in honor of Dr. M. Francis Keller. Fran was the leader of the bioblitz in Belize, associated with UC Davis and the Bohart Insect Museum, when this specimen was collected.

##### Diagnosis.

*Ummidia frankellerae* females can be differentiated from all species by the presence of patches on either side of the spinnerets which are, in color and texture, similar to the book lungs and from all other geographically proximate species by the presence of a brush consisting of many spinules embedded in dense setae on the retrolateral face of tarsus IV. Females can be distinguished from all geographically proximate species except *U. pustulosa* by the sternum being relatively longer that wide (1.2× long as wide vs subequal in other species) and from all except *U. cuicatec* by having spermathecae which curve laterally with a distal coil.

**Figure 49.**
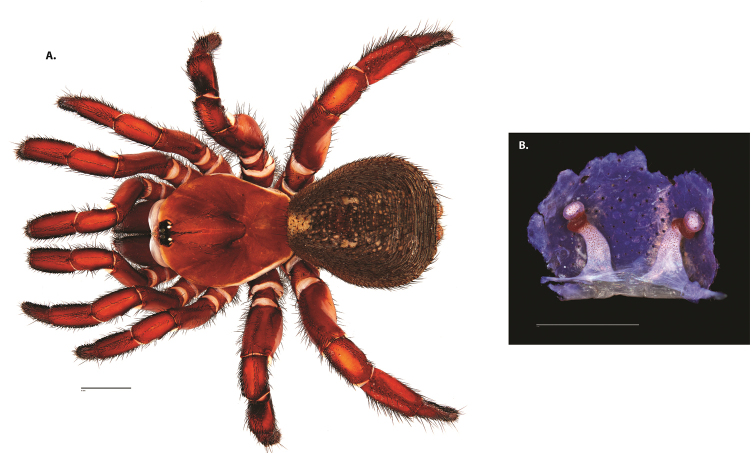
*Ummidia frankellerae* sp. nov. from Cayo, Belize. Female holotype (BMEA101781, BME**A** female habitus illustration **B** cleared spermathecae, Scale bars: 4.0 mm (**A**), 1.0 mm (**B**).

##### Description of female holotype.

*Specimen preparation and condition*. Specimen preserved in 80% EtOH. Spermathecae removed and cleared, in vial with specimen. Right leg III and IV in 100% EtOH. *General coloration*. Carapace, chelicerae, and legs dark reddish brown 5YR 3/4. Abdomen black 7.5YR 2.5/1. spinnerets dark yellowish brown 10YR 4/6. *Cephalothorax*. Carapace 10.88 long, 9.33 wide. Pars cephalica 7.29 long. Foveal groove procurved, 1.07 long, 2.03 wide. All eyes on moderate tubercle. AER procurved. PER straight. Eye group 1.17 long, 2.01 wide, AME 0.44, PME 0.28, ALE 0.64, PLE 0.39. Sternum very setose around outer third and scattered across center STRl 7.12, STRw 5.81. Chelicerae with anterior row comprising seven teeth, posterior margin with seven teeth. Palpal endites with 31 cuspules spread across proximal half of endite and 34 cuspules distally, ENDw 2.47, ENDl 4.19. Labium with eight cuspules, LBw 2.13, LBl 1.43. Rastellum with many strong spines on process and up cheliceral face for ~ 2.5× length of process. Abdomen covered in heavy dark hairs in pustulose sockets; with pale speckles concentrated at apodemes and small pale anterior patch. *Legs*. F1 6.7; F1w 2.42; P1 4.32; Ti1 4.04; Mt1 2.65; Tr1 1.42; F3 5.79; F3w 3.04; P3 4; Ti3 3.36, Sd3 1.85; Mt3 2.68; Tr3 2.32; F4 7.46; F4w 3.38; P4 4.23; Ti4 4.54; Mt4 4.41; Tr4 2.79. Retrolateral face tarsus lacking comb or brush, rather leg IV Mt and Tr with many spinules on PL ad RL faces. *Pedipalps*. PF 6.09, PP 3.65, PTi 3.88, PTr 3.11. Spermathecae tiled laterally with distal coil, bulbs facing anterolaterally.

##### Variation, females.

Known only from female type specimen.

##### Males.

Unknown.

##### Known only from the type material.

#### Ummidia zilchi

Taxon classificationAnimaliaAraneaeHalonoproctidae

Kraus, 1955

AA476AD7-B8AB-5CAF-AE6B-0E0196D9BC1B

Fig. 50 , Map 5

Ummidia zilchi Kraus, 1955b: 7; Type Material. HOLOTYPE 1♂ (8569_1) from San Salvador, El Salvador, 13.6921 -89.219^6^, 700 m a.s.l., A. Zilch, 5.viii.1957, deposited in Senckenberg Museum, examined. Three paratypes (8568, 8570, 8571) associated with specimen. Specimens 8570 and 8571 are immatures and 8568 is misidentified (see Ummidia hondurena sp. nov.)
Ummidia zilchi Bücherl, 1957: 382.

##### Diagnosis.

*Ummidia zilchi* males can be distinguished from all geographically proximate species except *U. huascazaloya* by the palpal tibia being elongate (3.5×long as wide) and a relatively short embolus (half the length of the palpal tibia) with an even curve. Males can be differentiated from all geographically proximate species except *U. zebrina* and *U. carlosviquezi* by having a defined comb of spinules on the retrolateral face of tarsus IV. Males can be further differentiated from *U. huascazaloya*, *U. anaya*, *U. cuicatec*, *U. brandicarlileae*, *U. zebrina*, *U. yojoa*, and *U. varablanca* by having relatively more spines on the prolateral (19 vs 0-11) and retrolateral (36 vs 1-17) faces of tibia I.

**Figure 50.**
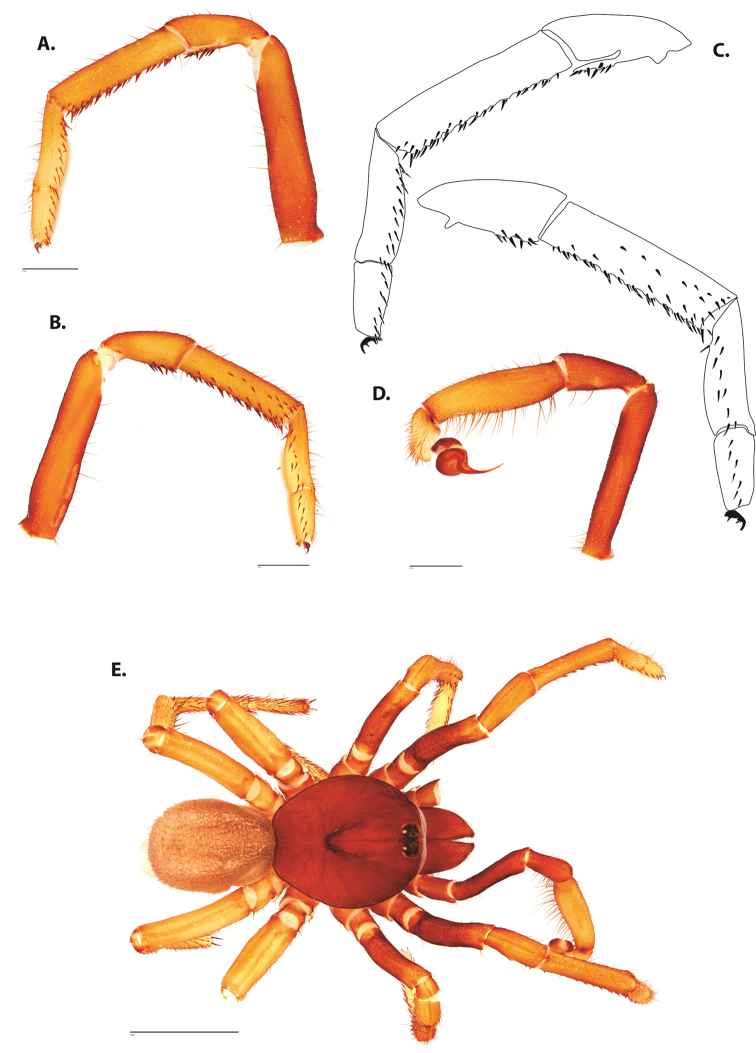
*Ummidia zilchi* (Kraus, 1955) male holotype (SENCK8569_1) from San Salvador, El Salvador. **A** retrolateral aspect, leg I **B** prolateral aspect, leg I **C** line drawings, leg I retrolateral and prolateral aspects **D** retrolateral aspect, pedipalp **E** habitus illustration. Scale bars: 1.0 mm (**A–B**), 4.0 mm (**E**).

##### Description of male holotype.

*Specimen preparation and condition*. Specimen preserved in 80% EtOH. Left palp in vial. Right leg IV Mt-Tr missing. *General coloration*. Carapace and checlicerae dark reddish brown 2.5YR 2.5/4, legs dark yellowish brown 10YR 4/6. Abdomen dark reddish gray 5YR 5/2. *Cephalothorax*. Carapace 5.47 long, 5.04 wide. Pars cephalica 3.55 long. Foveal groove procurved, 0.38 long, 0.83 wide. Tubercle high and well defined. AER procurved. PER straight. Eye group 0.68 long, 1.26 wide, AME 0.35, PME 0.22, ALE 0.44, PLE 0.24. Sternum moderately setose, STRl 3.25, STRw 2.93. Chelicerae with anterior tooth row comprising eight teeth, posterior margin with five teeth. Palpal endites with 23 relatively small cuspules over proximal half of endite face, lacking distal endite cuspules, ENDw 1.34, ENDl 2.29. Labium with three relatively small cuspules, LBw 1.19, LBl 0.64. Rastellum with many spines. Abdomen setose with pale speckles. *Legs*. F1 5.13; F1w 1.1; P1 2.64; Ti1 3.26; Mt1 2.27; Tr1 1.33; F3 3.41; F3w 3.41; P3 1.95; Ti3 2.06; Sd3 1.43; Mt3 2.05; Tr3 1.8; F4 4.61; F4w 1.27; P4 2.08; Ti4 3.01; Mt4 3.34; Tr4 1.68. Retrolateral face of tarsus IV with defined comb over length of tarsus. Leg I spination pattern: TSp 0, TSpv 11, TSrd 0, TSr 2, TSrv 36, MtSp 9, MtSr 17, TrSp 7, TrSr 10. *Pedipalps*. PTl 3.58, PTw 1.06, Bl 1.8. Embolus very short with even curve.

##### Variation, males

**(n = 3).**CL 5.47–7.19, 6.22±0.51; CW 5.04–6.88, 5.84±0.55; Cap 3.55–4.64, 3.99±0.33; ENDl 0.68–0.89, 0.77±0.06; ENDw 1.26–1.63, 1.4±0.12; STRl 3.25–3.96, 3.49±0.24; STRw 2.93–3.97, 3.37±0.31; LBl 0.64–0.97, 0.78±0.1; LBw 0.94–1.65, 1.26±0.21; F1 5.13–6.45, 5.69±0.39; F1w 1.1–1.57, 1.29±0.14; P1 2.63–3.23, 2.83±0.2; Ti1 3.26–4.3, 3.78±0.3; Mt1 2.26–2.63, 2.38±0.12; Tr1 1.17–1.33, 1.27±0.05; F3 3.41–4.53, 3.87±0.34; F3w 1.36–1.83, 1.59±0.14; P3 1.95–2.56, 2.23±0.18; Ti3 2.06–2.56, 2.28±0.15; Mt3 2.04–2.62, 2.24±0.19; Tr3 1.59–1.8, 1.72±0.07; F4 4.61–6.16, 5.25±0.46; F4w 1.27–1.76, 1.45±0.16; P4 2.08–2.71, 2.37±0.18; Ti4 3.01–3.99, 3.47±0.28; Mt4 3.34–4.15, 3.75±0.23; Tr4 1.68–2.15, 1.92±0.14; TSp 9–11, 10±0.58; TSpv 6–11, 8.33±1.45; TSr 0–2, 1.33±0.67; TSrv 22–36, 29±4.04; PTl 3.46–4.16, 3.73±0.22; PTw 1.06–1.3, 1.15±0.07; BL 1.8–2.13, 1.92±0.11.

##### Females.

Unknown.

##### Material examined.

**Belize: La Democracia**: Tropical Education Center, 17.36 -88.537^1^, 30 m a.s.l. (BMEA101782, 22–25.vi.2019, 1♂, JE Bond, BME) **Mexico: Oaxaca**: Palomares, 17.1392 -95.0606^6^, 113 m a.s.l. (UMM0167, vii.1909, 1♂, AMNH); **El Salvador: San Salvador**: San Salvador 13.6921 -89.219^6^, 700 m a.s.l. (8569/1, 5.viii.1957, 1♂, A Zilch, SMF).

#### Ummidia hondurena sp. nov.

Taxon classificationAnimaliaAraneaeHalonoproctidae

99C10AED-43BE-57B3-9DD0-3C9FC0C5B3F8

http://zoobank.org/A12E5E84-3FB2-470F-93CA-82BD88CC5D55

**Figure 51.**
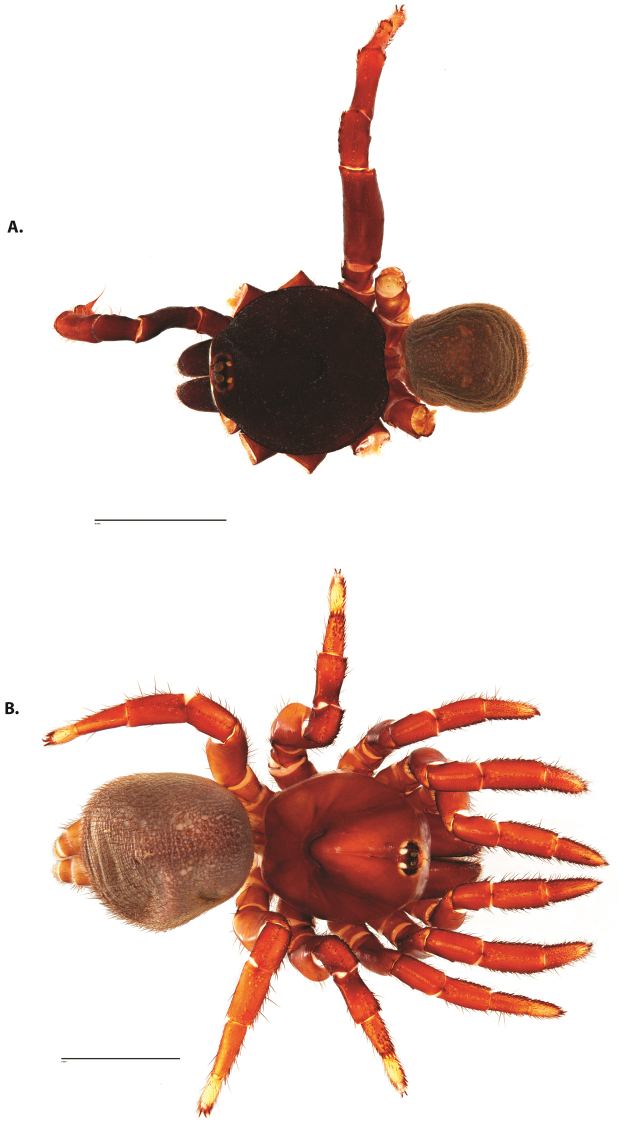
*Ummidia hondurena* sp. nov. from Honduras. **A** male habitus illustration UMM172 **B** female habitus illustration SENCK8568_1. Scale bars: 4.0 mm.

**Figure 52.**
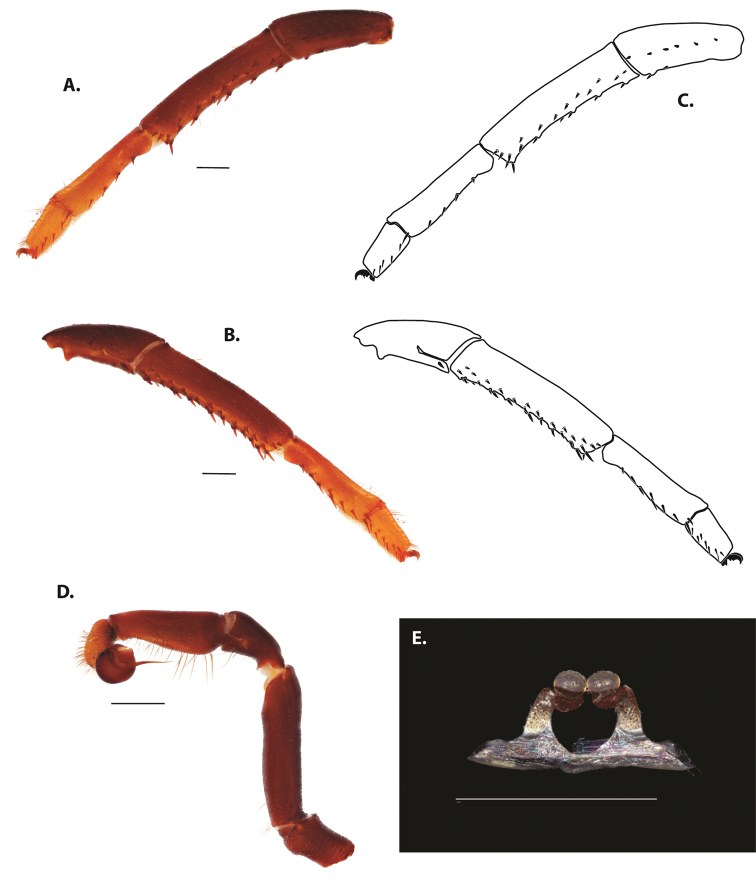
*Ummidia hondurena* sp. nov. from Honduras. **A–D** male holotype (UMM172) **A** prolateral aspect, leg I **B** retrolateral aspect, leg I **C** line drawings, leg I prolateral and retrolateral aspects **D** retrolateral aspect, pedipalp **E** cleared spermathecae female paratype (SENCK8568_1). Scale bars: 1.0 mm.

Figs 51 , 52 , Map 5

##### Type material.

HOLOTYPE: 1 ♂ (UMM172, AMNH) from Honduras, 14.5036 -86.3206^9^, 650 m a.s.l.. PARATYPE: 1 ♀ (8568, SMF) from Finca San Jorge, Santa Ana, Santa Ana, San Salvador, 13.9778 -89.5647^5^, 1000 m a.s.l., 25.iv.1951. Female paratype was originally misidentified as *Ummidia zilchi* (Krauss, 1955).

##### Etymology.

The specific epithet is a noun (feminine, singular) taken in apposition and is in reference to the type locality and its people.

##### Diagnosis.

*Ummidia hondurena* can be differentiated from all other geographically proximate species except *U. riverai* and *U. rugosa* by the presence of a comb of alternating large and small strong setae on the retrolateral face of tarsus IV (Fig. 3C). Males can be differentiated from *U. zilchi*, *U. carlosviquezi*, *U. varablanca*, and *U. quepoa* by possessing a sinuous embolus, rather than one with an even single curve and from *U. yojoa* and *U. varablanca* by having many more prolateral (26 vs 0) and retrolateral (29 vs 2-3) spines on tibia I. Males can be further distinguished from *U. riverai* and *U. rugosa* by lacking a pale dorsal heart patch on the abdomen. Females can be distinguished from all geographically proximate species except *U. rugosa* and *U. erema* by having spermathecae which bend first medially and then anteriorly.

##### Description of male holotype.

*Specimen preparation and condition*. Specimen preserved in 80% EtOH. *General coloration*. Carapace and chelicerae reddish black 2.5YR 2.5/1, legs dark reddish brown 5YR 2.5/2, tarsi yellowish red 5YR 4/6. Abdomen black 10YR 2/1. *Cephalothorax*. Carapace 5.11 long, 4.99 wide. Pars cephalica 3.36 long. Foveal groove procurved, 0.35 long, 0.86 wide. Tubercle moderate. AER procurved. PER straight. Eye group 0.57 long, 1.18 wide, AME 0.32, PME 0.2, ALE 0.46, PLE 0.23. Sternum with posterior fringe, sparsely setose anteriorly, STRl 2.79, STRw 2.54. Chelicerae with anterior tooth row comprising four teeth, posterior margin with six teeth. Palpal endites with 15 cuspules spread over proximal half of endite face, lacking distal endite cuspules, ENDw 0.94, ENDl 1.77. Labium with eight cuspules, LBw 1.01, LBl 0.69. Rastellum with eight spines along distal cheliceral margin, three on process. Abdomen setose. *Legs*. F1 4.73; F1w 1.35; P1 2.13; Ti1 3; Mt1 2.1; Tr1 0.93; F3 3.51; F3w 1.24; P3 1.68; Ti3 2.19; Sd3 1.4; Mt3 1.76; Tr3 1.19; F4 4.48; F4w 1.19; P4 2.02; Ti4 2.76; Mt4 2.73; Tr4 1.14. Retrolateral face of tarsus IV with sparse comb of alternating thicker and thinner spinules. Leg I spination pattern: TSp 2, TSpv 14, TSrd 0, TSr 3, TSrv 26, MtSp 5, MtSr 9, TrSp 5, TrSr 8. *Pedipalps*. PTl 2.09, PTw 0.81, Bl 1.78. Embolus of moderate length, slightly sinuous.

##### Variation, males.

Known only from male type specimen.

##### Description of female paratype.

*Specimen preparation and condition*. Specimen preserved in 80% EtOH. Spermathecae removed, cleared, in vial with specimen. *General coloration*. Carapace; chelicerae; and legs dark brown 7.5YR 3/4, tarsi yellow 10YR 7/6. Abdomen very dark gray 7.5YR 3/1, spinnerets yellowish brown 10YR 7/6. *Cephalothorax*. Carapace 5.43 long, 5.22 wide. Pars cephalica 3.92 long. Foveal groove procurved, 0.51 long, 1.1 wide. Eye tubercle moderate and defined under all eyes. AER procurved. PER straight. Eye group 0.79 long, 1.26 wide, AME 0.29, PME 0.29, ALE 0.45, PLE 0.4. Sternum sparsely setose, thicker at edges. STRl 2.98, STRw 3.11. Chelicerae with anterior row comprising five teeth, posterior margin with six teeth. Palpal endites with 45 large cuspules across proximal half and 12 smaller cuspules distally, ENDw 1.29, ENDl 2.18. Labium with 17 large cuspules with six forming an anterior row, LBw 1.28, LBl 0.96. Rastellum with several large spines along medial and distal margins of process, few small spines above margin. *Abdomen*. Evenly setose and dorsally pustulose. *Legs*. F1 3.76; F1w 1.31; P1 2.27; Ti1 2.14; Mt1 1.41; Tr1 0.86; F3 3.05; F3w 1.57; P3 1.82; Ti3 1.78, Sd3 1.15; Mt3 1.29; Tr3 1.09; F4 3.77; F4w 1.56; P4 2.03; Ti4 1.87; Mt4 1.94; Tr4 0.94. Retrolateral face tarsus IV with defined comb of spinules subtended by small hairs. *Pedipalps*. PF 3.4, PP 1.88, PTi 2.03, PTr 1.76. Spermathecae with medial bend, bulbs facing anteriorly.

##### Variation, females.

Known only from female type specimen.

#### Ummidia yojoa sp. nov.

Taxon classificationAnimaliaAraneaeHalonoproctidae

7F6682EF-A09F-5471-92E8-38B99D67CC64

http://zoobank.org/29E4FB5A-6D03-4635-B9DC-6326655C5443

Fig. 53 , Map 5

##### Type material.

HOLOTYPE: 1 ♂ (UMM186) from Agua Azul, Lake Yojoa, Dept. of Cortés, Honduras, 14.8715 -87.9821^6^, 639 m a.s.l., 30.v.1964, coll. RE Woodruff, AMNH.

##### Etymology.

The specific epithet is a noun taken in apposition and is in reference to the type locality, Lake Yojoa.

##### Diagnosis.

*Ummidia yojoa* males are relatively stocky spiders and can be differentiated from all geographically proximate species by the presences of a soft brush on the retrolateral face of tarsus IV and from all except *U. zebrina* and *U. varablanca* by lacking prolateral spines and having very few retrolateral spines (3 vs 10-44) on tibia I. Males can be further distinguished from *U. zilchi*, *U. carlosviquezi*, *U. varablanca*, and *U. quepoa* by possessing a sinuous embolus, rather than one with an even single curve and from *U. riverai* and *U. rugosa* by lacking a pale dorsal heart patch.

**Figure 53.**
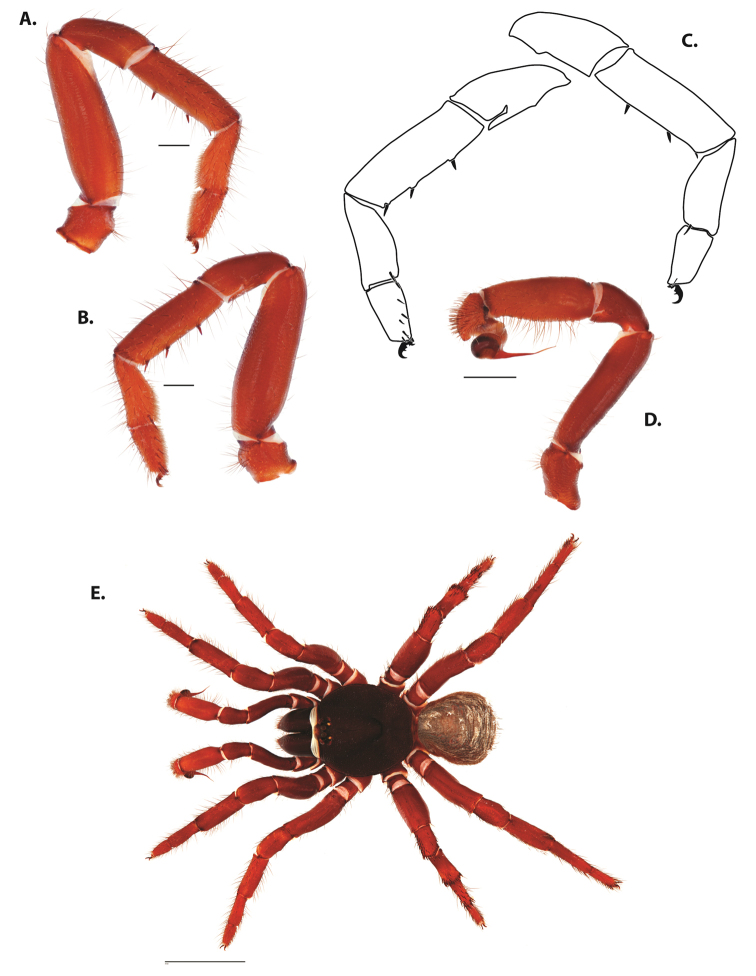
*Ummidia yojoa* sp. nov. male holotype (UMM186) from Dept. of Cortés, Honduras **A** prolateral aspect, leg I **B** retrolateral aspect, leg I **C** line drawings, leg I prolateral and retrolateral aspects **D** retrolateral aspect, pedipalp **E** habitus illustration. Scale bars: 1.0 mm (**A–B**), 4.0 mm (**E**).

##### Description of male holotype.

*Specimen preparation and condition*. Specimen preserved in 80% EtOH. Left palp and leg I removed, in vial with specimen. *General coloration*. Carapace and chelicerae reddish black 2.5YR 2.5/1, legs dark reddish brown 5YR 2.5/3. Abdomen very dark gray 5YR 3/1. *Cephalothorax*. Carapace 4.71 long, 4.51 wide. Pars cephalica 3.31 long. Foveal groove procurved, 0.46 long, 1.03 wide. Tubercle relatively low. AER procurved. PER straight. Eye group 0.64 long, 1.36 wide, AME 0.31, PME 0.17, ALE 0.37, PLE 0.25. Sternum with posterior fringe, sparsely setose anteriorly, STRl 3.03, STRw 2.7. Chelicerae with anterior tooth row comprising four teeth, posterior margin with seven teeth. Palpal endites with 13 cuspules spread over proximal half of endite face, lacking distal endite cuspules, ENDw 1.05, ENDl 1.93. Labium with two cuspules, LBw 0.86, LBl 0.74. Rastellum with many spines on rastellar process. Abdomen setose. *Legs*. F1 3.66; F1w 1.15; P1 1.96; Ti1 2.4; Mt1 1.57; Tr1 1.02; F3 2.95; F3w 1.44; P3 1.49; Ti3 1.51; Sd3 1.05; Mt3 1.49; Tr3 1.45; F4 3.84; F4w 1.35; P4 1.74; Ti4 2.39; Mt4 2.37; Tr4 1.41. Retrolateral face of tarsus IV without brush or comb. Leg I spination pattern: TSp 0, TSpv 0, TSrd 0, TSr 0, TSrv 3, MtSp 1, MtSr 2, TrSp 1, TrSr 6. *Pedipalps*. PTl 2.09, PTw 0.89, Bl 1.84. Embolus of moderate length, sinuous.

##### Variation, males.

Known only from male type specimen.

##### Females.

Unknown.

#### Ummidia matagalpa sp. nov.

Taxon classificationAnimaliaAraneaeHalonoproctidae

87E8A3C5-0EDA-5016-9058-0D4232DF5C5A

http://zoobank.org/E170115A-920E-4F88-BA02-01BD7BE40EF5

Fig. 54 , Map 5

##### Type material.

HOLOTYPE: 1 ♀ (UMM118) from Matagalpa, Matagalpa, Nicaragua, 12.9207 -85.9172^6^, 720 m a.s.l., coll. WB Richardson, AMNH.

##### Etymology.

The specific epithet is a noun taken in apposition and is in reference to the type locality, Matagalpa.

##### Diagnosis.

*Ummidia matagalpa* females can be differentiated from all other geographically proximate species by having spermathecae with only a slight medial curve and from all except *U. zilchi*, *U. carlosviquezi*, and *U. cerrohoya* by the presence of a defined comb on the retrolateral face of tarsus IV.

##### Description of female holotype.

*Specimen preparation and condition*. Specimen preserved in 80% EtOH. Spermathecae removed, cleared, in vial with specimen. *General coloration*. Carapace, chelicerae, and legs very dusky red 10R 2.5/2. Abdomen brown 10YR 4/3, spinnerets dark brown 7.5YR 3/4. *Cephalothorax*. Carapace 10.57 long, 9.76 wide. Pars cephalica 7.72 long. Foveal groove procurved, 1.25 long, 2.59 wide. Eye tubercle moderate tubercle on ME. AER slightly procurved. PER slightly recurved. Eye group 1.09 long, 2.63 wide, AME 0.44, PME 0.39, ALE 0.64, PLE 0.42. Sternum sparsely setose around outer edges; thicker anteriorly, STRl 6.4, STRw 5.93. Chelicerae with anterior row comprising seven teeth, posterior margin with eight teeth. Palpal endites with 23 cuspules spread across proximal half of endite and eleven smaller cuspules distally, ENDw 2.5, ENDl 4.21. Labium with four cuspules, LBw 2.08, LBl 1.74. Rastellum with many strong spines on process and up cheliceral face for ~ ½ × length of process. *Abdomen*. Evenly setose. *Legs*. F1 5.83; F1w 2.55; P1 4.15; Ti1 3.83; Mt1 2.28; Tr1 1.37; F3 5.6; F3w 3.21; P3 3.5; Ti3 3.33, Sd3 2.26; Mt3 2.31; Tr3 2.49; F4 7.17; F4w 3.15; P4 3.95; Ti4 4.2; Mt4 3.7; Tr4 2.53. Retrolateral face tarsus IV with proximal row of doubled spinules and small subtending spinules and defined comb over distal half of tarsus. *Pedipalps*. PF 5.04, PP 3.34, PTi 3.3, PTr 2.84. Spermathecae straight, tilted medially, bulbs facing anteriorly.

**Figure 54.**
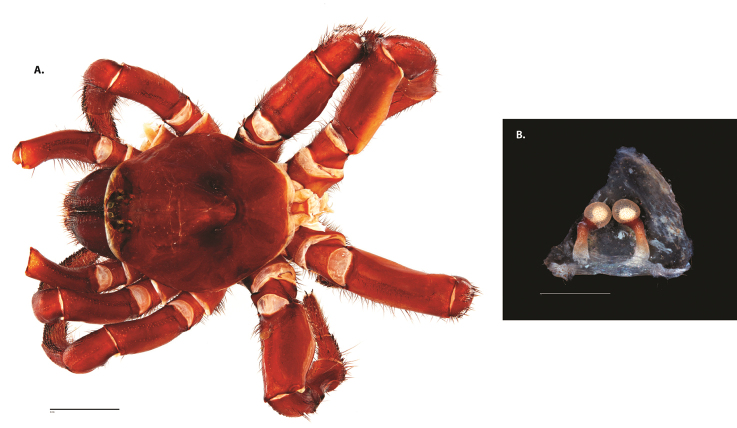
*Ummidia matagalpa* sp. nov. from Matagalpa, Nicaragua. Female holotype (UMM118) **A** female habitus illustration **B** cleared spermathecae. Scale bars: 1.0 mm (**A–B**), 4.0 mm (**E**).

##### Variation, females.

Known only from female type specimen.

##### Males.

Unknown.

#### Ummidia rugosa

Taxon classificationAnimaliaAraneaeHalonoproctidae

(Karsch, 1880)

746E8681-2FDE-5444-8F54-7485C103FA91

**Figure 55.**
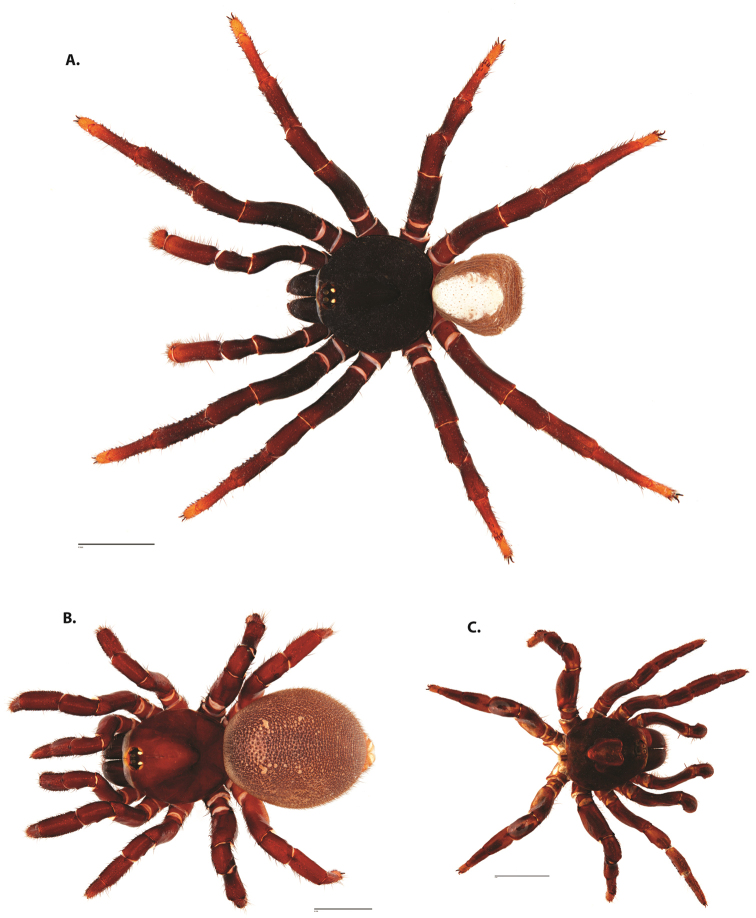
*Ummidia rugosa* (Karsch, 1880) from Costa Rica. **A** male habitus illustration UMM631 **B** female habitus illustration of female exemplar (UMM122) **C** habitus illustration of female holotype (ZMB3244). Scale bars: 4.0 mm.

**Figure 56.**
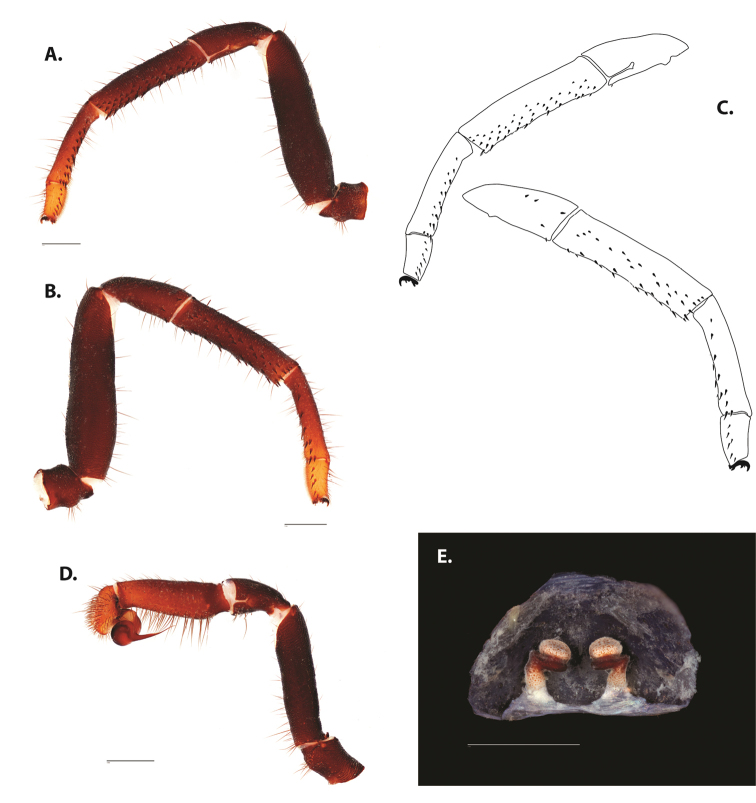
*Ummidia rugosa* (Karsch, 1880) from Costa Rica. A-D male exemplar (UMM631) **A** retrolateral aspect, leg I **B** prolateral aspect, leg I **C** line drawings, leg I retrolateral and prolateral aspects **D** retrolateral aspect, pedipalp **E** cleared spermathecae female exemplar (UMM122). Scale bars: 1.0mm.

Figs 55 , 56 , Map 5

Pachylomerus rugosus Karsch, 1880: 388; female holotype (ZMB3244) from Costa Rica, 9.7703 -83.7751^9^, 1388 m a.s.l., coll Hoffman, deposited in ZMB, examined.
Hebestatis lanthanus Valerio, 1988: 228; male holotype and female allotype from Universidad de Costa Rica, main campus, San Jose Province, Costa Rica, 9.95 -84.0667^1^, 1207m, coll R Aymerich iv.1981, deposited Museo de Zoología, University of Costa Rica, **syn. nov.** We were unable to examine the holotype specimen, but are certain, after examinations of Valerio’s (1988) description and illustrations, and examination of paratype (UMM544) deposited AMNH that these specimens were misidentified as belonging to the genus Hebestatis and that the holotype is actually a male specimen of U. rugosa based on the embolus and presence of a pale dorsal heart patch.

##### Diagnosis.

*Ummidia rugosa* males can be differentiated from all geographically proximate species except *U. riverai* by the presence of a pale dorsal heart patch and all but *U. riverai* and *U. hondurena* by the presence of a comb of alternating long and short heavy hairs on the retrolateral face of tarsus IV. Males can be distinguished from *U. zilchi*, *U. carlosviquezi*, *U. varablanca*, and *U. quepoa* by possessing a sinuous embolus, rather than one with an even single curve and from *U. yojoa* and *U. varablanca* by the presence of many prolateral (28 vs 0) and retrolateral (42 vs 2-3) spines on tibia I. Females can be distinguished from all geographically proximate species except *U. hondurena* and *U. erema* by having spermathecae which bend first medially and then anteriorly.

##### Remarks.

The abdomen of the female holotype specimen of *Ummidia rugosa* is missing. For this reason, we have also included a description of a female exemplar.

##### Description of female holotype.

*Specimen preparation and condition*. Specimen preserved in 80% EtOH. Specimen in extremely poor condition, previously pinned, abdomen and spermathecae missing. Many limbs broken. *General coloration*. Carapace, chelicerae, and legs reddish black 2.5YR 2.5/1. *Cephalothorax*. Carapace 5.84 long, 5.45 wide. Pars cephalica 4.62 long. Foveal groove procurved, 0.69 long, 1.45 wide. Eye tubercle moderate. AER procurved. PER recurved. Eye group 0.9 long, 1.51 wide, AME 0.38, PME 0.32, ALE 0.57, PLE 0.36. Sternum with outer fringe of setae only, STRl 3.34, STRw 3.37. Chelicerae with anterior row comprising five teeth, posterior margin with eight teeth. Palpal endites with 37 very large cuspules spread across proximal half of endite face and 21 cuspules distally, ENDw 1.4, ENDl 1.13. Labium with 12 very large cuspules, LBw 0.97, LBl 1.13. Rastellum with ten spines along distal cheliceral margin and four medially on process. *Legs*. F1 4.28; F1w 1.52; P1 2.43; Ti1 2.26; Mt1 1.69; Tr1 0.93; F3 3.6; F3w 1.71; P3 2.15; Ti3 2.08, Sd3 1.4; Mt3 1.37; Tr3 1.01; F4 4.45; F4w 1.88; P4 2.43; Ti4 2.39; Mt4 2.2; Tr4 1.25. Retrolateral face tarsus IV with defined comb of alternating long and short spinules. *Pedipalps*. PF 4.05, PP 2.15, PTi 2.17, PTr 2.23.

##### Description of female exemplar.

*Specimen preparation and condition*. Specimen preserved in 80% EtOH. Spermathecae removed, cleared, in vial with specimen. *General coloration*. Carapace, chelicerae, and legs very dusky red 10R 2.5/2. Abdomen very dark brown 10YR 2/2, spinnerets dark yellowish brown 10YR 4/6. *Cephalothorax*. Carapace 7.25 long, 6.93 wide. Pars cephalica 5.23 long. Foveal groove procurved, 0.56 long, 1.4 wide. Eye tubercle low under ME. AER procurved. PER straight. Eye group 1.03 long, 1.73 wide, AME 0.33, PME 0.34, ALE 0.61, PLE 0.47. Sternum with posterior fringe, sparsely setose anteriorly, STRl 4.13, STRw 4.18. Chelicerae with anterior row comprising five teeth, posterior margin with six teeth. Palpal endites with 33 very large cuspules spread across proximal half of endite face and 48 smaller cuspules distally, ENDw 1.89, ENDl 3.07. Labium with eleven large cuspules, LBw 1.69, LBl 1.21. Rastellum reduced with long rounded spines along margin and smaller spines on process. *Abdomen*. Evenly setose with pale speckles concentrated at apodemes and with dorsal opalescence. *Legs*. F1 4.98; F1w 1.77; P1 2.93; Ti1 2.99; Mt1 2.07; Tr1 1.07; F3 4.24; F3w 2.07; P3 2.41; Ti3 2.2, Sd3 1.48; Mt3 1.83; Tr3 1.52; F4 5.34; F4w 2.01; P4 2.7; Ti4 2.71; Mt4 2.7; Tr4 1.3. Retrolateral face tarsus IV with comb of alternating long and short spinules. *Pedipalps*. PF 4.59, PP 2.53, PTi 2.73, PTr 2.55. Spermathecae bent medially then coiled anteriorly, bulbs facing anteriorly.

##### Variation, females

**(n = 13).**CL 4.73–8.77, 6.59±0.36; CW 4.55–8.79, 6.29±0.35; Cap 3.45–6.3, 4.81±0.26; ENDl 0.65–1.21, 0.93±0.04; ENDw 1.16–2.11, 1.55±0.08; STRl 2.73–5.16, 3.77±0.21; STRw 2.82–5.04, 3.76±0.19; LBl 0.78–1.41, 1.1±0.06; LBw 0.97–1.84, 1.45±0.08; F1 3.17–6.21, 4.59±0.25; F1w 1.17–2.13, 1.61±0.08; P1 1.87–3.71, 2.61±0.14; Ti1 1.8–3.67, 2.67±0.16; Mt1 1.13–2.59, 1.86±0.11; Tr1 0.78–1.46, 1.01±0.05; F3 2.78–5.2, 3.87±0.22; F3w 1.34–2.49, 1.85±0.1; P3 1.61–3.04, 2.22±0.12; Ti3 1.48–2.93, 2.09±0.12; Mt3 1.13–2.22, 1.63±0.1; Tr3 1.01–1.73, 1.34±0.08; F4 3.22–6.36, 4.71±0.27; F4w 1.34–2.41, 1.88±0.1; P4 1.75–3.35, 2.5±0.14; Ti4 1.77–3.24, 2.49±0.15; Mt4 1.65–3.18, 2.39±0.15; Tr4 1–1.68, 1.27±0.06; PF 2.89–5.69, 4.26±0.24; PP 1.59–3.06, 2.25±0.13; PTi 1.57–3.39, 2.41±0.16; PTr 1.71–3.22, 2.33±0.13.

##### Description of male exemplar.

*Specimen preparation and condition*. Specimen preserved in 80% EtOH. *General coloration*. Carapace, chelicerae, and legs reddish black 2.5YR 2.5/1, tarsi yellowish red 5YR 4/6. Abdomen very dark gray 7.5YR 3/1 with heart patch light gray 5Y 7/1. *Cephalothorax*. Carapace 6.14 long, 6.38 wide. Pars cephalica 4.41 long. Foveal groove procurved, 0.52 long, 1.22 wide. All eyes on moderate tubercle. AER procurved. PER straight. Eye group 0.82 long, 1.51 wide, AME 0.27, PME 0.25, ALE 0.45, PLE 0.24. Sternum sparsely setose, STRl 3.56, STRw 3.61. Chelicerae with anterior tooth row comprising four teeth, posterior margin with five teeth. Palpal endites with 30 cuspules spread over proximal 3/4 of endite face, lacking distal endite cuspules, ENDw 1.43, ENDl 2.36. Labium with nine cuspules, LBw 1.34, LBl 0.85. Rastellum with many small rounded spines concentrated along distal margin. Abdomen setose, setation reduced over heart patch. *Legs*. F1 6.13; F1w 1.61; P1 2.97; Ti1 4.06; Mt1 3; Tr1 1.12; F3 4.43; F3w 1.52; P3 2.21; Ti3 2.53; Sd3 1.54; Mt3 2.51; Tr3 1.38; F4 5.51; F4w 1.47; P4 2.52; Ti4 3.46; Mt4 3.54; Tr4 1.73. Retrolateral face of tarsus IV with loose comb surrounded in setae. Leg I spination pattern: TSp 12, TSpv 16, TSrd 0, TSr 5, TSrv 37, MtSp 9, MtSr 17, TrSp 6, TrSr 9. *Pedipalps*. PTl 2.96, PTw 1.05, Bl 2.41. Embolus sinuous.

##### Variation, males

**(n = 7).**CL 4.42–7, 5.47±0.36; CW 4.52–7.25, 5.64±0.37; Cap 3.04–4.76, 3.82±0.24; ENDl 0.62–0.91, 0.74±0.04; ENDw 1.19–1.59, 1.37±0.06; STRl 2.46–3.81, 3.08±0.19; STRw 2.39–3.73, 3.06±0.2; LBl 0.67–0.94, 0.81±0.03; LBw 0.95–1.34, 1.12±0.06; F1 4.18–6.19, 5.12±0.31; F1w 0.16–1.88, 1.28±0.21; P1 2.03–3.1, 2.5±0.16; Ti1 2.82–4.11, 3.38±0.2; Mt1 1.99–3, 2.49±0.15; Tr1 0.9–1.21, 1.05±0.05; F3 3.18–4.43, 3.72±0.2; F3w 1.11–1.77, 1.36±0.09; P3 1.49–2.43, 1.9±0.13; Ti3 1.86–2.55, 2.17±0.11; Mt3 1.7–2.51, 2.03±0.12; Tr3 0.98–1.39, 1.24±0.06; F4 3.79–5.87, 4.72±0.29; F4w 1.05–1.62, 1.33±0.08; P4 1.73–2.71, 2.17±0.15; Ti4 2.41–3.58, 2.9±0.18; Mt4 2.44–3.58, 2.99±0.18; Tr4 1.15–1.73, 1.34±0.08; TSp 6–26, 12.29±2.67; TSpv 2–18, 10±2.09; TSr 1–6, 3.43±0.65; TSrv 14–37, 26.14±3.58; PTl 1.98–3.01, 2.49±0.15; PTw 0.8–1.15, 0.95±0.05; BL 1.7–2.41, 2.08±0.1.

##### Material examined.

**Costa Rica**: 9.7703 -83.7751^9^, 1388 m a.s.l. (ZMB3244, 1♀, Hoffman, ZMB); **Alajuela**: Upala, 10.898 -85.0195^1^, 300–400 m a.s.l. (UMM0682, 24.ii-28.iv.2013, 1♂, C Chacoa, BME); Volcán Tenorio National Park, El Pilón Station, 10.7153 -84.9923^1^, 809 m a.s.l. (UMM0754, 1♀, J Valverde, BME); **Cartago**: Chitaría, Turrialba, 9.9327 -83.5938^5^, 721 m a.s.l. (UMM0122, 12.viii.1982, 1♀, LD Gomez, AMNH); Jardín Lankester, Las Concavas, 9.8395 -83.8902^4^, 1368 m a.s.l. (UMM0226, 7.xi.1979, 1♀, O Rodrigues, AMNH); Tres Ríos, La Carpintera, 9.8832 -83.9833^6^, 1836 m a.s.l. (UMM0119, 13.iv.1982, 1♀, CE Valerio, AMNH); (UMM0137, 16.v.1983, 1♀, M Boza, AMNH); **Guanacaste**: Cerro Pedregal, 10.9308 -85.4825^1^, 731 m a.s.l. (UMM0680, xi-xii.1990, 1♂, BME); Estación Cacao, 10.9291 -85.4952^1^, 1100 m a.s.l. (UMM0759a, vii.1989-iii.1990, 1♂, C Moraga, BME); Guanacaste Conservation Area Liberia, Santa Rosa National Park, Santa Rosa Station, 10.8364 -85.6155^1^, 296 m a.s.l. (UMM0749, 1–31.i.1991, 1♀, D Janzen, BME); Palo Verde, 10.3828 -85.3351^5^, 130 m a.s.l. (UMM0115, 16–22.i.1978, 1♀, CE Valerio, CM Zuniga, AMNH); Pitilla Station, 9 km S Santa Cecilia, 10.9904 -85.4272^1^, 692 m a.s.l. (UMM0752, 29.iii-5.iv.1995, 1♀, C Moraga, BME); **Puntarenas**: Monteverde, 10.3069 -84.8097^5^, 1427 m a.s.l. (UMM0215, 10.v.1970, 1♀, R Buskirk, AMNH); **San José**: Monte de la Cruz, 9.855 -84.1373^5^, 2091 m a.s.l. (UMM0466, 5.iii.1988, 1♀, G Barrantes, AMNH); (UMM0467, 5.iii.1988, 1♀, O Pearson, AMNH); Grandilla, 9.9295 -84.0193^5^, 1290 m a.s.l. (UMM0631, 26.v.1986, 1♂, CE Valerio, AMNH); San Pedro, La Granja, 9.9275 -84.0487^5^, 1212 m a.s.l. (UMM0544, 4.iv.1983, 1♂, P Benedetto, AMNH); Cerro Escazú, 9.8941 -84.1683^1^, 1444 m a.s.l. (UMM0744, x.1996, 1♀, C Flores, BME); San Antonio de Escazú, 9.8919 -84.1368^5^, 1426 m a.s.l. (UMM0478, 2.vi.1983, 1♂, W Eberhard, AMNH); **Nicaragua: Granada**: San Joaquín, Volcán Mombacho, organic coffee plantation, 12.4219 -86.5448^1^, 700–800 m a.s.l. (UMM0684, 1998–1999, 1♂, JM Maes, BME).

#### Ummidia carlosviquezi sp. nov.

Taxon classificationAnimaliaAraneaeHalonoproctidae

3AC7CFB2-651C-58B7-8265-435A739CA573

http://zoobank.org/299C26B2-6D75-438E-B23D-03795ADF968E

**Figure 57.**
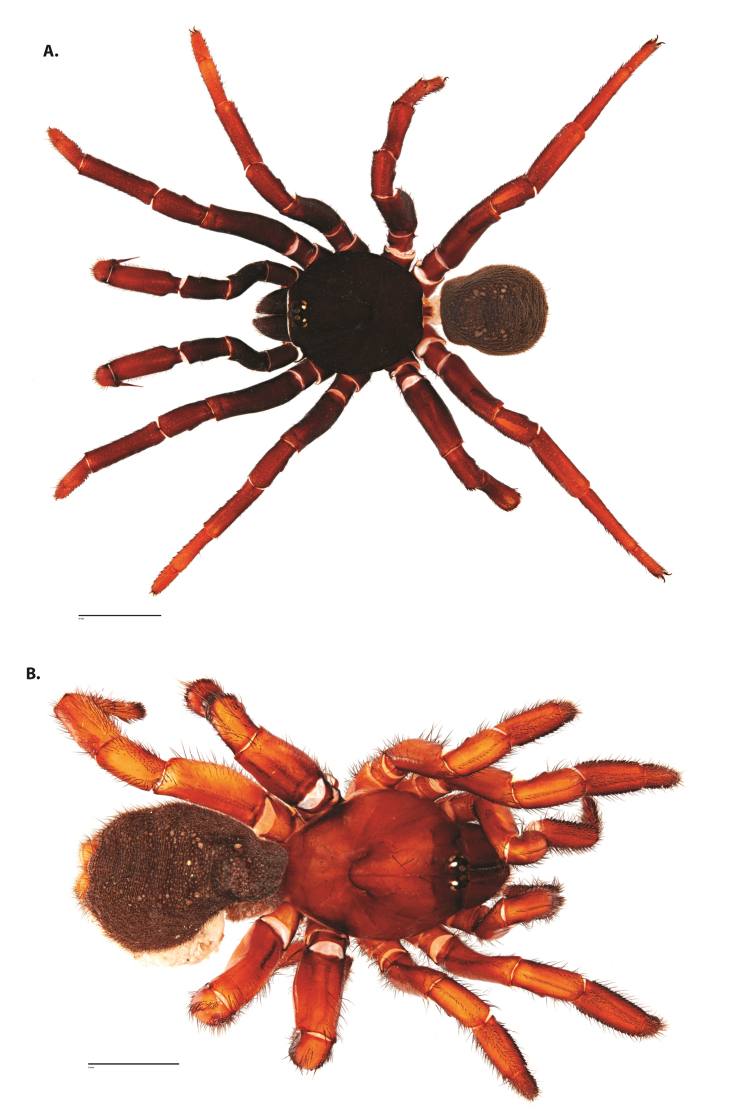
*Ummidia carlosviquezi* sp. nov. from Puntarenas, Costa Rica. **A** male habitus illustration UMM239 **B** female habitus illustration UMM747. Scale bars: 4.0 mm.

**Figure 58.**
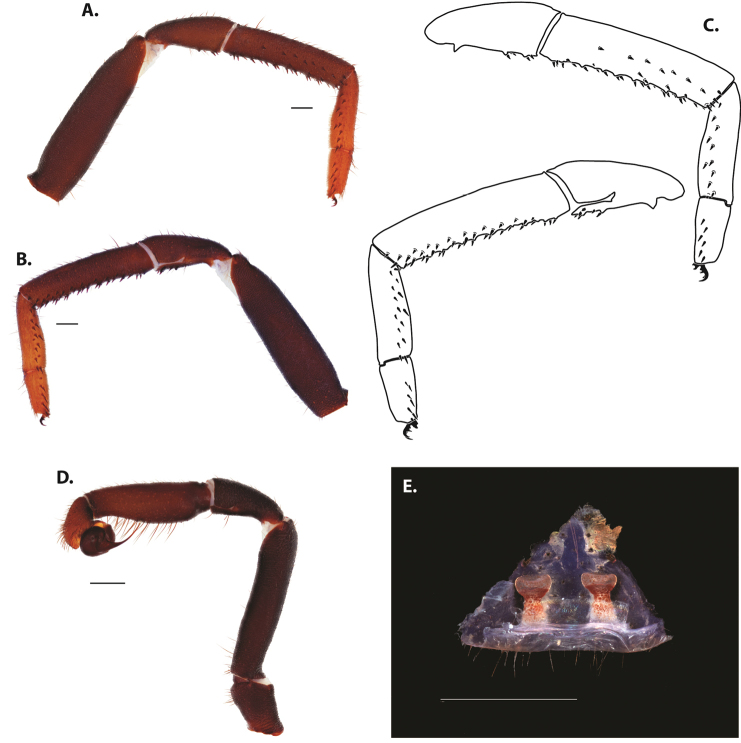
*Ummidia carlosviquezi* sp. nov. from Puntarenas, Costa Rica **A–D** male holotype (UMM239) **A** prolateral aspect, leg I **B** retrolateral aspect, leg I **C** line drawings, leg I prolateral and retrolateral aspects **D** retrolateral aspect, pedipalp **E** cleared spermathecae female paratype (UMM747). Scale bars: 1.0 mm.

Figs 57 , 58 , Map 5

##### Type material.

HOLOTYPE: 1 ♂ (UMM239) from 6 kilometers south of San Vito, Puntarenas, Costa Rica, 8.7125 -83.0051^1^, 355 m a.s.l., 13–18.iii.1967, coll. C Valerio, AMNH. PARATYPE: 1 ♀ (UMM747) from Las Cruces, Puntarenas, Costa Rica, 8.7919 -82.9551^1^, 1227 m a.s.l. coll. C Viquez, P Jordan 7–8.xi.2001, BME.

##### Etymology.

The specific epithet is a patronym in honor of Carlos Viquez, a Costa Rican arachnologist who assisted the authors in the field and provided a great deal of the Costa Rican material used in this revision, to include one of the type specimens for this species.

##### Diagnosis.

*Ummidia carlosviquezi* males can be distinguished from *U. hondurena*, *U. yojoa*, *U. rugosa*, and *U. erema* by having a relatively short embolus with a single even curve and from *U. yojoa* and *U. varablanca* by the presence of many prolateral (22 vs 0) and retrolateral (35 vs 2-3) spines on tibia I. Males can be further differentiated from *U. rugosa* and *U. riverai* by the lack of a pale dorsal heart patch on the abdomen and from *U. matagalpa* and *U. quepoa* by the presence of a comb on the retrolateral face of tarsus IV. Females can be distinguished from all geographically proximate species except *U. cerrohoya* by having straight, simple spermathecae.

##### Description of male holotype.

*Specimen preparation and condition*. Specimen preserved in 80% EtOH. Left palp and leg I, right leg IV removed, in vial with specimen. *General coloration*. Carapace, chelicerae, and legs reddish black 10R 2.5/1, tarsi yellowish red 5YR 4/6. Abdomen reddish black 2.5YR 2.5/1. *Cephalothorax*. Carapace 6.7 long, 6.18 wide. Pars cephalica 4.13 long. Foveal groove procurved, 0.4 long, 1.12 wide. All eyes on moderate tubercle. AER procurved. PER recurved. Eye group 0.85 long, 1.39 wide, AME 0.35, PME 0.25, ALE 0.48, PLE 0.36. Sternum sparsely setose around outer 1/3, thicker anteriorly, STRl 3.73, STRw 3.49. Chelicerae with anterior tooth row comprising six teeth, posterior margin with six teeth. Palpal endites with 21 cuspules spread over proximal half of endite face, and four cuspules distally, ENDw 1.43, ENDl 2.53. Labium with five cuspules, LBw 1.37, LBl 0.91. Rastellum with many spines on process. Abdomen setose. *Legs*. F1 5.96; F1w 1.58; P1 3.1; Ti1 4.34; Mt1 2.6; Tr1 1.42; F3 4.33; F3w 1.66; P3 2.16; Ti3 2.4; Sd3 1.29; Mt3 2.47; Tr3 1.85; F4 5.55; F4w 1.66; P4 2.46; Ti4 3.68; Mt4 3.8; Tr4 1.94. Retrolateral face of tarsus IV with defined comb over length of tarsus. Leg I spination pattern: TSp 10, TSpv 11, TSrd 0, TSr 1, TSrv 34, MtSp 11, MtSr 15, TrSp 6, TrSr 8. *Pedipalps*. PTl 3.45, PTw 1.19, Bl 2.02. Embolus relatively short with even curve.

##### Variation, males

**(n = 14).**CL 5.17–7.82, 6.51±0.21; CW 4.69–7.41, 6.05±0.21; Cap 3.4–5.24, 4.16±0.15; ENDl 0.66–0.93, 0.79±0.02; ENDw 1.26–1.99, 1.45±0.05; STRl 2.93–4.43, 3.63±0.12; STRw 2.65–4.19, 3.28±0.13; LBl 0.65–1.12, 0.89±0.04; LBw 1.02–1.61, 1.29±0.05; F1 4.07–7.19, 5.78±0.22; F1w 1.14–1.8, 1.4±0.05; P1 2.15–3.39, 2.82±0.1; Ti1 2.58–4.88, 3.85±0.17; Mt1 1.78–3.02, 2.45±0.09; Tr1 1–1.42, 1.24±0.03; F3 3.15–5.06, 4.2±0.15; F3w 1.37–2.11, 1.63±0.06; P3 1.62–2.61, 2.16±0.08; Ti3 1.77–2.97, 2.42±0.09; Mt3 1.5–2381, 172.29±169.9; Tr3 1.24–2.02, 1.75±0.05; F4 4.11–6.93, 5.63±0.22; F4w 1.27–1.99, 1.54±0.05; P4 2–2.96, 2.44±0.08; Ti4 2.62–4.83, 3.67±0.16; Mt4 2.61–5.07, 3.95±0.18; Tr4 1.49–2.29, 1.86±0.06; TSp 0–13, 3.64±1.13; TSpv 0–26, 8.43±1.92; TSr 0–11, 1.36±0.75; TSrv 9–38, 25.86±2.13; PTl 2.2–4.07, 3.37±0.15; PTw 0.85–1.35, 1.17±0.04; BL 1.59–2.34, 1.9±0.05.

##### Description of female paratype.

*Specimen preparation and condition*. Specimen preserved in 80% EtOH. Spermathecae removed, cleared, in vial with specimen. *General coloration*. Carapace, chelicerae, and legs dark reddish brown 5YR 3/4. Abdomen black 7.5YR 2.5/1, spinnerets dark yellowish brown 10YR 4/6. *Cephalothorax*. Carapace 8.71 long, 7.08 wide. Pars cephalica 5.46 long. Foveal groove procurved, 0.79 long, 1.63 wide. Eye tubercle moderate under ME. AER procurved. PER straight. Eye group 0.79 long, 1.63 wide, AME 0.44, PME 0.38, ALE 0.62, PLE 0.39. Sternum sparsely setose around edges, thicker anteriorly STRl 4.99, STRw 4.58. Chelicerae with anterior row comprising six teeth, posterior margin with seven teeth. Palpal endites with 30 cuspules spread across proximal half of endite and 56 smaller cuspules distally, ENDw 1.81, ENDl 3.09. Labium with eight cuspules, LBw 1.76, LBl 1.41. Rastellum with strong spines along margin and bottom half of process with smaller spines on upper half of process and up cheliceral face ~ 1.5× length of process. *Abdomen*. Evenly setose with pale speckles clustered at apodemes. *Legs*. F1 5.37; F1w 1.89; P1 3.25; Ti1 3.25; Mt1 2.1; Tr1 1.25; F3 4.78; F3w 2.14; P3 2.74; Ti3 2.47, Sd3 1.51; Mt3 2.04; Tr3 1.83; F4 5.68; F4w 2.45; P4 2.77; Ti4 3.49; Mt4 3.27; Tr4 1.96. Retrolateral face tarsus IV with distinct comb over central half of tarsus becoming loose distally. *Pedipalps*. PF 4.85, PP 2.84, PTi 3.11, PTr 2.7. Spermathecae simple and straight, bulbs facing anteriorly.

##### Variation, females

**(n = 8).**CL 4.2–9.79, 7.03±0.72; CW 3.86–9.13, 6.3±0.63; Cap 2.87–6.75, 4.73±0.47; ENDl 0.58–1.21, 0.9±0.08; ENDw 1.09–2.05, 1.56±0.11; STRl 2.34–5.98, 4.19±0.43; STRw 2.45–5.69, 4.01±0.38; LBl 0.72–1.63, 1.12±0.11; LBw 0.91–2.11, 1.49±0.14; F1 2.61–6.32, 4.39±0.49; F1w 1.02–2.11, 1.55±0.15; P1 1.57–3.85, 2.72±0.3; Ti1 1.43–3.9, 2.61±0.32; Mt1 1.01–2.69, 1.75±0.21; Tr1 0.85–1.58, 1.16±0.09; F3 2.24–5.15, 3.79±0.38; F3w 1.21–2.65, 1.95±0.18; P3 1.32–3.38, 2.28±0.25; Ti3 1.21–3, 2.08±0.22; Mt3 0.98–2.28, 1.67±0.17; Tr3 0.84–2.37, 1.64±0.18; F4 2.94–6.58, 4.69±0.43; F4w 1.22–6.47, 2.46±0.59; P4 1.52–3.46, 2.34±0.21; Ti4 1.63–3.78, 2.76±0.28; Mt4 1.63–3.97, 2.72±0.3; Tr4 1.13–3.55, 1.78±0.28; PF 2.26–5.84, 4±0.46; PP 1.35–3.27, 2.33±0.25; PTi 1.39–3.76, 2.51±0.32; PTr 1.16–3.39, 2.25±0.28.

**Costa Rica: Cartago**: Cantón-Central, 9.7156 -83.7503^9^, 1743 m a.s.l. (UMM0587, 20.iv.1973, 1♂, Araya, Rodolfo, AMNH); **Guanacaste**: Estación Cacao, 10.9291 -85.4952^1^, 1100 m a.s.l. (UMM0759b, vii.1989-iii.1990, 1♂, C Moraga, BME); Guanacaste Conservation Area, La Cruz, Guanacaste National Park, Maritza Station, 10.9625 -85.4952^1^, 600 m a.s.l. (UMM0761, 1–31.v.1996, 1♂, M Pereira, BME); La Cruz, 9 km S Santa Cecilia, Pitilla Station, 10.9904 -85.4272^1^, 700 m a.s.l. (UMM0760, 1–31.x.1996, 1♂, C Moraga, BME); Tenorio National Park, Volcán Tenorio, 10.6764 -85.0225^1^, 1661 m a.s.l. (UMM0756, 4.xi.2003–15.ii.2004, 1♂, A Azofeifa, BME); **Heredia**: Santa Rosa, Cacao, 9.9705 -84.1^1^, 1114 m a.s.l. (UMM0671, 1♂, BME); **Limón**: Barbilla, 10.0695 -83.3675^1^, 22 m a.s.l. (UMM0753, 20–24.x.2000, 1♀, Mantillo, BME); Hitoy Cerere, 9.6436 -83.0711^1^, 364 m a.s.l. (UMM0679, 1♂, BME); R.B. Hitoy Cerere. Send. Espavel, 9.6614 -83.0307^1^, 560 m a.s.l. (UMM0763, 18.ix-3.x.2003, 1♂, W Arana, B Gamboa, E Rojas, BME); Río Yorkin and Río Telire, 9.5599 -82.924^1^, 1300 m a.s.l. (UMM0677, 24–26.x.2008, 1♂, A Solis, BME); **Puntarenas**: Platanares, 9.138 -83.2777^1^, 413 m a.s.l. (UMM0678, 16.vii-4.viii.2001, 1♂, R Gonzálz, BME); 6km S San Vito, 8.7125 -83.0051^1^, 355 m a.s.l. (UMM0239, 13–18.iii.1967, 1♂, AMNH); Gulfito, 8.6391 -83.1625^5^, 112 m a.s.l. (UMM0120, iv.1972, 1♀, ICECU, AMNH); Las Cruces, 8.7919 -82.9551^1^, 1227 m a.s.l. (UMM0747, 1♀, BME); **San José**: (UMM0624, 5.iv.1980, 1♀, AMNH); Grandilla, 9.9295 -84.0193^5^, 1290 m a.s.l. (UMM0545, 10.vi.1986, 1♀, CE Valerio, AMNH); BME building, 9.973 -84.0937^1^, 1127 m a.s.l. (UMM0672, 4.v.2001, 1♂, A Solis, BME); La Cangreja National Park, Ecotropica Station, 9.6883 -84.3692^1^, 300–400 m a.s.l. (UMM0758, 13–17.vii.2004, 1♂, Porras, Cárdenas, Gamboa, Briceno, Moraga, Mata, BME); La Congreja National Park, 9.7032 -84.3972^1^, 448 m a.s.l. (UMM0748, 1♀, BME); La Fonda, 10.0495 -84.0077^1^, 1606 m a.s.l. (UMM0745, 1♀, BME); San José, 9.933 -84.0831^6^, 1156 m a.s.l. (UMM0203, 1♀, MCZ); Zurqui de Moravia, 10.0495 -84.0159^1^, 1600 m a.s.l. (UMM0762, 22.vii-8.viii.2010, 1♂, W Porras, BME); **Nicaragua: Granada**: Volcán Mombacho, non-organic coffee plantation, 12.4219 -86.5448^1^, 700–800 m a.s.l. (UMM0685, 2.vi.1998, 1♂, JM Maes, BME).

#### Ummidia varablanca sp. nov.

Taxon classificationAnimaliaAraneaeHalonoproctidae

2A24507E-E400-5024-A983-788EDF04CA9F

http://zoobank.org/6E49C034-87EB-40A4-9F6E-C833F38F0894

**Figure 59.**
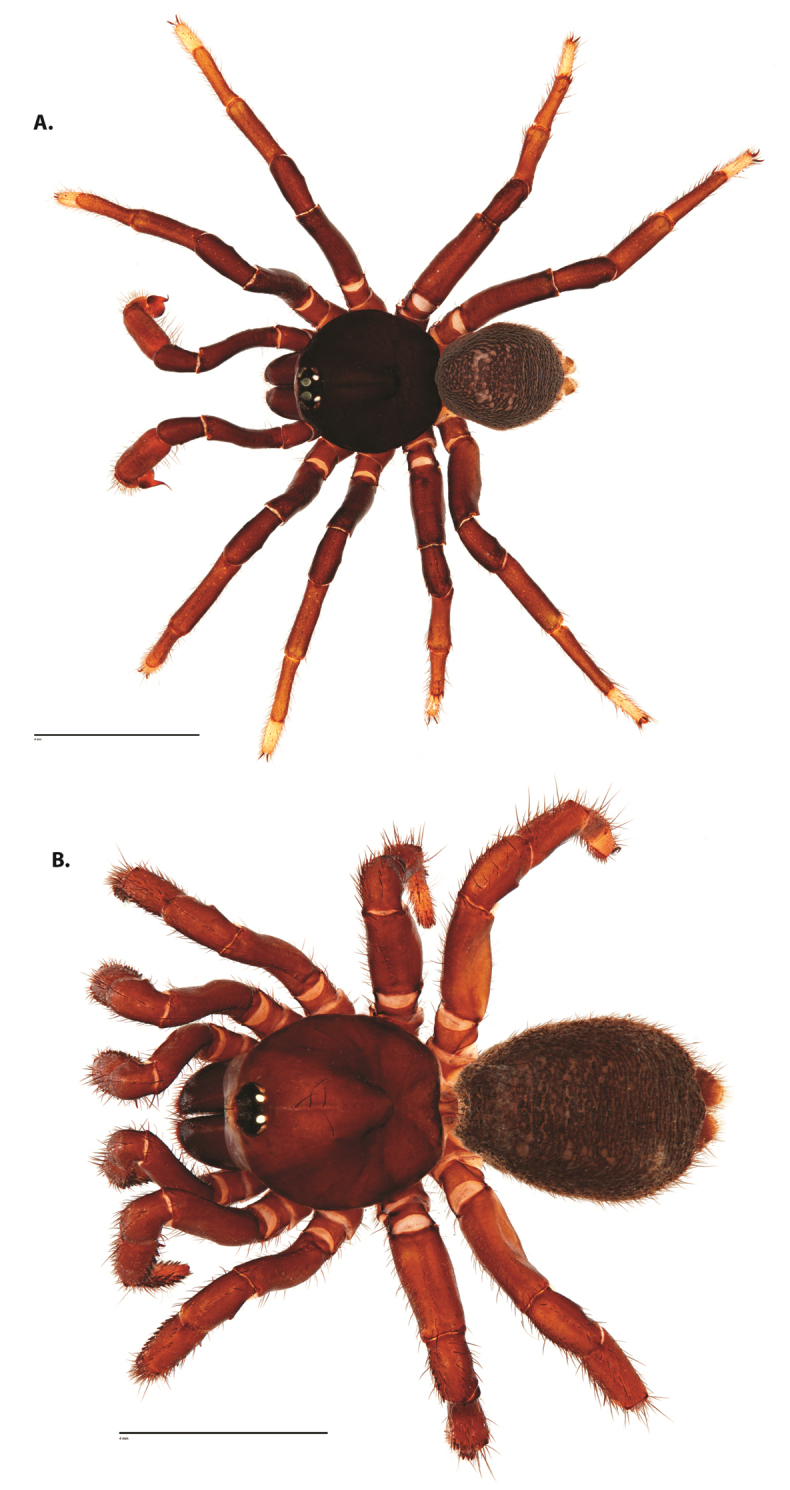
*Ummidia varablanca* sp. nov. from Heredia, Costa Rica. **A** male habitus illustration UMM669 **B** female habitus illustration UMM746. Scale bars: 4.0 mm.

**Figure 60.**
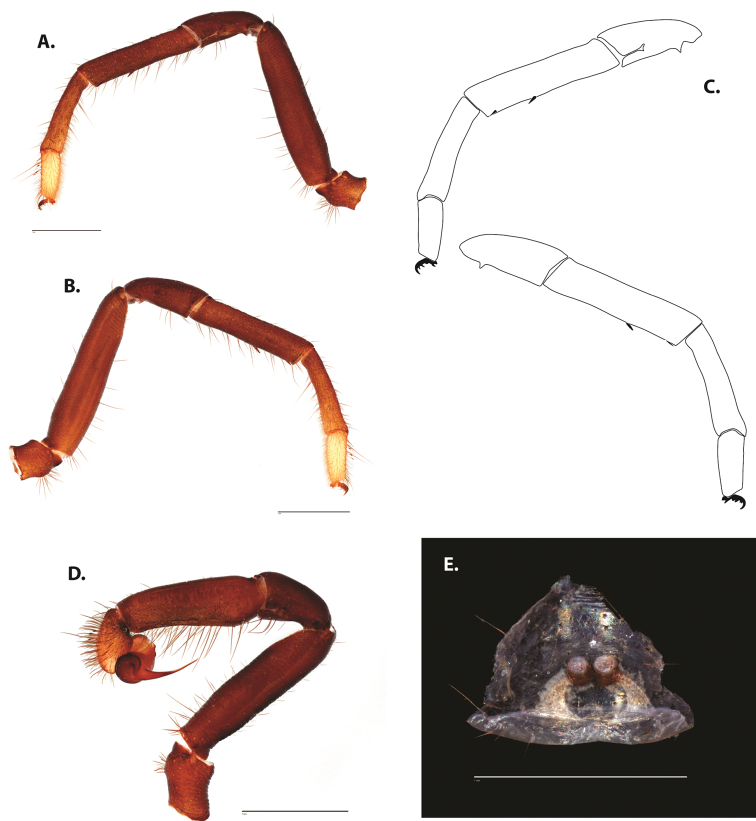
*Ummidia varablanca* sp. nov. from Heredia, Costa Rica **A–D** male holotype (UMM669) **A** prolateral aspect, leg I **B** retrolateral aspect, leg I **C** line drawings, leg I prolateral and retrolateral aspects **D** retrolateral aspect, pedipalp **E** cleared spermathecae female paratype (UMM746). Scale bars: 1.0 mm.

Figs 59 , 60 , Map 5

##### Type material.

HOLOTYPE: 1 ♂ (UMM669) from 9 kilometers east of Vara Blanca transect, Heredia, Costa Rica, 10.0024 -84.1216^1^, 1152 m a.s.l., 8.iii.2005, BME. PARATYPE: 1 ♀ (UMM746) from Alto Tapezco, San José, Costa Rica, 9.8982 -84.1553^1^, 2.viii.2001, coll. MA Zumbado, BME.

##### Etymology.

The specific epithet is a noun taken in apposition and is in reference to the type locality, Vara Blanca.

##### Diagnosis.

*Ummidia varablanca* are relatively small spiders (CL 3–4.8) with distinctly light tarsi. Males can be distinguished from *U. hondurena*, *U. yojoa*, *U. rugosa*, and *U. erema* by having a relatively short embolus with a single even curve and from all except *U. zebrina* and *U. yojoa* by lacking prolateral spines and having very few retrolateral spines (2 vs 10-44) on tibia I. Males can be further differentiated from *U. rugosa* and *U. riverai* by the lack of a pale dorsal heart patch on the abdomen. Females can be differentiated from all geographically proximate species by having spermathecae which curve medially then bend strongly anteriorly.

##### Description of male holotype.

*Specimen preparation and condition*. Specimen preserved in 80% EtOH. *General coloration*. Carapace, chelicerae, and legs reddish black 2.5YR 2.5/1, tarsi brownish yellow 10YR 6/8. Abdomen black 5YR 2.5/1. *Cephalothorax*. Carapace 3.35 long, 3.46 wide. Pars cephalica 2.44 long. Foveal groove procurved, 0.3 long, 0.6 wide. All eyes on defined tubercle. AER procurved. PER straight. Eye group 0.53 long, 1.03 wide, AME 0.27, PME 0.18, ALE 0.3, PLE 0.19. Sternum sparsely setose, STRl 1.94, STRw 2.05. Chelicerae with anterior tooth row comprising three teeth, posterior margin with five teeth. Palpal endites with mix of 21 small and large, hastate and non-hastate cuspules spread over proximal 4/5 of endite face, lacking distal endite cuspules, ENDw 0.71, ENDl 1.21. Labium with five cuspules, LBw 0.8, LBl 0.51. Rastellum with six fine spines along distal margin. Abdomen setose. *Legs*. F1 3.37; F1w 0.74; P1 1.66; Ti1 2.2; Mt1 1.65; Tr1 0.9; F3 2.54; F3w 0.88; P3 1.22; Ti3 1.63; Sd3 0.92; Mt3 1.4; Tr3 1.01; F4 3.16; F4w 0.83; P4 1.48; Ti4 1.97; Mt4 1.86; Tr4 0.98. Retrolateral face of tarsus IV without brush or comb. Leg I spination pattern: TSp 0, TSpv 0, TSrd 0, TSr 0, TSrv 2, MtSp 0, MtSr 0, TrSp 0, TrSr 0. *Pedipalps*. PTl 1.8, PTw 0.64, Bl 1.23. Embolus evenly curved.

##### Variation, males

**(n = 7).**CL 2.99–4.04, 3.45±0.12; CW 3.04–3.96, 3.5±0.11; Cap 2.08–2.82, 2.38±0.09; ENDl 0.46–0.63, 0.54±0.02; ENDw 0.83–1.19, 0.99±0.05; STRl 1.72–2.21, 1.93±0.06; STRw 1.64–2.17, 1.94±0.06; LBl 0.44–0.58, 0.51±0.02; LBw 0.67–0.85, 0.78±0.02; F1 2.82–3.87, 3.26±0.12; F1w 0.72–0.9, 0.81±0.02; P1 1.38–1.8, 1.56±0.05; Ti1 1.75–2.47, 2.03±0.1; Mt1 1.2–1.86, 1.47±0.09; Tr1 0.73–1.74, 0.94±0.14; F3 2.07–2.81, 2.42±0.09; F3w 0.78–1.04, 0.88±0.03; P3 1.03–1.37, 1.18±0.04; Ti3 1.24–1.77, 1.5±0.08; Mt3 1.12–1.5, 1.33±0.06; Tr3 0.78–1.09, 0.96±0.04; F4 2.71–3.66, 3.07±0.12; F4w 0.74–0.93, 0.84±0.02; P4 1.18–1.55, 1.37±0.05; Ti4 1.64–2.27, 1.87±0.08; Mt4 1.67–2.24, 1.87±0.08; Tr4 0.73–1.01, 0.93±0.04; TSp 0–8, 1.71±1.13; TSpv 0–6, 3±0.79; TSr 0–2, 0.57±0.3; TSrv 2–19, 10±1.9; PTl 1.28–2.09, 1.66±0.1; PTw 0.59–0.75, 0.66±0.02; BL 1.23–1.53, 1.34±0.04.

##### Description of female paratype.

*Specimen preparation and condition*. Specimen preserved in 80% EtOH. Spermathecae removed, cleared, in vial with specimen. *General coloration*. Carapace, chelicerae, and legs very dark brown 7.5YR 2.5/3, TrIV strong brown 7.5YR 5/8. Abdomen black 7.5YR 2.5/1, spinnerets dark yellowish brown 10YR 3/6. *Cephalothorax*. Carapace 4.03 long, 3.81 wide. Pars cephalica 3.07 long. Foveal groove procurved, 0.45 long, 0.96 wide. All eyes on moderate tubercle. AER procurved. PER straight. Eye group 0.6 long, 1.04 wide, AME 0.25, PME 0.2, ALE 0.36, PLE 0.27. Sternum sparsely setose, STRl 2.25, STRw 2.45. Chelicerae with anterior row comprising five teeth, posterior margin with six teeth. Palpal endites with 37 large cuspules across proximal 3/4 of endite face and ten smaller cuspules distally, ENDw 0.91, ENDl 1.56. Labium with seven large cuspules, LBw 0.98, LBl 0.64. Rastellum reduced with long rounded spines along margin. *Abdomen*. Evenly setose with pale speckles clustered at apodemes. *Legs*. F1 2.66; F1w 0.99; P1 1.58; Ti1 1.52; Mt1 1.08; Tr1 0.74; F3 2.37; F3w 1.15; P3 1.24; Ti3 1.27, Sd3 0.89; Mt3 0.93; Tr3 0.84; F4 2.86; F4w 1.15; P4 1.38; Ti4 1.51; Mt4 1.41; Tr4 0.9. Retrolateral face tarsus IV with comb of alternating long and short spinules. *Pedipalps*. PF 2.52, PP 1.34, PTi 1.38, PTr 1.38. Spermathecae curved medially and then bent anteriorly, bulbs facing anteriorly.

##### Variation, females

**(n = 3).**CL 3.96–4.83, 4.27±0.28; CW 3.81–4.32, 4.07±0.15; Cap 2.82–3.42, 3.1±0.17; ENDl 0.6–0.66, 0.64±0.02; ENDw 1.04–1.15, 1.08±0.04; STRl 2.25–2.82, 2.51±0.17; STRw 2.45–2.6, 2.52±0.04; LBl 0.64–0.73, 0.67±0.03; LBw 0.98–1.1, 1.03±0.04; F1 2.66–3.12, 2.87±0.13; F1w 0.99–1.12, 1.04±0.04; P1 1.58–1.88, 1.73±0.09; Ti1 1.52–1.77, 1.61±0.08; Mt1 1.08–1.22, 1.13±0.04; Tr1 0.74–0.86, 0.78±0.04; F3 2.37–2.68, 2.52±0.09; F3w 1.15–1.32, 1.23±0.05; P3 1.24–1.42, 1.36±0.06; Ti3 1.27–1.38, 1.34±0.03; Mt3 0.93–1.1, 1±0.05; Tr3 0.84–1.1, 0.95±0.08; F4 2.86–3.45, 3.07±0.19; F4w 1.15–1.37, 1.22±0.07; P4 1.38–1.79, 1.58±0.12; Ti4 1.51–1.88, 1.67±0.11; Mt4 1.41–1.65, 1.53±0.07; Tr4 0.9–1.09, 0.98±0.06; PF 2.52–2.82, 2.65±0.09; PP 1.34–1.63, 1.46±0.09; PTi 1.38–1.6, 1.5±0.07; PTr 1.38–1.67, 1.48±0.1.

##### Material examined.

**Costa Rica: Cartago**: El Copal, Pejibaye, 9.7838 -83.7489^1^, 992 m a.s.l. (UMM0751, vi.2006, 1♀, C Viquez, BME); Esperanzo del Gaurco, Tapanti National Park, Biological Station, 9.7585 -83.7843^1^, 1261 m a.s.l. (UMM0668, 3.v.2002, 1♂, BME); **Guanacaste**: Rio San Lorenzo, Tierras Morenas, 10.569 -85.0206^1^, 1500 m a.s.l. (UMM0681, 1995, 1♂, G Rodriguez, BME); **Heredia**: Heredia, 6 km ENE of Vara Blanca transect, 10.0024 -84.1216^1^, 1152 m a.s.l. (UMM0670, 9.iv.2002, 1♂, BME); Heredia, 9 km E of Vara Blanca transect, 10.0024 -84.1216^1^, 1152 m a.s.l. (UMM0669, Summer 2005, 1♂, BME); Vara Blanca, 10.1678 -84.1475^1^, 1876 m a.s.l. (UMM0750, 16.iii.2002, 1♀, BME); **Puntarenas**: Agujas, 8.5137 -83.5453^1^, 85 m a.s.l. (UMM0675, 1♂, BME); Monteverde, El Valle de Gutiérrez, 10.299 -84.803^1^, 1600 m a.s.l. (UMM0673, 5–7.x.2009, 1♂, BME); Monteverde, San Luis, Buen Amigo, 10.2721 -84.824^1^, 1300 m a.s.l. (UMM0674, ii.1995, 1♂, Z Fuentes, BME); **San José**: Alto Tapezco, 9.8982 -84.1553^1^, 1615 m a.s.l. (UMM0746, 1♀, BME).

#### Ummidia quepoa sp. nov.

Taxon classificationAnimaliaAraneaeHalonoproctidae

CB612683-080E-5302-9075-D30877C27500

http://zoobank.org/94DD790E-40BB-481B-868C-7670674A83EF

**Figure 61.**
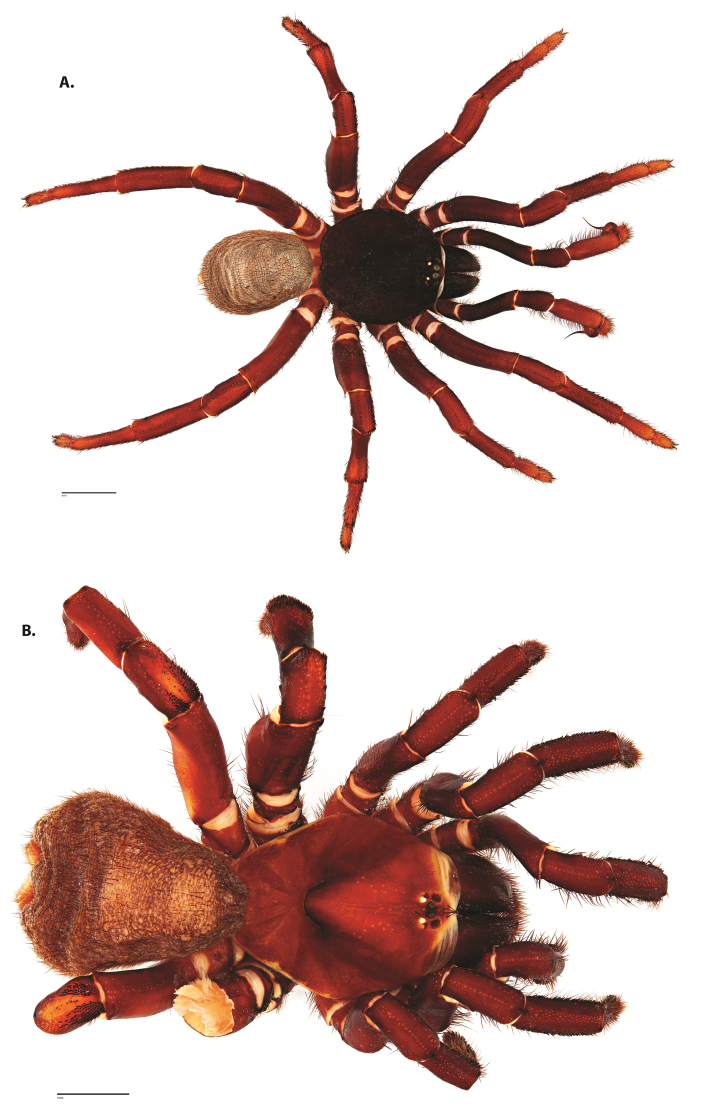
*Ummidia quepoa* sp. nov. from Quepos, Puntarenas, Costa Rica. **A** male habitus illustration UMM757 **B** female habitus illustration UMM210. Scale bars: 4.0 mm.

**Figure 62.**
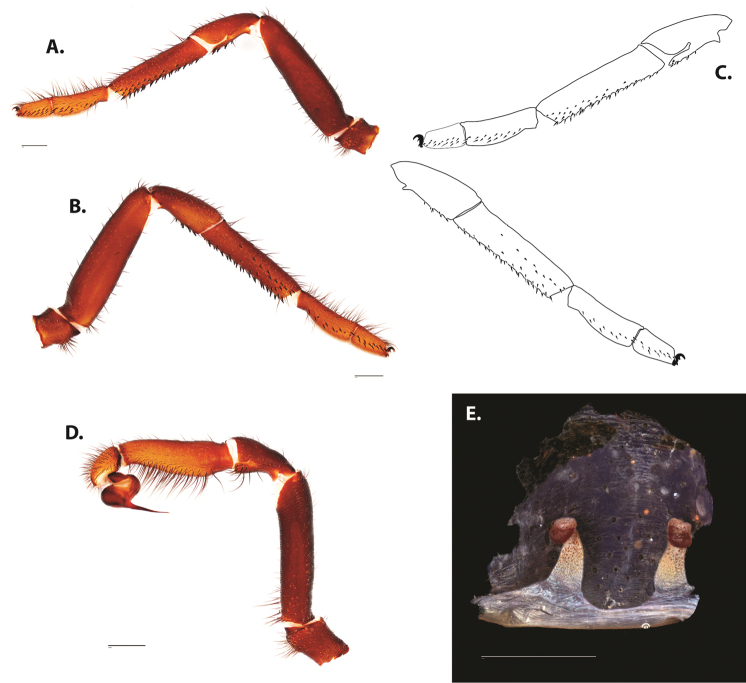
*Ummidia quepoa* sp. nov. from Quepos, Puntarenas, Costa Rica **A–D** male holotype (UMM757) **A** prolateral aspect, leg I **B** retrolateral aspect, leg I **C** line drawings, leg I prolateral and retrolateral aspects **D** retrolateral aspect, pedipalp **E** cleared spermathecae female paratype (UMM210). Scale bars: 1.0 mm.

Figs 61 , 62 , Map 5

##### Type material.

HOLOTYPE: 1 ♂ (UMM757) from Lucía Lutz farm, Quepos, Puntarenas, Costa Rica, 9.4828 -84.0948^1^, 102 m a.s.l., 26–28.v.2010, coll. A Solis BME.

##### Etymology.

The specific epithet is a noun taken in apposition and refers to the Quepoa, who inhabited the region of the type locality during the colonial era.

##### Diagnosis.

*Ummidia quepoa* are relatively large spiders (CL 8–13). Males can be distinguished from *U. hondurena*, *U. yojoa*, *U. rugosa*, and *U. erema* by having a relatively short embolus with a single even curve and from *U. yojoa* and *U. varablanca* by the presence of many prolateral (23 vs 0) and retrolateral (44 vs 2-3) spines on tibia I. Males can be further differentiated from *U. rugosa* and *U. riverai* by the lack of a pale dorsal heart patch on the abdomen, though occasionally with a faint opalescence. Females can be differentiated from all geographically proximate species by having spermathecae which curve laterally and then bend posteriorly and then anteriorly at the distal end.

##### Description of male holotype.

*Specimen preparation and condition*. Specimen preserved in 80% EtOH. *General coloration*. Carapace and chelicerae reddish black 2.5YR 2.5/1, legs dark reddish brown 5YR 3/4. Abdomen very dark brown 10YR 2/2. *Cephalothorax*. Carapace 8.81 long, 8.35 wide. Pars cephalica 6.14 long. Foveal groove procurved, 0.84 long, 1.77 wide. All eyes on moderate tubercle, highest under AME. AER procurved. PER slightly recurved. Eye group 0.99 long, 2.07 wide, AME 0.48, PME 0.26, ALE 0.48, PLE 0.39. Sternum sparsely setose around outer 1/3, STRl 5.06, STRw 4.78. Chelicerae with anterior tooth row comprising five teeth, posterior margin with seven teeth. Palpal endites with 22 small cuspules across proximal half of endite face, lacking distal endite cuspules, ENDw 1.93, ENDl 3.56. Labium with three cuspules, LBw 1.67, LBl 1.25. Rastellum with many spines on process. Abdomen setose. *Legs*. F1 7.47; F1w 1.91; P1 3.8; Ti1 5.03; Mt1 3.09; Tr1 1.48; F3 5.99; F3w 2.36; P3 3.11; Ti3 3.76; Sd3 2.31; Mt3 3.5; Tr3 2.33; F4 8.12; F4w 2.27; P4 3.51; Ti4 4.91; Mt4 5.87; Tr4 2.71. Retrolateral face of tarsus IV with loose comb surrounded in setae. Leg I spination pattern: TSp 11, TSpv 13, TSrd 0, TSr 2, TSrv 42, MtSp 11, MtSr 16, TrSp 11, TrSr 16. *Pedipalps*. PTl 4, PTw 1.37, Bl 2.88. Embolus evenly curved.

##### Variation, males

**(n = 4).**CL 8.15–10.57, 9.18±0.51; CW 7.75–10.11, 8.64±0.51; Cap 5.73–7.2, 6.21±0.34; ENDl 0.91–1.25, 1.07±0.08; ENDw 1.74–2.31, 2.06±0.12; STRl 4.71–6.19, 5.27±0.32; STRw 4.32–5.56, 4.77±0.28; LBl 1.22–1.6, 1.33±0.09; LBw 1.49–1.98, 1.7±0.1; F1 6.8–10.43, 8.38±0.8; F1w 1.88–2.39, 2.02±0.12; P1 3.65–5.18, 4.22±0.34; Ti1 4.58–7.31, 5.81±0.62; Mt1 2.83–4.84, 3.7±0.46; Tr1 1.26–1.81, 1.56±0.12; F3 5.53–7.22, 6.14±0.37; F3w 2.28–2.87, 2.45±0.14; P3 2.98–3.87, 3.26±0.2; Ti3 3.3–4.44, 3.78±0.24; Mt3 3.04–4.77, 3.71±0.37; Tr3 2.15–3.13, 2.5±0.22; F4 7.49–9.73, 8.41±0.47; F4w 2.04–2.6, 2.29±0.12; P4 3.21–4.23, 3.61±0.22; Ti4 4.76–6.57, 5.49±0.42; Mt4 5.2–7.45, 6.18±0.47; Tr4 2.41–3.19, 2.71±0.17; TSp 0–11, 7.5±2.6; TSpv 12–30, 20±4.45; TSr 1–2, 1.75±0.25; TSrv 25–57, 42.5±6.64; PTl 3.67–5.1, 4.4±0.34; PTw 1.27–1.61, 1.41±0.07; BL 2.39–3.38, 2.89±0.2.

##### Description of female paratype.

*Specimen preparation and condition*. Specimen preserved in 80% EtOH. Spermathecae removed, cleared, in vial with specimen. *General coloration*. Carapace, chelicerae, and legs very dusky red 10R 2.5/2. Abdomen very dark brown 10YR 2/2, spinnerets yellowish brown 10YR 5/6. *Cephalothorax*. Carapace 13.04 long, 11.21 wide. Pars cephalica 8.59 long. Foveal groove procurved, 1.6 long, 3.48 wide. Eye tubercle moderate under ME. AER procurved. PER straight. Eye group 1.41 long, 2.1 wide, AME 0.47, PME 0.32, ALE 0.73, PLE 0.38. Sternum sparsely setose, STRl 7.56, STRw 7.08. Chelicerae with anterior row comprising six teeth, posterior margin with five teeth. Palpal endites with 19 cuspules spread across proximal half of endite and 29 smaller cuspules distally, ENDw 3.14, ENDl 4.86. Labium with seven cuspules, LBw 2.66, LBl 1.85. Rastellum with many strong spines on process and up cheliceral face for ~ 2× length of process. *Abdomen*. Evenly setose with pale speckles concentrated at apodemes and with a dorsal light patch. *Legs*. F1 7.77; F1w 2.73; P1 4.97; Ti1 5.18; Mt1 3.6; Tr1 1.58; F3 6.05; F3w 3.32; P3 4.69; Ti3 3.77, Sd3 2.3; Mt3 3.31; Tr3 2.27; F4 7.93; F4w 3.11; P4 4.86; Ti4 4.66; Mt4 5.33; Tr4 2.36. Retrolateral face tarsus IV with brush 2–3 spinules deep and setose ventrally. *Pedipalps*. PF 7.28, PP 4.38, PTi 5.06, PTr 3.95. Spermathecae with lateral and then anterior bend, bulbs facing anterolaterally.

##### Variation, females

**(n = 2).**CL 10.75–12.1, 11.43±0.68; CW 9.38–10.69, 10.03±0.66; Cap 7.36–8.23, 7.8±0.44; ENDl 1.27–1.41, 1.34±0.07; ENDw 1.97–2.1, 2.04±0.07; STRl 6.66–8.01, 7.33±0.68; STRw 5.71–6.6, 6.15±0.45; LBl 1.78–1.84, 1.81±0.03; LBw 2.37–2.52, 2.45±0.07; F1 6.76–7.92, 7.34±0.58; F1w 2.36–2.82, 2.59±0.23; P1 4.1–4.83, 4.46±0.36; Ti1 4.34–5.26, 4.8±0.46; Mt1 2.99–3.49, 3.24±0.25; Tr1 1.52–1.63, 1.58±0.05; F3 5.77–6.62, 6.2±0.42; F3w 2.83–3.48, 3.16±0.33; P3 3.5–4.4, 3.95±0.45; Ti3 3.21–3.75, 3.48±0.27; Mt3 2.56–2.92, 2.74±0.18; Tr3 2.17–2.39, 2.28±0.11; F4 7.32–8.4, 7.86±0.54; F4w 2.77–3.31, 3.04±0.27; P4 3.87–4.57, 4.22±0.35; Ti4 3.96–4.63, 4.3±0.33; Mt4 4.34–5.42, 4.88±0.54; Tr4 1.93–2.53, 2.23±0.3; PF 6.11–7.34, 6.72±0.62; PP 3.63–4.36, 4±0.36; PTi 4.22–5.33, 4.78±0.56; PTr 3.19–3.93, 3.56±0.37.

##### Material examined.

**Costa Rica: Alajuela**: Guatuso, Cabanga, Finca de José Martínez, 10.59 -84.8528^1^, 478 m a.s.l. (UMM0764, 14.vi-14.vii.2010, 1♂, JA Azofeifa, BME); Upala, 10.898 -85.0195^1^, 300–400 m a.s.l. (UMM0683, 5.x-8.xii.2015, 1♂, C Chacoa, BME); **Puntarenas**: Aguirre, Finca de Lucía Lutz, 9.4828 -84.0948^1^, 102 m a.s.l. (UMM0757, 26–28.v.2010, 1♂, A Solis, BME); Agujas, 8.5794 -83.387^1^, 29 m a.s.l. (UMM0676, 1♂, BME); **San José**: San José, 9.933 -84.0831^6^, 1156 m a.s.l. (UMM0210, 1♀, N Banks, AMNH).

#### Ummidia cerrohoya sp. nov.

Taxon classificationAnimaliaAraneaeHalonoproctidae

054DA00E-686E-5DD0-BC2F-B5A0FD390D55

http://zoobank.org/52BBA1A7-D829-4F80-BEC3-9279C488F878

Fig. 63 , Map 5

##### Type material.

HOLOTYPE: 1 ♀ (UMM452) from the eastern slopes of the Cerro Hoya, Los Santos, Panama, 7.2986 -80.0014^6^, 940 m a.s.l., 6.iv.1966, coll. CW Myers, AMNH

##### Etymology.

The specific epithet is a noun taken in apposition and refers to the type locality, the eastern slopes of the Cerro Hoya.

##### Diagnosis.

*Ummidia cerrohoya* can be differentiated from all geographically proximate species by having very few (4 vs 20 or more) proximal endite cuspules. Females can be further distinguished from *U. hondurena*, *U. yojoa*, *U. rugosa*, *U. varablanca*, *U. quepoa*, and *U. erema* by having a distinct comb of long spinules on the retrolateral face of tarsus IV and from all geographically proximate species except *U. carlosviquezi* by having straight, simple spermathecae.

##### Description of female holotype.

*Specimen preparation and condition*. Specimen preserved in 80% EtOH. Spermathecae removed, cleared, in vial with specimen. *General coloration*. Carapace, chelicerae, and legs very dark brown 7.5YR 2.5/2. Abdomen black 5YR 2.5/1. *Cephalothorax*. Carapace 6.05 long, 5.58 wide. Pars cephalica 4 long. Foveal groove procurved, 0.45 long, 1.22 wide. All eyes on moderate tubercle. AER procurved. PER slightly procurved. Eye group 0.82 long, 1.33 wide, AME 0.27, PME 0.3, ALE 0.48, PLE 0.35. Sternum with posterior fringe, sparsely setose anteriorly, STRl 3.44, STRw 3.44. Chelicerae with anterior row comprising six teeth, posterior margin with nine teeth. Palpal endites with four large cuspules and 37 smaller cuspules distally, ENDw 1.4, ENDl 2.43. Labium with seven large cuspules, LBw 1.21, LBl 0.95. Rastellum with many strong spines on process and up cheliceral face for ~ 1× length of process. *Abdomen*. Evenly setose. *Legs*. F1 3.95; F1w 1.5; P1 2.42; Ti1 2.33; Mt1 1.61; Tr1 1.04; F3 3.24; F3w 1.75; P3 2.02; Ti3 1.84, Sd3 1.21; Mt3 1.57; Tr3 1.46; F4 4.31; F4w 1.73; P4 2.16; Ti4 2.47; Mt4 2.32; Tr4 1.35. Retrolateral face tarsus IV with defined comb of long spinules over length of tarsus. *Pedipalps*. PF 3.55, PP 1.97, PTi 2.16, PTr 1.98. Spermathecae simple, straight, bulbs facing anteriorly.

**Figure 63.**
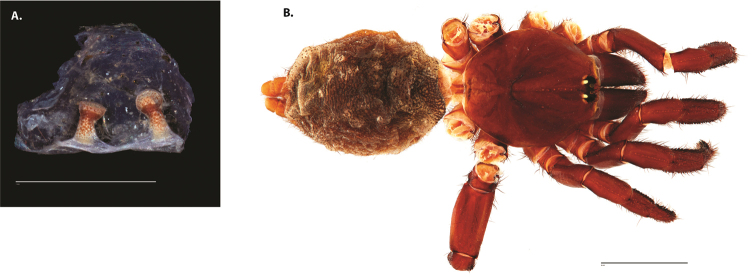
*Ummidia cerrohoya* sp. nov. from Los Santos, Panama. Female holotype (UMM452) **A** cleared spermathecae **B** female habitus illustration. Scale bars: 1.00 mm (**A**), 4.0mm (**B**).

##### Variation, females.

Known only from female holotype.

##### Males.

Unknown.

#### Ummidia erema

Taxon classificationAnimaliaAraneaeHalonoproctidae

(Chamberlin, 1925)

5AE70C78-7DEF-5846-B61D-5E37D962290C

**Figure 64.**
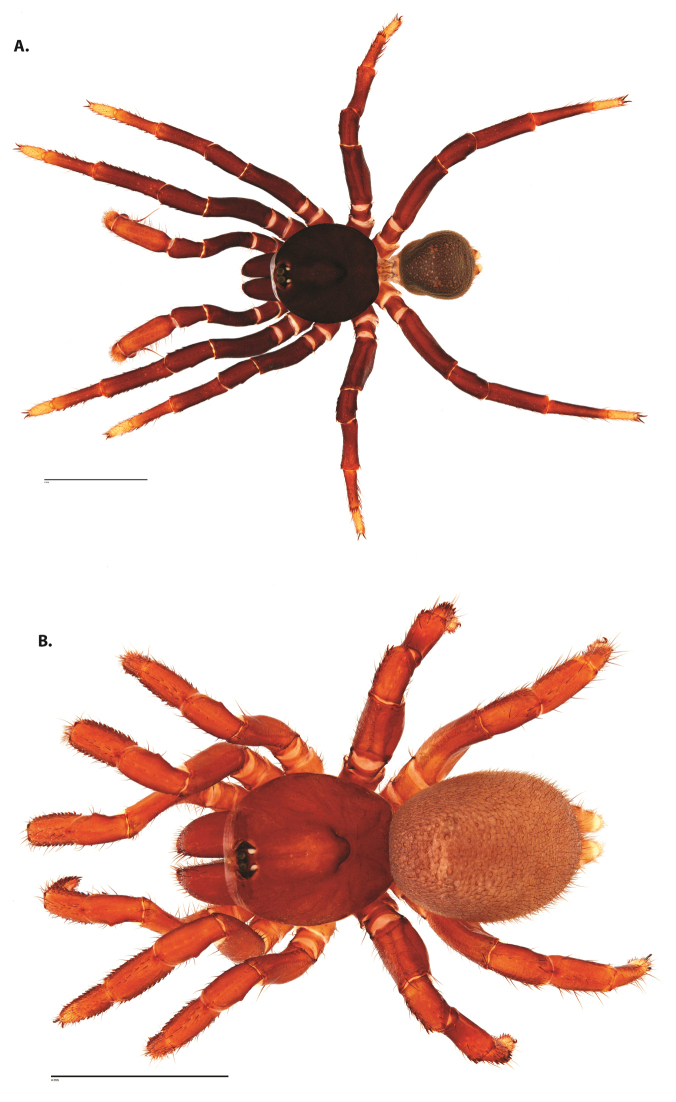
*Ummidia erema* (Chamberlin, 1925) from Barro Colorado Island, Panama. **A** male habitus illustration UMM535 **B** female habitus illustration MCZ16003. Scale bars: 4.0 mm.

**Figure 65.**
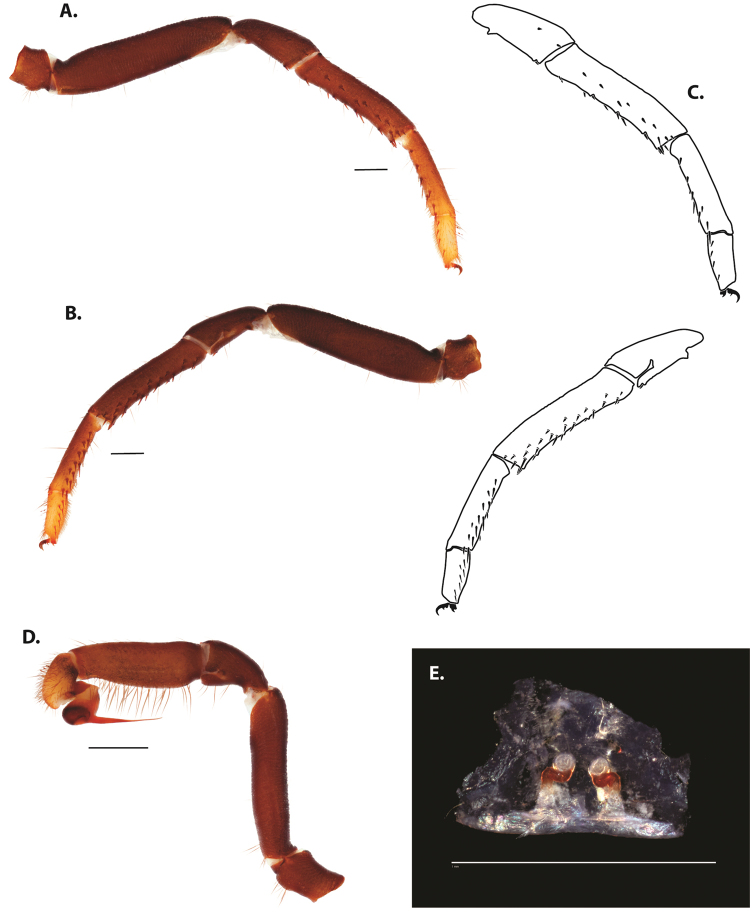
*Ummidia erema* (Chamberlin, 1925) from Barro Colorado Island, Panama. A-D male exemplar (UMM535) **A** prolateral aspect, leg I **B** retrolateral aspect, leg I **C** line drawings, leg I prolateral and retrolateral aspects **D** retrolateral aspect, pedipalp **E** cleared spermathecae female holotype (MCZ16003). Scale bars: 1.0 mm.

Figs 64 , 65 , Map 5

Pachylomerus eremus Chamberlin, 1925d: 211; HOLOTYPE: 1 ♀ (IZ16003) from Barro Colorado Island, Panama Canal Zone, Panama, 9.1520 -79.8467^5^, 156 m a.s.l., coll. WC Allee, deposited in the MCZ, examined.

##### Diagnosis.

*Ummidia erema* are relatively small spiders (CL 3.8–4.6) with distinctly light tarsi. Males can be distinguished from *U. zilchi*, *U. carlosviquezi*, *U. varablanca*, and *U. quepoa* by possessing a sinuous embolus, rather than one with an even single curve and from *U. yojoa* and *U. varablanca* by the presence of many prolateral (15 vs 0) and retrolateral (21 vs 2 or 3) spines on tibia I. Males can be further differentiated from *U. rugosa* and *U. riverai* by the lack of a pale dorsal heart patch on the abdomen. Females can be distinguished from all geographically proximate species except *U. hondurena* and *U. rugosa* by having spermathecae which bend first medially and then anteriorly.

##### Description of female holotype.

*Specimen preparation and condition*. Specimen preserved in 80% EtOH. Spermathecae removed, cleared, in vial with specimen *General coloration*. Carapace, chelicerae, and legs dark reddish brown 2.5YR 2.5/4, leg IV tarsi brownish yellow 10YR 6/8. Abdomen dark brown 2.5YR 3/2, spinnerets yellow 10YR 7/6. *Cephalothorax*. Carapace 3.79 long, 3.68 wide. Pars cephalica 2.83 long. Foveal groove procurved, 0.82 long, 0.3 wide. Eye tubercle moderate. AER procurved. PER relatively straight. Eye group 1 long, 0.58 wide, AME 0.22, PME 0.2, ALE 0.34, PLE 0.28. Sternum sparsely setose, thicker around anterior edges, STRl 2.32, STRw 2.25. Chelicerae with anterior row comprising three teeth, posterior margin with six teeth. Palpal endites with 27 large cuspules across proximal half of endite face and eleven cuspules distally, ENDw 0.95, ENDl 1.66. Labium with eight cuspules; five large and three small, LBw 0.9, LBl 0.63. Rastellum with six spines along distal cheliceral margin and five spines medially on process. *Abdomen*. Evenly setose. *Legs*. F1 2.88; F1w 0.99; P1 1.71; Ti1 1.66; Mt1 1.19; Tr1 0.82; F3 2.31; F3w 1.12; P3 1.33; Ti3 1.26, Sd3 0.87; Mt3 1; Tr3 0.98; F4 2.86; F4w 1.15; P4 1.46; Ti4 1.54; Mt4 1.19; Tr4 0.82. Retrolateral face tarsus IV with defined comb of alternating long and short spinules. *Pedipalps*. PF 2.66, PP 1.43, PTi 1.43, PTr 1.57. Spermathecae bent medially and then anteriorly, bulbs facing anteriorly.

##### Variation, females

**(n = 3).**CL 3.79–4.59, 4.16±0.23; CW 3.68–4.38, 3.95±0.21; Cap 2.83–3.31, 3.06±0.14; ENDl 0.58–0.65, 0.61±0.02; ENDw 0.98–1.06, 1.01±0.02; STRl 2.26–2.6, 2.39±0.11; STRw 2.25–2.67, 2.4±0.14; LBl 0.63–0.81, 0.74±0.06; LBw 0.9–1.05, 0.96±0.04; F1 2.51–3.11, 2.83±0.18; F1w 0.94–1.1, 1.01±0.05; P1 1.34–1.82, 1.62±0.15; Ti1 1.37–1.86, 1.63±0.14; Mt1 1.06–1.29, 1.18±0.07; Tr1 0.72–0.87, 0.8±0.04; F3 2.26–2.65, 2.4±0.12; F3w 1.12–1.27, 1.18±0.05; P3 1.18–1.5, 1.34±0.09; Ti3 1.25–1.38, 1.3±0.04; Mt3 0.92–1.12, 1.01±0.06; Tr3 0.98–1.09, 1.04±0.03; F4 2.78–3.24, 2.96±0.14; F4w 1.11–1.34, 1.2±0.07; P4 1.41–1.78, 1.55±0.12; Ti4 1.34–1.69, 1.52±0.1; Mt4 1.31–1.58, 1.45±0.08; Tr4 0.9–1.02, 0.95±0.04; PF 2.53–2.94, 2.71±0.12; PP 1.26–1.62, 1.44±0.1; PTi 1.42–1.58, 1.49±0.05; PTr 1.45–1.65, 1.56±0.06.

##### Description of male exemplar.

*Specimen preparation and condition*. Specimen preserved in 80% EtOH. Left palp and leg I removed, in vial with specimen. *General coloration*. Carapace, chelicerae, and legs reddish black 10R 2.5/1, tarsi reddish yellow 7.5YR 6/8. Abdomen black 5YR 2.5/1. *Cephalothorax*. Carapace 3.9 long, 3.83 wide. Pars cephalica 2.75 long. Foveal groove procurved, 0.31 long, 0.64 wide. Tubercle high. AER procurved. PER straight. Eye group 0.59 long, 1.03 wide, AME 0.35, PME 0.19, ALE 0.37, PLE 0.28. Sternum sparsely setose around outer 1/3, STRl 2.19, STRw 2.1. Chelicerae with anterior tooth row comprising five teeth, posterior margin with seven teeth. Palpal endites with 28 hastate cuspules of varying size across proximal 3/4 of endite face, lacking distal endite cuspules, ENDw 0.9, ENDl 1.5. Labium with ten cuspules, LBw 0.83, LBl 0.55. Rastellum greatly reduced with six small cuspules. Abdomen setose. *Legs*. F1 3.88; F1w 0.87; P1 1.88; Ti1 2.74; Mt1 1.83; Tr1 0.98; F3 2.95; F3w 1.07; P3 1.41; Ti3 1.72; Sd3 1.11; Mt3 1.46; Tr3 1.13; F4 3.74; F4w 0.98; P4 1.64; Ti4 2.19; Mt4 2.18; Tr4 1.23. Retrolateral face of tarsus IV lacking defined brush or comb. Leg I spination pattern: TSp 8, TSpv 7, TSrd 0, TSr 1, TSrv 20, MtSp 7, MtSr 13, TrSp 4, TrSr 7. *Pedipalps*. PTl 2.12, PTw 0.78, Bl 1.87. Embolus relatively long, slightly sinuous.

##### Variation, males.

Known only from male exemplar.

##### Material examined.

**Panama: Panama Canal Zone**: Barro Colorado Island, 9.152 -79.8467^5^, 156 m a.s.l. (MCZ1261, 1♀, WC Allee, MCZ) (UMM0514, 1946, 1♀, Zetek, AMNH) (UMM0512, 1.i.1947, 1♀, NLH Krauss, AMNH); (UMM0535, 18.iv.1946, 1♂, TC Schneirla, AMNH); Ft. San Lorenzo, 9.3227 -80.0014^4^, 15 m a.s.l. (UMM0513, 1juv, Zetek, AMNH).

### South America and the Caribbean

#### Ummidia quijichacaca sp. nov.

Taxon classificationAnimaliaAraneaeHalonoproctidae

4C77DF71-22B3-56E6-BC1E-F403ACDA341F

http://zoobank.org/36DF98D3-1C6D-43A0-85D2-61FFF3E03A8A

Fig. 66 , Map 6

##### Type material.

HOLOTYPE: 1 ♀ (UMM230) from Monserrate above Bogotá, Cundinamarca, Colombia, 4.6049 -74.0565^5^, 3152 m a.s.l., 27.iv.1965, coll. PR Craig, DL Craig, AMNH.

##### Etymology.

The specific epithet is a noun taken in apposition. It is the Muisca name for Monserrate, Quijichacaca, which translates to “grandmother’s foot”.

##### Diagnosis.

*Ummidia quijichacaca* can be differentiated from all geographically proximate species by having the carapace angular and longer than wide and legs which are darker distally. Females can be differentiated from *U. tibacuy*, *U. asperula*, and *U. neblina* by the presence of a defined comb of long spinules on the retrolateral face of tarsus IV. Females can be further distinguished from all geographically proximate species by having spermathecae which are straight with wide, flat, anteriorly facing bulbs.

**Figure 66.**
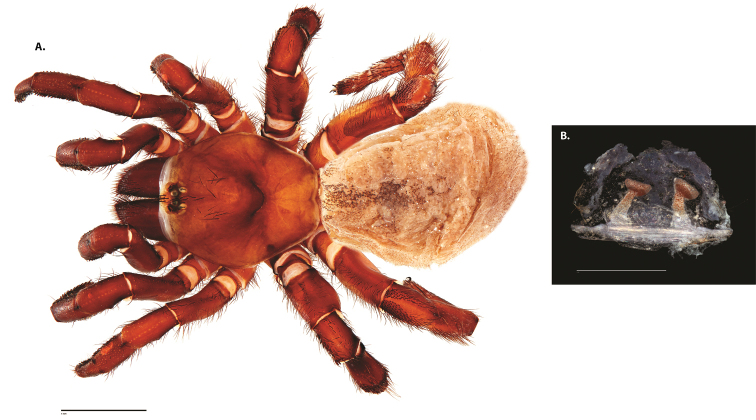
*Ummidia quijichacaca* sp. nov. female holotype (UMM230) from Monserrate, Colombia **A** female habitus illustration **B** cleared spermathecae. Scale bars: 4.0 mm (**A**), 1.0 mm (**B**).

##### Description of female holotype.

*Specimen preparation and condition*. Specimen preserved in 80% EtOH. Spermathecae removed, cleared, in vial with specimen. *General coloration*. Carapace, chelicerae, and legs dark reddish brown 5YR 3/4. Abdomen brown 7.5YR 4/2, spinnerets brownish yellow 10YR 6/6. *Cephalothorax*. Carapace 6.83 long, 6.24 wide. Pars cephalica 4.58 long. Foveal groove procurved, 0.46 long, 1.46 wide. All eyes on low tubercle. AER procurved. PER straight. Eye group 1.06 long, 1.54 wide, AME 0.35, PME 0.4, ALE 0.56, PLE 0.41. Sternum with posterior fringe, sparsely setose anteriorly, STRl 4.05, STRw 4.32. Chelicerae with anterior row comprising five teeth, posterior margin with six teeth. Palpal endites with 27 cuspules spread across proximal half of endite and 24 cuspules distally; equal in size to proximal cuspules anteriorly, smaller posteriorly, ENDw 1.57, ENDl 2.7. Labium with seven cuspules, LBw 1.7, LBl 1.24. Rastellum with many strong spines on process and up cheliceral face for ~ 2× length of process. *Abdomen*. Evenly setose. *Legs*. F1 4.67; F1w 1.63; P1 2.98; Ti1 2.87; Mt1 2.02; Tr1 1.06; F3 3.75; F3w 2.08; P3 2.38; Ti3 2.24, Sd3 1.55; Mt3 1.93; Tr3 1.44; F4 5.05; F4w 2.05; P4 2.69; Ti4 2.93; Mt4 3.1; Tr4 1.43. Retrolateral face tarsus IV with defined comb of long spinules over length of tarsus. *Pedipalps*. PF 4.22, PP 2.45, PTi 2.49, PTr 2.3. Spermathecae simple, straight, bulbs facing anteriorly.

##### Variation, females.

Known only from female holotype.

##### Males.

Unknown.

#### Ummidia tibacuy sp. nov.

Taxon classificationAnimaliaAraneaeHalonoproctidae

4B8F9691-3669-5010-B0FB-1BDDC6337ED4

http://zoobank.org/B41D7D1F-E8E4-4F46-B83D-D3F7AD184A27

Fig. 67 , Map 6

##### Type material.

HOLOTYPE: 1 ♀ (ICV-Av-1151) form the Reserva Qauinini Via a Nilo, Tibacuy, Cundinamarca, Colombia, 4.3283 -74.5081^5^, 1603 m a.s.l. coll. Catalina July 2018, NUCMNH.

##### Etymology.

The specific epithet is a noun taken in apposition and is in reference to the type locality.

##### Diagnosis.

*Ummidia tibacuy* females can be differentiated from all geographically proximate species by the presence of a comb of alternating long and short hairs on the retrolateral face of tarsus IV and by having spermathecae bend medially and then anteriorly with the bulbs coiled back onto the stalks, bulbs facing anteriorly. They can be further differentiated by the legs having a striped appearance due to the lightening of the leg articles near the joints and the tarsi being very light in color and by the carapace being wider than long and rounded.

**Figure 67.**
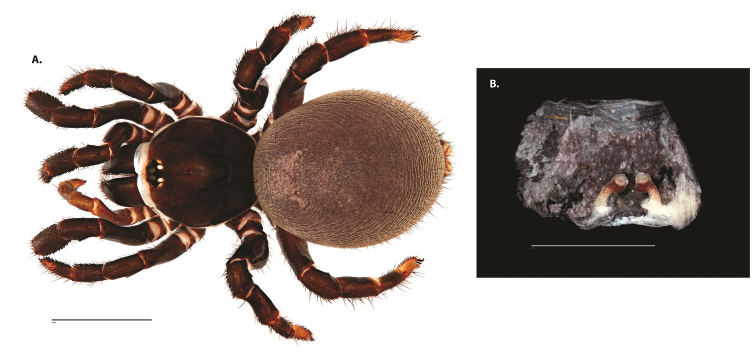
*Ummidia tibacuy* sp. nov. female holotype (AV1151) from Tibacuy, Colombia **A** female habitus illustration **B** cleared spermathecae. Scale bars: 4.0 mm (**A**), 1.0 mm (**B**).

##### Description of female holotype.

*Specimen preparation and condition*. Specimen preserved in 80% EtOH. Spermathecae removed, cleared, in vial with specimen. Left leg I removed, in vial with specimen *General coloration*. Carapace, chelicerae, and legs reddish black 2.5YR 2.5/1, tarsi brownish yellow 10YR 6/6. Abdomen black 7.5YR 2.5/1, spinnerets brownish yellow 10YR 6/6. *Cephalothorax*. Carapace 4.42 long, 4.56 wide. Pars cephalica 3.21 long. Foveal groove procurved, 0.24 long, 0.62 wide. Eye tubercle moderate. AER procurved. PER slightly procurved. Eye group 0.68 long, 1.1 wide, AME 0.27, PME 0.22, ALE 0.3, PLE 0.3. Sternum sparsely setose, thicker at edges, STRl 2.78, STRw 2.71. Chelicerae with anterior row comprising four teeth, posterior margin with six teeth. Palpal endites with 38 large cuspules across proximal 2/3 and 15 smaller cuspules distally, ENDw 1.09, ENDl 1.75. Labium with 13 large cuspules, LBw 0.95, LBl 0.75. Rastellum with rounded spines concentrated on distal/medial margins of process, otherwise with very long hairs. *Abdomen*. Evenly setose, hairs longer dorsally. *Legs*. F1 3.06; F1w 1.08; P1 1.81; Ti1 1.69; Mt1 1.17; Tr1 0.76; F3 2.58; F3w 1.32; P3 1.57; Ti3 1.36, Sd3 0.95; Mt3 1.05; Tr3 1.01; F4 3.08; F4w 1.36; P4 1.86; Ti4 1.6; Mt4 1.48; Tr4 0.85. Retrolateral face tarsus IV with defined comb of alternating long and short spinules. *Pedipalps*. PF 2.94, PP 1.26, PTi 0.5, PTr 0.11. Spermathecae with medial bend, bulbs facing anteriorly.

##### Variation, females.

Known only from female holotype.

##### Males.

Unknown.

#### Ummidia asperula

Taxon classificationAnimaliaAraneaeHalonoproctidae

(Simon, 1889)

ADA6C5F2-A273-5B9E-9C79-BF2DC8FEE7F1

**Figure 68.**
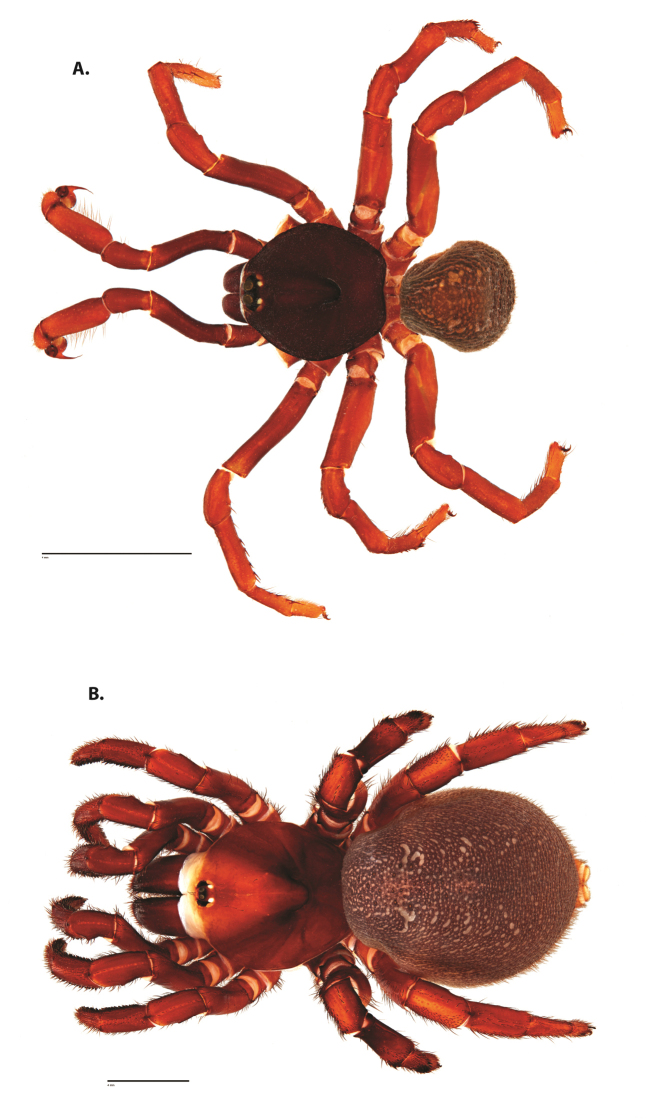
*Ummidia asperula* (Simon 1889) from Venezuela **A** male habitus illustration UMM577 **B** female habitus illustration AR4147. Scale bars: 4.0 mm.

**Figure 69.**
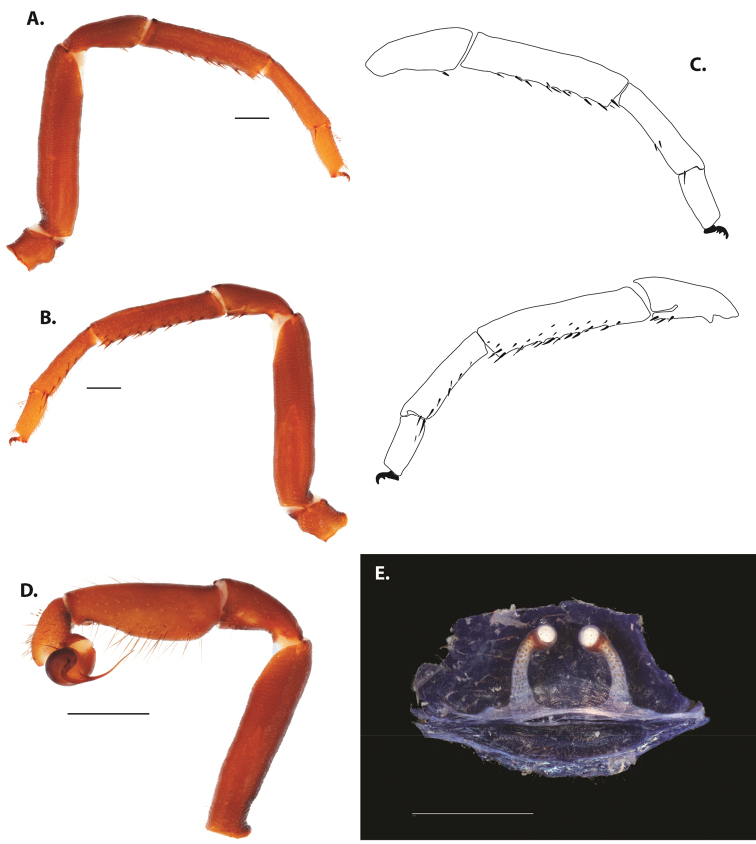
*Ummidia asperula* (Simon 1889) from Venezuela **A–D** male exemplar (UMM577) **A** prolateral aspect, leg I **B** retrolateral aspect, leg I **C** line drawings, leg I prolateral and retrolateral aspects **D** retrolateral aspect, pedipalp **E** cleared spermathecae AR4147. Scale bars: 1.0 mm.

Figs 68 , 69 , Map 6

Pachylomerus asperulus Simon, 1889: 179; HOLOTYPE 1 ♀ (AR4147) from Venezuela (label); from Catia La Mar near Caracas, north slopes of Silla near Maiquetía, Venezuela, 10.5778 -66.9973^5^, 261 m a.s.l. (Simon, 1889), deposited in MNHN, examined.

##### Diagnosis.

*Ummidia asperula* is a relatively small species which can be differentiated from all geographically proximate species by the lack of a distinct tarsal comb on the retrolateral face of tarsus IV in both males and females and from *U. erema*, *U. tibacuy*, and *U. neblina* by tarsi not being light in color. Males can be further distinguished from *U. erema* by the embolus being of moderate length with a single even curve, by having few prolateral spines on tibia I (3 vs 15). Females can be differentiated from all geographically proximate species except *U. salebrosa* by having narrow stalked spermathecae which curve slightly medially and then coil with bulbs facing dorsally. Females can be distinguished from *U. tunapuna* by having all eyes on a high, defined tubercle and by lacking a setal fringe on the lateral and posterior edges of the carapace.

##### Description of male exemplar.

*Specimen preparation and condition*. Specimen preserved in 80% EtOH. Left palp and leg I, right leg I removed, in vial with specimen. *General coloration*. Carapace and chelicerae reddish black 2.5YR 2.5/1, legs very dusky red 2.5YR 2.5/2, tarsi strong brown 7.5YR 5/8. Abdomen dark reddish gray 2.5YR 3/1. *Cephalothorax*. Carapace 3.69 long, 3.66 wide. Pars cephalica 2.62 long. Foveal groove procurved, 0.32 long, 0.69 wide. Tubercle high. AER procurved. PER slightly procurved. Eye group 0.6 long, 1.07 wide, AME 0.34, PME 0.18, ALE 0.37, PLE 0.21. Sternum sparsely setose around outer 1/3, STRl 2.05, STRw 1.96. Chelicerae with anterior tooth row comprising five teeth, posterior margin with six teeth. Palpal endites with 16 small subhastate cuspules spread across proximal half of endite face, lacking distal endite cuspules, ENDw 0.73, ENDl 1.41. Labium with nine cuspules, LBw 0.77, LBl 0.58. Rastellum with eight spines along distal cheliceral margin, three on process. Abdomen setose. *Legs*. F1 3.71; F1w 0.78; P1 1.61; Ti1 2.56; Mt1 1.65; Tr1 0.93; F3 2.64; F3w 0.91; P3 1.28; Ti3 1.56; Sd3 1.1; Mt3 1.33; Tr3 1.12; F4 3.37; F4w 0.88; P4 1.44; Ti4 1.97; Mt4 1.91; Tr4 1.05. Retrolateral face of tarsus IV without brush or comb. Leg I spination pattern: TSp 1, TSpv 2, TSrd 0, TSr 3, TSrv 23, MtSp 0, MtSr 8, TrSp 0, TrSr 1. *Pedipalps*. PTl 1.86, PTw 0.71, Bl 1.39. Embolus evenly curved.

##### Variation, males.

Known only from male exemplar.

##### Description of female holotype.

*Specimen preparation and condition*. Specimen preserved in 80% EtOH. Spermathecae removed, cleared, in vial with specimen. Left leg III removed, in vial with specimen. *General coloration*. Carapace, chelicerae, and legs dark reddish brown 2.5YR 2.5/4. Abdomen very dark brown 7.5YR 2.5/2, spinnerets yellowish brown 10YR 5/6. *Cephalothorax*. Carapace 7.61 long, 7.61 wide. Pars cephalica 5.75 long. Foveal groove procurved, 0.86 long, 1.7 wide. Eye tubercle high, defined, and with all eyes on and close set. AER procurved. PER procurved. Eye group 1.01 long, 1.39 wide, AME 0.34, PME 0.32, ALE 0.55, PLE 0.39. Sternum moderately setose, STRl 4.8, STRw 4.48. Chelicerae with anterior row comprising six teeth, posterior margin with seven teeth. Palpal endites with 21 cuspules spread across proximal half of endite and 19 cuspules distally, ENDw 1.86, ENDl 3.27. Labium with nine cuspules, LBw 1.75, LBl 1.24. Rastellum with many strong sines on process with heavy hairs on cheliceral face above process. *Abdomen*. Evenly setose with pale speckles concentrated at apodemes and slight lightening along the dorsal midline. *Legs*. F1 5.05; F1w 2; P1 3.3; Ti1 3.06; Mt1 2.01; Tr1 1.04; F3 4.48; F3w 2.35; P3 2.91; Ti3 2.75, Sd3 1.71; Mt3 1.82; Tr3 1.46; F4 5.44; F4w 2.24; P4 3.39; Ti4 3.2; Mt4 3.09; Tr4 1.64. Retrolateral face tarsus IV with loose comb of spinules surrounded in hairs. *Pedipalps*. PF 4.71, PP 2.9, PTi 2.85, PTr 2.52. Spermathecae with slight medial bend, small bulbs facing dorsally.

##### Variation, females.

Known only from female holotype.

##### Material examined.

**Venezuela**: Catia La Mar near Caracas, north slopes of Silla near Maiquetía, 10.5778 -66.9973^5^, 261 m a.s.l. (AR4147, 1889, 1♀, Simon, MNHN) **Bolivar**: Rancho Grande, 6.4142 -66.5169^9^, 188 m a.s.l. (UMM577, 13.v.1954, 1♂, W Beebe et. al., AMNH).

#### Ummidia neblina sp. nov.

Taxon classificationAnimaliaAraneaeHalonoproctidae

60396A10-C805-5C58-B52C-53DF66726450

http://zoobank.org/9DC5787E-EB11-459B-8660-1340AD850B22

Fig. 70 , Map 6

##### Type material.

HOLOTYPE: 1 ♀ (UMM141, AMNH) from Neblina Base Camp, Amazonas, Venezuela, 1.2395 -65.6675^6^, 547 m a.s.l., coll. R Stupakoff 24.ii.1984.

##### Etymology.

The specific epithet is a noun taken in apposition and is in reference to the type locality, Neblina Base Camp.

##### Diagnosis.

*Ummidia neblina* females can be differentiated from all geographically proximate species by the following combination of characters: retrolateral face of tarsus IV with a comb of alternating long and short spinules, tarsi light in color, and spermathecal stalks straight and medially tilted with wide bulbs.

**Figure 70.**
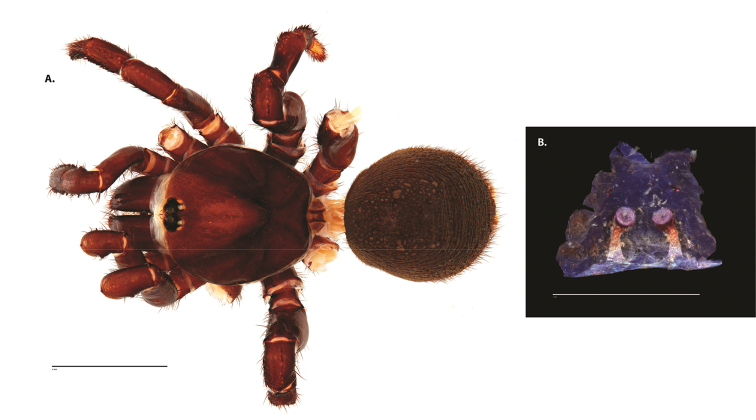
*Ummidia neblina* sp. nov. from Amazonas, Venezuela. Female holotype (UMM141) **A** female habitus illustration **B** cleared spermathecae. Scale bars: 4.0 mm (**A**), 1.0 mm (**B**).

##### Description of female holotype.

*Specimen preparation and condition*. Specimen preserved in 80% EtOH. Spermathecae removed, cleared, in vial with specimen. Many limbs removed all in vial with specimen. *General coloration*. Carapace, chelicerae, and legs very dark brown 10YR 2/2, Tr IV brownish yellow 10YR 6/8. Abdomen black 10YR 2/1, spinnerets yellowish brown 10YR 5/6. *Cephalothorax*. Carapace 4.74 long, 4.84 wide. Pars cephalica 3.43 long. Foveal groove procurved, 0.36 long, 0.99 wide. All eyes on moderate tubercle. AER procurved. PER procurved. Eye group 0.78 long, 1.2 wide, AME 0.27, PME 0.27, ALE 0.45, PLE 0.27. Sternum sparsely setose, STRl 2.91, STRw 2.95. Chelicerae with anterior row comprising five teeth, posterior margin with six teeth. Palpal endites with 38 large cuspules across proximal 4/5 of endite face and 35 smaller cuspules distally, ENDw 1.15, ENDl 1.98. Labium with 13 large cuspules, LBw 1.08, LBl 0.82. Rastellum reduced, large rounded spines medially and along margin with smaller spines above. *Abdomen*. Evenly setose with pale speckles. *Legs*. F1 3.39; F1w 1.26; P1 2.12; Ti1 1.99; Mt1 1.21; Tr1 0.86; F3 2.88; F3w 1.45; P3 1.78; Ti3 1.6, Sd3 1.13; Mt3 1.14; Tr3 1.1; F4 3.55; F4w 1.48; P4 1.96; Ti4 1.93; Mt4 1.69; Tr4 0.92. Retrolateral face tarsus IV with comb of alternating long and short spinules. *Pedipalps*. PF 3.03, PP 1.7, PTi 1.93, PTr 1.86. Spermathecae simple, tilted medially, bulbs facing anteriorly.

##### Variation, females.

Known only from female holotype.

##### Males.

Unknown.

#### Ummidia tunapuna sp. nov.

Taxon classificationAnimaliaAraneaeHalonoproctidae

2120B645-7C72-5AA4-85E5-41BD4F04A8E4

http://zoobank.org/EB375663-FDEC-493B-B214-505FB0EDD36C

Fig. 71 , Map 6

##### Type material.

HOLOTYPE 1: ♀ (UMM724, AMNH) from Tunapuna near Pax Guest House, St. George, Trinidad and Tobago, 10.6638 -61.3950^4^, 179 m a.s.l., 28–29.vi.1999, coll. P Miller, W Miller, G Stratton, B Suter, V Suter, AMNH.

##### Etymology.

The specific epithet is a noun taken in apposition and is in reference to the type locality, Tunapuna.

##### Diagnosis.

*Ummidia tunapuna* females can be differentiated from all other geographically proximate species by the presence of a setal fringe on the lateral and posterior margins of the carapace and by having spermathecal stalks relatively straight with a slight medial bend distally and bulbs facing anteromedially. Females can be further distinguished from *U. erema*, *U. tibacuy*, *U. asperula*, and *U. neblina* by the presence of a distinct comb of long spinules only on the retrolateral face of tarsus IV.

**Figure 71.**
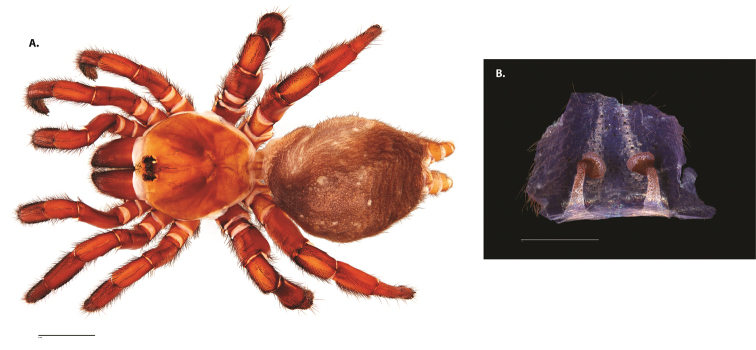
*Ummidia tunapuna* sp. nov. female holotype (UMM724) from St. George, Trinidad and Tobago **A** female habitus illustration, scale bar = 4.0 mm **B** cleared spermathecae, scale bar = 1.0 mm. Scale bars: 4.0 mm (**A**), 1.0 mm (**B**).

##### Description of female holotype.

*Specimen preparation and condition*. Specimen preserved in 80% EtOH. Spermathecae removed, cleared, in vial with specimen. *General coloration*. Carapace strong brown 7.5YR 3/4, chelicerae and legs dark reddish brown 5YR 3/4. Abdomen very dark brown 7.5YR 2.5/2, spinnerets dark yellowish brown 10YR 4/6. *Cephalothorax*. Carapace 7.89 long, 7.72 wide. Pars cephalica 5.4 long. Foveal groove procurved, 0.58 long, 2.04 wide. Eye tubercle low. AER procurved. PER slightly recurved. Eye group 1.1 long, 1.93 wide, AME 0.41, PME 0.34, ALE 0.67, PLE 0.46. Sternum sparsely setose around outer edges, thicker anteriorly, STRl 4.93, STRw 5.02. Chelicerae with anterior row comprising seven teeth, posterior margin with seven teeth. Palpal endites with 22 cuspules spread across proximal half of endite and 54 cuspules distally, equal in size to proximal cuspules anteriorly, smaller posteriorly, ENDw 1.99, ENDl 3.09. Labium with five cuspules, LBw 1.89, LBl 1.47. Rastellum with very many strong spines on process and along margin continuing up cheliceral face ~ 3 ´ length of rastellar process. *Abdomen*. Evenly setose with pale speckles concentrated into patches at apodemes. *Legs*. F1 5.01; F1w 1.64; P1 3.26; Ti1 3.08; Mt1 2.28; Tr1 1.34; F3 4.04; F3w 2.22; P3 2.8; Ti3 2.34, Sd3 1.58; Mt3 2.02; Tr3 2.11; F4 4.96; F4w 2.19; P4 2.77; Ti4 3.11; Mt4 3.03; Tr4 1.67. Retrolateral face tarsus IV with defined comb over 2/3 of article. *Pedipalps*. PF 4.48, PP 2.61, PTi 2.88, PTr 2.73. Spermathecae simple on relatively long, weak stalks, bulbs facing anteromedially.

##### Variation, females.

Known only from female holotype.

##### Males.

Unknown.

#### Ummidia salebrosa

Taxon classificationAnimaliaAraneaeHalonoproctidae

(Simon, 1892)

D8EF91FE-B648-5B76-8D52-687644B62C3B

Fig. 72 , Map 6

Pachylomerus salebrosus Simon, 1892: 530; HOLOTYPE: 1 ♀ (1894/300) from St Vincent, 13.2536 -61.187^6^, 863 m a.s.l., coll. HH Smith, deposited in NHMUK, examined.

##### Diagnosis.

*Ummidia salebrosa* females can be differentiated from all geographically proximate species except *U. asperula* by having narrow stalked spermathecae which curve slightly medially and then coil with bulbs facing dorsally. Females can be differentiated from *U. quijichacaca*, *U. asperula*, *U. tunapuna*, and *U. nidulans* by having very large palpal endite and labial cuspules.

**Figure 72.**
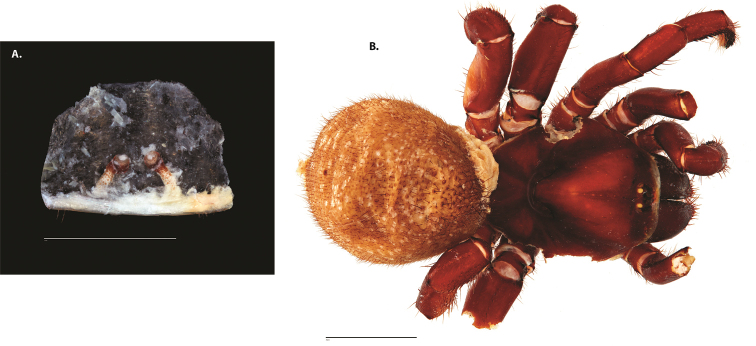
*Ummidia salebrosa* (Simon, 1892) female holotype (NHMUK1894) from St. Vincent **A** cleared spermathecae **B** female habitus illustration. Scale bars:1.0 mm (**A**), 4.0 mm (**B**).

##### Description of female holotype.

*Specimen preparation and condition*. Specimen preserved in 80% EtOH. Specimen in extremely poor condition, most legs missing below the femur. Spermathecae, removed, cleared, in vial with specimen. *General coloration*. Carapace, chelicerae, and legs dark reddish brown 2.5YR 2.5/3. Abdomen dark yellowish brown 10YR 3/4. *Cephalothorax*. Carapace 7.03 long, 6.63 wide. Pars cephalica 5.1 long. Foveal groove procurved, 0.74 long, 1.81 wide. Eye tubercle relatively high. AER procurved. PER relatively straight. Eye group 0.73 long, 1.39 wide, AME 0.32, PME 0.24, ALE 0.46, PLE 0.3. Sternum sparsely setose around outer edges, thicker anteriorly, STRl 4.43, STRw 4.13. Chelicerae with anterior row comprising three teeth, posterior margin with five teeth. Palpal endites with 26 very large cuspules spread across proximal half of endite face and 30 cuspules distally, ENDw 1.53, ENDl 2.93. Labium with eight very large cuspules, LBw 1.56, LBl 1.11. Rastellum with many strong spines on process. *Abdomen*. Evenly setose. *Legs*. F1 4.46; F1w 1.69; F3 3.88; F3w 2.05; P3 2.57; F4 5.17; F4w 2.11; P4 2.85; Ti4. Retrolateral face tarsus IV without comb or brush. *Pedipalps*. PF 4.05, PP 2.4, PTi 2.47, PTr 2.05. Spermathecae straight with distal coil, blubs facing dorsally.

##### Variation, females.

Known only from female holotype.

##### Males.

Unknown.

#### Ummidia nidulans

Taxon classificationAnimaliaAraneaeHalonoproctidae

(Fabricius, 1787)

633BACD6-C4C5-5929-B921-5FC0E8F919AF

Fig. 73 , Map 6

Aranea nidulans Fabricius, 1787: 343; female NEOTYPE designated herein (UMM297, deposited in AMNH) from Munro College, St. Elizabeth, Jamaica, 17.9244 -77.6869^4^, coll. R. Physick v.1959; original holotype presumably lost.
Mygale nidulans Latreille, 1804: 166.
Cteniza nidulans Sells, 1837: 207.
Cteniza venatoria Koch, 1838: 12.
Actinopus nidulans Westwood, 1840: 173.
Actinopus venatorius Koch, 1850: 75.
Pachylomerus nidulans Ausserer, 1871: 147.
Pachylomerus nidulans Simon, 1892: 87.
Ummidia nidulans Rudloff, 1996: 48.

##### Diagnosis.

*Ummidia nidulans* is a relatively large species (CL 11–13.5), which can be differentiated from *U. tibacuy*, *U. asperula*, and *U. neblina* by the presence of a defined comb of long spinules on the retrolateral face of tarsus IV. Females can be further distinguished from all geographically proximate species by having a large number of distal endite cuspules (> 90 vs 30-54) and spermathecae which are straight with rounded, anteriorly facing bulbs.

##### Female neotype.

*Specimen preparation and condition*. Specimen preserved in 80% EtOH. Spermathecae removed, cleared, in vial with specimen. *General coloration*. Carapace, chelicerae, and legs dark reddish brown 2.5YR 2.5/4. Abdomen black 5YR 2.5/1, spinnerets dark brown 7.5YR 3/4. *Cephalothorax*. Carapace 13.48 long, 12.72 wide. Pars cephalica 9.26 long. Foveal groove procurved, 1.42 long, 3.29 wide. Eye tubercle moderate under ME. AER procurved. PER slightly recurved. Eye group 1.3 long, 2.89 wide, AME 0.6, PME 0.44, ALE 0.68, PLE 0.58. Sternum sparsely setose, thicker anteriorly, STRl 7.9, STRw 8.3. Chelicerae with anterior row comprising 12 teeth, posterior margin with nine teeth. Palpal endites with 24 cuspules spread across proximal half of endite and 103 smaller cuspules distally, ENDw 3.28, ENDl 5.32. Labium with 14 cuspules, LBw 2.77, LBl 1.86. Rastellum with many strong spines on process, none along margin, with small spines above process. *Abdomen*. Evenly setose with pale speckles forming patches at apodemes, lighter dorsally. *Legs*. F1 7.41; F1w 2.81; P1 5.11; Ti1 4.57; Mt1 4.47; Tr1 2.42; F3 6.8; F3w 3.47; P3 4.61; Ti3 4.16, Sd3 2.6; Mt3 2.72; Tr3 2.26; F4 7.83; F4w 3.44; P4 4.57; Ti4 4.57; Mt4 4.47; Tr4 2.42. Retrolateral face tarsus IV with defined comb of long spinules over length of tarsus. *Pedipalps*. PF 6.57, PP 4.14, PTi 4.44, PTr 3.68. Spermathecae simple on relatively long, weak stalks, bulbs facing anteriorly.

**Figure 73.**
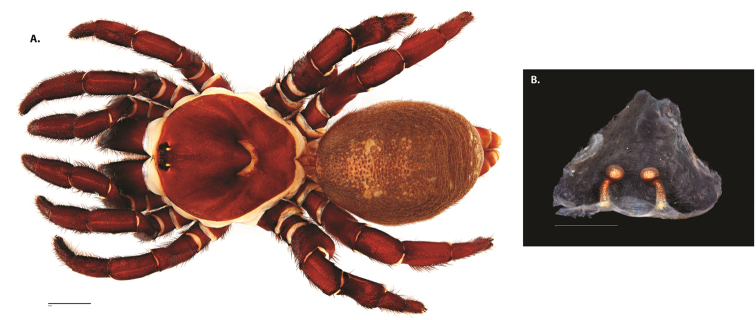
*Ummidia nidulans* (Fabricius, 1787) from St. Elizabeth, Jamaica. Female neotype (UMM297) **A** female habitus illustration **B** cleared spermathecae. Scale bars: 4.0 mm (**A**), 1.0 mm (**B**).

##### Variation, females.

**(n = 5)**CL 4.95–13.48, 11.14±1.59; CW 4.65–12.72, 10.43±1.5; Cap 3.75–10.11, 8.02±1.12; ENDl 0.68–1.39, 1.16±0.13; ENDw 1.15–2.89, 2.28±0.31; STRl 2.98–8.12, 6.7±0.96; STRw 2.98–8.3, 6.02±1.18; LBl 1.07–2.19, 1.83±0.2; LBw 1.22–3.11, 2.49±0.34; F1 3.23–8.04, 6.58±0.88; F1w 1.28–3, 2.5±0.31; P1 2.05–5.25, 4.32±0.59; Ti1 1.91–5.41, 4.28±0.62; Mt1 1.18–3.75, 2.89±0.45; Tr1 0.92–1.96, 1.61±0.19; F3 2.75–7, 5.8±0.78; F3w 1.58–3.65, 3.1±0.39; P3 1.87–4.74, 3.9±0.53; Ti3 1.55–4.21, 3.42±0.5; Mt3 1.13–3.14, 2.46±0.35; Tr3 1.12–3.13, 2.28±0.34; F4 3.53–8.34, 7.03±0.89; F4w 1.57–3.65, 3.01±0.38; P4 1.94–4.78, 3.99±0.53; Ti4 2.04–5.02, 4.1±0.55; Mt4 1.81–4.57, 3.79±0.53; Tr4 1.21–3.01, 2.29±0.31; PF 2.88–7.44, 5.91±0.8; PP 1.71–4.24, 3.58±0.48; PTi 1.69–4.72, 3.85±0.56; PTr 1.59–4.28, 3.36±0.47.

##### Males.

Unknown.

##### Material examined.

**Jamaica**: 18.1192 -77.2977 9, 311 m a.s.l. (UMM0476, 13.viii.1923, 1♀, JT Lloyd, AMNH); **Clarendon**: Mosley Hall, 18.108 -77.2986 9, 309 m a.s.l. (UMM0305, i.1966, 1♀, R Kern, AMNH); **Portland**: Cinchona, 18.1056 -76.68 6, 1553 m a.s.l. (UMM0201, 1♀, CT Brues, MCZ); **St. Elizabeth**: Munro College, 17.9244 -77.6869 4, 774 m a.s.l. (UMM0297, 1–31.v.1959, 1♀, R Physick, AMNH); AMNH).

#### Ummidia glabra

Taxon classificationAnimaliaAraneaeHalonoproctidae

(Doleschall, 1871)

1EE2BF7E-2BFD-57DF-ADD2-2EF151ABF43E

##### Note.

*Pachylomerus glaber* Doleschall, in Ausserer, 1871: 146; female HOLOTYPE (AR5) from Brazil, coll. Natterer vii.1818 deposited NHMV. This specimen is in such delicate condition that it could not be shipped. We were able to examine photos and conclude that the holotype is, in fact, correctly identified as *Ummidia*. However, having no other collected material from the region we are unable to provide a description for this species.

## Supplementary Material

XML Treatment for Ummidia

XML Treatment for Ummidia audouini

XML Treatment for Ummidia neilgaimani

XML Treatment for Ummidia gingoteague

XML Treatment for Ummidia carabivora

XML Treatment for Ummidia rongodwini

XML Treatment for Ummidia okefenokee

XML Treatment for Ummidia richmond

XML Treatment for Ummidia macarthuri

XML Treatment for Ummidia beatula

XML Treatment for Ummidia colemanae

XML Treatment for Ummidia funerea

XML Treatment for Ummidia rosillos

XML Treatment for Ummidia mercedesburnsae

XML Treatment for Ummidia paulacushingae

XML Treatment for Ummidia modesta

XML Treatment for Ummidia waunekaae

XML Treatment for Ummidia gertschi

XML Treatment for Ummidia timcotai

XML Treatment for Ummidia gabrieli

XML Treatment for Ummidia pesiou

XML Treatment for Ummidia rodeo

XML Treatment for Ummidia pustulosa

XML Treatment for Ummidia huascazaloya

XML Treatment for Ummidia anaya

XML Treatment for Ummidia cuicatec

XML Treatment for Ummidia brandicarlileae

XML Treatment for Ummidia zebrina

XML Treatment for Ummidia riverai

XML Treatment for Ummidia frankellerae

XML Treatment for Ummidia zilchi

XML Treatment for Ummidia hondurena

XML Treatment for Ummidia yojoa

XML Treatment for Ummidia matagalpa

XML Treatment for Ummidia rugosa

XML Treatment for Ummidia carlosviquezi

XML Treatment for Ummidia varablanca

XML Treatment for Ummidia quepoa

XML Treatment for Ummidia cerrohoya

XML Treatment for Ummidia erema

XML Treatment for Ummidia quijichacaca

XML Treatment for Ummidia tibacuy

XML Treatment for Ummidia asperula

XML Treatment for Ummidia neblina

XML Treatment for Ummidia tunapuna

XML Treatment for Ummidia salebrosa

XML Treatment for Ummidia nidulans

XML Treatment for Ummidia glabra
